# Mechanochemical Synthesis of Fluorinated Imines

**DOI:** 10.3390/molecules27144557

**Published:** 2022-07-17

**Authors:** Karolina Ciesielska, Marcin Hoffmann, Maciej Kubicki, Donata Pluskota-Karwatka

**Affiliations:** Faculty of Chemistry, Adam Mickiewicz University in Poznań, Uniwersytetu Poznańskiego 8, 61-614 Poznań, Poland; karcie5@amu.edu.pl (K.C.); mmh@amu.edu.pl (M.H.); mkubicki@amu.edu.pl (M.K.)

**Keywords:** mechanochemistry, imines, fluorine, manual grinding, Schiff’s bases

## Abstract

A number of imines, including 12 new compounds, previously not reported in the literature, derived from variously fluorinated benzaldehydes and different anilines or chiral benzylamines were synthesized by a solvent-free mechanochemical method, which was based on the manual grinding of equimolar amounts of the substrates at the room temperature. In a very short reaction time of only 15 min, the method produced the expected products with good-to-excellent yields. The yields were comparable or significantly higher than those reported in the literature for the imines synthesized by other methods. Importantly, the conditions used for the reactions with aniline derivatives also resulted in the high yields of imines obtained from chiral benzylamines, and can be extended to the synthesis with other similar amines. Structures of all imines were confirmed by NMR spectroscopy: ^1^H, ^13^C and ^19^F. For four compounds, X-ray structures were also obtained. The synthetic approach presented in this paper contributes to the prevention of environmental pollution and can be easily extended for larger-scale syntheses. The mechanochemical solvent-free method provides a convenient strategy particularly useful for the preparation of fluorinated imines being versatile intermediates or starting material in the synthesis of drugs and other fine chemicals.

## 1. Introduction

Imines, also called Schiff’s bases, are an important group of chemicals widely used in organic synthesis both as intermediates and starting materials. The high reactivity of imines results from the presence of the multifunctional C = N bond in their structure, which is able to undergo a wide spectrum of chemical transformations, including reduction, condensation, cyclisation, cycloaddition, nucleophilic addition as well as multicomponent reactions, leading to the formation of various biologically and chemically relevant products. As ligands readily complexing the metals of the *d* and *f* blocks, imines are also valuable reagents in coordination chemistry [1,2,3,4,5]. The diversity of compounds containing the azomethine unit shows that the synthetic potential of imines both as substrates and intermediates is indeed great.

Due to its ability to undergo interactions with a wide range of biological targets, the imine function is an essential pharmacophore in nitrogen-containing bioactive compounds [6,7,8,9,10]. There are many different pharmacophores. Among them, one of the most important is fluorine [11]. Fluorination seems to be a standard strategy for modulating the properties of chemical compounds and plays an important role in providing therapeutic agents [12,13]. The pharmacological potential of fluorinated compounds results from the fact that the replacement of hydrogen atoms with fluorine ones does not often violate the molecule conformation; however, due to the fluorine electron-withdrawing inductive effect, may significantly change the chemical and biological properties of the parental molecules [14,15]. This may, in turn, influence interactions with biological targets as well as the metabolism of drugs.

Various approaches to the preparation of fluorine-containing imines are reported in the literature. Here, a few examples are presented. The condensation of α-keto ester, ethyl 3,3,3-trifluoropyruvate, with generated in situ salt of benzylamines with acetic or formic acid, resulted in the regioselective synthesis of the corresponding imines [16]. The reactions were conducted at reflux in the boiling organic solvent (dichloromethane or toluene) for 6 to 64 h, and required the isolation of products by column chromatography [16]. The synthesis of fluorine-containing *Pinus* diterpenic imines, the insect attractants based on naturally occurring *Pinus* diterpenic resin acids, was achieved by the condensation of dehydroabietylamine with *metha*-fluoro or *para*-trifluoromethyl benzaldehydes [17]. Three imines as pre-ligand compounds were synthesized by the classical method: a mixture of *ortho*-fluorobenzaldehyde with *para*-fluoroaniline or *para*-methoxyaniline was stirred in *n*-hexane in the presence of MgSO_4_ for 2 h leading to the formation of the expected products [18]. A series of imines derived from 2,3,4,5,6-pentalfuorobenzaldehyde or 2,6-difluorobenzaldehyde and anilines or other aromatic amines were prepared in ethanol and recrystallized in pentane, diethyl ether or THF [19]. A few fluorine-containing imines were also obtained from the reactions of appropriate benzylamines with arylamines in the presence of an iridium catalyst [20]. Fluorine-containing imines were also synthesized by the acid-catalyzed condensation of 3-hydroxybenzaldehyde and various fluorinated anilines [21]. Apart from being target compounds, fluorinated imines are also used as substrates in the preparation of other fine chemicals, for example, fluorine-containing chiral amines, which, in medicinal chemistry, play the role of important building blocks. Although fluorinated imines are perceived as convenient and versatile starting materials for the synthesis of different functionalized amines, they may be difficult to obtain [22].

Despite the enormous effort that has already been made to improve the existing synthetic methods, due to the great importance of imines, new methodologies for their preparation are constantly being developed. When developing them, more and more attention is being paid to the principles of green and sustainable chemistry as well as mechanochemistry. Mechanochemical methodologies offer advantages over conventional synthetic routes with regard to the amount of solvents used and the energy consumed, and provide more efficient organic waste management; thus, they are becoming popular techniques for organic synthesis [23,24,25]. The preparation of imines by the mechanochemical approach has already been reported [26,27,28,29,30]. Synthetic protocols required the use of mills [27,28,29,30], catalytic amounts of iodine [27], workup of the obtained products [27,28] and long milling times (60 [30] or 90 [28] min). Regarding the fluorine-containing imines, we found only one study on the mechanochemical strategy used for the synthesis of such compounds. The imines were monofluorinated in one of the two phenyl rings present in their structures, and were prepared from derivatives of benzaldehyde and aniline [31]. However, the method involved the use of small quantities of organic solvent and yields of the products were not reported [31].

Being aware of the importance of imines and surprised to find only a few papers related to the mechanochemical methodology of the synthesis of these compounds, we decided to investigate the usefulness of this strategy for the preparation of fluorine-containing imines. These compounds subjected to the Pudovik reaction enable to obtain the fluorinated α-aminophospfonates in which our laboratory is particularly interested. Therefore, herein, we report the mechanochemical synthesis of four series of fluorine-containing imines derived from the reactions of 2-fluoro-, 2,4-difluoro-, 2,4,6-trifluoro- and 2,3,4,5,6-pentafluorobenzaldehyde with the chiral amines: (*R*)-(+)-4-methoxy-α-methylbenzylamine, (*R*)-(+)-α-methylbenzylamine, and a range of aniline derivatives having electron-donating or electron-withdrawing substituents in different positions of the aromatic ring.

## 2. Results and Discussion

**Scheme 1:**
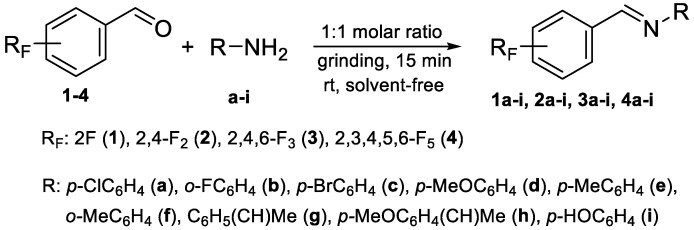
Machanochemical approach to fluorine-containing imines formed in the reactions of fluorinated aldehydes with various aromatic amines or chiral benzylamines.

Four differently fluorinated benzaldehydes subjected to mechanochemical reactions with aromatic amines or chiral benzylamines resulted in the formation of the corresponding imines (Scheme 1, Table 1).

The synthetic method was based on the manual grinding of equimolar amounts of the reagents and was used for the substrates being in a solid state (aldehydes: **3**, **4** and amines: **a**, **c**, **d**, **e, i**, Table 1) as well as in a liquid state (compounds: **1**, **2**, **b**, **f**, **g**, **h**, Table 1). In order to study the scope of the reaction, activated amines, having electron-donating groups, as well as deactivated amines, containing electron-withdrawing substituents, were used (Table 1). The reagents were ground for 15 min. This time was selected as suitable for all types of substrates used in our studies. For activated amines, 10 min was sufficient. However, this was not enough in the case of deactivated amines. Prolonged grinding (20 min) did not result in a better yield. Therefore, in order to standardize the reaction time and to develop the most general method possible, we chose 15 min, bearing in mind that fine tuning of the reaction conditions is very likely for each individual combination of substrates.

The products were obtained in good-to-excellent yields (68–99%, Table 1) with three outliers, **2b**, **3b** and **3i**, whose yields were, respectively, 58%, 56% and 45% only. To improve the yield, the synthesis with an auxiliary additive (K_2_CO_3_) was attempted in the case of **2b**. However, any yield benefits were observed; therefore, no more reactions were performed this way. Most of the 36 crude products did not contain substrates, indicating their full conversion. These imines were directly subjected to structural studies. In the ^1^H, ^13^C and ^19^F NMR spectra obtained for these compounds, no side products or unreacted substrates could be detected (for the NMR spectra of all synthesized imines, see ESI, Figures S1–S108). The yields of these imines were calculated on the basis of the crude products masses. When the monitoring of the reaction progress showed the presence of unreacted starting material (amine) among the product formed, the reaction yield was calculated from the ^1^H NMR spectrum by an internal standard method and was marked with “*” (Table 1). The synthetic method should provide analytically pure products; therefore, we developed a very rapid, easy and simple procedure for purification of imines in case of incomplete reactions. The procedure was based on the filtration of the mixture solution through a 3 cm thick layer of silica gel placed on the foam funnel. This resulted in the separation of the product from traces of unreacted amine with no loss of the yield (Table 2, entries: 2 and 4), with a slightly lower yield (Table 2, entries: 1, 3, 5, 6 and 7) or with a lower yield of about 10% (Table 2, entries: 8–10).

**Figure 1:**
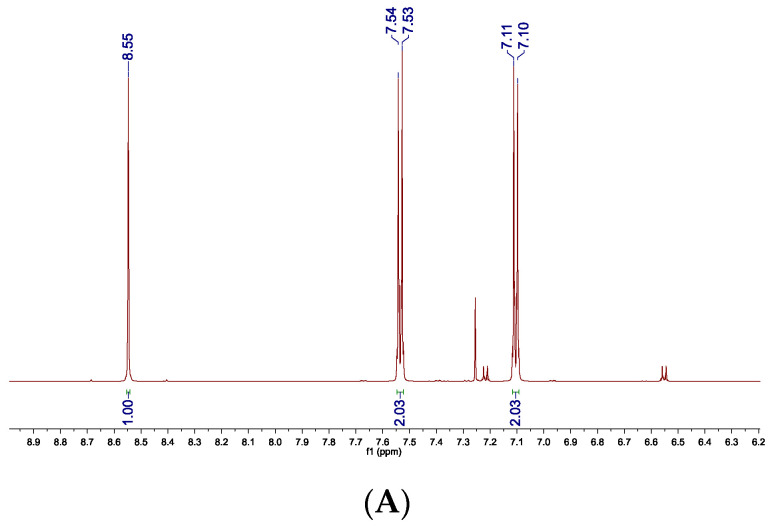

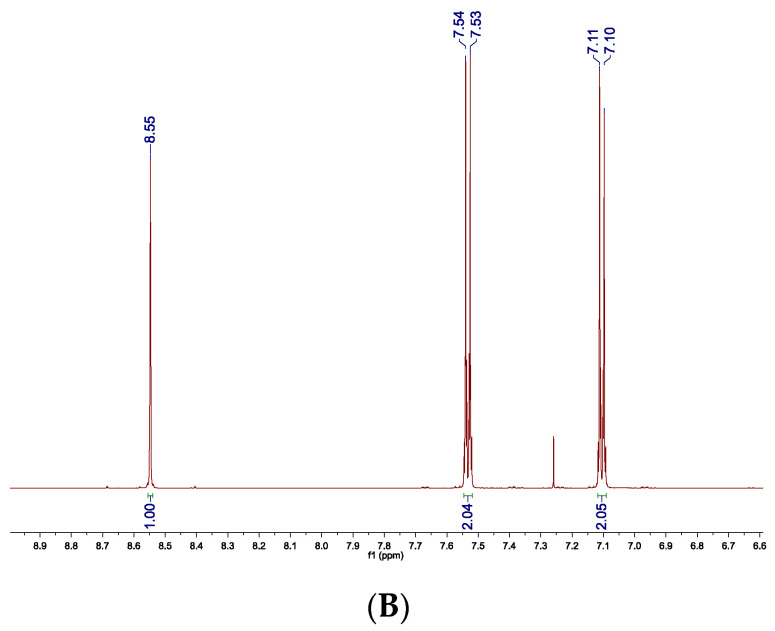
^1^H NMR spectrum of crude **4c** (**A**) and **4c** after filtration through silica gel (**B**). Two small doublets derived from *p*-bromoaniline observed in the spectrum of crude product at δ = 7.22 and 6.55 ppm are not present in the spectrum recorded for the purified amine.

The ^1^H NMR spectra of **4c** recorded before (A) and after purification (B) can be a visualization of the method’s effectiveness (Figure 1).

**Figure 2:**
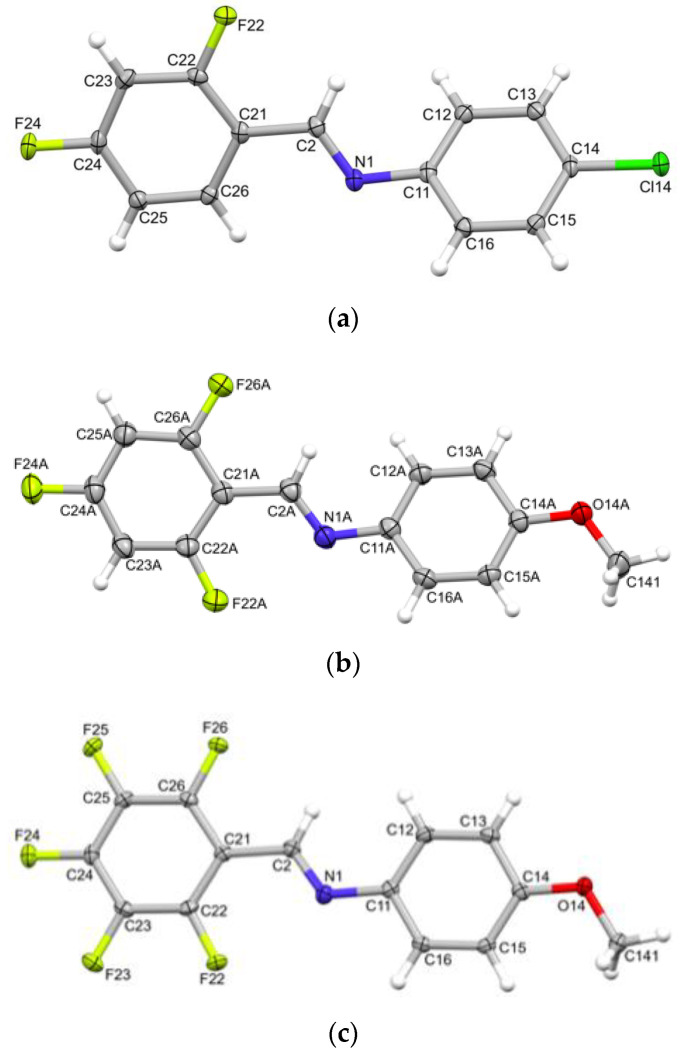
ORTEP views of the molecules (**a**) **2a**, (**b**) one of the symmetry-independent molecules of **3d**(A), and (**c**) **4d**. Ellipsoids are drawn at the 50% probability level; hydrogen atoms are represented by spheres of arbitrary radii.

The yields of imines obtained were influenced by the substituents in the aromatic rings of amines. The nucleophilic addition occurring with the amines having electron-donating groups produced the corresponding imines in better yields, in comparison to the reactions performed with deactivated amines (Table 1). It is worth noticing that, among the obtained imines, 12 (shown in blue, Table 1), to the best of our knowledge, are new compounds, previously not reported in the literature. Six of them (**2g**, **3g** and **1–4 h**) represent derivatives of chiral benzylamines. It should be stressed that all imines containing a stereogenic center were obtained with the high yields (72–98%, Table 1, imines **1–4 g** and **1–4 h**). With regard to the other obtained imines, we were surprised to find in the literature yields reported for only nine compounds: **3a**, **4c**, **1–4d**, **2e**, **4e** and **4i** (Table 1). A few of these imines were synthesized by classical methods based on the use of boiling toluene and the Dean–Stark apparatus (**3a** [32] and **3d** [33]) or chlorobenzene and dowex (**2d** [42]). For the synthesis of **1d**, **2e**, **4d** and **4e**, milder conditions, such as hexane and magnesium sulfate under an inert atmosphere [18], or ethanol [41] or dichloromethane [36,42] at ambient temperatures were used. The mechanochemical method described in this work produced imines with yields comparable to or higher than those reported in the literature (Table 1), and under more environmentally friendly conditions: in a short reaction time of only 15 min and without the use of a solvent (Table 1). The majority of the reports described the remaining imines as generated in situ intermediates involved in various syntheses. Therefore, the imines were not analyzed by spectroscopic methods and their yields were not determined. In this work, for the first time, we presented their NMR spectra and determined the yields of syntheses performed by the mechanochemical method. Moreover, the imines being in the solid state were subjected to crystallization and crystals suitable for X-ray diffraction studies were obtained for **1c** (this structure was reported previously [43]), **2a**, **3d** and **4d**. X-ray analyses unambiguously confirmed the molecular structures of these imines. To the best of our knowledge, the crystal structures of **2a**, **3d** and **4d** have not been reported to date. Single crystals of the compounds were obtained by slow evaporation from hexane (**1c**), chloroform (**2a** and **3d**) and dichloromethane (**4d**) solutions. Perspective views of the molecules **2a**, **3d** and **4d**, as observed in their crystal structures, are presented in Figure 2. An analogous view of the molecule **1c** is shown in ESI (Figure S109).

The scalability of the developed mechanochemical method was examined further. For this purpose, larger-scale reactions were performed for the exemplified substrates. The reaction of 2,4-difluorobenzaldehyde with *p*-anisidine performed at a 10-times-larger scale (2.6 mmol) than the original one (0.26 mmol) proceeded without any problems with a 95% yield (Table 2, entry 5). The reactions performed at 2-, 4-, 6- and 8-times-larger scales also led to the formation of products in very high yields (Table 3). The yields were only slightly lower than those of the original scale reactions (Table 3, entries: 1–6 and 8) or even slightly higher (entry 7). These results show the great potential of the scalability of our method.

## 3. Conclusions

A number of imines derived from differently fluorinated benzaldehydes were synthesized in good-to-excellent yields by the mechanochemical method based on manual grinding with various aniline derivatives as well as chiral benzylamines. Most reactions occurred without any problems, the resulting imines were not contaminated by the remaining substrates and did not require any post-synthetic purification or isolation.

It seems that the amine structures affected the yields stronger than the structures of the aldehydes used. The highest yields were obtained for products formed from amines containing electron-donating groups, as could be rationalized by the stronger nucleophilic character of these substrates resulting from the greater reactivity of the electron pair of the amine nitrogen atom. However, it must be stressed that yields of imines derived from amines having electron-withdrawing substituents were also good.

12 out of 36 synthesized imines represent new compounds that had not been previously reported in the literature. In this study, we provided the NMR data not only for the newly synthesized compounds, but also for all other imines, which in earlier works, were reported only as spectroscopically uncharacterized intermediates. Moreover, we provided crystal structures for **2a**, **3d** and **4d** as representatives of imines having 2, 3 and 5 fluorine atoms, respectively.

Consuming only mechanical energy, not requiring a solvent and producing products in a very short time, this method prevents environment pollution and meets some of the criteria of “green chemistry”. The mechanochemical strategy presented in this work is very convenient and useful, particularly for the preparation of fluorinated imines, and can be easily extended to larger-scale syntheses.

## 4. Methods

### 4.1. General Methods

Reagent-grade chemicals were used. TLC was performed on Merck Kieselgel 60-F254 with EtOAc/hexane as an eluent, and products were detected by UV light (254 nm). NMR spectra were recorded with the instrument operating at 600 MHz (^1^H), 150 MHz (^13^C) and 300 MHz (^19^F). Chemical shifts (δ) are presented in ppm and calibrated from the residual signals of CDCl_3_ (7.26 ppm) and CD_3_OD (3.30 ppm) for ^1^H NMR, and CDCl_3_ (77.16 ppm) and CD_3_OD (49.05 ppm) for ^13^C NMR. High-resolution mass spectra were measured using electrospray ionization (ESI, positive-ion mode) and spectrometer mass QTOF (Impact HD, Bruker Daltonics, Billerica, MA, USA).

### 4.2. General Procedure for the Imines Synthesis

The syntheses were conducted in a fume hood.

Equimolar amounts of aldehyde (0.26 mmol) and amine (0.26 mmol) were placed in a glass round-bottom flask and ground manually with a glass rod without solvent at room temperature for 15 min. The obtained solid or oil was subjected to TLC analysis without any purification. When the analysis showed the presence of unreacted starting material, in addition to the expected product, the mixture was subjected to NMR studies performed with DCM (dichloromethane) as an internal standard, the use of which enabled the calculation of the reaction yield. The yields calculated from the imines’ ^1^H NMR spectra by an internal standard method are marked with “*”. In most cases, TLC analysis showed the presence of pure product. The reaction yield was then calculated from the mass of the crude product, the purity of which was confirmed by the NMR spectra recorded without the internal standard. The spectra (^1^H, ^13^C and ^19^F NMR) obtained for all imines synthesizes are presented in ESI. For the 12 new imines, HRMS spectra were also measured and included into ESI.

*N*-(4-chlorophenyl)-1-(2-fluorophenyl)methanimine (**1a**).

Pale-yellow solid (52 mg, 86%). ^1^H NMR (600 MHz, CDCl_3_): δ = 8.75 (s, 1H, –CH = N), 8.17–8.15 (m, 1H, CHar), 7.49–7.45 (dm, 1H, CHar), 7.37–7.35 (dm, *J* = 8.8 Hz, 2H, 2 CHar), 7.26–7.23 (m, 1H, CHar), 7.18–7.16 (dm, *J* = 8.8 Hz, 2H, 2 CHar), 7.15–7.12 (ddd, *J* = 10.5, 8.4 Hz, 1H, CHar) ppm. ^19^F NMR (300 MHz, CDCl_3_): δ = −121.46 to −121.54 (m, 1F) ppm. ^13^C NMR (150 MHz, CDCl_3_): δ = 162.89 (d, *J* = 253.8 Hz, Car), 153.79 (d, *J* = 5 Hz, N = C–H), 150.36 (s, Car), 133.20 (d, *J* = 8.8 Hz, CHar), 131.86 (s, Car), 129.26 (s, 2 CHar), 127.86 (d, *J* = 2.3 Hz, CHar), 124.53 (d, *J* = 3.5 Hz, CHar), 123.73 (d, *J* = 8.9 Hz, Car), 122.31 (s, 2 CHar), 115.92 (d, *J* = 21.1 Hz, CHar) ppm.

*N*-(4-chlorophenyl)-1-(2,4-difluorophenyl)methanimine (**2a**).

Pale-yellow solid (63 mg, 96%). ^1^H NMR (600 MHz, CDCl_3_): δ = 8.67 (s, 1H, –CH = N), 8.18 (m, 1H, CHar), 7.36 (dm, *J* = 9.0 Hz, 2H, 2 CHar), 7.16 (dm, *J* = 8.9 Hz, 2H, 2 CHar), 6.98 (m, 1H, CHar), 6.88 (m, 1H, CHar) ppm. ^19^F NMR (300 MHz, CDCl_3_): δ = −104.18 to −104.29 (m, 1F), −117.36 to −117.46 (m, 1F) ppm. ^13^C NMR (150 MHz, CDCl_3_): δ = 165.11 (dd, *J* = 255.1, 12.3 Hz, Car), 163.17 (dd, *J* = 256.4, 12.3 Hz, Car), 152.53 (m, N = C–H), 150.12 (s, Car), 131.96 (s, Car), 129.41 (dd, *J* = 10.2, 4.1 Hz, CHar), 129.29 (s, 2 CHar), 122.27 (s, 2 CHar), 120.36 (dd, *J* = 9.2, 3.7 Hz, Car), 112.37 (dd, *J* = 21.8, 3.6 Hz, CHar), 104.16 (t, *J* = 25.3 Hz, CHar) ppm.

*N*-(4-chlorophenyl)-1-(2,4,6-trifluorophenyl)methanimine (**3a**).

Yellow solid (64 mg, 91%). ^1^H NMR (600 MHz, CDCl_3_): δ = 8.55 (s, 1H, –CH = N), 7.36 (dm, *J* = 8.8 Hz, 2H, 2 CHar), 7.15 (dm, *J* = 8.7 Hz, 2H, 2 CHar), 6.78 (tm, *J* = 8.6 Hz, 2H, 2 CHar) ppm. ^19^F NMR (300 MHz, CDCl_3_): δ = −103.95 to −104.97 (m, 1F), −110.73 to −110.82 (m, 2F) ppm. ^13^C NMR (150 MHz, CDCl_3_): δ = 164.93 (m, Car), 163.53 (m, Car), 163.26 (m, Car), 161.81 (m, Car), 150.66 (m, N = C–H, Car), 132.23 (s, Car), 129.32 (s, 2 CHar), 122.13 (s, 2 CHar), 110.63 (m, Car), 101.26 (m, 2 CHar) ppm.

*N*-(4-chlorophenyl)-1-(perfluorophenyl)methanimine (**4a**).

Pale-yellow solid (62 mg, 78%). ^1^H NMR (600 MHz, CDCl_3_): δ = 8.55 (s, 1H, –CH = N), 7.39 (dm, *J* = 8.7 Hz, 2H, 2 CHar), 7.18 (dm, *J* = 8.7 Hz, 2H, 2 CHar) ppm. ^19^F NMR (300 MHz, CDCl_3_): δ = −141.96 to 142.09 (m, 2F), −149.47 to −149.65 (tt, *J* = 20.8, 3.9 Hz, 1F), −161.69 to −161.89 (m, 2F) ppm. ^13^C NMR (150 MHz, CDCl_3_): δ = 149.70 (s, Car), 148.74 (m, N = C–H), 147.10 (m, Car), 145.36 (m, Car), 143.57 (m, Car), 141.81 (m, Car), 138.64 (m, Car), 137.02 (m, Car), 133.08 (s, Car), 129.48 (s, 2 CHar), 122.18 (s, 2 CHar) ppm.

*N*,1-bis(2-fluorophenyl)methanimine (**1b**).

Yellow oil (49 mg, 86%). ^1^H NMR (600 MHz, CDCl_3_): δ = 8.84 (s, 1H, –CH = N), 8.22 (m, 1H, CHar), 7.49–7.45 (m, 1H, CHar), 7.25 (m, 1H, CHar), 7.20–7.11 (m, 5H, 5 CHar) ppm. ^19^F NMR (300 MHz, CDCl_3_): δ = −121.51 to −121.59 (m, 1F), −126.95 to −127.09 (m, 1F) ppm. ^13^C NMR (150 MHz, CDCl_3_): δ = 162.95 (d, *J* = 254.1 Hz, Car), 155.98 (m, N = C–H), 155.28 (d, *J* = 249.3 Hz, Car), 139.92 (d, *J* = 10.4 Hz, Car), 133.36 (d, *J* = 8.8 Hz, CHar), 128.01 (d, *J* = 2.3 Hz, CHar), 127.05 (d, *J* = 7.6 Hz, CHar), 124.51 (m 2 CHar), 123.78 (d, *J* = 8.9 Hz, Car), 121.86 (d, *J* = 1.5 Hz, CHar), 116.27 (d, *J* = 20.1 Hz, CHar), 115.86 (d, *J* = 21.0 Hz, CHar) ppm.

1. -(2,4-difluorophenyl)-*N*-(2-fluorophenyl)methanimine (**2b**).

White oil (36 mg, 58% *). ^1^H NMR (600 MHz, CDCl_3_): δ = 8.76 (s, 1H, –CH = N), 8.24 (m, 1H, CHar), 7.21–7.13 (m, 4H, 4 CHar), 6.99 (m, 1H, CHar), 6.88 (m, 1H, CHar) ppm. ^19^F NMR (300 MHz, CDCl_3_): δ = −104.03 to −104.15 (m, 1F), −117.38 to −117.48 (m, 1F), −126.97 to −127.09 (m, 1F) ppm. ^13^C NMR (150 MHz, CDCl_3_): δ = 166.22 (dd, *J* = 255.2, 12.3 Hz, Car), 163.20 (dd, *J* = 256.7, 12.3 Hz, Car), 156.09 (s, Car), 154.73 (m, N = C–H), 154.43 (s, Car), 139.66 (d, *J* = 10.3 Hz, Car), 129.58 (dd, *J* = 10.2, 3.9 Hz, CHar), 127.15 (d, *J* = 7.7 Hz, CHar), 124.53 (d, *J* = 3.9 Hz, CHar), 121.85 (m, CHar), 116.30 (d, *J* = 20.1 Hz, CHar), 112.36 (dd, *J* = 21.7, 3.4 Hz, CHar), 104.1 (t, *J* = 25.2 Hz, CHar) ppm.

*N*-(2-fluorophenyl)-1-(2,4,6-trifluorophenyl)methanimine (**3b**).

Pale-yellow oil (37 mg, 56% *). ^1^H NMR (600 MHz, CDCl_3_): δ = 8.64 (s, 1H, –CH = N), 7.21–7.17 (m, 1H, CHar), 7.17–7.12 (m, 3H, 3 CHar), 6.79–6.74 (m, 2H, 2 CHar) ppm. ^19^F NMR (300 MHz, CDCl_3_): δ = −102.05 to −102.17 (m, 1F), −108.76 to −108.84 (m, 2F), −127.13 to −127.26 (m, 1F) ppm. ^13^C NMR (150 MHz, CDCl_3_): δ = 164.16 (dt, *J* = 255.5, 15.8 Hz, Car), 162.72 (ddd, *J* = 260.4, 15.0, 8.9 Hz, 2 Car), 155.63 (s, Car), 153.98 (s, Car), 152.94 (m, N = C–H), 140.04 (d, *J* = 10.3 Hz, Car), 127.35 (d, *J* = 7.6 Hz, CHar), 124.50 (d, *J* = 3.8 Hz, CHar), 121.77 (d, *J* = 1 Hz, CHar), 116.28 (d, *J* = 20.1 Hz, CHar), 101.17 (m, 2 CHar) ppm.

*N*-(2-fluorophenyl)-1-(perfluorophenyl)methanimine (**4b**).

Pale-yellow solid (72 mg, 95%). ^1^H NMR (600 MHz, CDCl_3_): δ = 8.67 (s, 1H, –CH = N), 7.26–7.23 (m, 1H, CHar), 7.20–7.16 (m, 3H, 3 CHar) ppm. ^19^F NMR (300 MHz, CDCl_3_): δ = −126.71 to −126.83 (m, 1F), −141.81 to −141.96 (m, 2F), −149.36 to −149.54 (m, 1F), −161.76 to −161.97 (m, 2F) ppm. ^13^C NMR (150 MHz, CDCl_3_): δ = 154.82 (d, *J* = 250,7 Hz, Car), 151.27 (t, *J* = 2.8 Hz, N = C–H), 147.11 (m, Car), 145.39 (m, Car), 143.64 (m, Car), 141.92 (m, Car), 139.10 (d, *J* = 10 Hz, Car), 138.66 (m, Car), 136.97 (m, Car), 128.19 (d, *J* = 7.7 Hz, CHar), 124.65 (d, *J* = 4 Hz, CHar), 122.05 (d, *J* = 0.7 Hz, CHar), 116.53 (d, *J* = 20 Hz, CHar) ppm.

*N*-(4-bromophenyl)-1-(2-fluorophenyl)methanimine (**1c**).

Orange solid (65 mg, 90%). ^1^H NMR (600 MHz, CDCl_3_): δ = 8.75 (s, 1H, –CH = N), 8.17–8.14 (m, 1H, CHar), 7.52–7.50 (dm, *J* = 8.7 Hz, 2H, 2 CHar), 7.49–7.45 (m, 1H, CHar), 7.26–7.23 (m, 1H, CHar), 7.15–7.13 (m, 1H, CHar), 7.12–7.10 (dm, *J* = 8.8 Hz, 2H, 2 CHar) ppm. ^19^F NMR (300 MHz, CDCl_3_): δ = −121.43 to −121.51 (m, 1F) ppm. ^13^C NMR (150 MHz, CDCl_3_): δ = 162.89 (d, *J* = 254 Hz, Car), 153.85 (d, *J* = 5 Hz, N = C–H), 150.85 (s, Car), 133.24 (d, *J* = 8.7 Hz, CHar), 132.22 (s, 2 CHar), 127.87 (d, *J* = 2.3 Hz, CHar), 124.54 (d, *J* = 3.6 Hz, CHar), 123.72 (d, *J* = 9 Hz, Car), 122.69 (s, 2 CHar), 119.72 (s, Car), 115.93 (d, *J* = 21 Hz, CHar) ppm.

*N*-(4-bromophenyl)-1-(2,4-difluorophenyl)methanimine (**2c**).

Pale-yellow solid (66 mg, 85%), mp = 93–95 °C. ^1^H NMR (600 MHz, CDCl_3_): δ = 8.67 (s, 1H, –CH = N), 8.18 (m, 1H, CHar), 7.51 (dt, *J* = 8.5 Hz, 2H, 2 CHar), 7.10 (dt, *J* = 8.5 Hz, 2H, 2 CHar), 6.98 (m, 1H, CHar), 6.88 (m, 1H, CHar) ppm. ^19^F NMR (300 MHz, CDCl_3_): δ = −104.17 (m, 1F), −117.38 (m, 1F) ppm. ^13^C NMR (150 MHz, CDCl_3_): δ = 165.12 (dd, *J* = 255.3, 12.4 Hz, Car), 163.18 (dd, *J* = 256.6, 12.3 Hz, Car), 152.59 (m, N = C–H), 150.61 (s, Car), 132.26 (s, 2 CHar), 129.43 (dd, *J* = 10.2, 4.1 Hz, CHar), 122.64 (s, 2 CHar), 120.35 (dd, *J* = 9.2, 3.7 Hz, Car), 119.81 (s, Car), 112.39 (dd, *J* = 21.8, 3.6 Hz, CHar), 104.18 (t, *J* = 25.3 Hz, CHar) ppm. HRMS (ESI, m/z) calcd. for C_13_H_9_BrF_2_N [M + H]^+^: 295.9886, found: 295.9891.

*N*-(4-bromophenyl)-1-(2,4,6-trifluorophenyl)methanimine (**3c**).

Pale-yellow solid (66 mg, 80%), mp = 74–76 °C. ^1^H NMR (600 MHz, CDCl_3_): δ = 8.54 (s, 1H, –CH = N), 7.80 (dm, *J* = 8.6 Hz, 2H, 2 CHar), 7.08 (dm, *J* = 8.6 Hz, 2H, 2 CHar), 6.77 (tm, *J* = 8.6 Hz, 2H, 2 CHar) ppm. ^19^F NMR (300 MHz, CDCl_3_): δ = −102.08 to −102.21 (m, 1F), −108.86 to −108.95 (t, *J* = 8.9 Hz, 2F) ppm. ^13^C NMR (150 MHz, CDCl_3_): δ = 164.05 (dt, *J* = 255.5, 15.7 Hz, Car), 162.6 (ddd, *J* = 260.2, 15.0, 9.0 Hz, 2 Car), 151.06 (s, Car), 150.60 (d, *J* = 1.3 Hz, N = C–H), 132.21 (s, 2 CHar), 122.43 (s, 2 CHar), 120.04 (s, Car), 110.60 (m, Car), 101.19 (m, 2 CHar) ppm. HRMS (ESI, m/z) calcd. for C_13_H_8_BrF_3_N [M + H]^+^: 313.9792, found: 313.9791.

*N*-(4-bromophenyl)-1-(perfluorophenyl)methanimine (**4c**).

Pale-yellow solid (77 mg, 84% *). ^1^H NMR (600 MHz, CDCl_3_): δ = 8.55 (s, 1H, –CH = N), 7.54 (dt, *J* = 8.5 Hz, 2H, 2 CHar), 7.11 (dt, *J* = 8.6 Hz, 2H, 2 CHar) ppm. ^19^F NMR (300 MHz, CDCl_3_): δ = −141.92 to −142.06 (m, 2F), −149.39 to −149.57 (m, 1F), −161.68 to −161.87 (m, 2F) ppm. ^13^C NMR (150 MHz, CDCl_3_): δ = 150.17 (s, Car), 148.77 (m, N = C–H), 147.07 (m, Car), 145.36 (m, Car), 143.57 (m, Car), 141.83 (m, Car), 138.66 (m, Car), 136.98 (m, Car), 132.43 (s, 2 CHar), 122.48 (s, 2 CHar), 120.96 (s, Car) ppm.

1-(2-. fluorophenyl)-*N*-(4-methoxyphenyl)methanimine (**1d**).

Brown oil (58 mg, 97%). ^1^H NMR (600 MHz, CDCl_3_): δ = 8.80 (s, 1H, –CH = N), 8.17 (m, 1H, CHar), 7.45–7.42 (m, 1H, CHar), 7.27 (m, 2H, 2 CHar), 7.23 (m, 1H, CHar), 7.12 (m, 1H, CHar), 6.94 (m, 2H, 2 CHar), 3.84 (s, 3H, OCH_3_) ppm. ^19^F NMR (300 MHz, CDCl_3_): δ = −122.01 (m, 1F) ppm. ^13^C NMR (150 MHz, CDCl_3_): δ = 162.72 (d, *J* = 253 Hz, Car), 158.57 (s, Car), 151.29 (d, *J* = 4.8 Hz, N = C–H), 144.79 (s, Car), 132.52 (d, *J* = 8.7 Hz, CHar), 127.67 (d, *J* = 2.6 Hz, CHar), 124.44 (d, *J* = 3.4 Hz, CHar), 124.22 (d, *J* = 9 Hz, Car), 122.39 (s, 2 CHar), 115.81 (d, *J* = 21.1 Hz, CHar), 114.40 (s, 2 CHar), 55.50 (s, OCH_3_) ppm.

1-(2,4-difluorophenyl)-*N*-(4-methoxyphenyl)methanimine (**2d**).

Yellow solid (61 mg, 95%). ^1^H NMR (600 MHz, CDCl_3_): δ = 8.72 (s, 1H, –CH = N), 8.21–8.17 (m, 1H, CHar), 7.26–7.24 (dm, *J* = 9 Hz, 2H, 2 CHar), 6.99–6.95 (m, 1H, CHar), 6.95–6.92 (dm, *J* = 9 Hz, 2H, 2 CHar), 6.88–6.85 (m, 1H, CHar), 3.84 (s, 3H, –OCH_3_) ppm. ^19^F NMR (300 MHz, CDCl_3_): δ = −105.57 (m, 1F), −117.98 (dd, *J* = 18.6, 8.7 Hz, 1F) ppm. ^13^C NMR (150 MHz, CDCl_3_): δ = 164.69 (dd, *J* = 254.0, 12.3 Hz, Car), 162.91 (dd, *J* = 255.5, 12.3 Hz, Car), 158.61 (s, Car), 150.03 (m, N = CH), 144.54 (s, Car), 129.14 (dd, *J* = 10, 4.3 Hz, CHar), 122.34 (s, 2 CHar), 120.82 (m, Car), 114.42 (s, 2 CHar), 112.21 (dd, *J* = 21.8, 3.6 Hz, CHar), 104.03 (t, *J* = 25.3 Hz, CHar), 55.49 (s, OCH_3_) ppm.

*N*-(4-methoxyphenyl)-1-(2,4,6-trifluorophenyl)methanimine (**3d**).

Pale-brown solid (69 mg, 99%), mp = 90–91 °C. ^1^H NMR (600 MHz, CDCl_3_): δ = 8.59 (s, 1H, –CH = N), 7.24 (dt, *J* = 8.9 Hz, 2H, 2 CHar), 6.94 (dt, *J* = 9 Hz, 2H, 2 CHar), 6.75 (tm, *J* = 8.6 Hz, 3H, 3 CHar), 3.83 (s, 3H, OCH_3_) ppm. ^19^F NMR (300 MHz, CDCl_3_): δ = −103.56 to −103.68 (m, 1F), −109.46 to −109.52 (t, *J* = 8.6 Hz, 2F) ppm. ^13^C NMR (150 MHz, CDCl_3_): δ = 163.59 (dt, *J* = 254.4, 15.8 Hz, Car), 162.46 (ddd, *J* = 259.0, 14.9, 9.1 Hz, 2 Car), 158.80 (s, N = CH), 148.08 (s, Car), 145.04 (s, Car), 122.23 (s, 2 CHar), 114.39 (s, 2 CHar), 111.09 (m, Car), 101.08 (m, 2 CHar), 55.47 (s, OCH_3_) ppm.

*N*-(4-methoxyphenyl)-1-(perfluorophenyl)methanimine (**4d**).

Pale-yellow solid (77 mg, 98%). ^1^H NMR (600 MHz, CDCl_3_): δ = 8.58 (s, 1H, –CH = N), 7.27 (dt, *J* = 9.0 Hz, 2H, 2 CHar), 6.94 (dt, *J* = 8.9 Hz, 2H, 2 CHar), 3.84 (s, 3H, OCH_3_) ppm. ^19^F NMR (300 MHz, CDCl_3_): δ = −142.61 to −142.73 (m, 2F), −151.01 to −151.18 (m, 1F), −162.20 to −162.40 (m, 2F) ppm. ^13^C NMR (150 MHz, CDCl_3_): δ = 159.42 (s, Car), 146.89 (m, Car), 145.75 (d, *J* = 2.7 Hz, N = C–H), 145.18 (m, Car), 144.05 (s, Car), 143.00 (m, Car), 141.30 (m, Car), 138.60 (m, Car), 136.92 (m, Car), 122.45 (s, 2 CHar), 114.49 (s, 2 CHar), 55.49 (s, OCH_3_) ppm.

1-(2-fluorophenyl)-*N*-(*p*-tolyl)methanimine (**1e**).

Orange oil (54 mg, 97%). ^1^H NMR (600 MHz, CDCl_3_): δ = 8.79 (s, 1H, –CH = N), 8.18 (td, *J* = 7.4, 1.8 Hz, 1H, CHar), 7.45–7.42 (m, 1H, CHar), 7.25–7.19 (m, 3H, 3 CHar), 7.16 (m, 2H, 2 CHar), 7.13–7.10 (m, 1H, CHar), 2.37 (s, 3H, CH_3_) ppm. ^19^F NMR (300 MHz, CDCl_3_): δ = −121.85 (m, 1F) ppm. ^13^C NMR (150 MHz, CDCl_3_): δ = 162.78 (d, *J* = 253 Hz, Car), 152.57 (d, *J* = 4.9 Hz, N = C–H), 149.31 (s, Car), 136.21 (s, Car), 132.70 (d, *J* = 8.6 Hz, CHar), 129.77 (s, 2 CHar), 127.79 (d, *J* = 2.6 Hz, CHar), 124.43 (d, *J* = 3.4 Hz, CHar), 124.10 (d, *J* = 9 Hz, Car), 120.93 (s, 2 CHar), 115.81 (d, *J* = 21.1 Hz, CHar), 21.0 (s, CH_3_) ppm.

1-(2,4-difluorophenyl)-*N*-(*p*-tolyl)methanimine (**2e**).

Brown solid (57 mg, 95%). ^1^H NMR (600 MHz, CDCl_3_): δ = 8.71 (s, 1H, –CH = N), 8.20 (m, 1H, CHar), 7.20 (dm, *J* = 8 Hz, 2H, 2 CHar), 7.15 (dt, *J* = 8.3 Hz, 2H, 2 CHar), 6.97 (m, 1H, CHar), 6.87 (m, 1H, CHar), 2.37 (s, 3H, CH_3_) ppm. ^19^F NMR (300 MHz, CDCl_3_): δ = −105.16 to −105.28 (m, 1F), −117.76 to −117.86 (m, 1F) ppm. ^13^C NMR (150 MHz, CDCl_3_): δ = 164.82 (dd, *J* = 254.3, 12.3 Hz, Car), 163.02 (dd, *J* = 255.9, 12.3 Hz, Car), 151.34 (m, N = C–H), 149.09 (s, Car), 136.31 (s, Car), 129.81 (s, 2 CHar), 129.30 (dd, *J* = 10, 4.2 Hz, CHar), 120.89 (s, 2 CHar), 120.72 (dd, *J* = 9.4, 3.7 Hz, Car), 112.22 (dd, *J* = 21.7, 3.5 Hz, CHar), 104.04 (t, *J* = 25.3 Hz, CHar), 21.00 (s, CH_3_) ppm.

*N*-*p*-tolyl-1-(2,4,6-trifluorophenyl)methanimine (**3e**).

Yellow solid (57 mg, 88%). ^1^H NMR (600 MHz, CDCl_3_): δ = 8.59 (s, 1H, –CH = N), 7.21 (d, *J* = 8.1 Hz, 2H, 2 CHar), 7.14 (dt, *J* = 8.3 Hz, 2H, 2 CHar), 6.77 (m, 2H, 2 CHar), 2.38 (s, 3H, CH_3_) ppm. ^19^F NMR (300 MHz, CDCl_3_): δ = −103.23 to −103.35 (m, 1F), −109.29 to −109.38 (m, 2F) ppm. ^13^C NMR (150 MHz, CDCl_3_): δ = 163.75 (dt, *J* = 254.6, 15.8 Hz, Car), 162.55 (ddd, *J* = 259.4, 15.1, 9.1 Hz, 2 Car), 149.62 (s, Car), 149.42 (s, N = C–H), 136.55 (s, Car), 129.79 (s, 2 CHar), 120.74 (s, 2 CHar), 110.99 (m, Car), 101.11 (m, 2 CHar), 21.01 (s, CH_3_) ppm.

1-(perfluorophenyl)-*N*-(*p*-tolyl)methanimine (**4e**).

Pale-orange solid (69 mg, 92%). ^1^H NMR (600 MHz, CDCl_3_): δ =8.57 (s, 1H, –CH = N), 7.22 (m, 2H, 2 CHar), 7.16 (dm, *J* = 8.3 Hz, 2H, 2 CHar), 2.38 (s, 3H, CH_3_) ppm. ^19^F NMR (300 MHz, CDCl_3_): δ = −142.43 (m, 2F), −150.55 (tt, *J* = 20.7, 3.5 Hz, 1F), −162.18 (m, 2F) ppm. ^13^C NMR (150 MHz, CDCl_3_): δ = 148.69 (s, Car), 147.35 (d, *J* = 2.7 Hz, N = C–H), 146.98 (m, Car), 145.27 (m, Car), 143.20 (m, Car), 141.48 (m, Car), 138.61 (m, Car), 137.51 (s, Car), 136.96 (m, Car), 129.92 (s, 2 CHar), 120.81 (s, 2 CHar), 21.04 (s, CH_3_) ppm.

1-(2-fluorophenyl)-*N*-(o-tolyl)methanimine (**1f**).

Yellow oil (49 mg, 87% *). ^1^H NMR (600 MHz, CDCl_3_): δ = 8.68 (s, 1H, –CH = N), 8.22 (m, 1H, CHar), 7.45–7.42 (m, 1H, CHar), 7.25–7.19 (m, 3H, 3 CHar), 7.15–7.10 (m, 2H, 2 CHar), 6.95–6.94 (m, 1H, CHar), 2.37 (s, 3H, CH_3_) ppm. ^19^F NMR (300 MHz, CDCl_3_): δ = −121.89 to −121.97 (m, 1F) ppm. ^13^C NMR (150 MHz, CDCl_3_): δ = 162.78 (d, *J* = 253.5 Hz, Car), 152.50 (d, *J* = 4.8 Hz, N = C–H), 151.00 (s, Car), 132.72 (d, *J* = 8.7 Hz, CHar), 132.04 (s, Car), 130.27 (s, CHar), 127.91 (d, *J* = 2.5 Hz, CHar), 126.73 (s, CHar), 125.96 (s, CHar), 124.41 (d, *J* = 3.5 Hz, CHar), 124.17 (d, *J* = 9 Hz, Car), 117.62 (s, CHar), 115.84 (d, *J* = 21 Hz, CHar), 17.80 (s, CH_3_) ppm.

1-(2,4-difluorophenyl)-*N*-(*o*-tolyl)methanimine (**2f**).

Yellow oil (45 mg, 74% *). ^1^H NMR (600 MHz, CDCl_3_): δ = 8.60 (s, 1H, –CH = N), 8.23 (m, 1H, CHar), 7.21 (m, 2H, 2 CHar), 7.13 (m, 1H, CHar), 6.96 (m, 1H, CHar), 6.93 (d, *J* = 7.8 Hz, 1H, CHar), 6.85 (m, 1H, CHar), 2.35 (s, 3H, CH_3_) ppm. ^19^F NMR (300 MHz, CDCl_3_): δ = −105.12 to −105.23 (m, 1F), −117.89 to −117.99 (m, 1F) ppm. ^13^C NMR (150 MHz, CDCl_3_): δ = 164.83 (dd, *J* = 254.3, 12.2 Hz, Car), 163.00 (dd, *J* = 256.0, 12.3 Hz, Car), 151.26 (m, N = C–H), 150.75 (s, Car), 132.09 (s, Car), 130.31 (s, CHar), 129.40 (dd, *J* = 10.1, 4.3 Hz, CHar), 126.75 (s, CHar), 126.05 (s, CHar), 120.78 (dd, *J* = 9.1, 3.6 Hz, Car), 117.54 (s, CHar), 112.20 (dd, *J* = 21.7, 3.5 Hz, CHar), 104.03 (t, *J* = 25.3 Hz, CHar), 17.78 (s, CH_3_) ppm. HRMS (ESI, m/z) calcd. for C_14_H_12_F_2_N [M + H]^+^: 232.0937, found: 232.0944.

*N*-*o*-tolyl-1-(2,4,6-trifluorophenyl)methanimine (**3f**).

Pale-yellow solid (64 mg, 98%). ^1^H NMR (600 MHz, CDCl_3_): δ = 8.50 (s, 1H, –CH = N), 7.24–7.20 (m, 2H, 2 CHar), 7.16–7.14 (m, 1H, 1 CHar), 6.92 (d, *J* = 8.9 Hz, 1H, CHar), 6.78–6.74 (m, 2H, 2 CHar), 2.36 (s, 3H, CH_3_) ppm. ^19^F NMR (300 MHz, CDCl_3_): δ = −103.32 to −103.44 (m, 1F), −109.42 to −109.48 (t, *J* = 8.6 Hz, 2F) ppm. ^13^C NMR (150 MHz, CDCl_3_): δ = 163.75 (dt, *J* = 254.3, 15.7 Hz, Car), 162.62 (ddd, *J* = 259.7, 15, 9.1 Hz, 2 Car), 151.29 (s, Car), 149.31 (m, N = C–H), 131.93 (s, Car), 130.31 (s, CHar), 126.74 (s, CHar), 126.29 (s, CHar), 117.27 (s, CHar), 111.03 (m, Car), 101.09 (m, 2 CHar), 17.72 (s, CH_3_) ppm.

1-(perfluorophenyl)-*N*-(*o*-tolyl)methanimine (**4f**).

Pale-yellow solid (71 mg, 96%). ^1^H NMR (600 MHz, CDCl_3_): δ = 8.50 (s, 1H, –CH = N), 7.26–7.22 (m, 2H, 2 CHar), 7.21–7.18 (m, 1H, CHar), 6.95 (dd, *J* = 7.7, 1.4 Hz, 1H, CHar), 2.37 (s, 3H, CH_3_) ppm. ^19^F NMR (300 MHz, CDCl_3_): δ = −142.49 to −142.63 (m, 2F), −150.62 to −150.80 (m, 1F), −162.11 to −162.31 (m, 2F) ppm. ^13^C NMR (150 MHz, CDCl_3_): δ = 150.33 (s, Car), 147.32 (m, N = C–H), 147.04 (m, Car), 145.35 (m, Car), 143.26 (m, Car), 141.53 (m, Car), 138.64 (m, Car), 136.97 (m, Car), 132.59 (s, Car), 130.54 (s, CHar), 127.10 (s, CHar), 126.80 (s, CHar), 116.87 (s, CHar), 17.68 (s, CH_3_) ppm.

1-(2-fluorophenyl)-*N*-(1-phenylethyl)methanimine (**1g**).

White oil (58 mg, 98%). ^1^H NMR (600 MHz, CDCl_3_): δ = 8.69 (s, 1H, –CH = N), 8.08 (m, 1H, CHar), 7.43 (m, 2H, 2 CHar), 7.39–7.35 (m, 1H, CHar), 7.34 (m, 2H, 2 CHar), 7.24 (m, 1H, CHar), 7.16 (m, 1H, CHar), 7.07–7.04 (m, 1H, CHar), 4.58 (q, *J* = 6.6 Hz, 1H, H(C)CH_3_), 1.60 (d, *J* = 6.6 Hz, 3H, H(C)CH_3_) ppm. ^19^F NMR (300 MHz, CDCl_3_): δ = −122.66 (m, 1F) ppm. ^13^C NMR (150 MHz, CDCl_3_): δ = 162.20 (d, *J* = 252 Hz, Car), 152.70 (d, *J* = 4.8 Hz, N = C–H), 144.97 (s, Car), 132.08 (d, *J* = 8.6 Hz, CHar), 128.43 (s, 2 CHar), 127.95 (d, *J* = 2.9 Hz, CHar), 126.88 (s, CHar), 126.59 (s, 2 CHar), 124.24 (d, *J* = 3.5 Hz, CHar), 123.98 (d, *J* = 9.4 Hz, Car), 115.56 (d, *J* = 19.1 Hz, CHar), 70.15 (s, N-C(H)CH_3_), 24.83 (s, N-C(H)CH_3_) ppm.

1-(2,4-difluorophenyl)-*N*-(1-phenylethyl)methanimine (**2g**).

White oil (59 mg, 93%). ^1^H NMR (600 MHz, CDCl_3_): δ = 8.60 (s, –CH = N), 8.09 (td, *J* = 8.6, 6.7 Hz, 1H, CHar), 7.41 (m, 2H, 2 CHar), 7.35–7.32 (m, 2H, 2 CHar), 7.25–7.22 (m, 1H, CHar), 6.91–6.87 (m, 1H, CHar), 6.81–6.78 (m, 1H, CHar), 4.55 (q, *J* = 6.7 Hz, 1H, H(C)CH_3_), 1.58 (d, *J* = 6.7 Hz, 3H, H(C)CH_3_) ppm. ^19^F NMR (300 MHz, CDCl_3_): δ = −106.44 to −106.55 (m, 1F), −118.61 to −118.70 (m, 1F) ppm. ^13^C NMR (150 MHz, CDCl_3_): δ = 164.47 (dd, *J* = 253, 12.3 Hz, Car), 162.40 (dd, *J* = 254.6, 12.1 Hz, Car), 151.54 (d, *J* = 2.8 Hz, N = C–H), 144.87 (s, Car), 129.34 (dd, *J* = 10, 4.6 Hz, CHar), 128.45 (s, 2 CHar), 126.93 (s, CHar), 126.55 (s, 2 CHar), 120.51 (dd, *J* = 9.7, 3.8 Hz, Car), 111.89 (dd, *J* = 21.5, 3.5 Hz, CHar), 103.78 (t, *J* = 25.4 Hz, CHar), 70.11 (s, N-C(H)CH_3_), 24.83 (s, N-C(H)CH_3_) ppm. HRMS (ESI, m/z) calcd. for C_15_H_14_F_2_N [M + H]^+^: 246.1094, found: 246.1103.

*N*-(1-phenylethyl)-1-(2,4,6-trifluorophenyl)methanimine (**3g**).

White oil (64 mg, 93%). ^1^H NMR (600 MHz, CDCl_3_): δ = 8.48 (s, 1H, –CH = N), 7.42 (d, *J* = 7.2 Hz, 2H, 2 CHar), 7.34 (t, *J* = 7.7 Hz, 2H, 2 CHar), 7.24 (m, 1H, CHar), 6.69 (m, 2H, 2 CHar), 4.52 (q, *J* = 6.6 Hz, 1H, H(C)CH_3_), 1.61 (d, *J* = 6.7 Hz, 3H, H(C)CH_3_) ppm. ^19^F NMR (300 MHz, CDCl_3_): δ = −104.68 to 104.80 (m, 1F), −110.02 to −110.14 (m, 2F) ppm. ^13^C NMR (150 MHz, CDCl_3_): δ = 163.28 (dt, *J* = 253.2, 15.7 Hz, Car), 162.22 (ddd, *J* = 257.9, 14.9, 9.3 Hz, 2 Car), 149.19 (s, N = C–H), 144.64 (s, Car), 128.47 (s, 2 CHar), 126.94 (s, CHar), 126.51 (s, 2 CHar), 110.83 (m, Car), 101.00–100.63 (m, 2 CHar), 71.48 (s, N-C(H)CH_3_), 25.00 (s, N-C(H)CH_3_) ppm. HRMS (ESI, m/z) calcd. for C_15_H_13_F_3_N [M + H]^+^: 264.1000, found: 264.0999.

1-(perfluorophenyl)-*N*-(1-phenylethyl)methanimine (**4g**).

Pale-yellow oil (56 mg, 72% *). ^1^H NMR (600 MHz, CDCl_3_): δ = 8.46 (s, 1H, –CH = N), 7.41 (d, *J* = 7.2 Hz, 2H, 2 CHar), 7.35 (t, *J* = 7.7 Hz, 2H, 2 CHar), 7.25 (m, 1H, CHar), 4.56 (q, *J* = 6.6 Hz, 1H, H(C)CH_3_), 1.61 (d, *J* = 6.7 Hz, 3H, H(C)CH_3_) ppm. ^19^F NMR (300 MHz, CDCl_3_): δ = −142.96 to −143.07 (m, 2F), −151.75 to −151.92 (m, 1F), −162.44 to −162.64 (m, 2F) ppm. ^13^C NMR (150 MHz, CDCl_3_): δ = 147.66 (m, N = C–H), 146.70 (m, Car), 145.00 (m, Car), 144.11 (s, Car), 142.84 (m, Car), 141.09 (m, Car), 138.48 (m, Car), 136.82 (m, Car), 128.57 (s, 2 CHar), 127.16 (s, CHar), 126.46 (s, 2 CHar), 71.65 (s, N-C(H)CH_3_), 25.02 (s, N-C(H)CH_3_) ppm.

1-(2-fluorophenyl)-*N*-(1-(4-methoxyphenyl)ethyl)methanimine (**1h**).

White oil (58 mg, 86%). ^1^H NMR (600 MHz, CDCl_3_): δ = 8.67 (s, 1H, –CH = N), 8.07–8.04 (m, 1H, CHar), 7.39–7.33 (m, 3H, 3 CHar), 7.17–7.14 (m, 1H, CHar), 7.07–7.04 (m, 1H, CHar), 6.89 (dm, *J* = 8.8 Hz, 2H, 2 CHar), 4.55 (q, *J* = 6.6 Hz, H(C)CH_3_), 3.80 (s, 3H, OCH_3_), 1.57 (d, *J* = 6.6 Hz, 3H, H(C)CH_3_) ppm. ^19^F NMR (300 MHz, CDCl_3_): δ = −122.71 (m, 1F) ppm. ^13^C NMR (150 MHz, CDCl_3_): δ = 162.20 (d, *J* = 252 Hz, Car), 158.53 (s, Car), 152.45 (d, *J* = 4.7 Hz, N = C–H), 137.09 (s, Car), 132.04 (d, *J* = 8.5 Hz, CHar), 127.96 (s, CHar), 127.64 (s, 2 CHar), 124.23 (d, *J* = 3.5 Hz, CHar), 124.01 (d, *J* = 9.2 Hz, Car), 115.60 (d, *J* = 15.2 Hz, CHar), 113.83 (s, 2 CHar), 69.52 (s, N-C(H)CH_3_), 55.28 (s, OCH_3_), 24.68 (s, N-C(H)CH_3_) ppm. HRMS (ESI, m/z) calcd. for C_16_H_17_FNO [M + H]^+^: 258.1294, found: 258.1283.

1-(2,4-difluorophenyl)-*N*-(1-(4-methoxyphenyl)ethyl)methanimine (**2h**).

White oil (65 mg, 91%). ^1^H NMR (600 MHz, CDCl_3_): δ = 8.58 (s, 1H, –CH = N), 8.09–8.05 (m, 1H, CHar), 7.32 (dd, 2H, *J* = 8.8, 0.5 Hz, 2 CHar), 6.90 (m, 1H, CHar), 6.88 (dd, *J* = 8.8 Hz, 2H, 2 CHar), 6.81–6.78 (m, 1H, CHar), 4.52 (q, 1H, *J* = 6.6 Hz, H(C)CH_3_), 3.09 (s, 3H, OCH_3_), 1.56 (d, 3H, *J* = 6.6 Hz, H(C)CH_3_) ppm. ^19^F NMR (300 MHz, CDCl_3_): δ = −106.63 (m, 1F), −118.70 (m, 1F) ppm. ^13^C NMR (150 MHz, CDCl_3_): δ = 164.39 (dd, *J* = 252.9, 12.6 Hz, Car), 162.38 (dd, *J* = 254.5, 12.1 Hz, Car), 158.5 (s, Car), 151.2 (m, N = C–H), 136.9 (s, Car), 129.3 (dd, *J* = 10, 4,5 Hz, CHar), 127.6 (s, 2 CHar), 120.54 (dd, *J* = 9.7, 3.7 Hz, Car), 113.84 (s, 2 CHar), 111.98–111.81 (dd, *J* = 21.5, 3.5 Hz, CHar), 103.77 (t, *J* = 25.4 Hz, CHar), 69.47 (s, N-C(H)CH_3_), 55.23 (s, OCH_3_), 24.66 (s, N-C(H)CH_3_) ppm. HRMS (ESI, m/z) calcd. for C_16_H_16_F_2_NO [M + H]^+^: 276.1199, found: 276.1201.

*N*-(1-(4-methoxyphenyl)ethyl)-1-(2,4,6-trifluorophenyl)methanimine (**3h**).

White oil (73 mg, 96%). ^1^H NMR (600 MHz, CDCl_3_): δ = 8.46 (s, 1H, N = C–H), 7.33 (dm, *J* = 8.8 Hz, 2H, 2 CHar), 6.88 (dm, *J* = 8.8 Hz, 2H, 2 CHar), 6.71–6.66 (m, 2 CHar), 4.49 (q, *J* = 6.6, 1H, H(C)CH_3_), 3.79 (s, 3H, OCH_3_), 1.59 (d, *J* = 6.7 Hz, 3H, H(C)CH_3_) ppm. ^19^F NMR (300 MHz, CDCl_3_): δ = −104.81 to −104.93 (m, 1F), −110.09 to −110.16 (m, 2F) ppm. ^13^C NMR (150 MHz, CDCl_3_): δ = 163.44 (dt, *J* = 253.1, 15.7 Hz, Car), 162.18 (ddd, *J* = 257.9, 15.0, 9.4 Hz, 2 Car), 158.56 (s, Car), 148.89 (s, N = C–H), 136.73 (s, Car), 127.56 (s, 2 CHar), 113.84 (s, 2 CHar), 110.86 (m, Car), 100.97–100.60 (m, 2 CHar), 70.82 (s, N-C(H)CH_3_), 55.21 (s, OCH_3_), 24.85 (s, N-C(H)CH_3_) ppm. HRMS (ESI, m/z) calcd. for C_16_H_15_F_3_NO [M + H]^+^: 294.1105, found: 294.1105

*N*-(1-(4-methoxyphenyl)ethyl)-1-(perfluorophenyl)methanimine (**4h**).

Pale-yellow solid (66 mg, 77%). ^1^H NMR (600 MHz, CDCl_3_): δ = 8.44 (s, 1H, –CH = N), 7.32 (dm, *J* = 8.8 Hz, 2H, 2 CHar), 6.90 (dm, *J* = 8.8 Hz, 2H, 2 CHar), 4.53 (q, *J* = 6.6 Hz, 1H, H(C)CH_3_), 3.80 (s, 3H, OCH_3_), 1.59 (d, *J* = 6.6 Hz, 3H, H(C)CH_3_) ppm. ^19^F NMR (300 MHz, CDCl_3_): δ = −143.03 (m, 2F), −151.91 (m, 1F), −162.54 (m, 2F) ppm. ^13^C NMR (150 MHz, CDCl_3_): δ = 158.75 (s, Car), 147.42 (d, *J* = 2.3 Hz, N = C-H), 146.70 (m, Car), 144.97 (m, Car), 142.82 (m, Car), 141.11 (m, Car), 138.50 (m, Car), 136.87 (m, Car), 136.18 (s, Car), 127.59 (s, 2 CHar), 113.99 (s, 2 CHar), 71.03 (s, N-C(H)CH_3_), 55.30 (s, OCH_3_), 24.89 (s, N-C(H)CH_3_) ppm. HRMS (ESI, m/z) calcd. for C_16_H_13_F_5_NO [M + H]^+^: 330.0917, found: 330.0908.

4-((2-fluorobenzylidene)amino)phenol (**1i**).

Pale-yellow solid (44 mg, 79% *). ^1^H NMR (600 MHz, CD_3_OD): δ = 8.78 (s, 1H, –CH = N), 8.07 (m, 1H, CHar), 7.50–7.46 (m, 1H, CHar), 7.26 (m, 1H, CHar), 7.18 (m, 3H, 2 CHar), 6.83 (dt, *J* = 8.8 Hz, 2H, 2 CHar), 6.61 (m, 1H, CHar) ppm. ^19^F NMR (300 MHz, CD_3_OD): δ = −121.27 to −121.35 (m, 1F) ppm. ^13^C NMR (150 MHz, CD_3_OD): δ = 164.09 (d, *J* = 252.2 Hz, Car), 158.10 (s, Car), 152.26 (d, *J* = 4.9 Hz, N = C–H), 144.62 (s, Car), 134.06 (d, *J* = 8.7 Hz, CHar), 128.66 (d, *J* = 2.5 Hz, CHar), 125.73 (d, *J* = 3.5 Hz, CHar), 125.31 (d, *J* = 9.2 Hz, Car), 123.56 (s, 2 CHar), 116.95 (d, *J* = 21.2, CHar), 116.92 (s, 2 CHar) ppm.

4-((2,4-difluorobenzylidene)amino)phenol (**2i**).

Pale-yellow solid (55 mg, 90% *), mp = 141–143 °C. ^1^H NMR (600 MHz, CD_3_OD): δ = 8.70 (s, 1H, –CH = N), 8.11 (m, 1H, CHar), 7.17 (dt, *J* = 8.9 Hz, 2H, 2 CHar), 7.04 (m, 1H, CHar), 6.81 (dt, *J* = 9 Hz, 2H, 2 CHar) ppm. ^19^F NMR (300 MHz, CD_3_OD): δ = −105.45 to −105.57 (m, 1F), −116.97 to −117.08 (m, 1F) ppm. ^13^C NMR (150 MHz, CD_3_OD): δ = 166.21 (dd, *J* = 252.8, 12.5 Hz, Car), 164.35 (dd, *J* = 251.6, 12.4 Hz, Car), 158.11 (s, Car), 150.96 (dd, *J* = 4.2, 1.5 Hz, N = C–H), 144.48 (s, Car), 130.35 (dd, *J* = 10.2, 4.2 Hz, CHar), 123.56 (s, 2 CHar), 122.15 (dd, *J* = 9.3, 3.8 Hz, Car), 116.91 (s, 2 CHar), 113.28 (dd, *J* = 22.0, 3.5 Hz, CHar), 105.10 (t, *J* = 25.8 Hz, CHar) ppm. HRMS (ESI, m/z) calcd. for C_13_H_10_F_2_NO [M + H]^+^: 234.0730, found: 234.0727.

4-((2,4,6-trifluorobenzylidene)amino)phenol (**3i**).

Pale-yellow solid (29 mg, 45% *), mp = 124–126 °C. ^1^H NMR (600 MHz, CD_3_OD): δ = 8.60 (s, 1H, –CH = N), 7.17 (dm, *J* = 8.8 Hz, 2 CHar), 6.98 (t, *J* = 8.9 Hz, 2 CHar), 6.82 (dm, *J* = 8.9 Hz, 2 CHar) ppm. ^19^F NMR (300 MHz, CD_3_OD): δ = −103.43 to −103.55 (m, 1F), −109.35 to −109.45 (m, 2F) ppm. ^13^C NMR (150 MHz, CD_3_OD): δ = 164.54 (m, 3 Car), 158.39 (s, Car), 148.99 (m, N = C–H), 144.84 (s, Car), 123.45 (s, 2 CHar), 118.58 (s, Car), 116.94 (s, 2 CHar), 102.15 (m, 2 CHar) ppm. HRMS (ESI, m/z) calcd. for C_13_H_9_F_3_NO [M + H]^+^: 252.0636, found: 252.0629.

4-(((perfluorophenyl)methylene)amino)phenol (**4i**).

Yellow solid (48 mg, 68% *). ^1^H NMR (600 MHz, CD_3_OD): δ = 8.61 (s, 1H, –CH = N), 7.22 (dt, *J* = 8.8 Hz, 2H, 2 CHar), 6.83 (dt, *J* = 8.9 Hz, 2H, 2 CHar) ppm. ^19^F NMR (300 MHz, CD_3_OD): δ = −142.96 to −143.06 (m, 2F), −152.90 to −153.06 (m, 1F), −163.57 to −163.75 (m, 2F) ppm. ^13^C NMR (150 MHz, CD_3_OD): δ = 158.96 (s, Car), 148.21 (m, Car), 146.57 (m, N = C–H), 146.46 (m, Car), 144.13 (s, Car), 142.58 (m, Car), 139.99 (m, Car), 138.35 (m, Car), 123.75 (s, 2 CHar), 116.93 (s, 2 CHar), 113.15 (m, Car) ppm.

### 4.3. X-ray Crystallography 

Diffraction data were collected by the ω-scan technique for **1c**, **2a** and **4d** at 100(1) K on a Rigaku XCalibur four-circle diffractometer with am Eos CCD detector equipped with a graphite-monochromatized MoK_α_ radiation source (λ = 0.71073 Å), and for **3d** at 130(1) K on a Rigaku SuperNova four-circle diffractometer with an Atlas CCD detector equipped with a Nova microfocus CuK_α_ radiation source (λ = 1.54178 Å). The data were corrected for Lorentz polarization as well as for absorption effects [44]. The structures were solved with SHELXT [45] and refined with the full-matrix least-squares procedure on F^2^ by SHELXL-2013 [46]. All non-hydrogen atoms were refined anisotropically; hydrogen atoms were placed in idealized positions and refined as ‘riding model’ with isotropic displacement parameters set at 1.2 (1.5 for methyl groups) times U_eq_ of appropriate carrier atoms. The relevant crystallographic data for **2a**, **4d** and **3d** together with the details of structure refinement are listed in Table 4. The appropriate data obtained for **1c** are presented in ESI, Table S1.

## Figures and Tables

**Table 1:** Structures and yields (compared to those reported in the literature, if available) of the imines obtained by the mechanochemical method. The imines shown in blue are new compounds, previously not described in the literature.

	Aldehyde	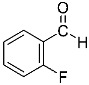 1	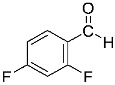 2	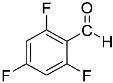 3	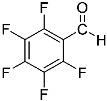 4
Amine					
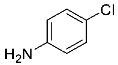 **a**		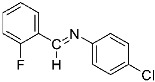 **1a** 86%	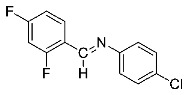 **2a** 96%	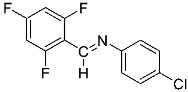 **3a** 91% [99 [32], 100 [33]]	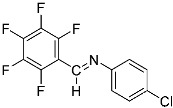 **4a** 78% [92 [34]]
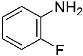 **b**		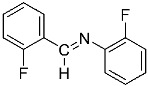 **1b** 86%	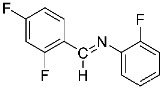 **2b** 58% *	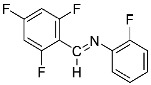 **3b** 56% *	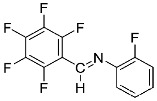 **4b** 95%
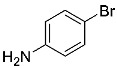 **c**		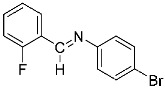 **1c** 90%	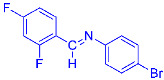 **2c** 85%	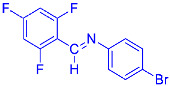 **3c** 80%	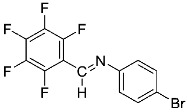 **4c** 84% * [83 [34]]
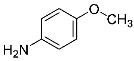 **d**		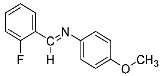 **1d** 97% [78 [18]]	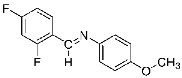 **2d** 95% [66 [35]]	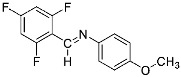 **3d** 99% [99 [32]]	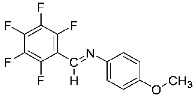 **4d** 98% [80 [36], 92 [37], 97 [38,39], 98 [40]]
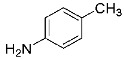 **e**		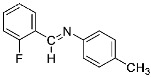 **1e** 97%	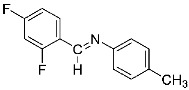 **2e** 95% [81 [41]]	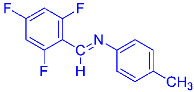 **3e** 88%	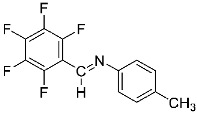 **4e** 92% [93 [42], 95 [34]]
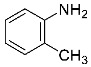 **f**		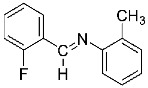 **1f** 87% *	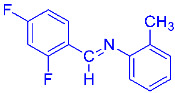 **2f** 74% *	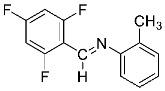 **3f** 98%	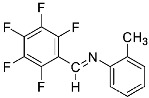 **4f** 96%
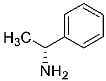 **g**		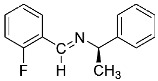 **1g** 98%	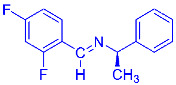 **2g** 93%	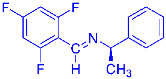 **3g** 93%	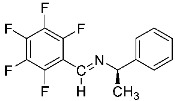 **4g** 72% *
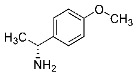 **h**		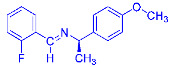 **1h** 86%	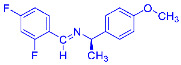 **2h** 91%	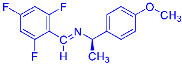 **3h** 96%	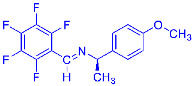 **4h** 77%
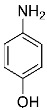 **i**		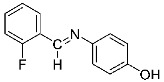 **1i** 79% *	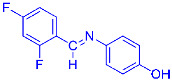 **2i** 90% *	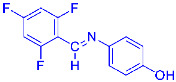 **3i** 45% *	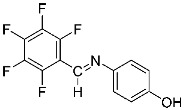 **4i** 68% * [54 [34]]

“*” Calculated from the ^1^H NMR spectrum by an internal standard method with DCM used as an internal standard. [ ] Yields reported in the literature.

**Table 2:** Comparison of the NMR and isolated yields obtained for the imines subjected to purification through filtration on silica gel.

Entry	Imine	NMR Yield [%]	Isolated Yield [%]
**1**	**2b**	58	57
**2**	**3b**	56	56
**3**	**4c**	84	76
**4**	**1f**	87	87
**5**	**2f**	74	68
**6**	**4g**	72	69
**7**	**1i**	79	75
**8**	**2i**	90	77
**9**	**3i**	45	35
**10**	**4i**	68	58

**Table 3:** Results of research on the scalability of the mechanochemical method.

Entry	Scale (Multiplicity of 0.26 mmol Used in the Original Scale)	Aldehyde (mmol) 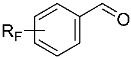	Amine (mmol)	Imine	Yield [%]
**1**	2 times larger	R_F_: 2,4-F_2_ (0.52)	*p*-Anisidine (0.52)	**2d**	96
**2**	4 times larger	R_F_: 2,4-F_2_ (1.04)	*p*-Anisidine (1.04)	**2d**	97
**3**	6 times larger	R_F_: 2,4-F_2_ (1.56)	*p*-Anisidine (1.56)	**2d**	97
**4**	8 times larger	R_F_: 2,4-F_2_ (2.08)	*p*-Anisidine (2.08)	**2d**	97
**5**	10 times larger	R_F_: 2,4-F_2_ (2.6)	*p*-Anisidine (2.6)	**2d**	95
**6**	2 times larger	R_F_: 2,3,4,5,6-F_5_ (0.52)	*o*-Toluidine (0.52)	**4f**	95
**7**	4 times larger	R_F_: 2,4,6-F_3_ (1.04)	(*R*)-(+)-4-methoxy-α-methylbenzylamine (1.04)	**3h**	98
**8**	8 times larger	R_F_: 2-F (2.08)	*p*-Toluidine (2.08)	**1e**	96

**Table 4:** Crystal data, data collection and structure refinement.

Compound	2a	3d	4d
Formula	C_13_H_8_ClF_2_N	C_14_H_10_F_3_NO	C_14_H_8_F_5_NO
Formula weight	251.65 /c	265.23 /c	301.21 /c
Crystal system	Monoclinic	Monoclinic	Triclinic
Space group	P2_1_/c	P2_1_/c	P-1
a (Å)	13.0347 (3)	13.50700 (18)	6.4217 (3)
b (Å)	11.4697 (3)	7.10152 (9)	7.3068 (4)
c (Å)	7.4832 (2)	24.8299 (3)	12.8876 (6)
α (°)	90	90	85.930 (4)
β (°)	106.440 (3)	92.4523 (11)	80.022 (4)
γ (°)	90	90	80.491 (4)
V (Å^3^)	1073.03 (5)	2379.51 (5)	586.82 (5)
Z	4	8	2
D_x_ (g cm^−3^)	1.558	1.481	1.705
F (000)	512	1088	304
μ (mm^−1^)	0.356	1.088	0.162
Reflections:			
Collected	9167	10141	9572
Unique (R_int_)	2355 (0.022)	4847 (0.022)	2626 (0.019)
With I > 2σ(I)	2088	4410	2163
R(F) [I > 2σ(I)]	0.0299	0.0364	0.0350
wR(F^2^) [I > 2σ(I)]	0.0693	0.0996	0.0966
R(F) [all data]	0.0365	0.0398	0.0446
wR(F^2^) [all data]	0.0727	0.1024	0.1035
Goodness of fit	1.073	1.049	1.047
Max/min Δρ (e·Å^−3^)	0.31/−0.20	0.25/−0.18	10.27/−0.28
CCDC number	2149774	2149775	2150609

CCDC 2149773-2149775 and 2150609 contain the supplementary crystallographic data for this paper. These data can be obtained free of charge from The Cambridge Crystallographic Data Centre via www.ccdc.cam.ac.uk/data_request/cif (accessed on 7 February 2022).

## Data Availability

The crystallographic data for compounds **1c**, **2a**, **3d** and **4d** can be obtained free of charge from The Cambridge Crystallographic Data Centre via www.ccdc.cam.ac.uk/data_request/cif (accessed on 1 June 2022).

## Supplementary Materials

The following supporting information can be downloaded at https://www.mdpi.com/article/10.3390/molecules27144557/s1: Figures S1–S108: NMR spectra (^1^H, ^13^C and ^19^F) of the synthesized imines; X-ray crystallography data of the compounds **1c**, **2a**, **3d** and **4d** (Table S1, Figure S109 and checkCIF reports); HRMS spectra of the new imines: **1h**, **2c**, **2f**, **2g**, **2h**, **2i**, **3c**, **3e**, **3g**, **3h**, **3i**, **4h**.
