# Supplementary material for: Mechanochemical Synthesis of Fluorinated Imines
Source: Molecules. 2022 Jul 17;27(14):4557. doi: 10.3390/molecules27144557 (PMC9323187; doi:10.3390/molecules27144557)
Supplement: Supplementary file 1 [file molecules-27-04557-s001.zip › molecules-1817996-supplementary.pdf]

Electronic Supplementary Information

Mechanochemical synthesis of fluorinated imines

Karolina Ciesielska, Marcin Hoffmann, Maciej Kubicki, Donata Pluskota-Karwatka *

Adam Mickiewicz University in Poznań, Faculty of Chemistry, Uniwersytetu Poznańskiego 8, 61-614 Poznań, Poland

1. NMR spectra of the imines **2**
2. X-ray crystallography data **57**
3. HRMS spectra of new compounds **75**

NMR spectra of the imines

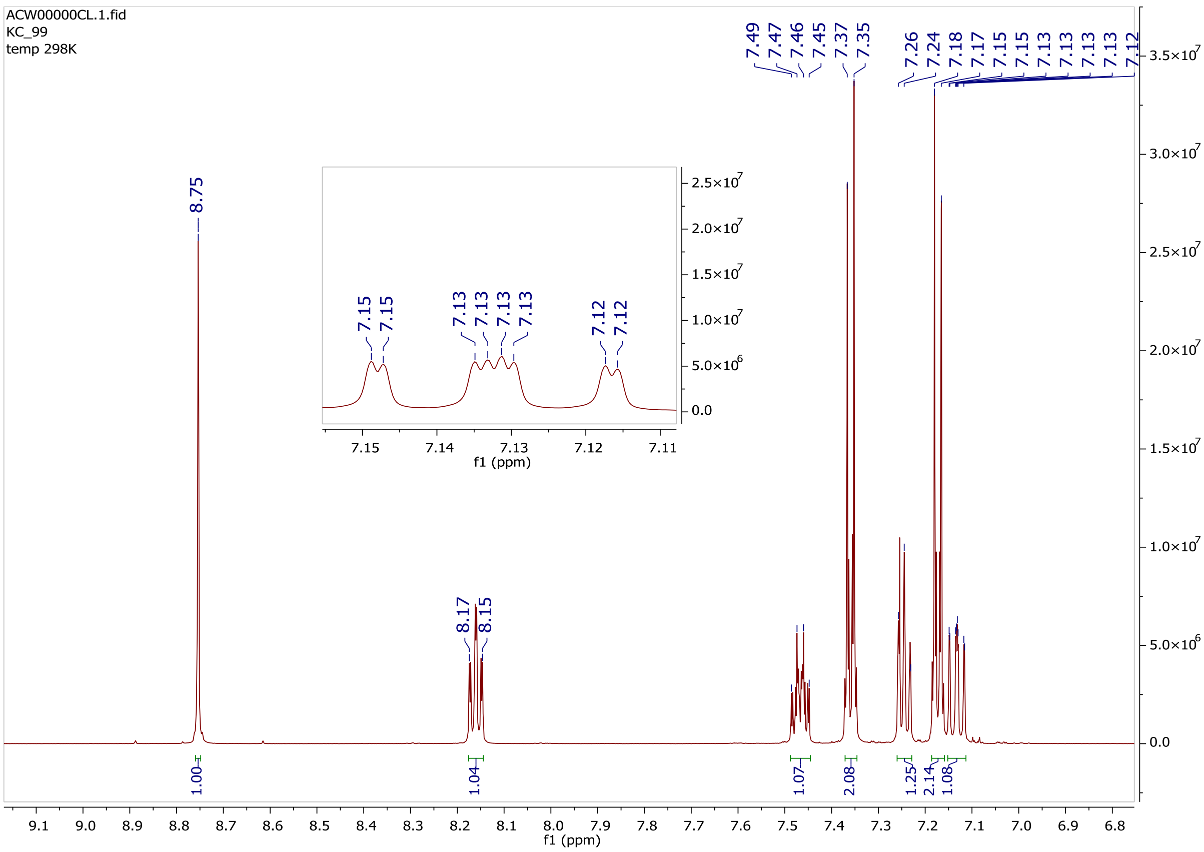

Figure S1. ^{1}H NMR spectrum of 1a ($CDCl_3$)

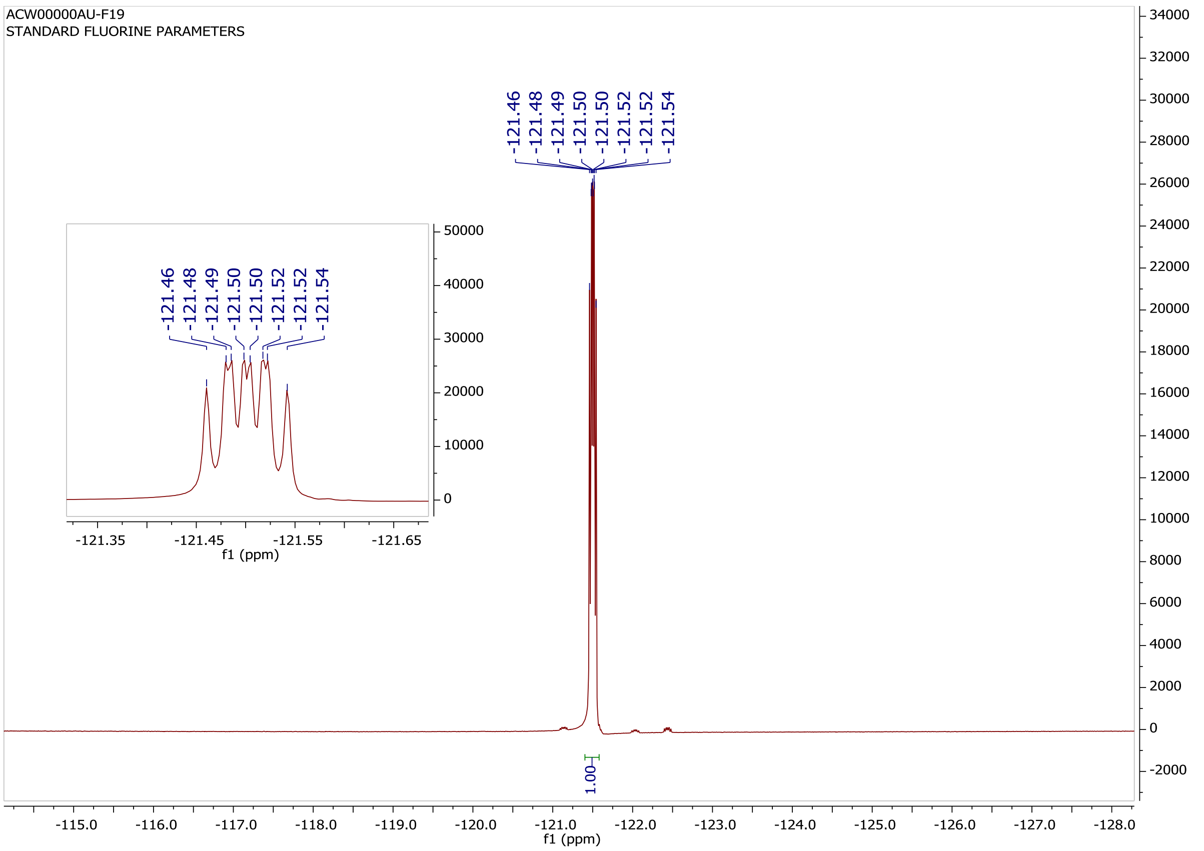

Figure S2. ^{19}F NMR spectrum of 1a ($CDCl_3$)

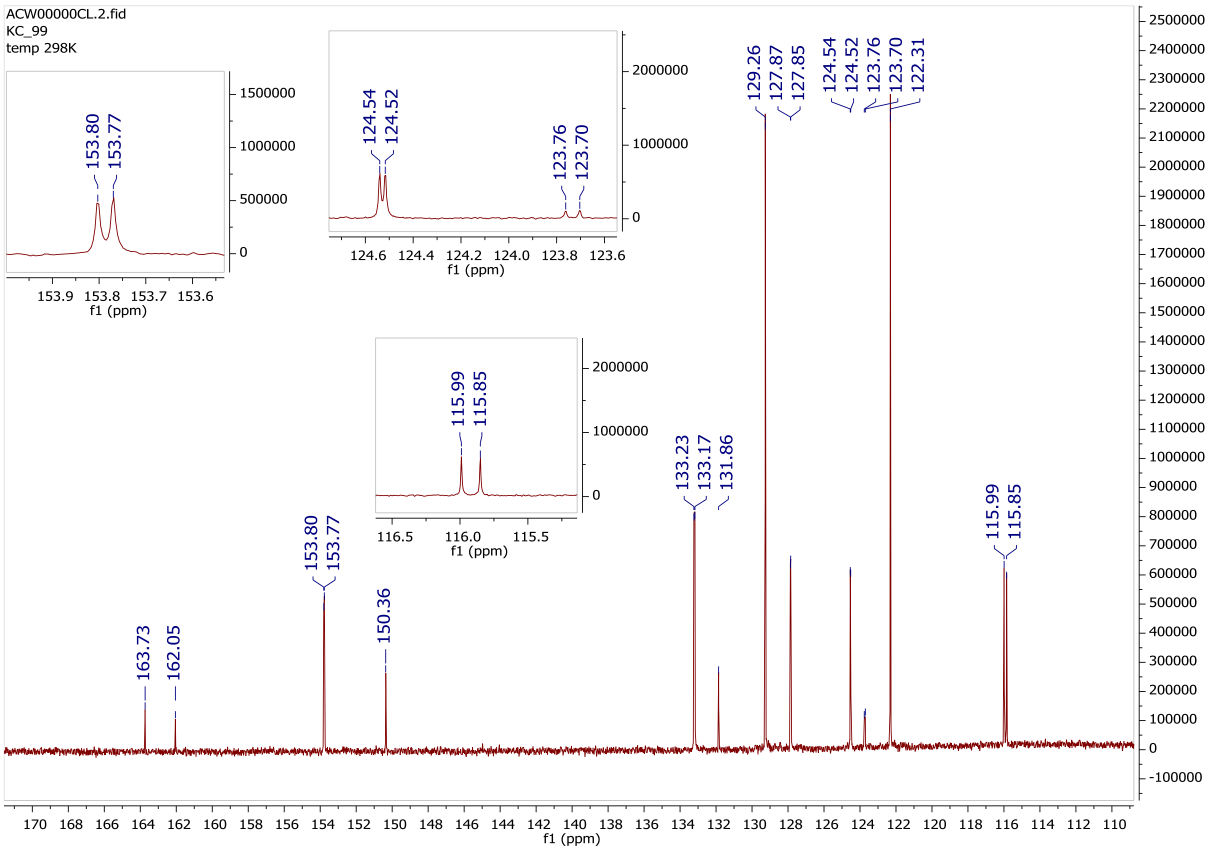

Figure S3. ^{13}**C NMR spectrum of 1a (CDCl$_3$)**

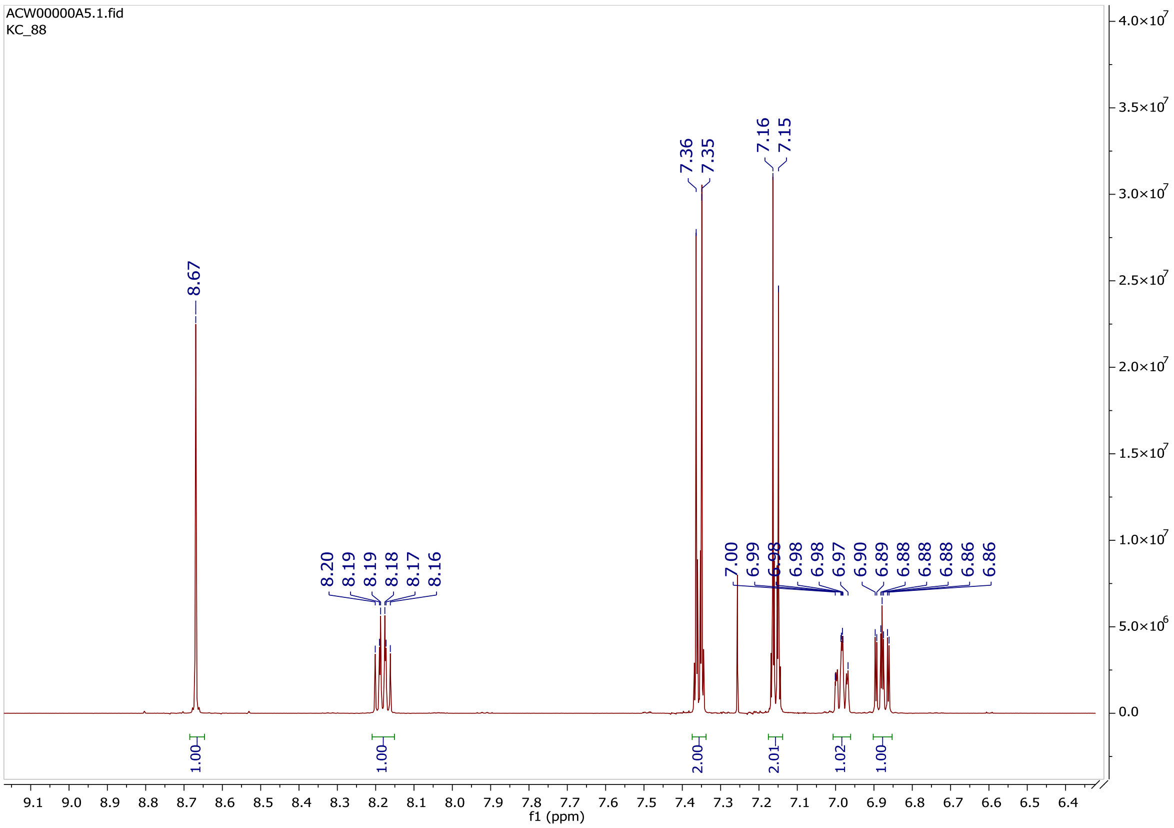

Figure S4. ^{1}**H NMR spectrum of 2a (CDCl$_3$)**

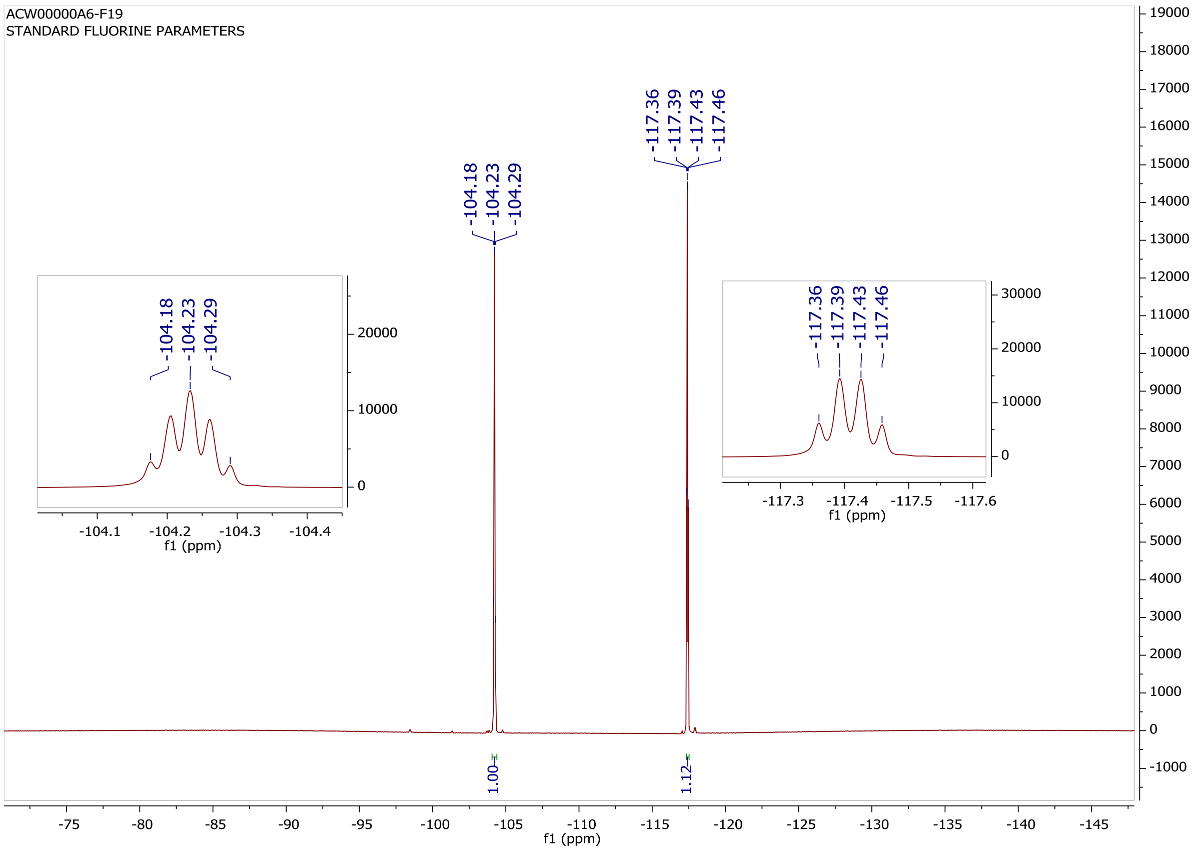

Figure S5. [19]**F NMR spectrum of 2a (CDCl₃)**

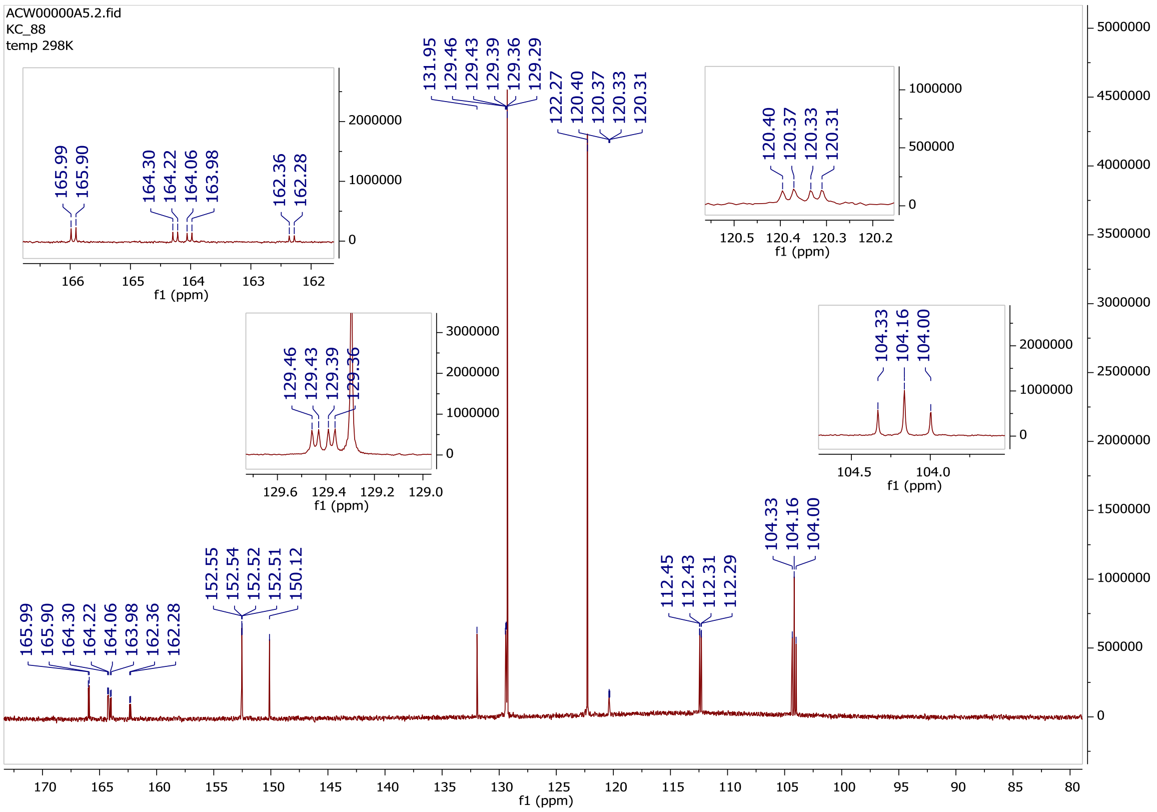

Figure S6. [13]**C NMR spectrum of 2a (CDCl₃)**

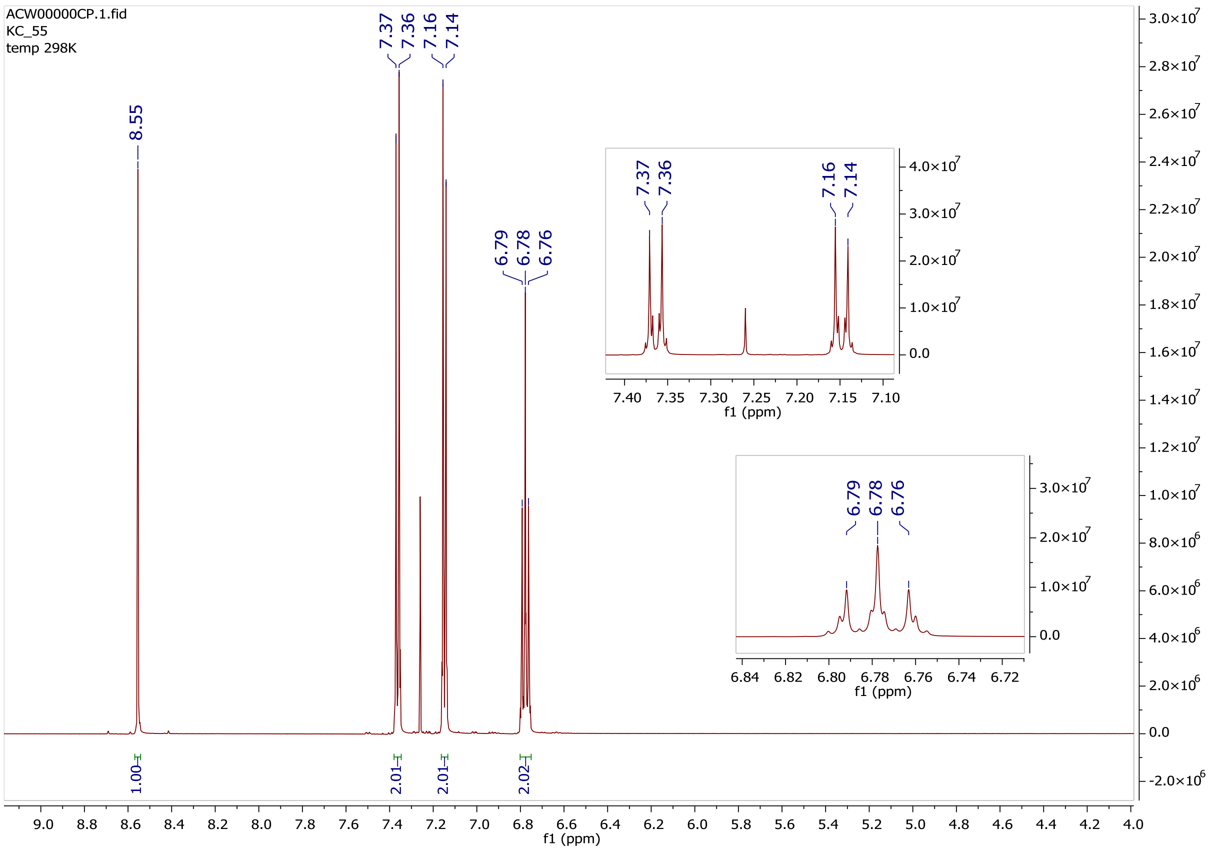

Figure S7. [1]**H NMR spectrum of 3a ($CDCl_3$)**

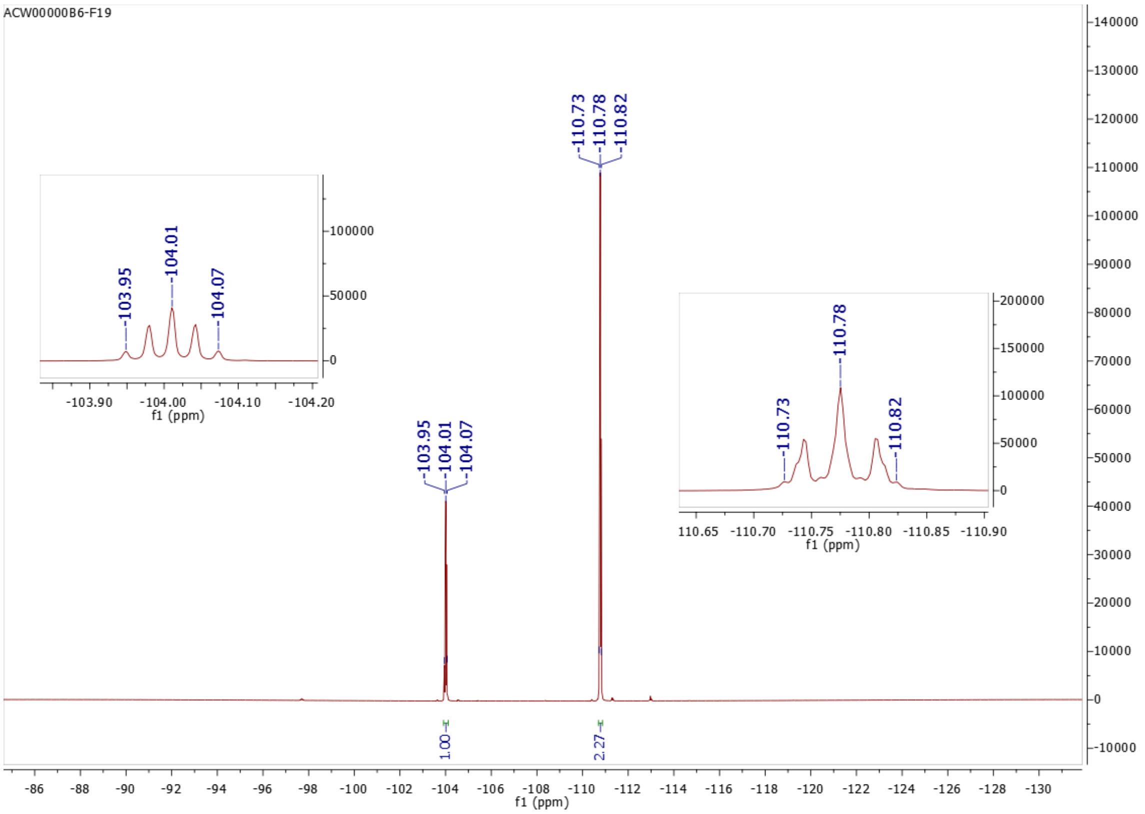

Figure S8. [19]**F NMR spectrum of 3a ($CDCl_3$)**

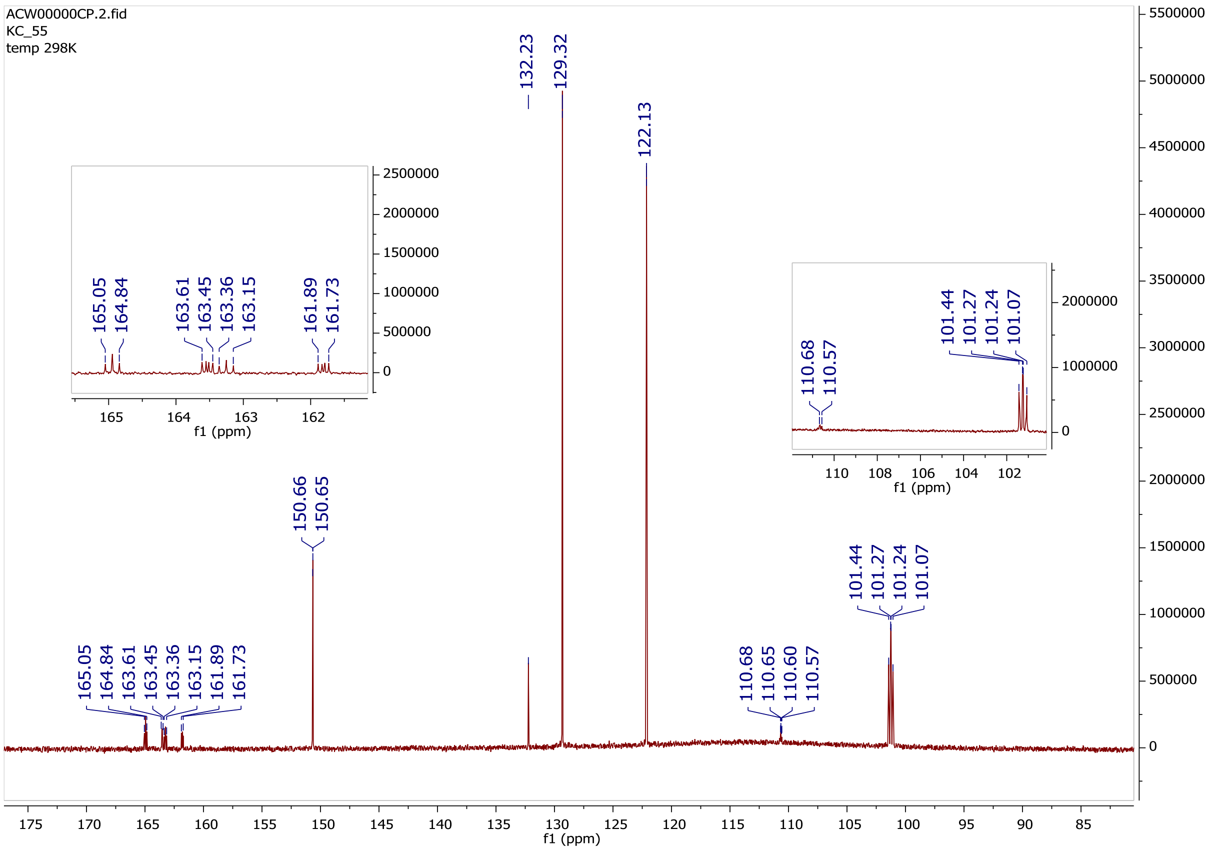

Figure S9. ^{13}**C NMR spectrum of 3a (CDCl$_3$)**

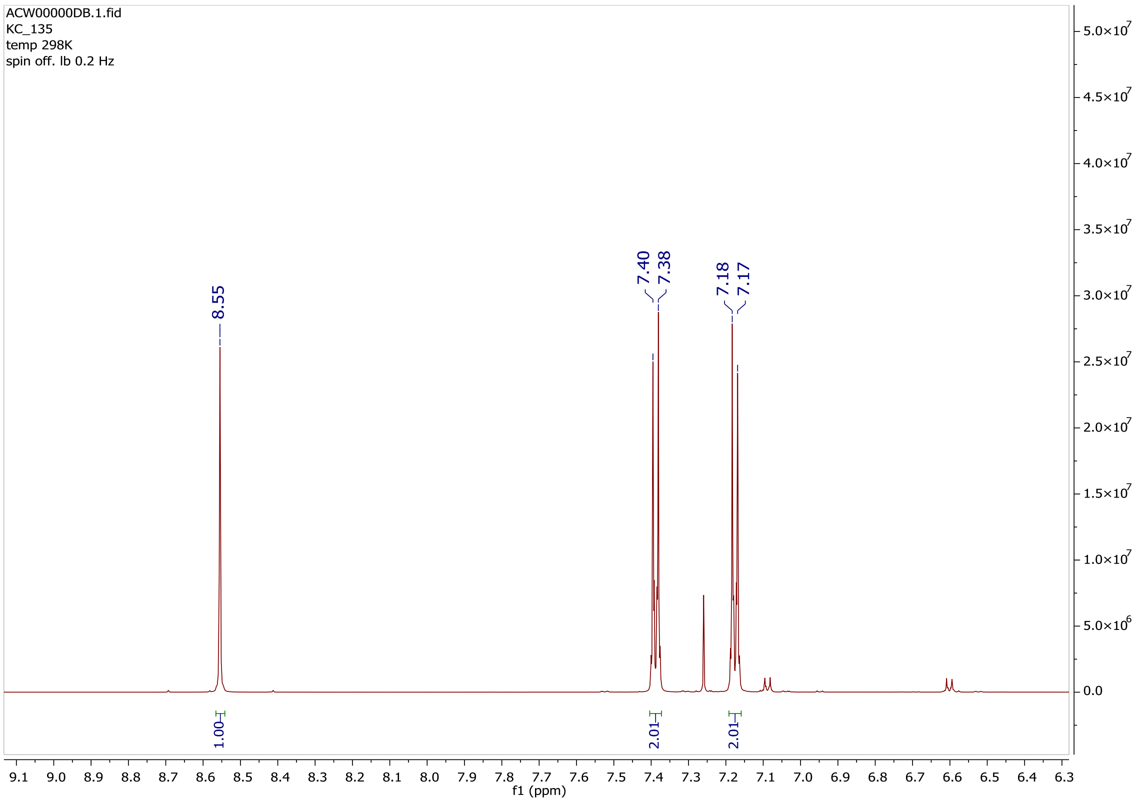

Figure S10. ^{1}**H NMR spectrum of 4a (CDCl$_3$)**

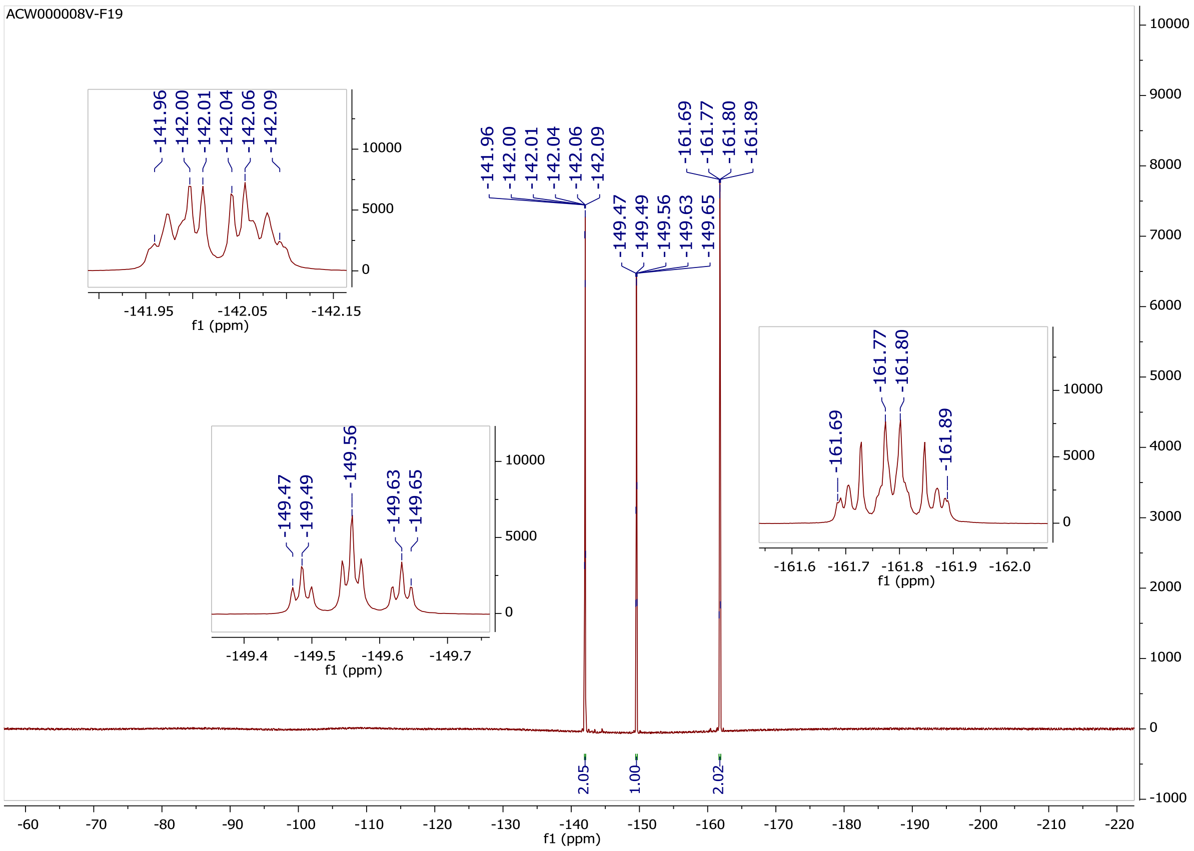

Figure S11. [19]**F NMR spectrum of 4a ($CDCl_3$)**

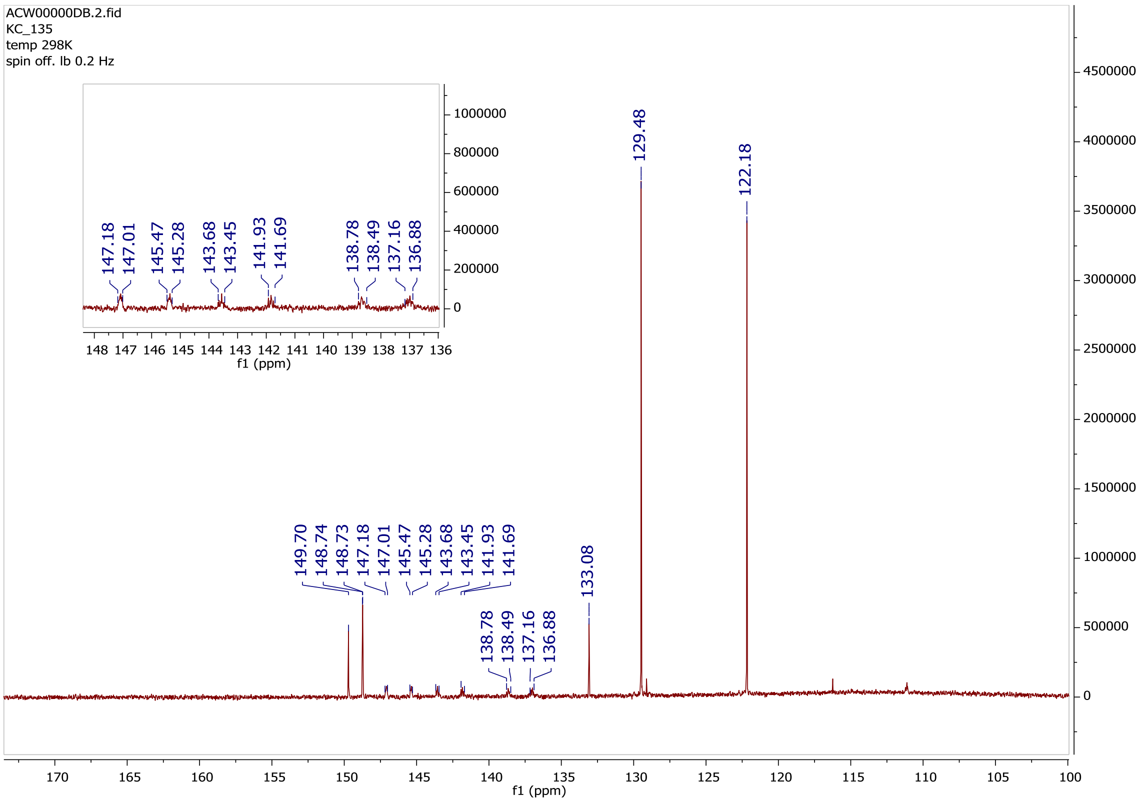

Figure S12. [13]**C NMR spectrum of 4a ($CDCl_3$)**

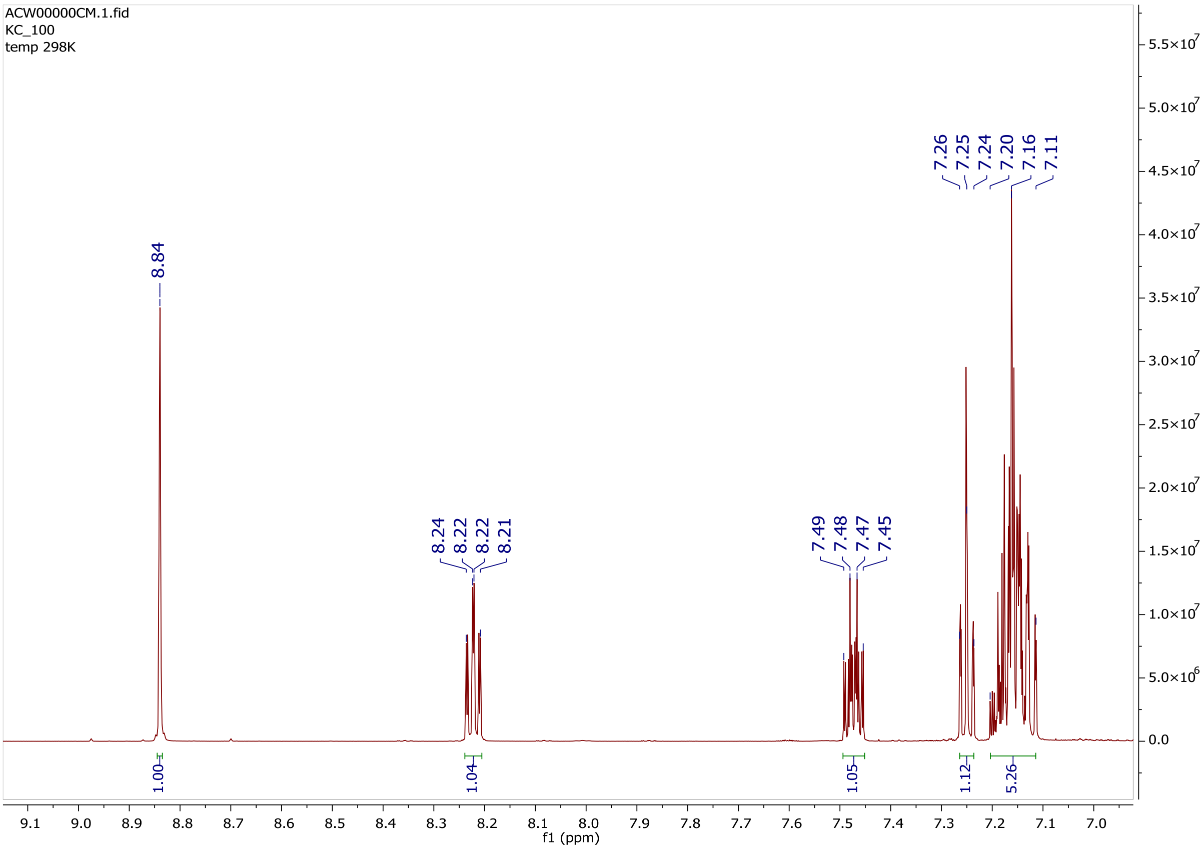

Figure S13. ^{1}H **NMR spectrum of 1b (** $CDCl_3$ **)**

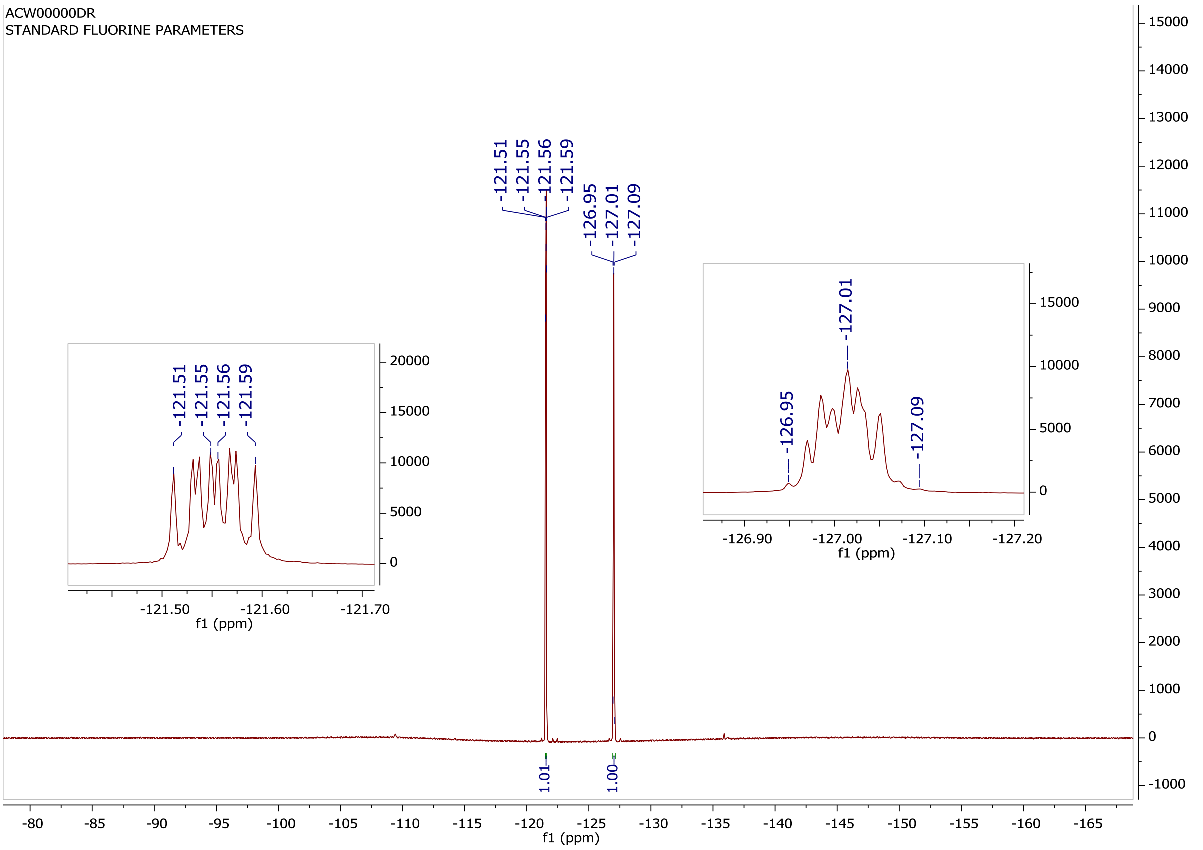

Figure S14. ^{19}F **NMR spectrum of 1b (** $CDCl_3$ **)**

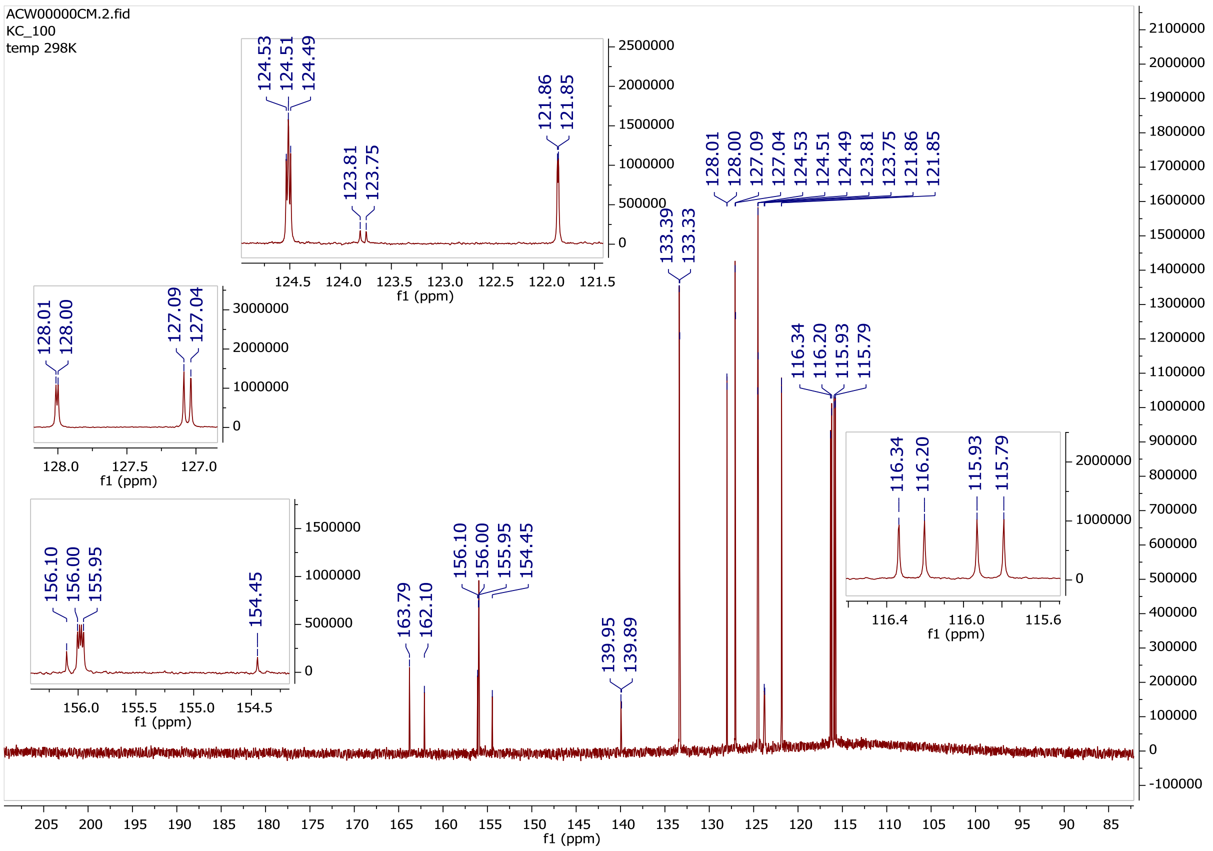

Figure S15. ^{13}**C NMR spectrum of 1b (CDCl$_3$)**

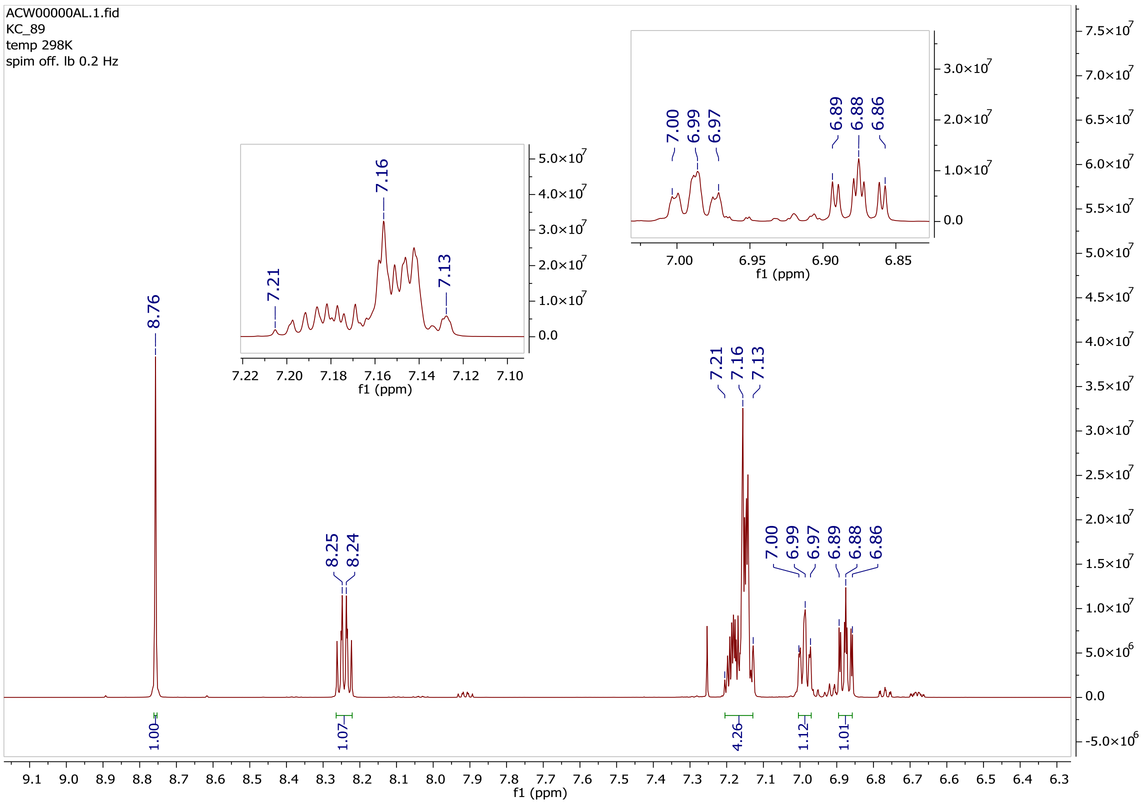

Figure S16. ^{1}**H NMR spectrum of 2b (CDCl$_3$)**

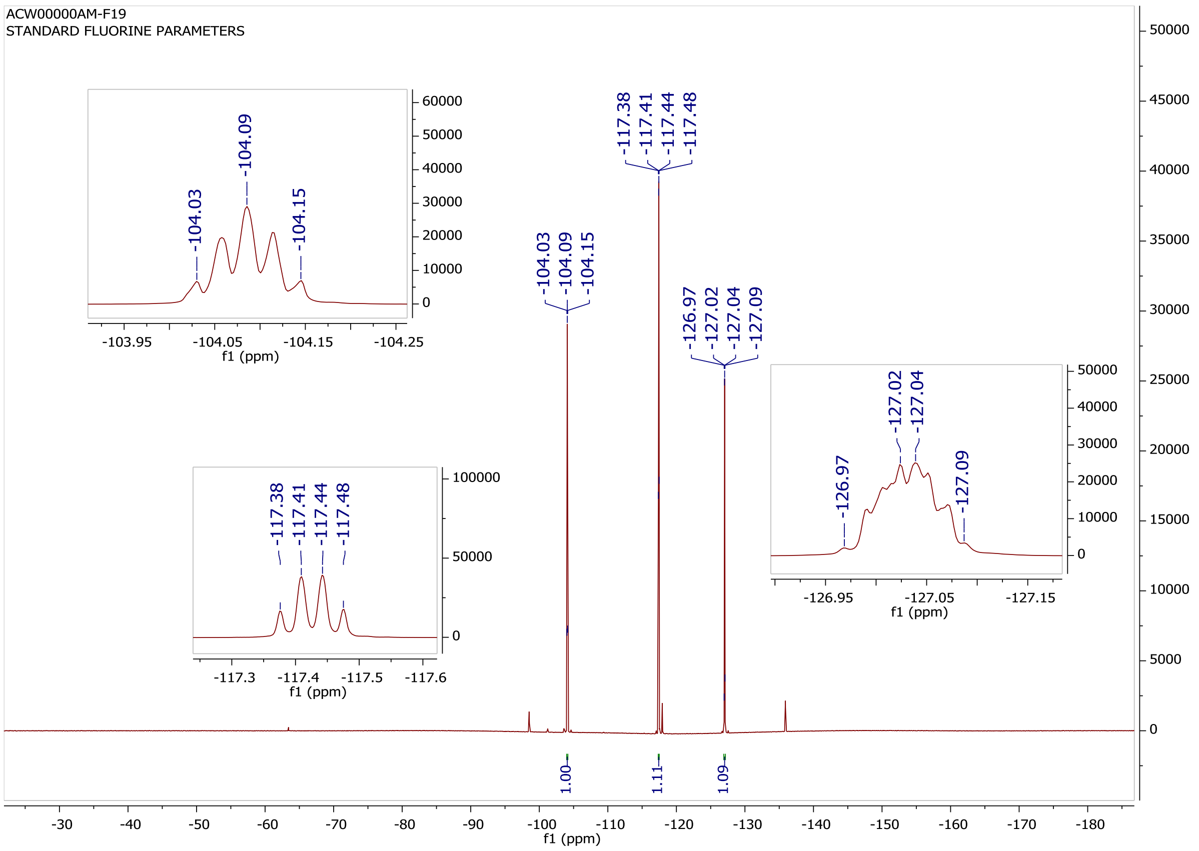

Figure S17. ^{19}**F NMR spectrum of 2b (CDCl**$_3$**)**

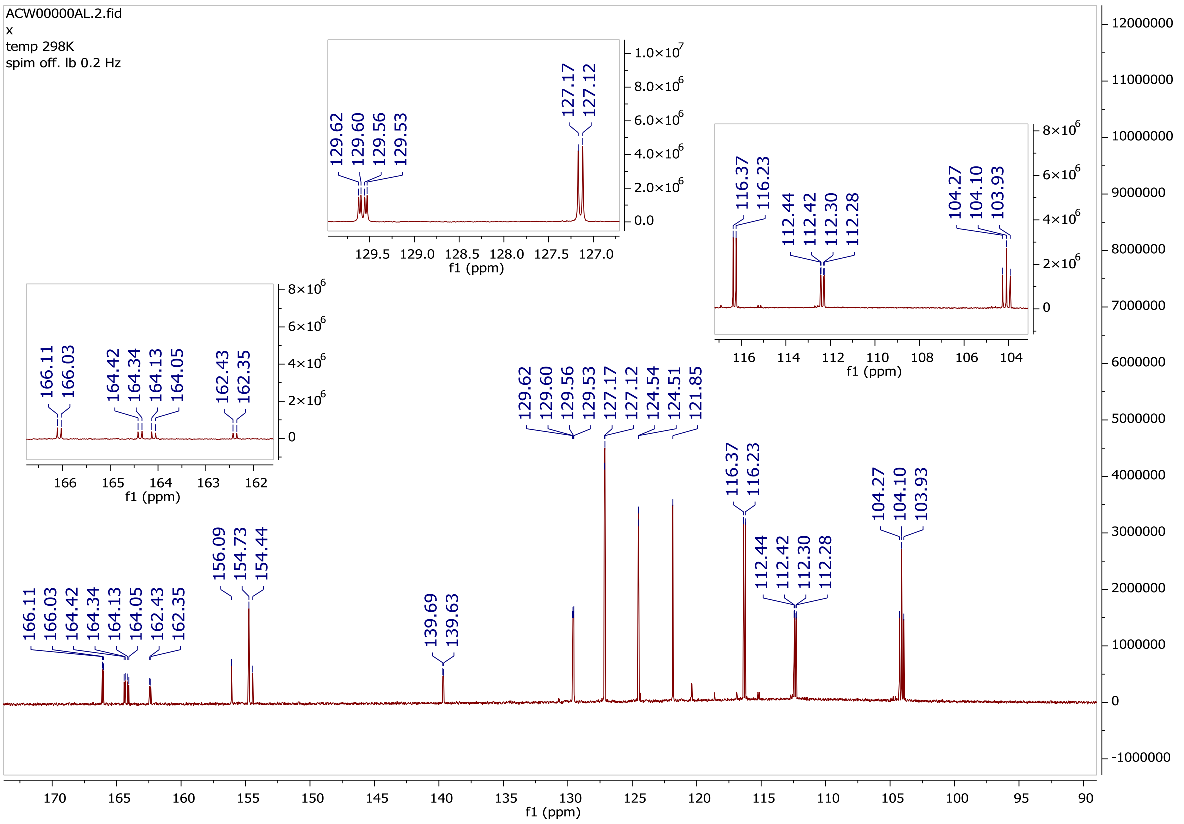

Figure S18. ^{13}**C NMR spectrum of 2b (CDCl**$_3$**)**

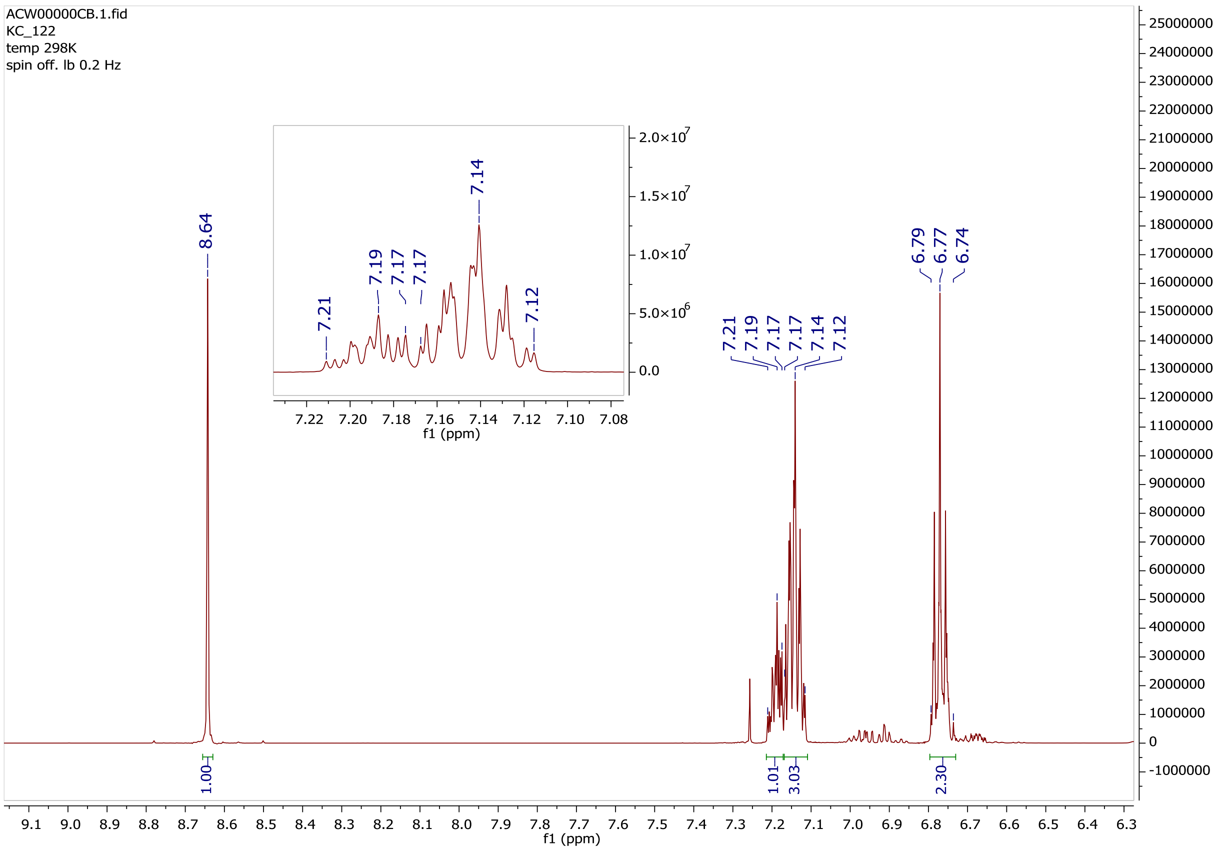

Figure S19. ^{1}**H NMR spectrum of 3b (CDCl$_3$)**

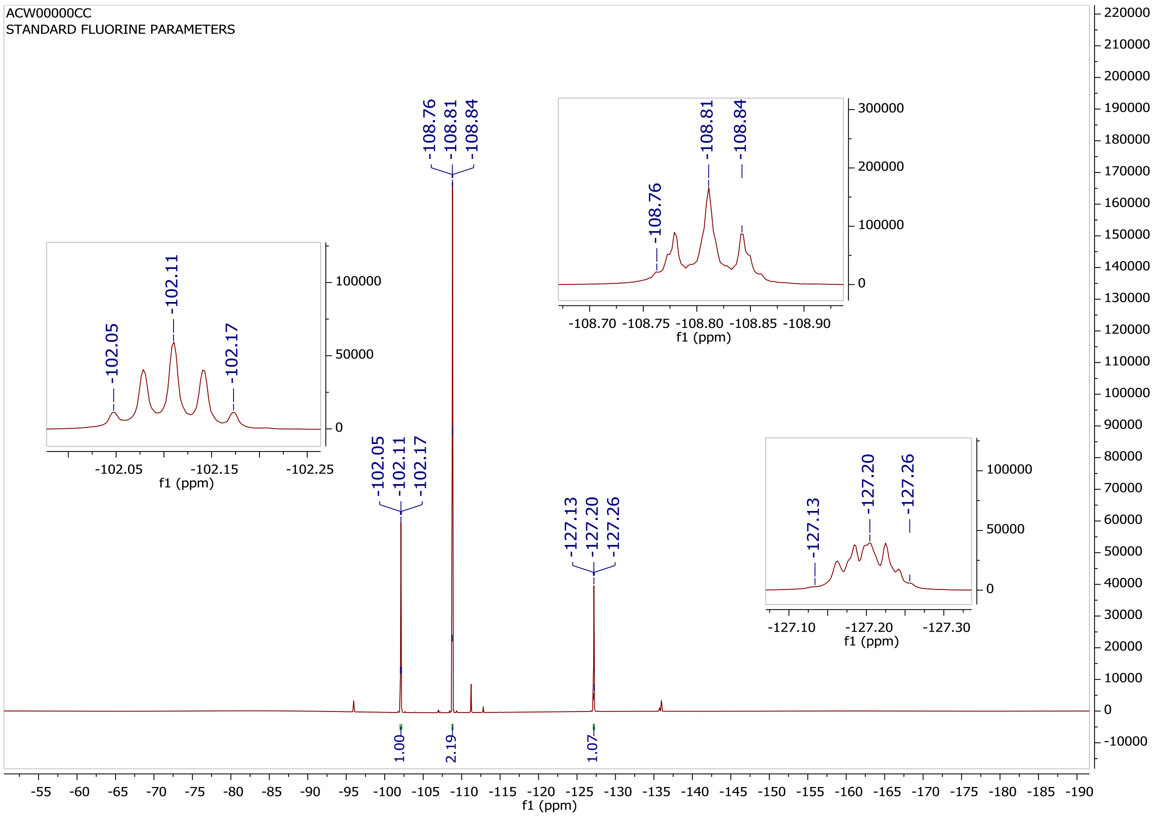

Figure S20. ^{19}**F NMR spectrum of 3b (CDCl$_3$)**

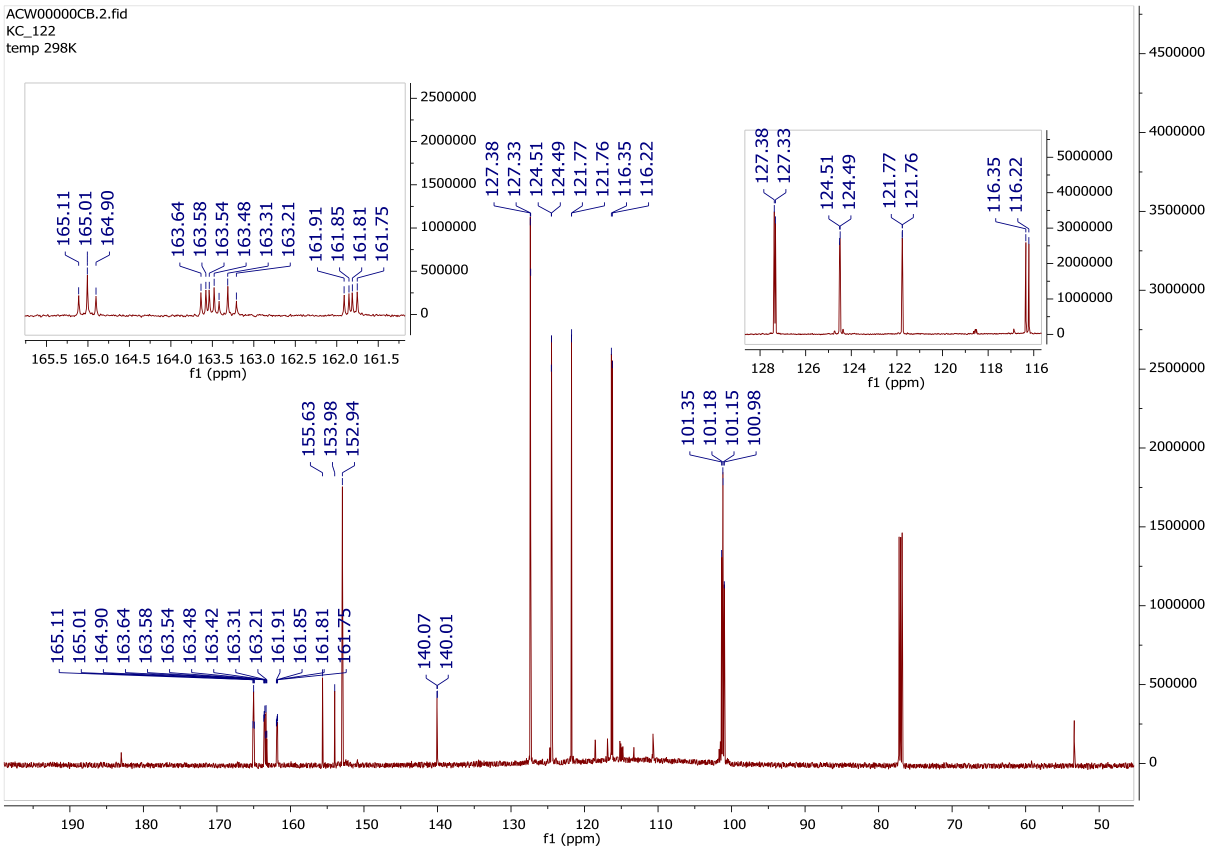

Figure S21. ^{13}**C NMR spectrum of 3b (CDCl$_3$)**

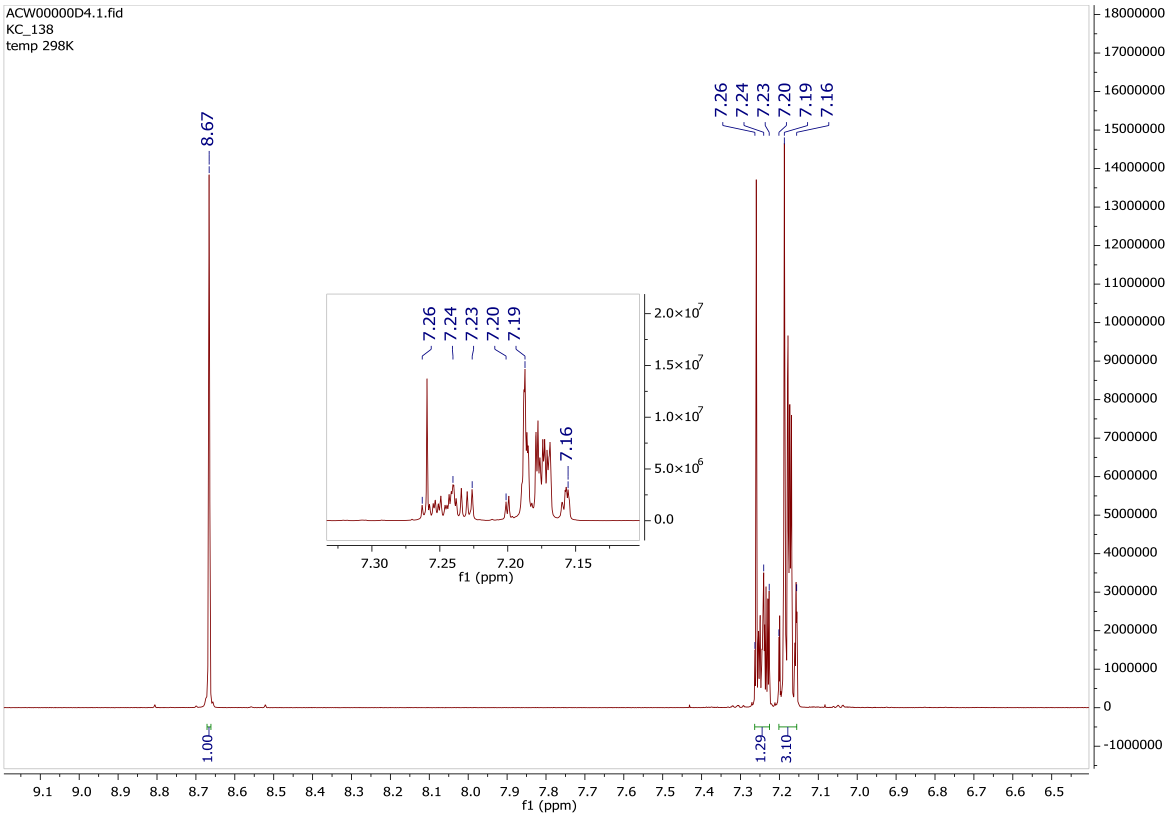

Figure S22. ^{1}**H NMR spectrum of 4b (CDCl$_3$)**

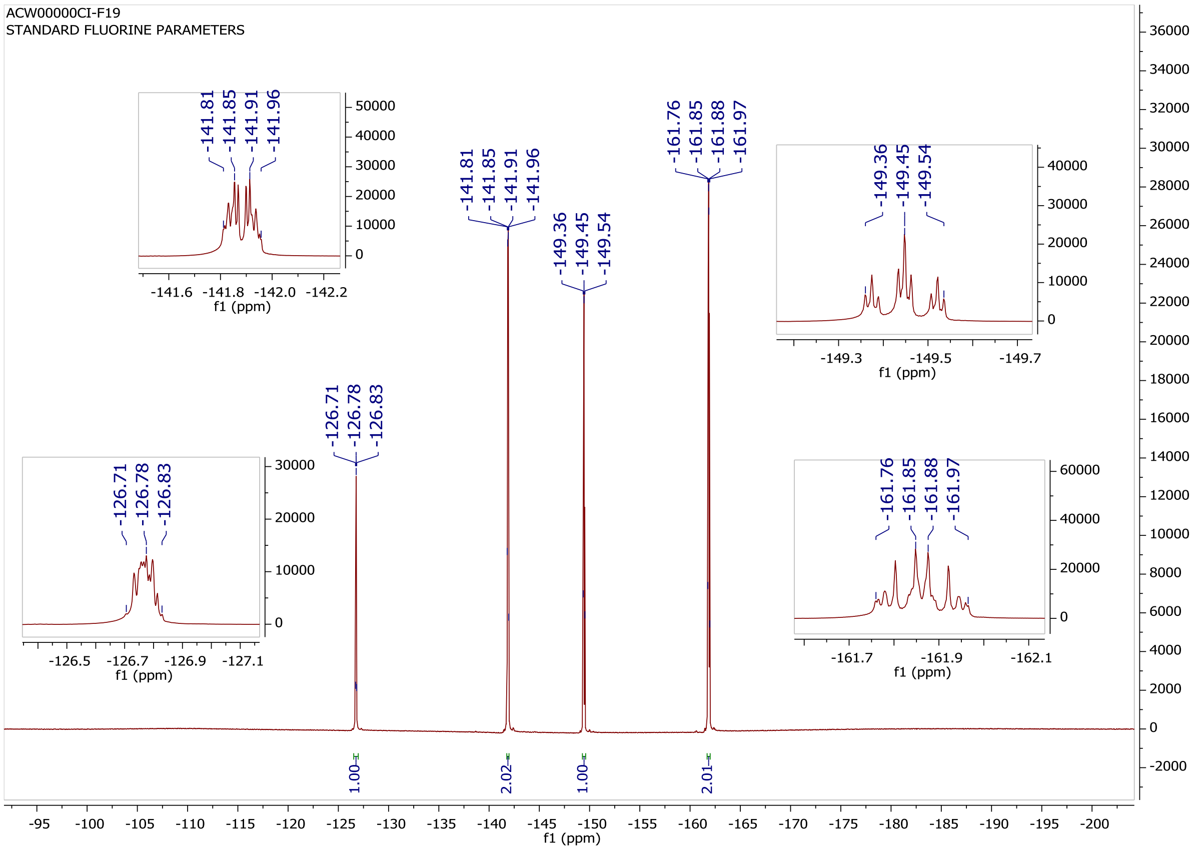

Figure S23. ^{19}F NMR spectrum of 4b ($CDCl_3$)

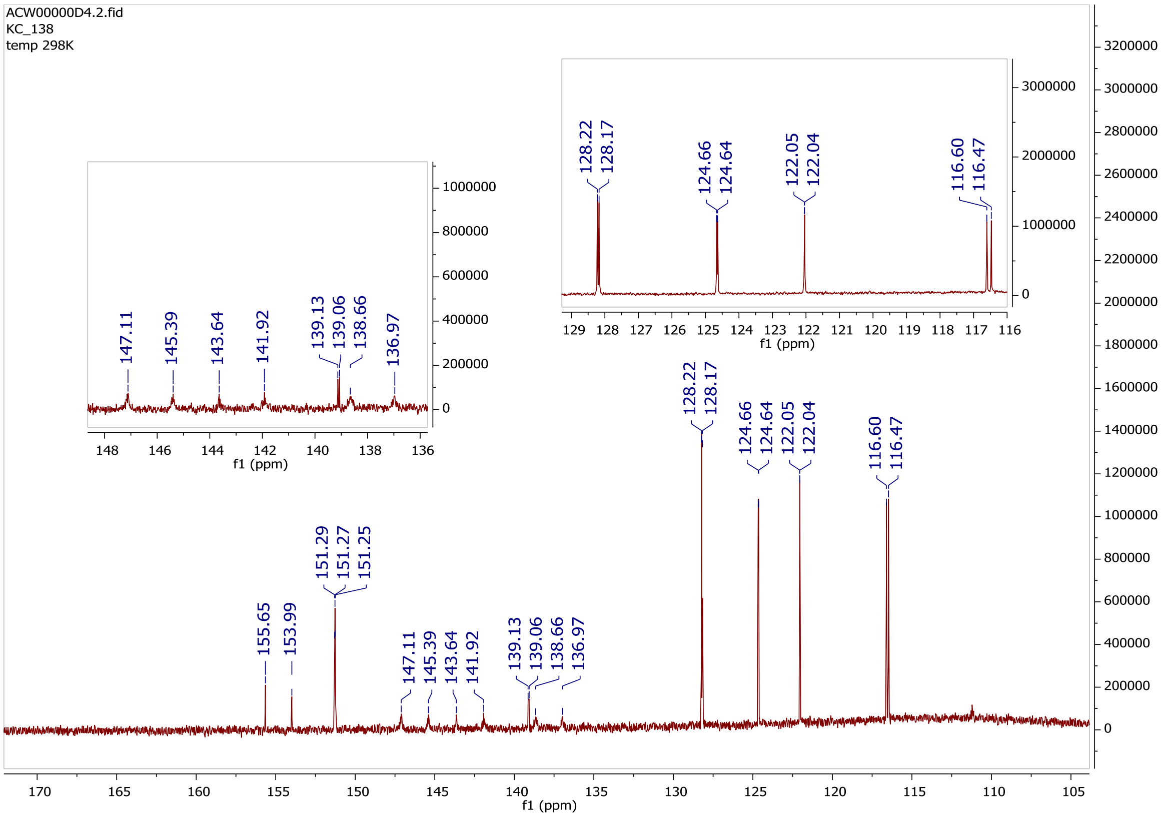

Figure S24. ^{13}C NMR spectrum of 4b ($CDCl_3$)

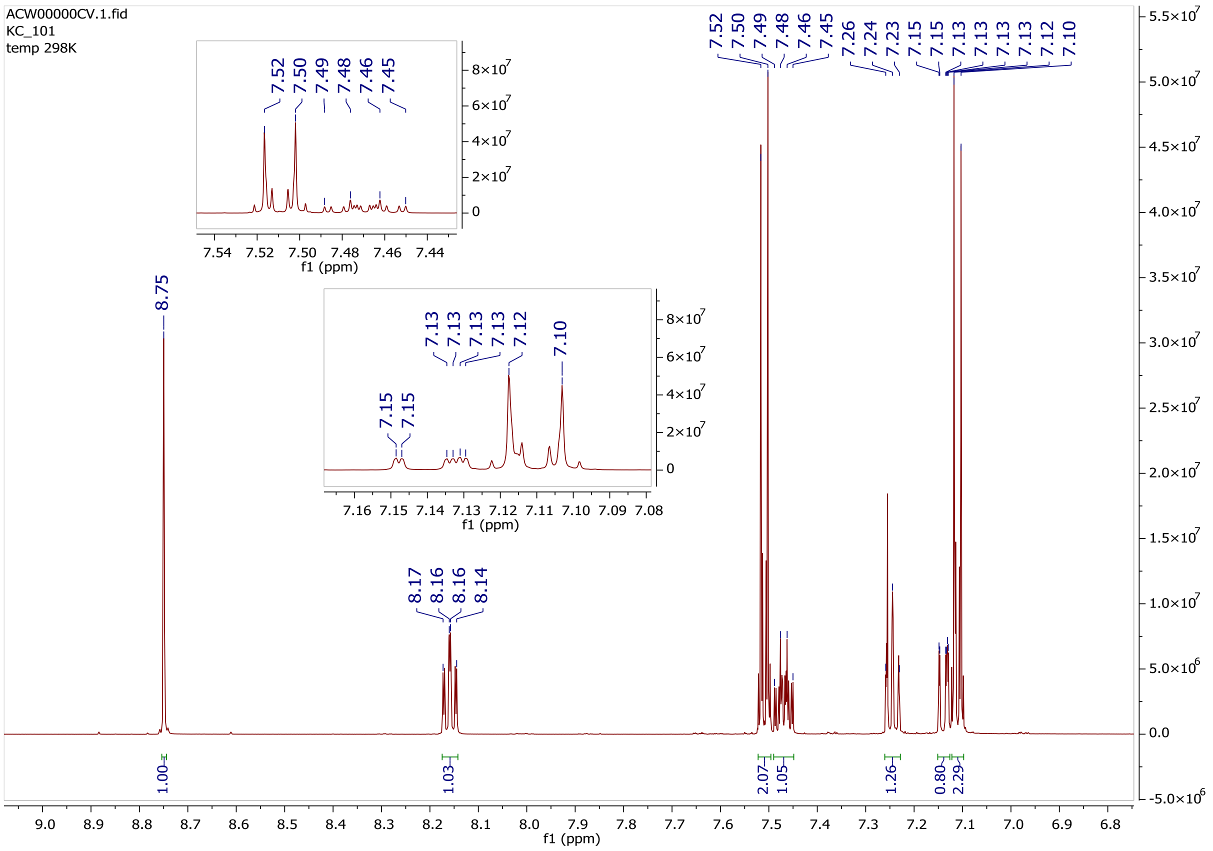

Figure S25. ^{1}H NMR spectrum of 1c ($CDCl_3$)

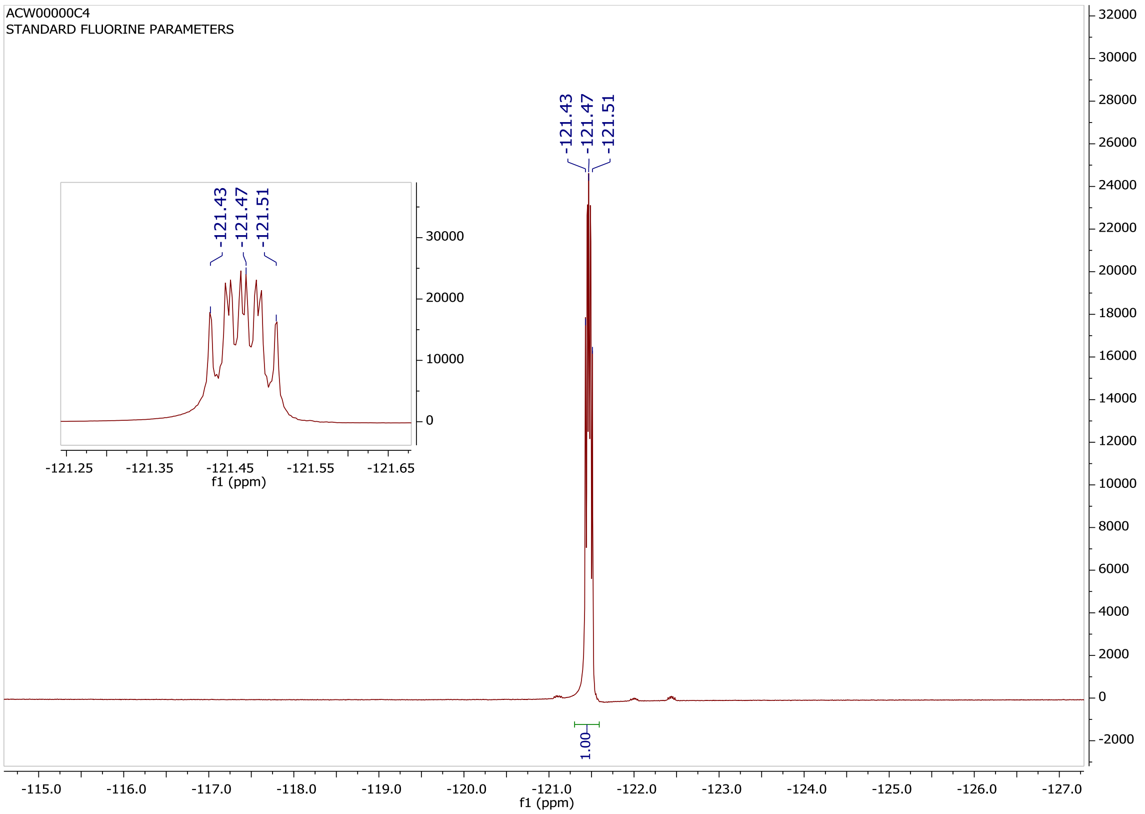

Figure S26. ^{19}F NMR spectrum of 1c ($CDCl_3$)

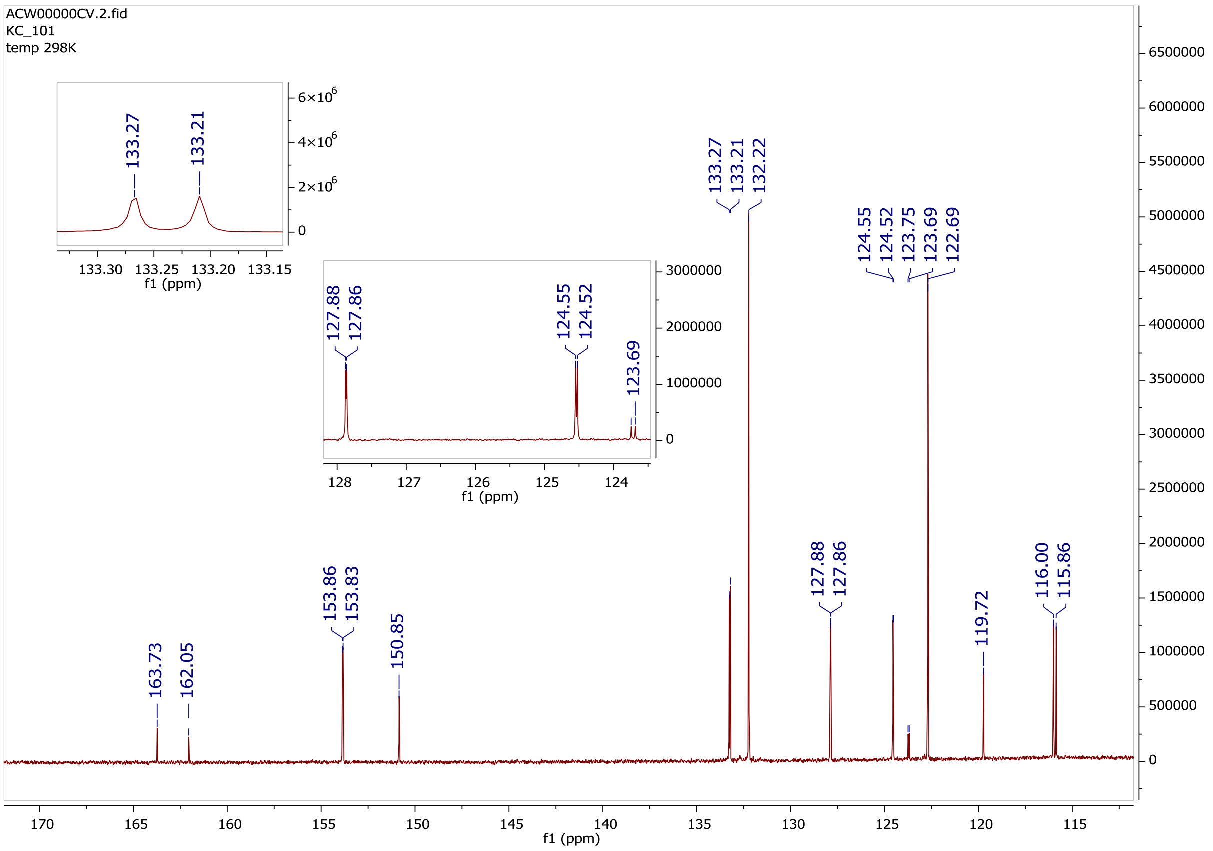

Figure S27. ^{13}**C NMR spectrum of 1c (CDCl$_3$)**

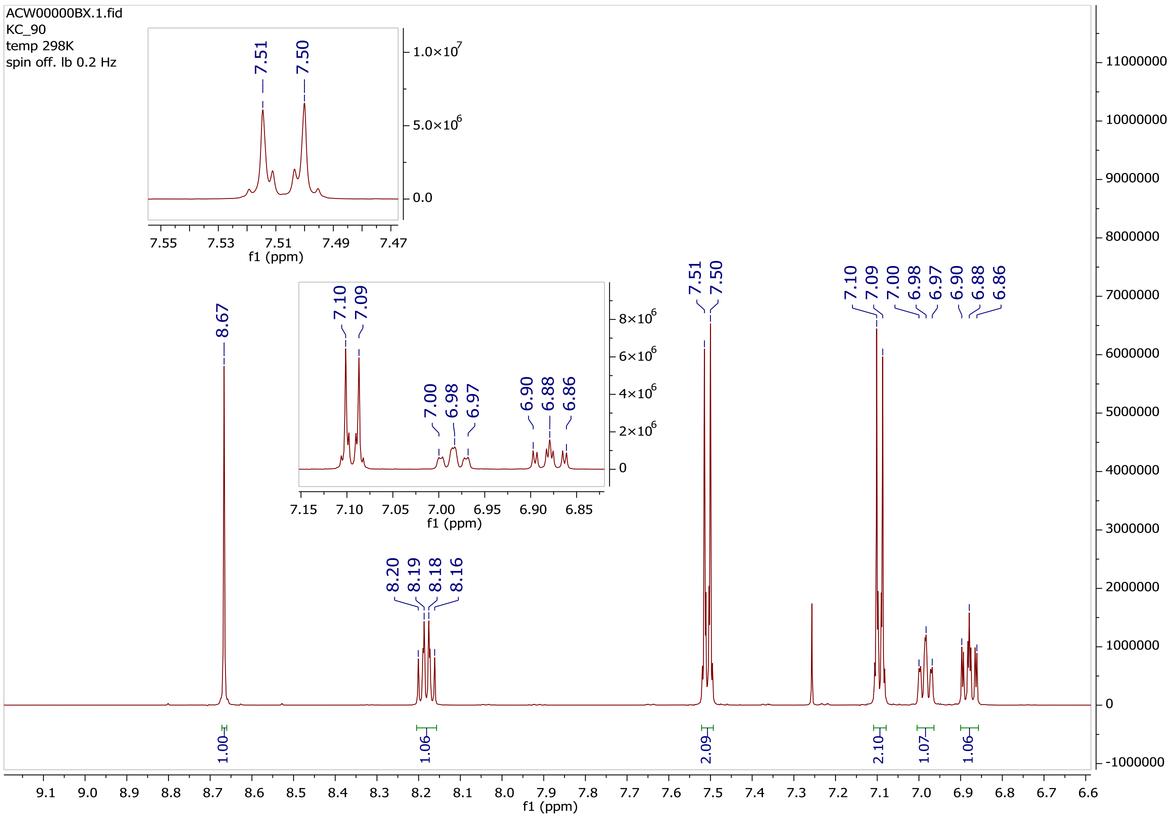

Figure S28. ^{1}**H NMR spectrum of 2c (CDCl$_3$)**

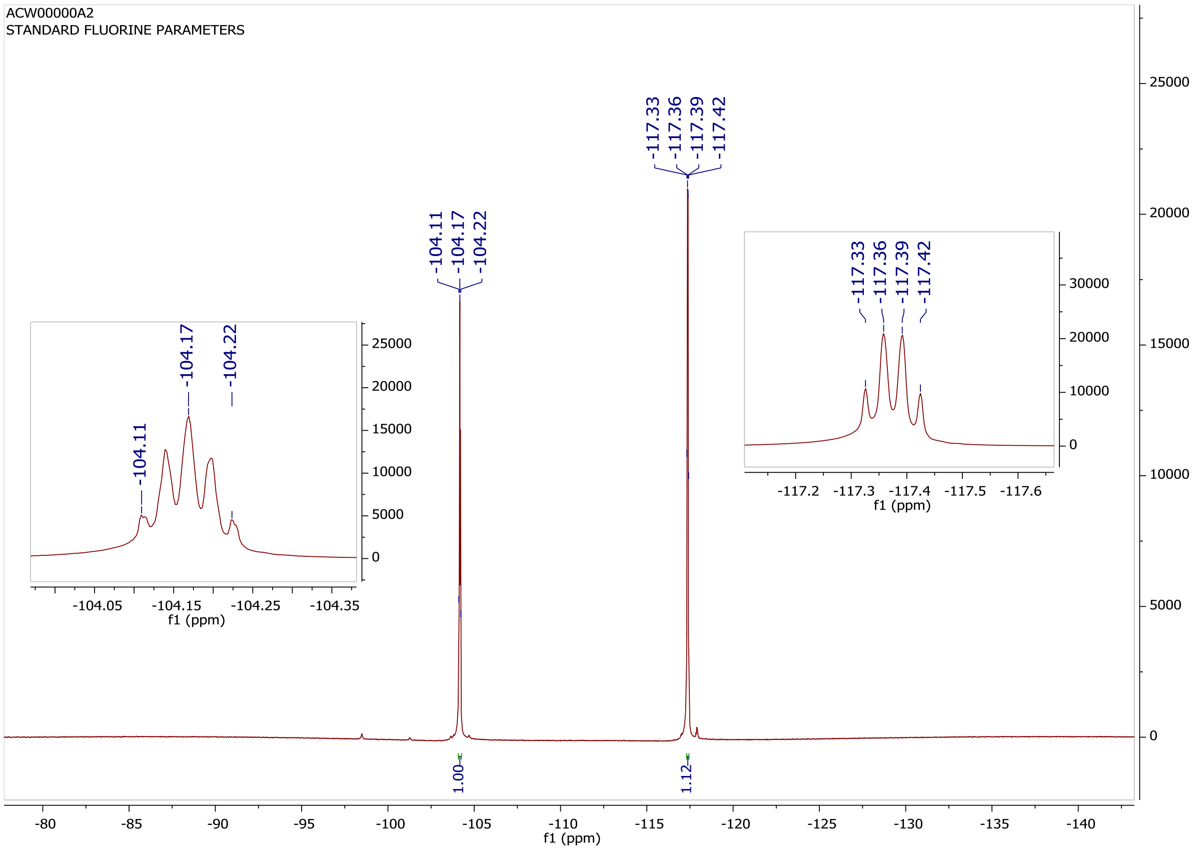

Figure S29. ^{19}**F NMR spectrum of 2c (CDCl$_3$)**

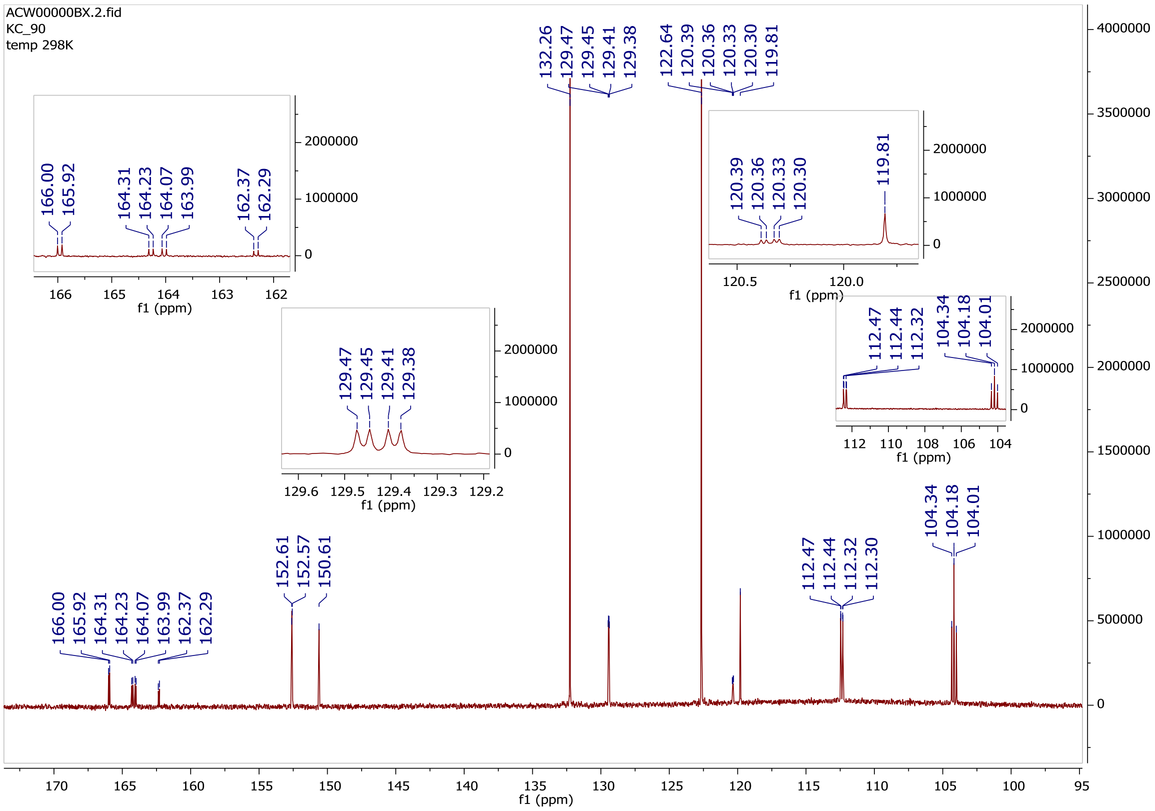

Figure S30. ^{13}**C NMR spectrum of 2c (CDCl$_3$)**

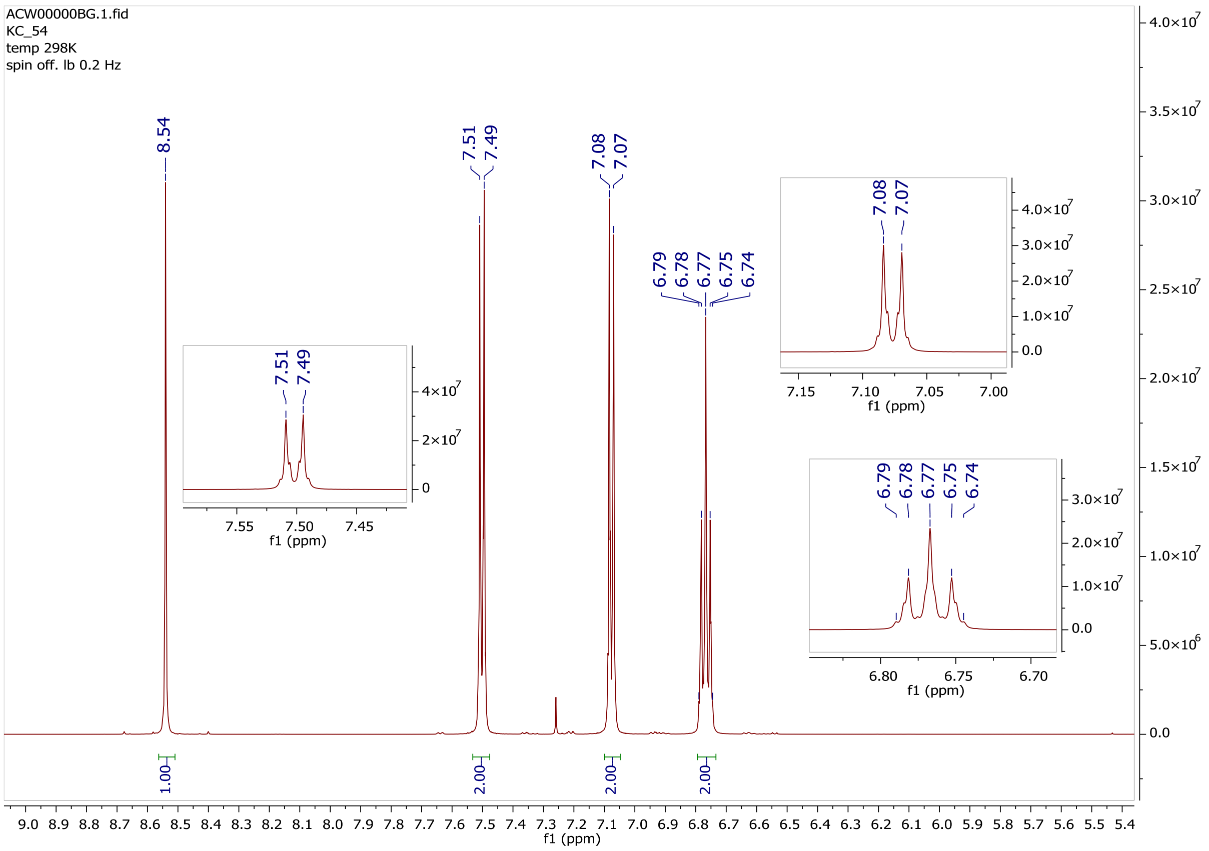

Figure S31. ^{1}H NMR spectrum of 3c ($CDCl_3$)

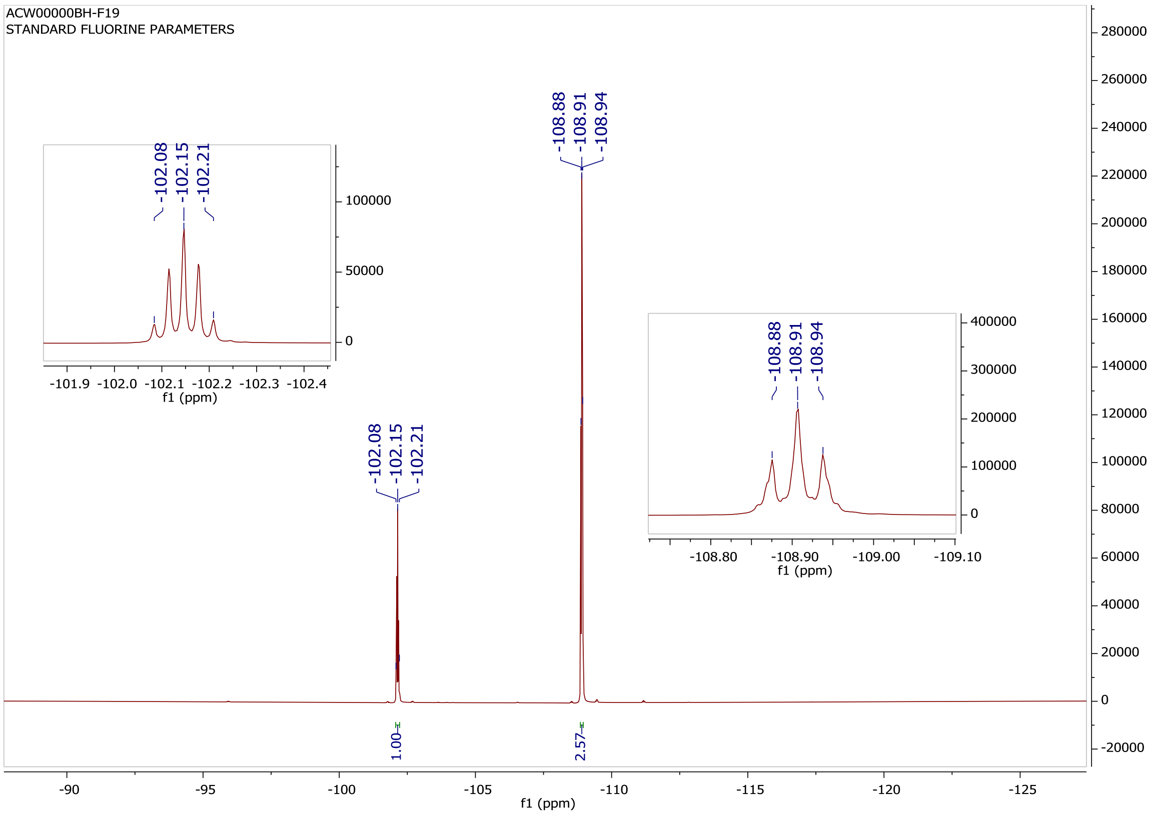

Figure S32. ^{19}F NMR spectrum of 3c ($CDCl_3$)

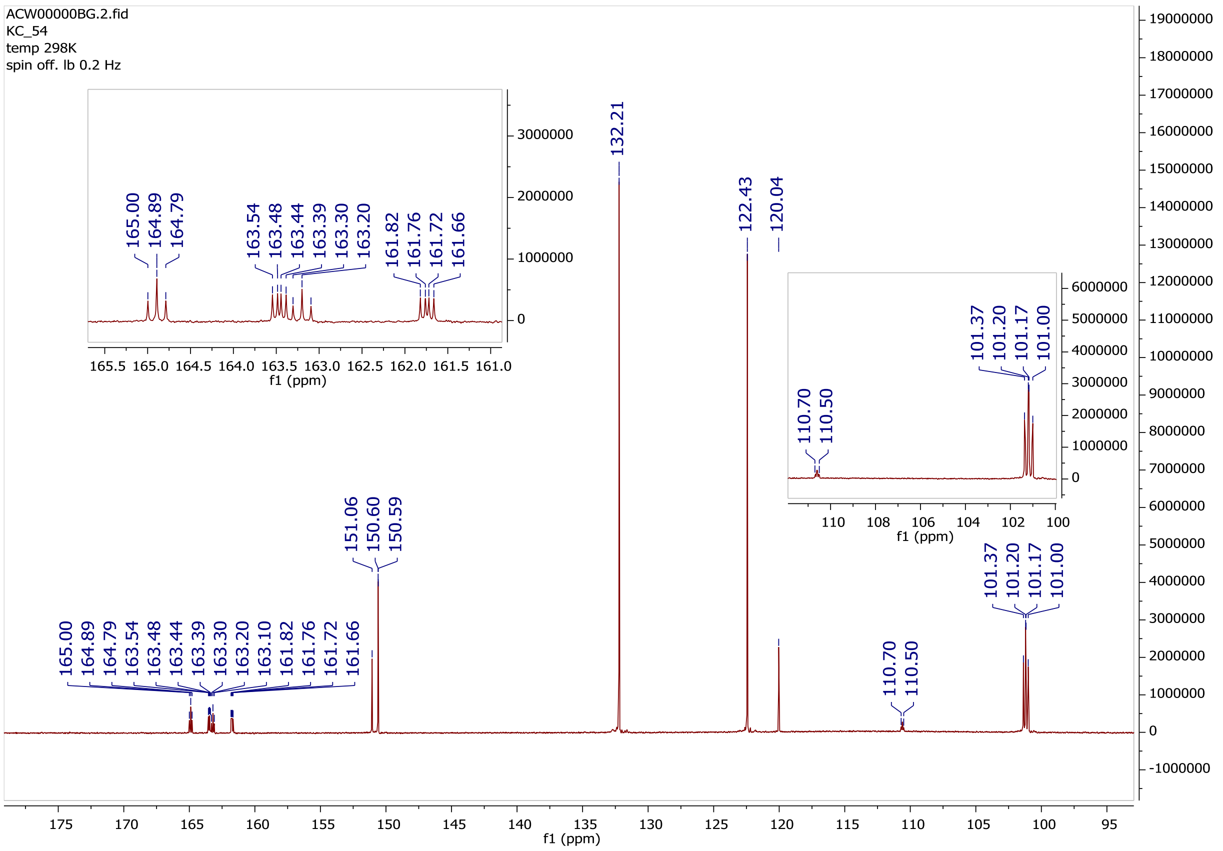

Figure S33. ^{13}C NMR spectrum of 3c ($CDCl_3$)

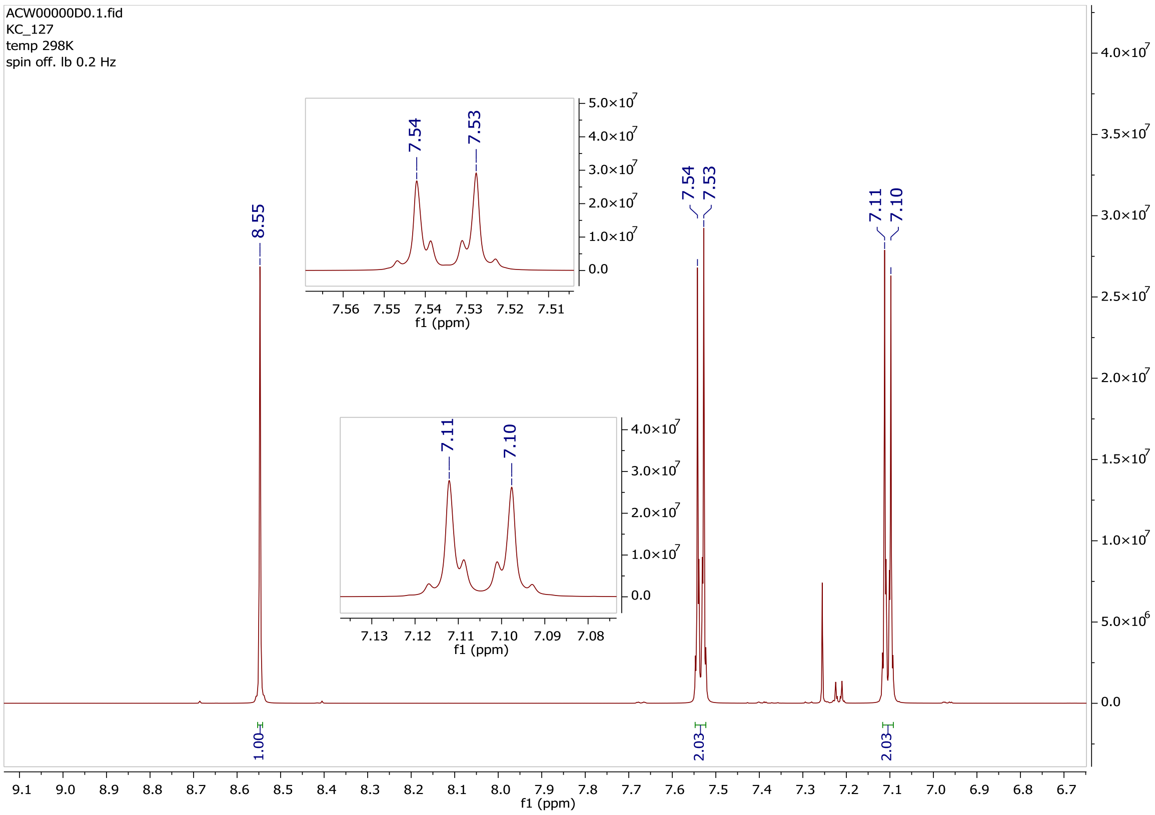

Figure S34. ^{1}H NMR spectrum of 4c ($CDCl_3$)

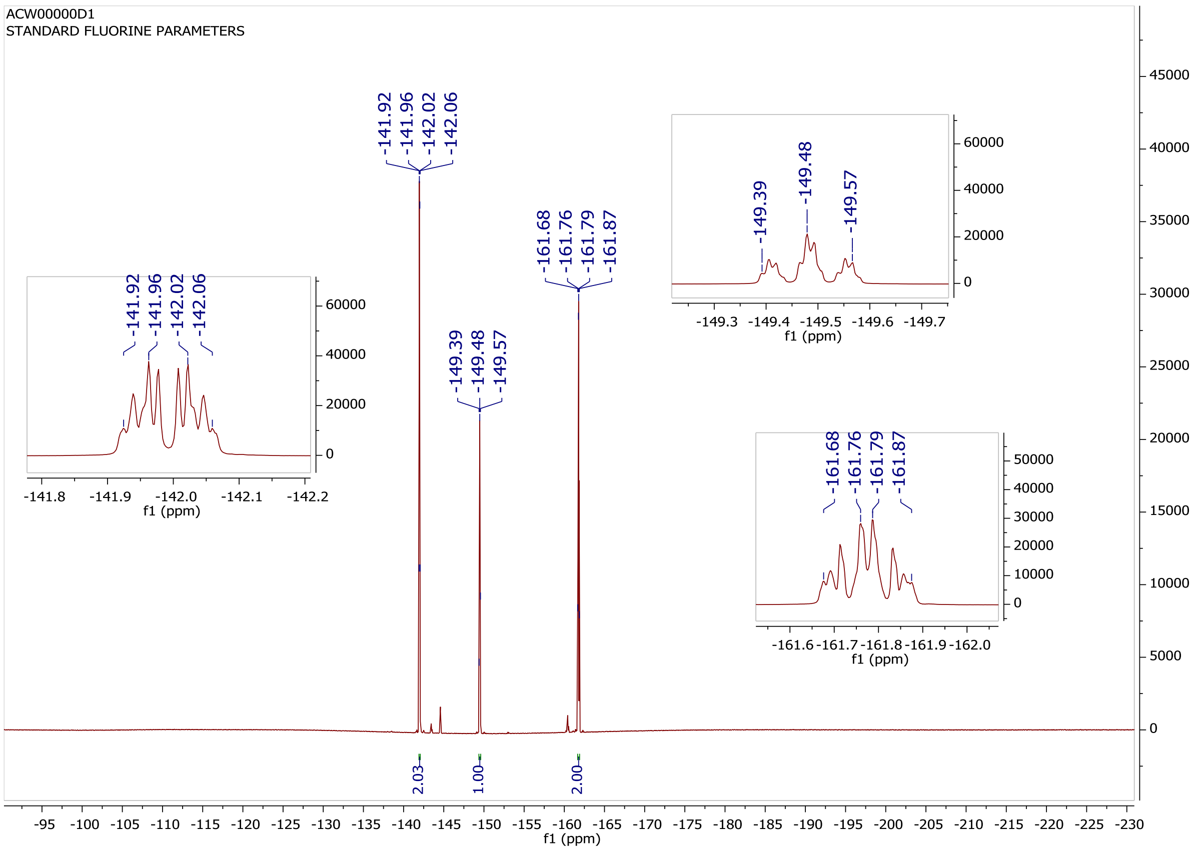

Figure S35. ^{19}F NMR spectrum of 4c ($CDCl_3$)

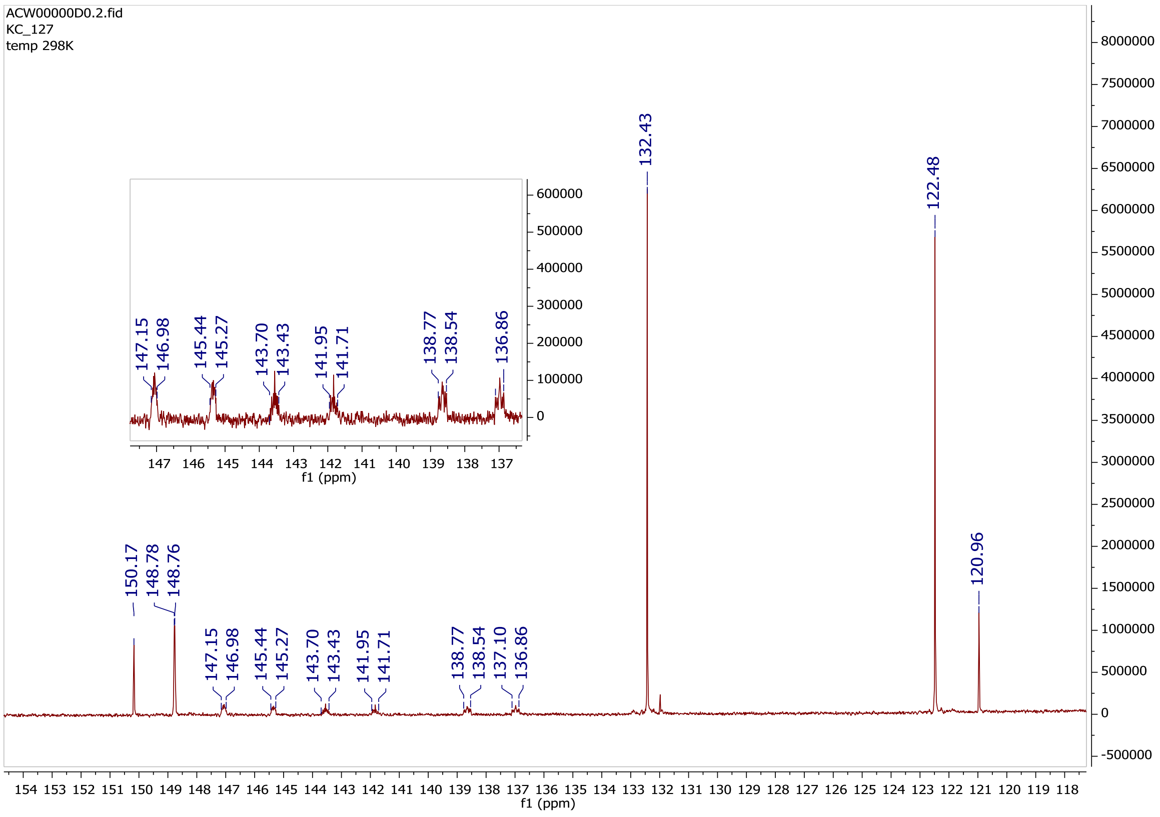

Figure S36. ^{13}C NMR spectrum of 4c ($CDCl_3$)

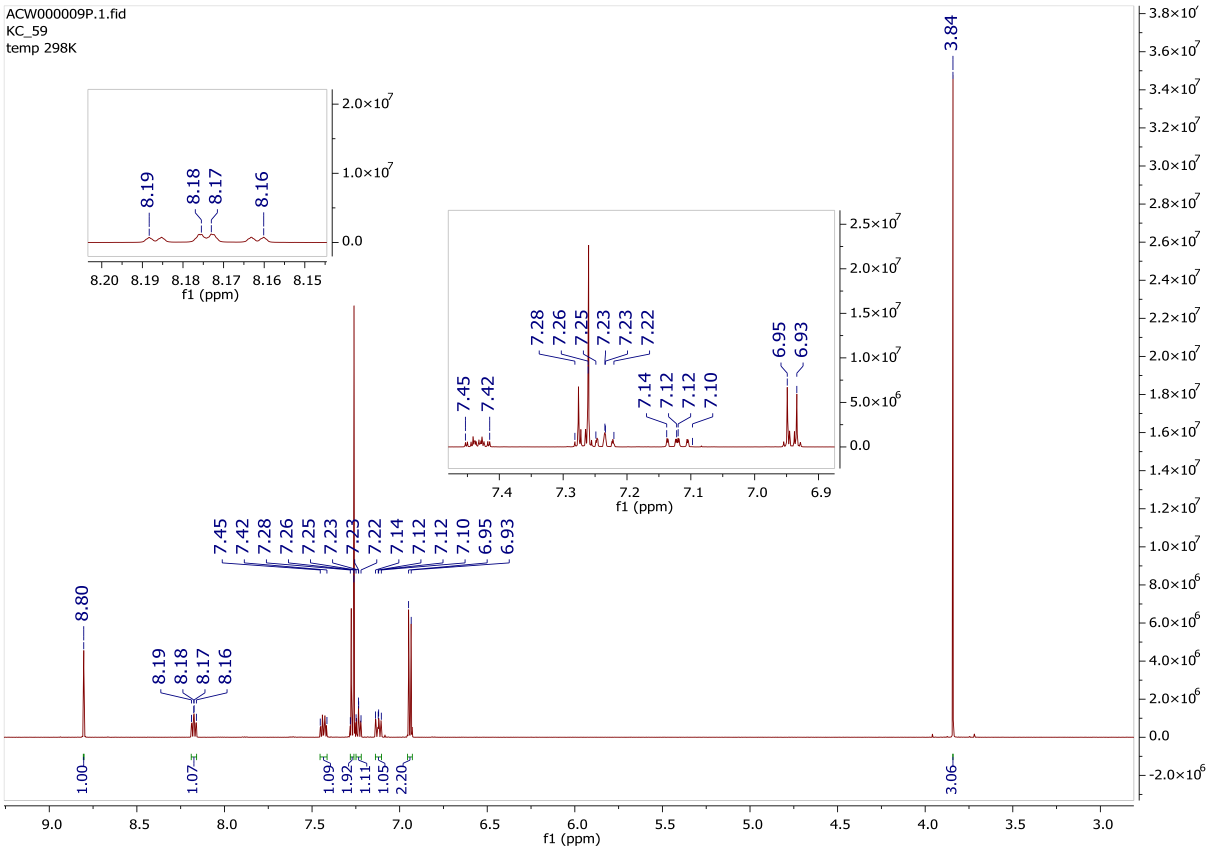

Figure S37. ^{1}H **NMR spectrum of 1d** ($CDCl_3$)

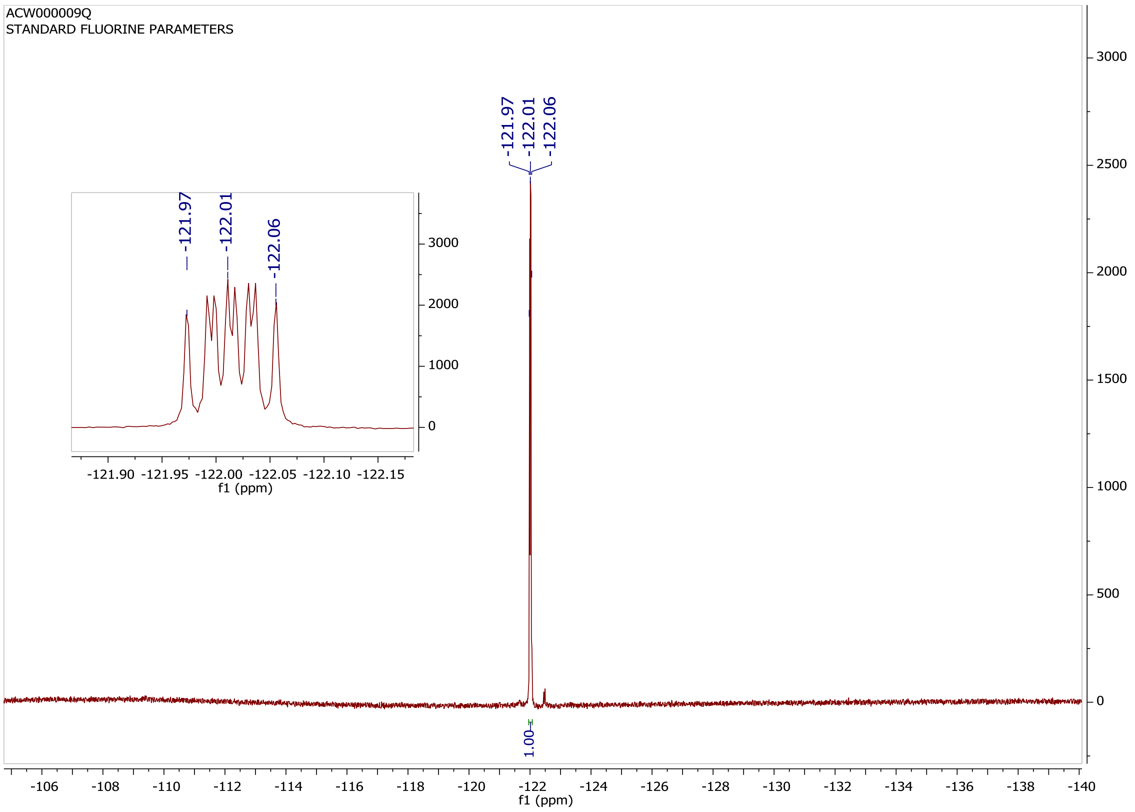

Figure S38. ^{19}F **NMR spectrum of 1d** ($CDCl_3$)

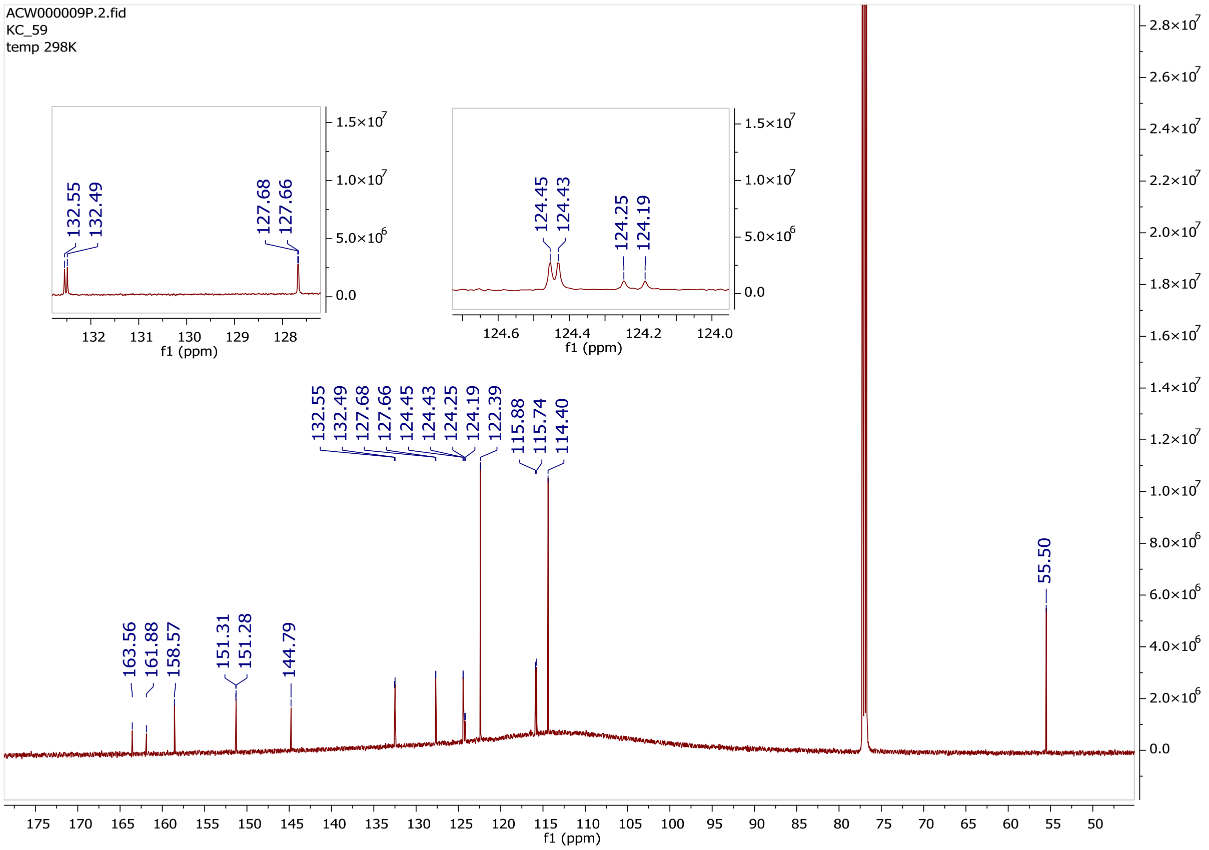

Figure S39. ^{13}**C NMR spectrum of 1d (CDCl$_3$)**

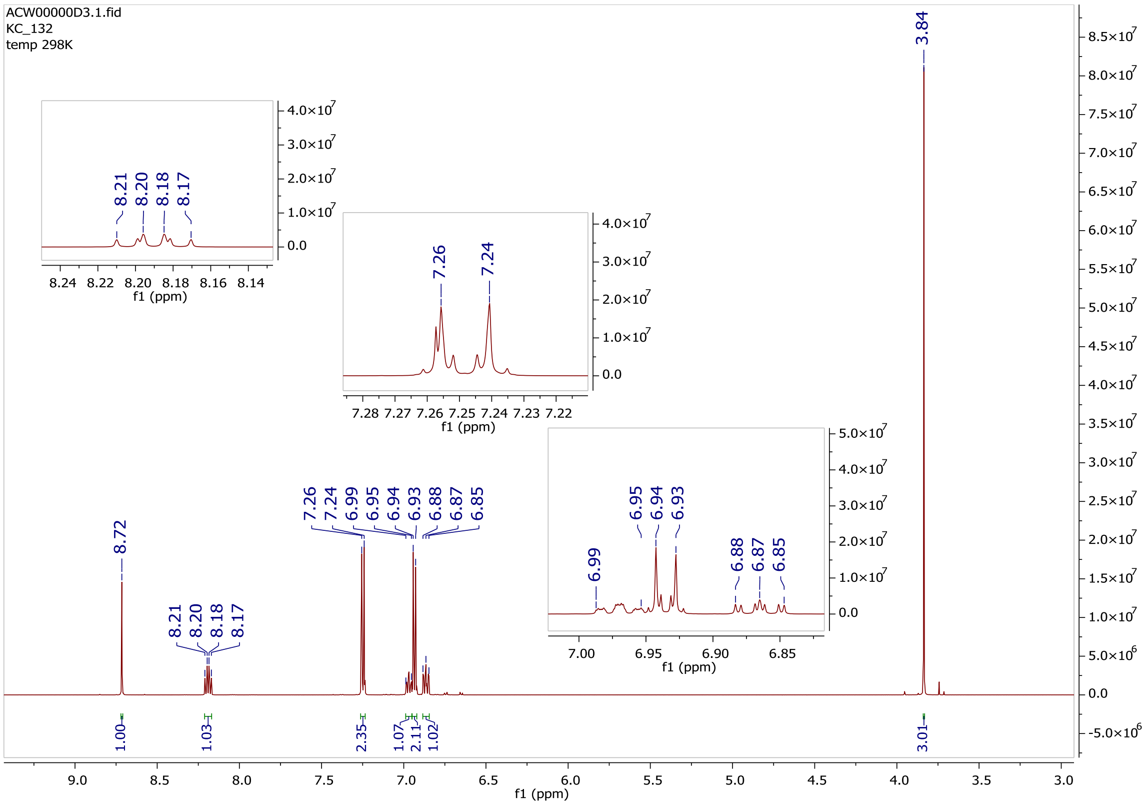

Figure S40. ^{1}**H NMR spectrum of 2d (CDCl$_3$)**

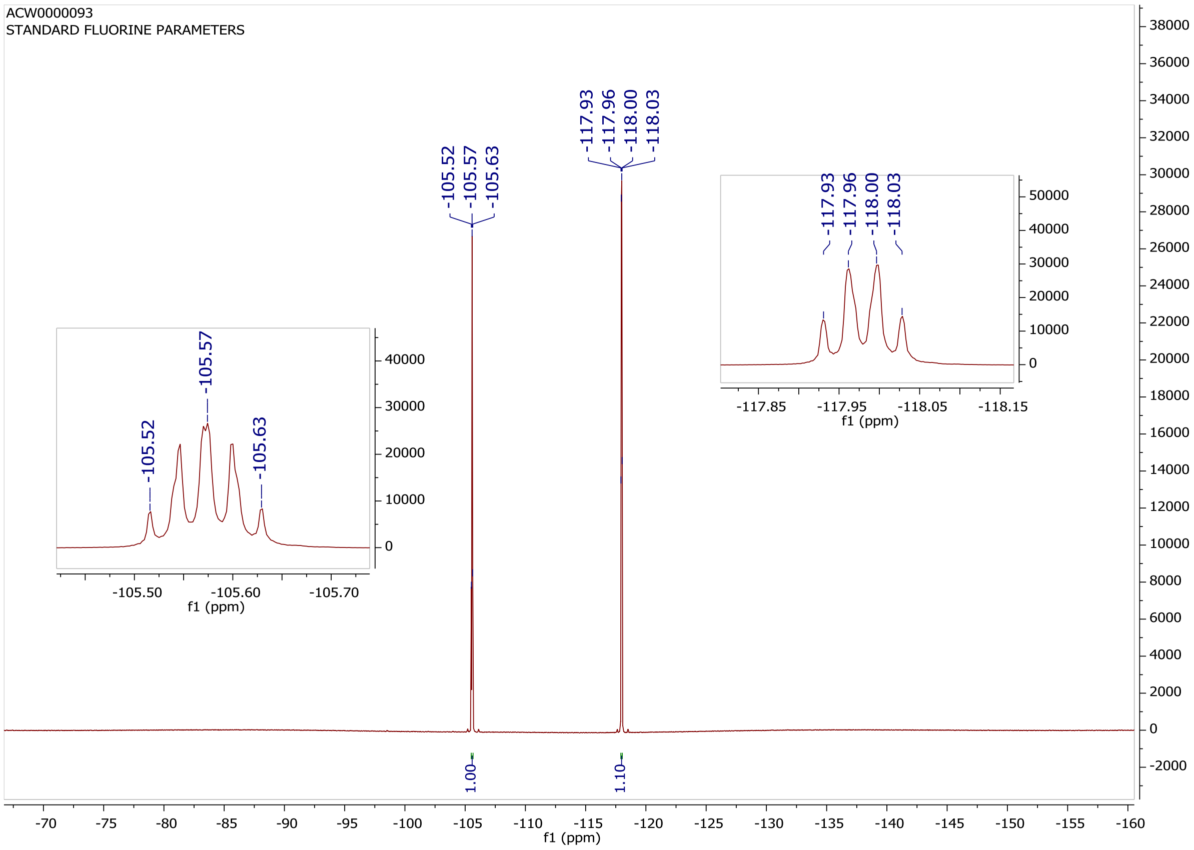

Figure S41. ^{19}**F NMR spectrum of 2d (CDCl$_3$)**

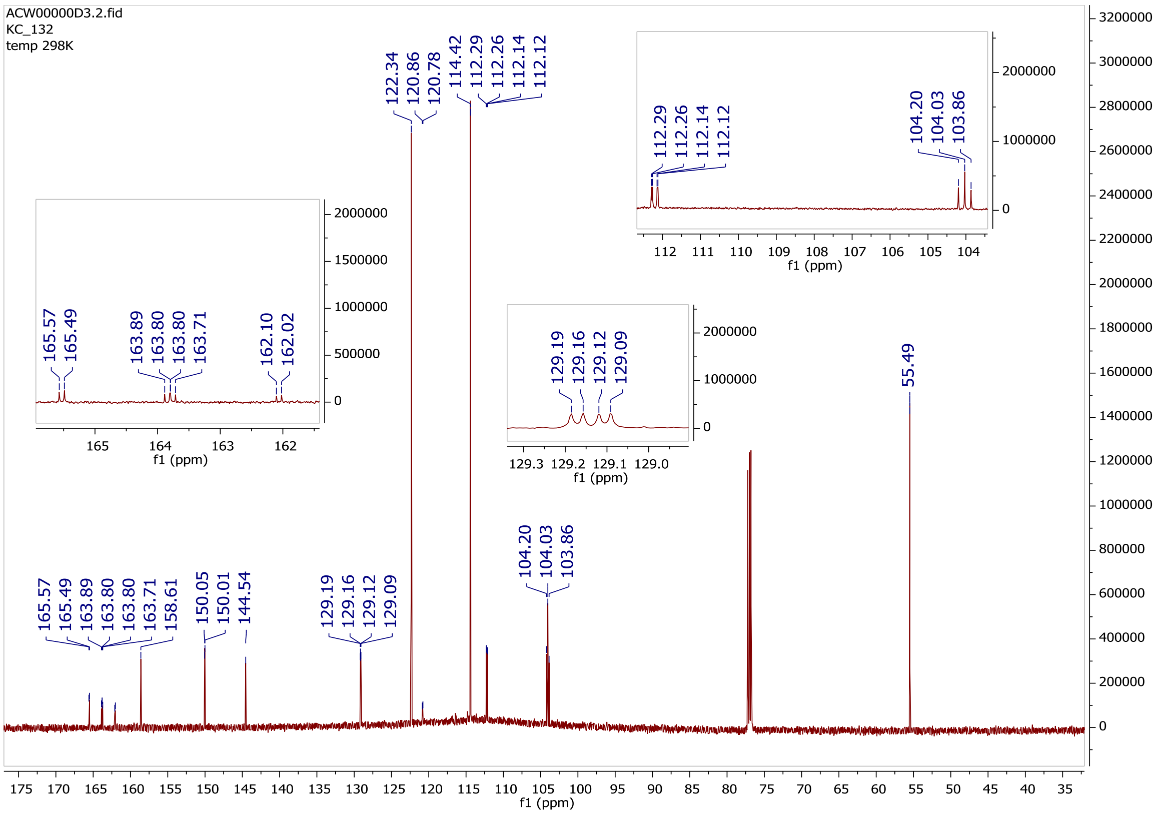

Figure S42. ^{13}**C NMR spectrum of 2d (CDCl$_3$)**

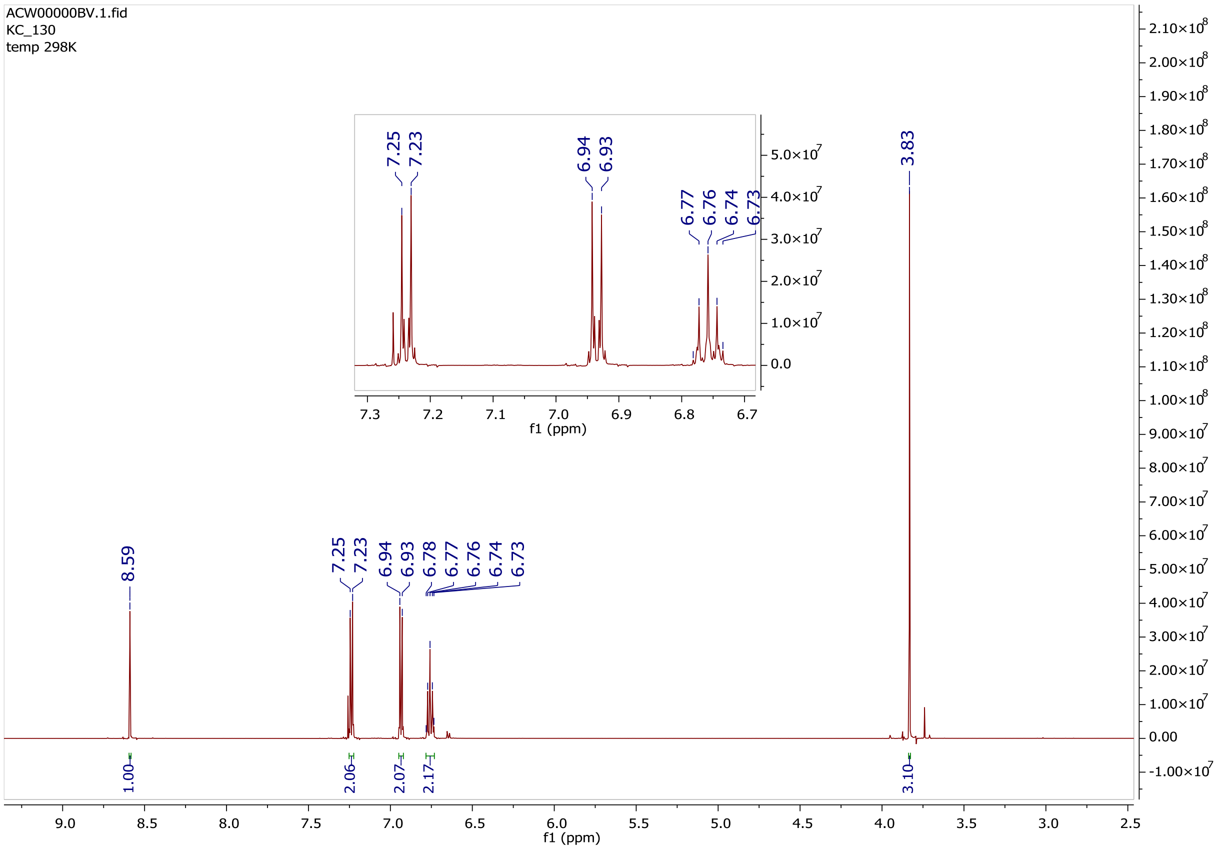

Figure S43. ^{1}H **NMR spectrum of 3d (CDCl$_3$)**

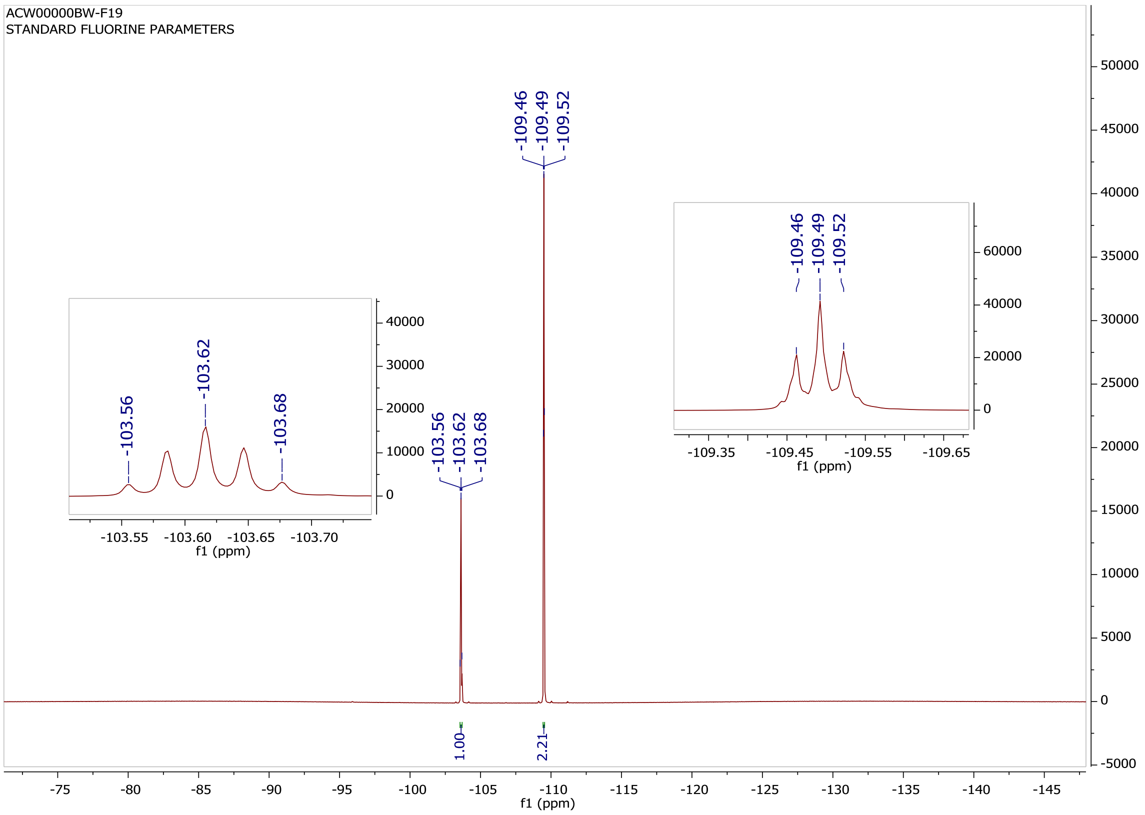

Figure S44. ^{19}F **NMR spectrum of 3d (CDCl$_3$)**

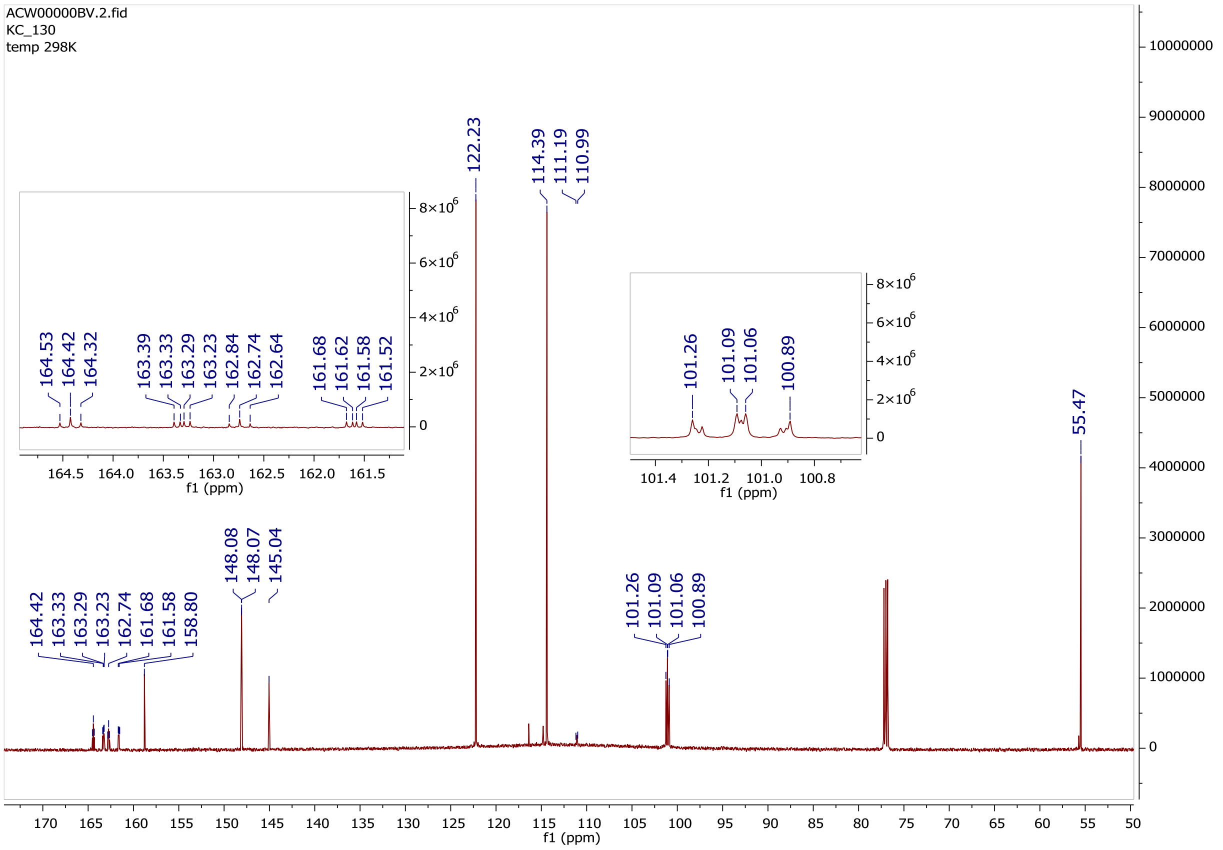

Figure S45. **^{13}C NMR spectrum of 3d ($CDCl_3$)**

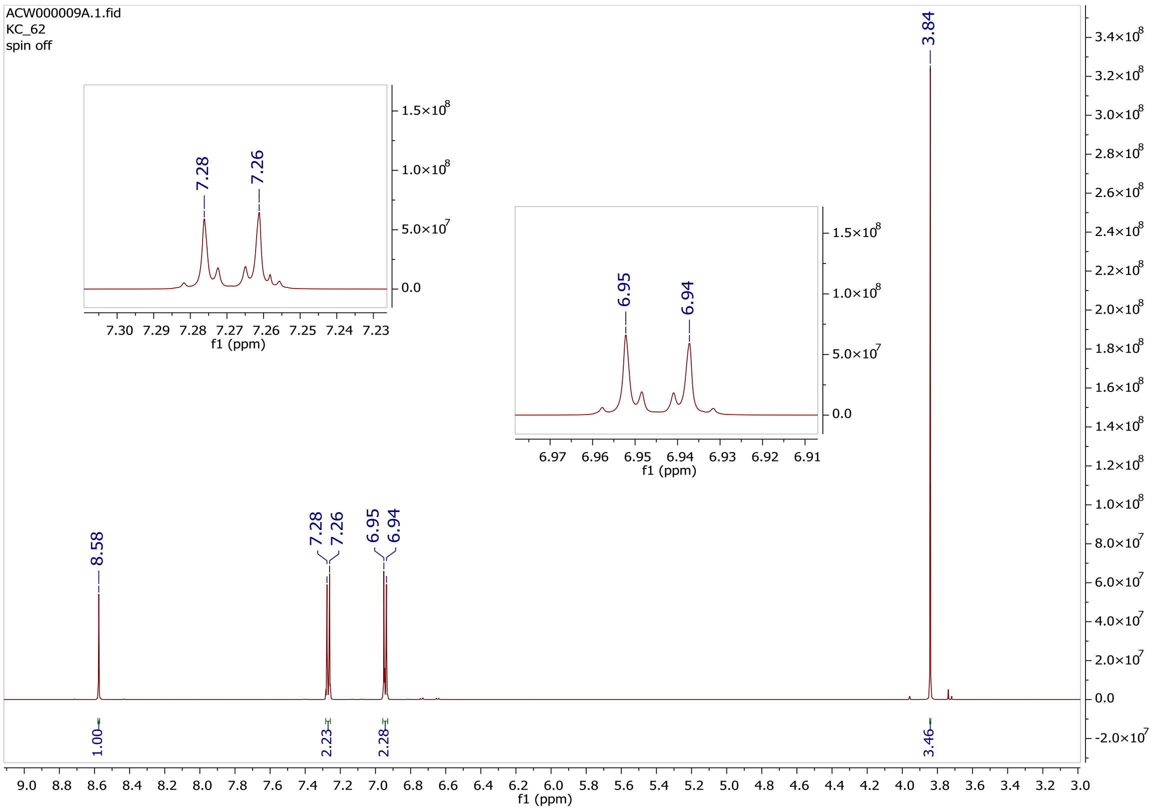

Figure S46. **^{1}H NMR spectrum of 4d ($CDCl_3$)**

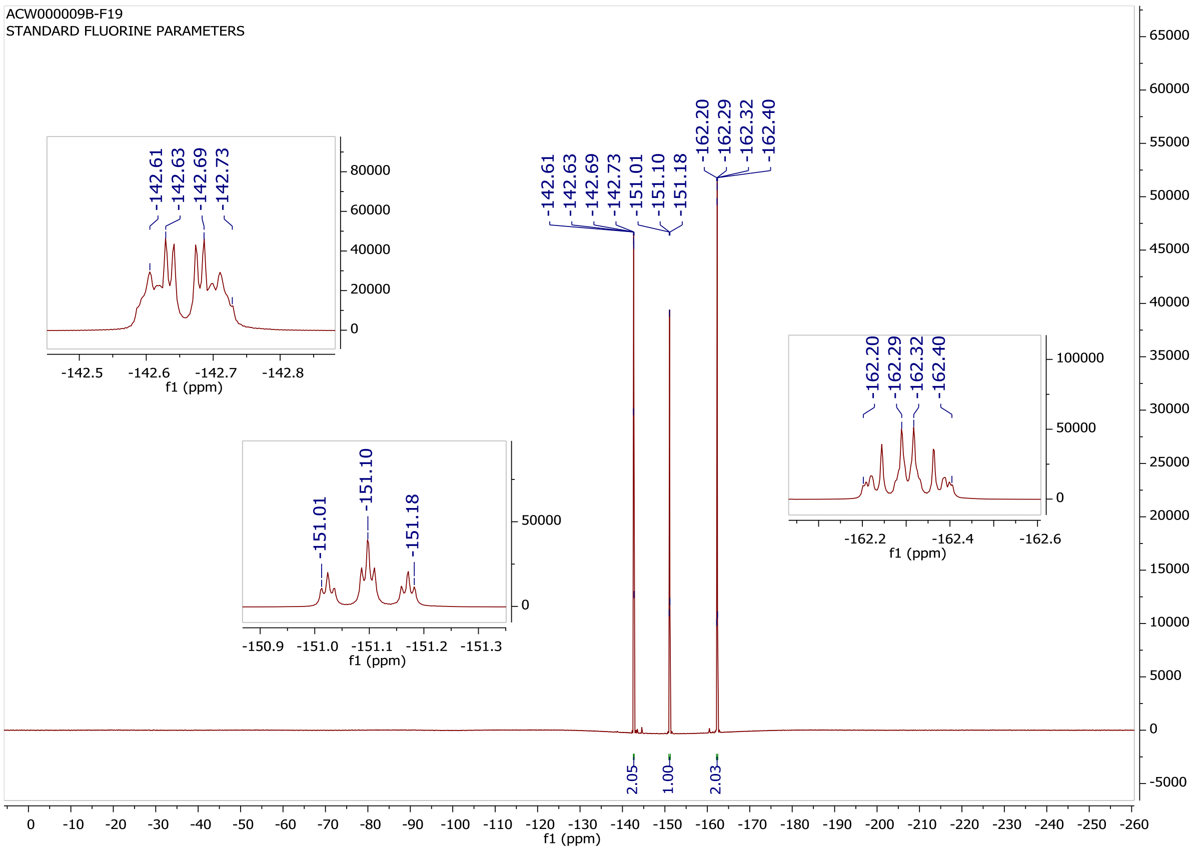

Figure S47. ^{19}F NMR spectrum of 4d ($CDCl_3$)

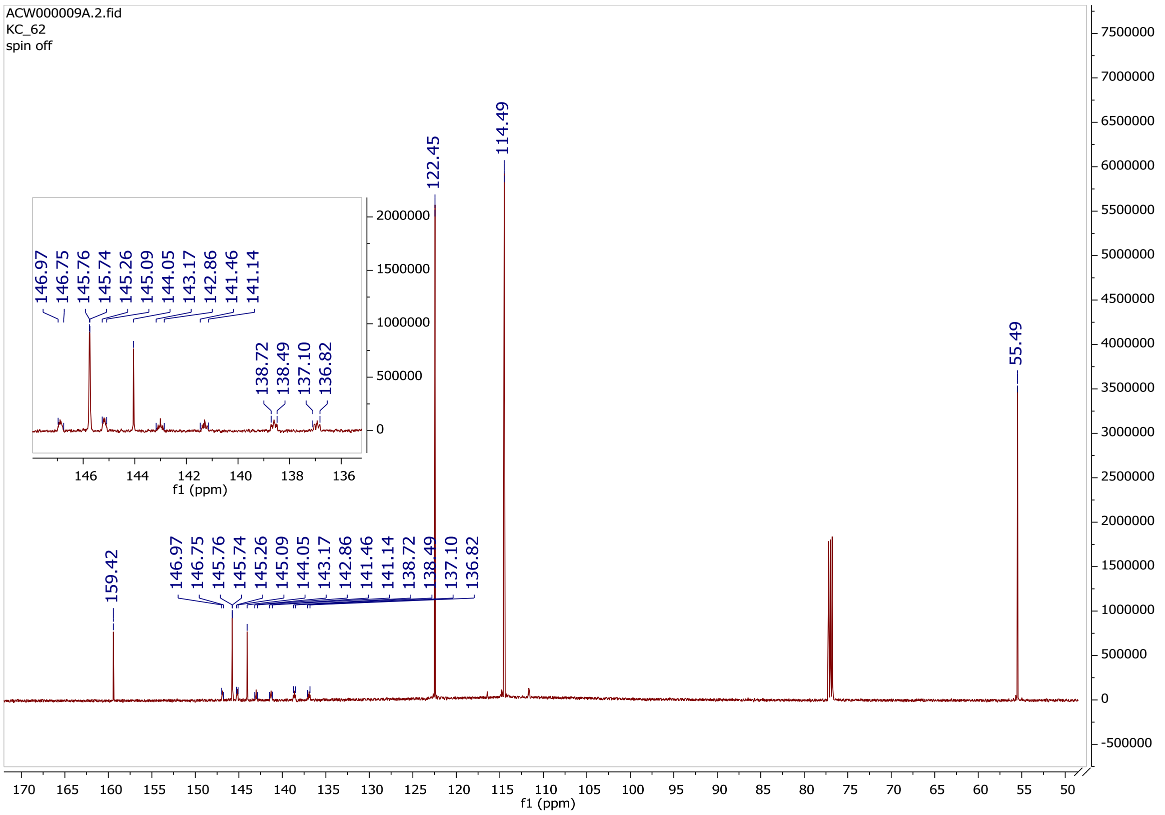

Figure S48. ^{13}C NMR spectrum of 4d ($CDCl_3$)

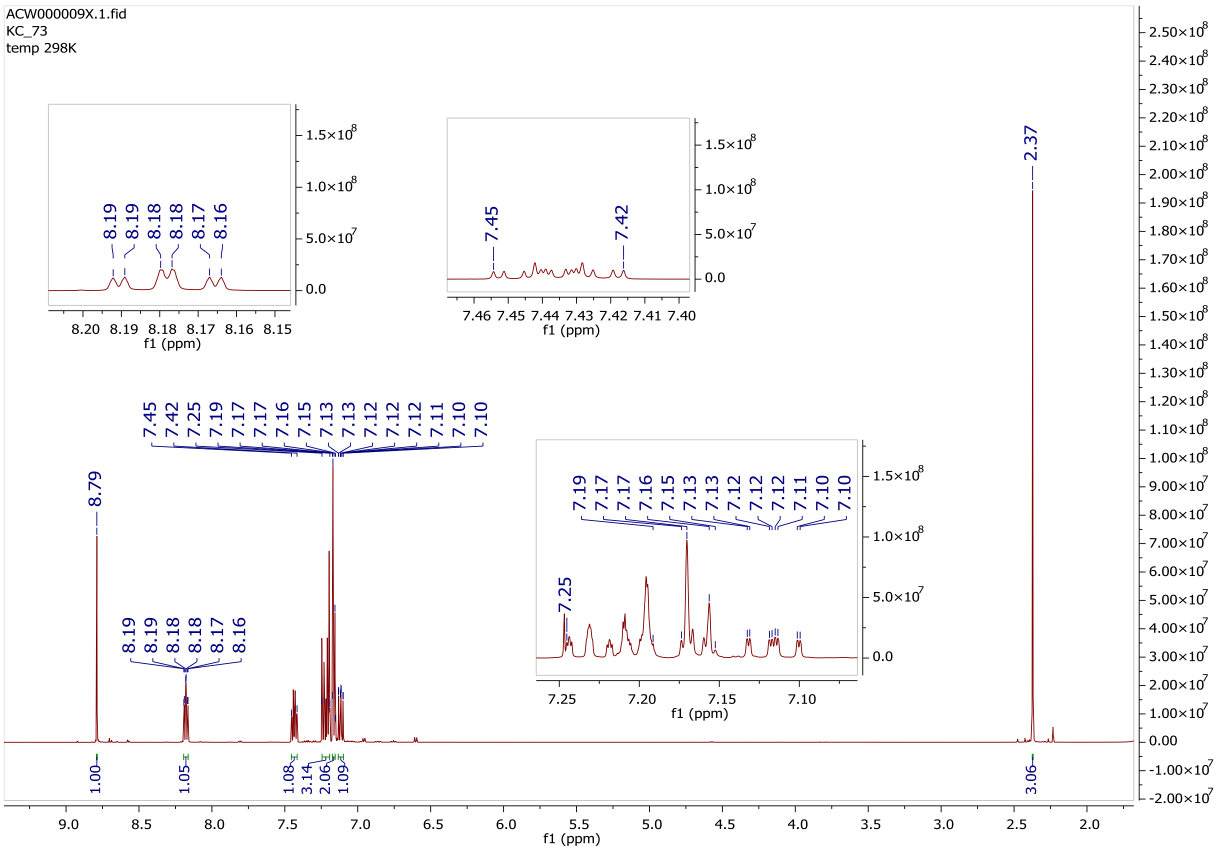

Figure S49. ^{1}H **NMR spectrum of 1e ($CDCl_3$)**

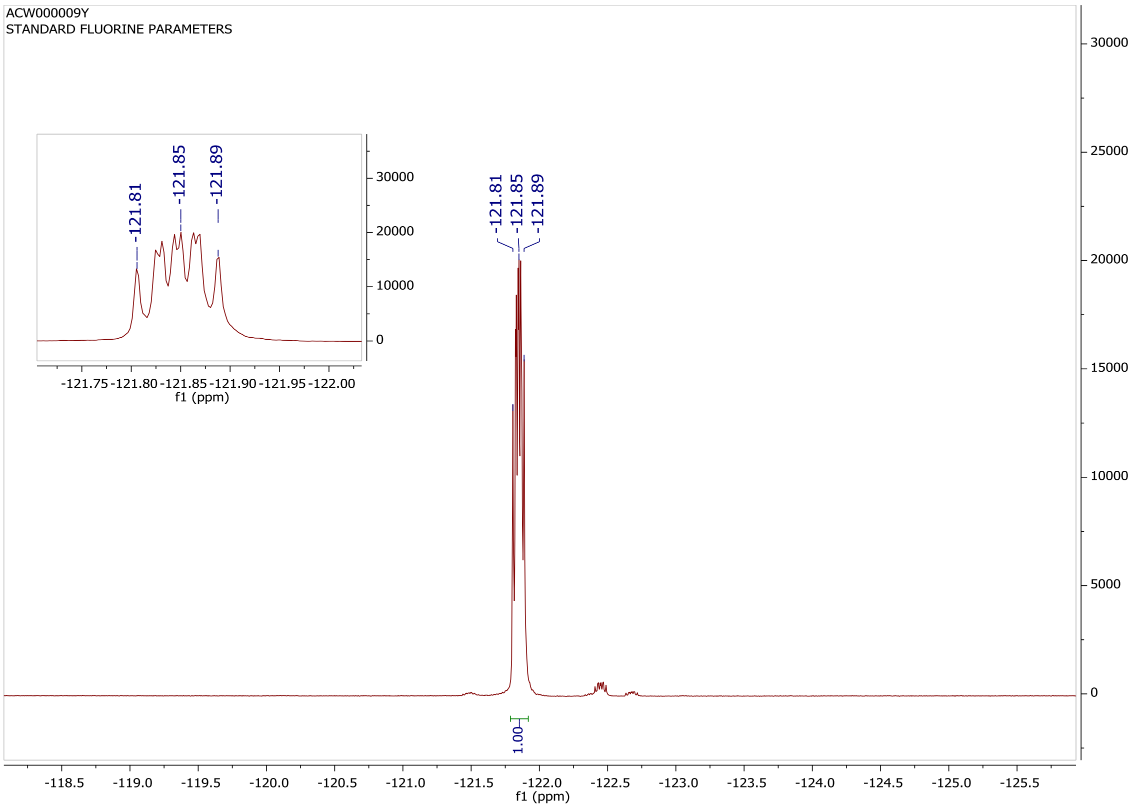

Figure S50. ^{19}F **NMR spectrum of 1e ($CDCl_3$)**

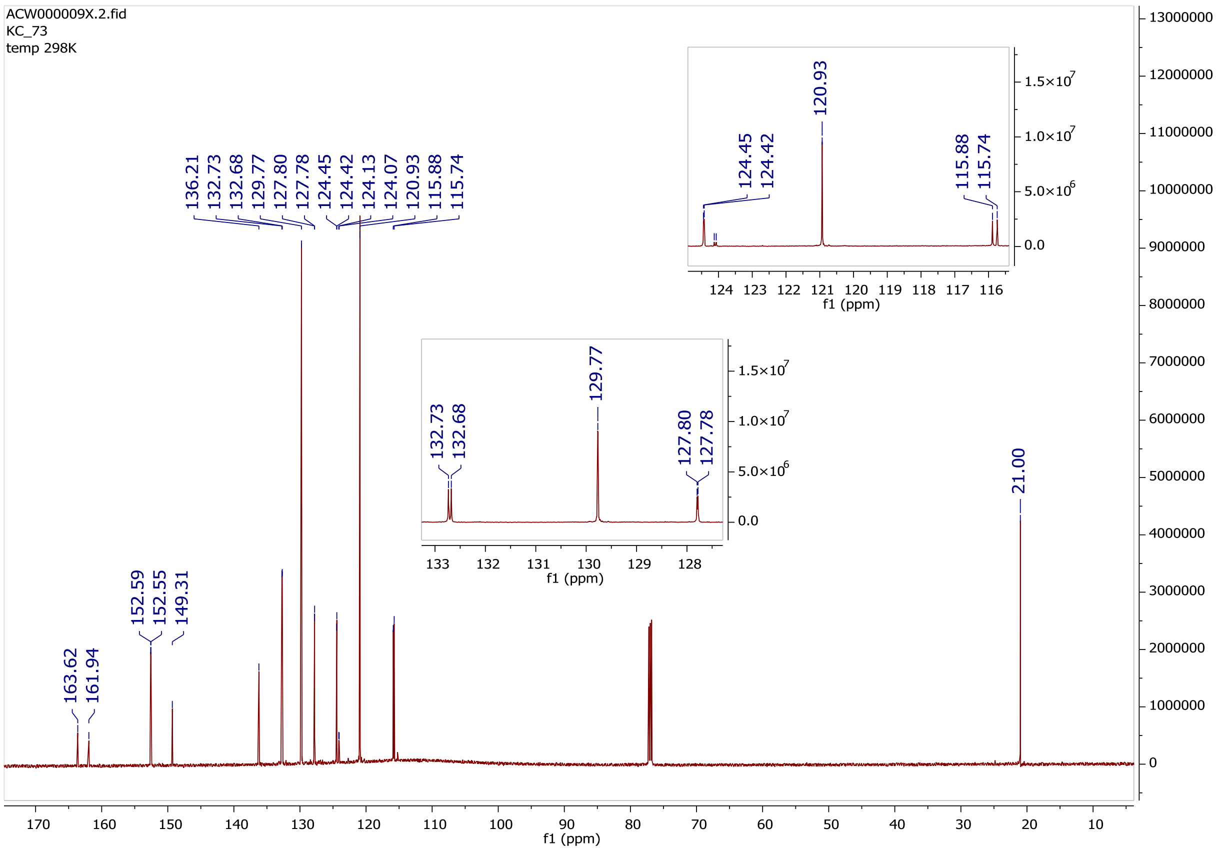

Figure S51. ^{13}**C NMR spectrum of 1e (**$CDCl_3$**)**

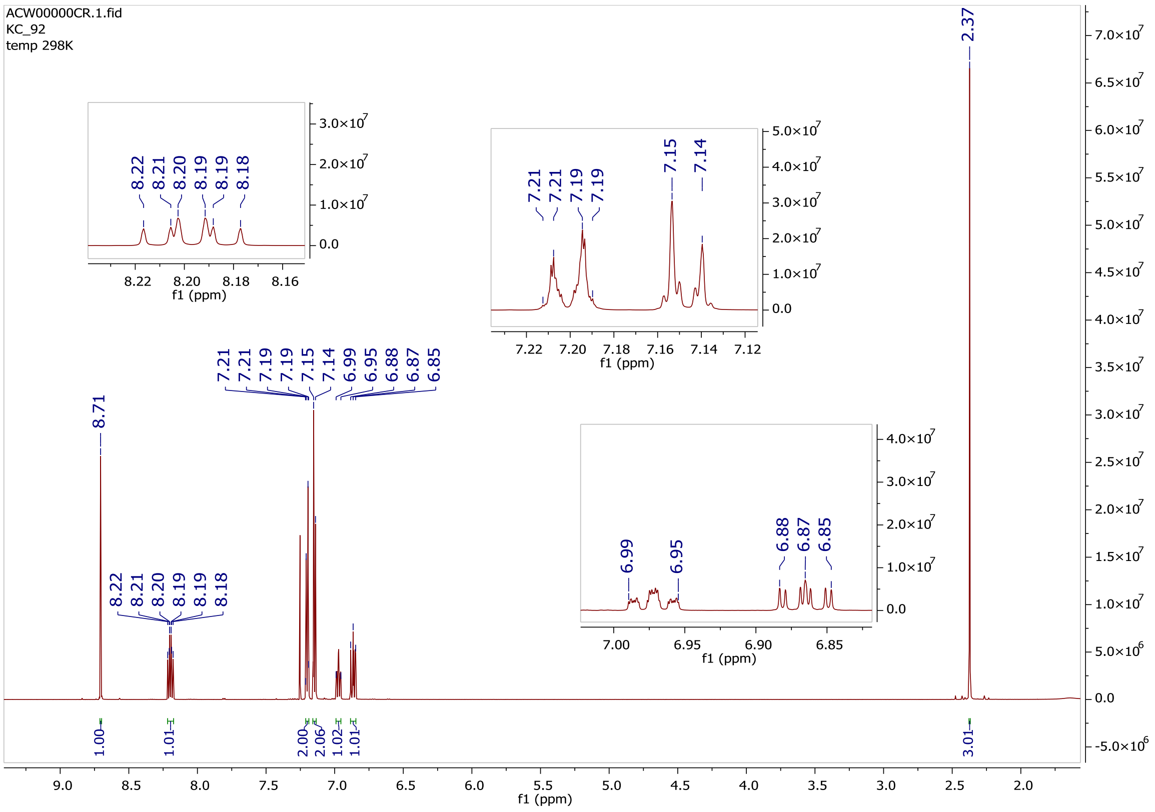

Figure S52. ^{1}**H NMR spectrum of 2e (**$CDCl_3$**)**

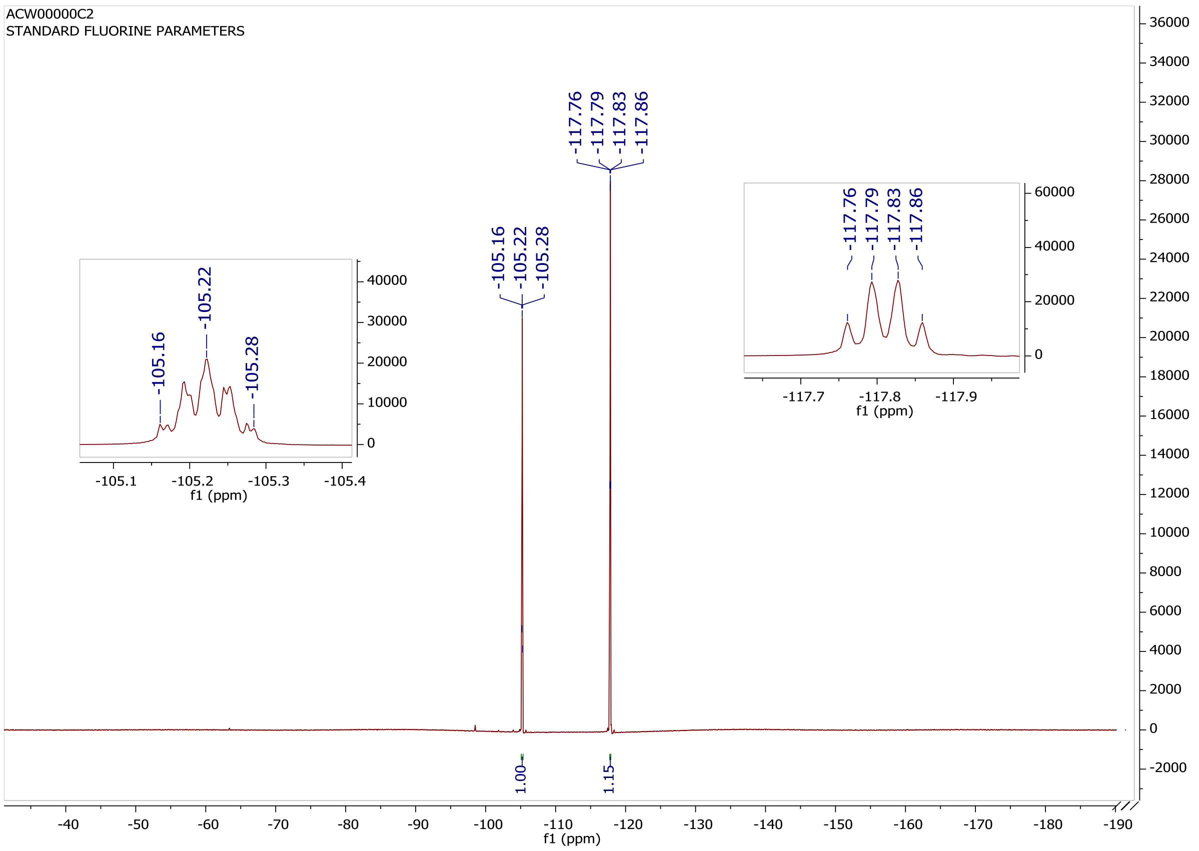

Figure S53. [19]**F NMR spectrum of 2e ($CDCl_3$)**

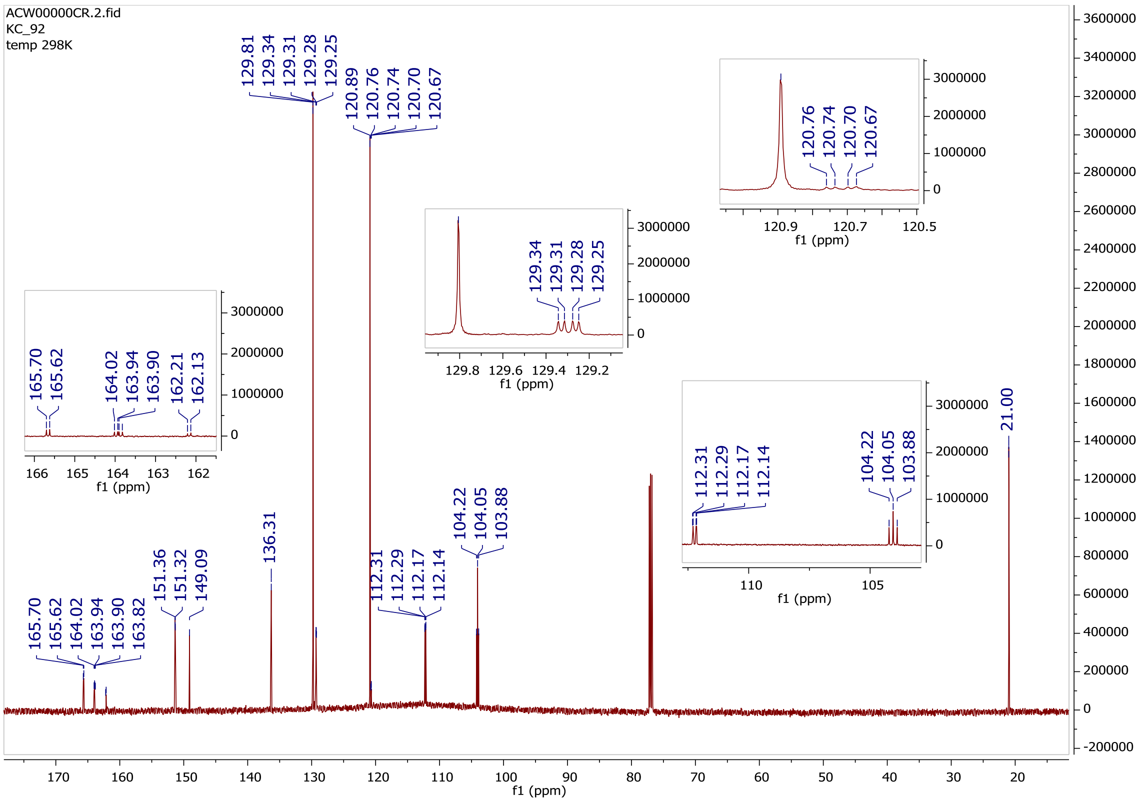

Figure S54. [13]**C NMR spectrum of 2e ($CDCl_3$)**

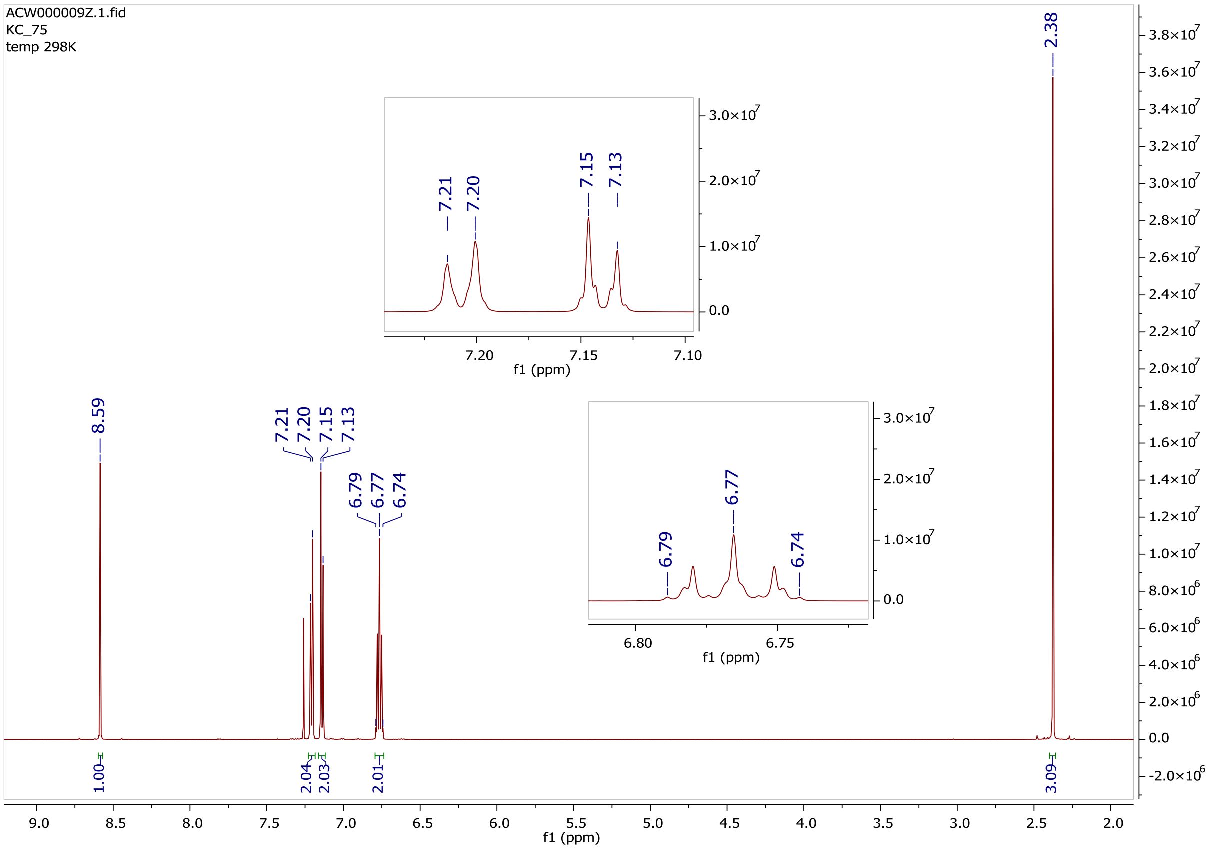

Figure S55. ^{1}H NMR spectrum of 3e ($CDCl_3$)

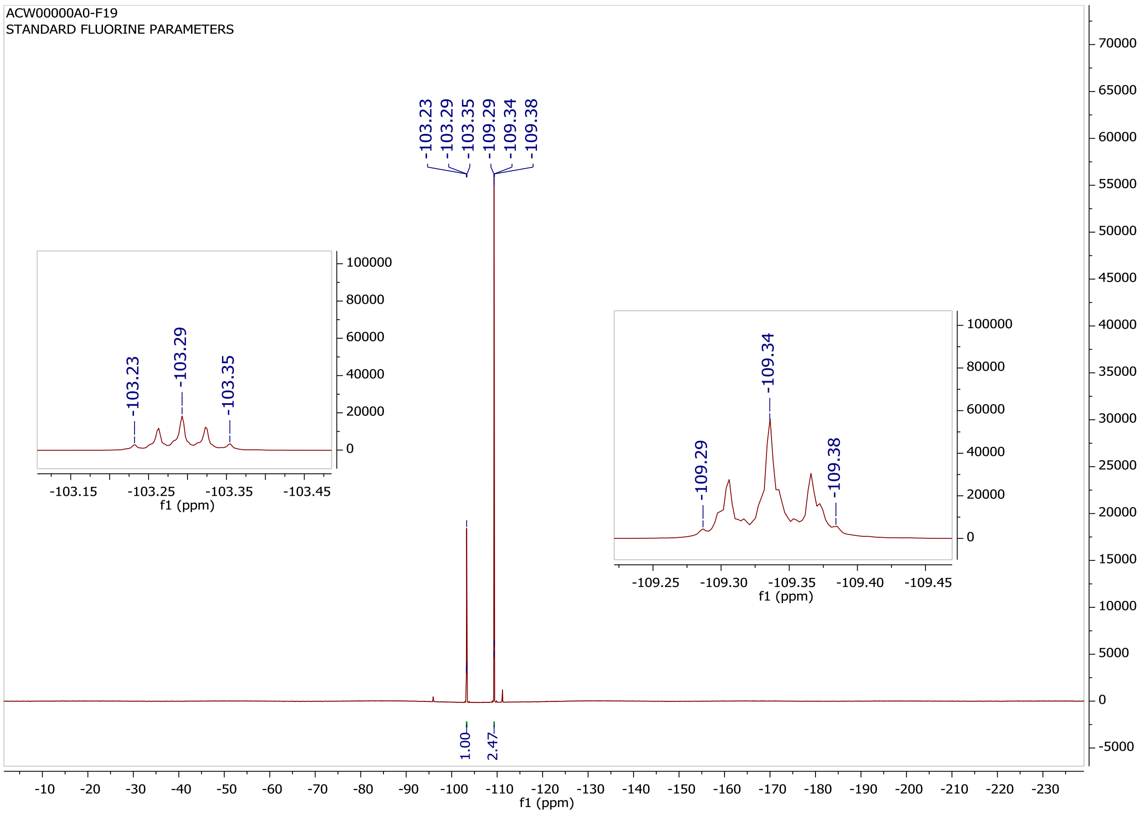

Figure S56. ^{19}F NMR spectrum of 3e ($CDCl_3$)

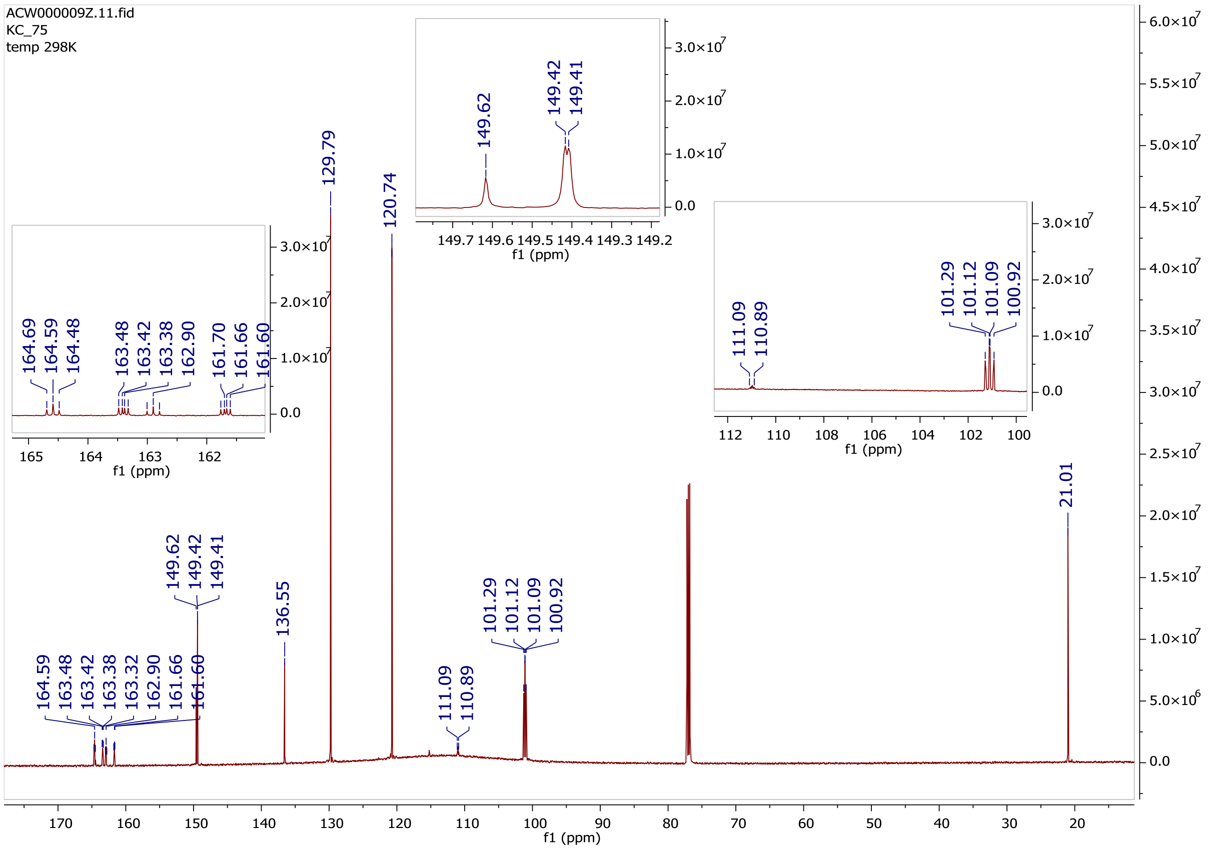

Figure S57. ^{13}C **NMR spectrum of 3e** ($CDCl_3$)

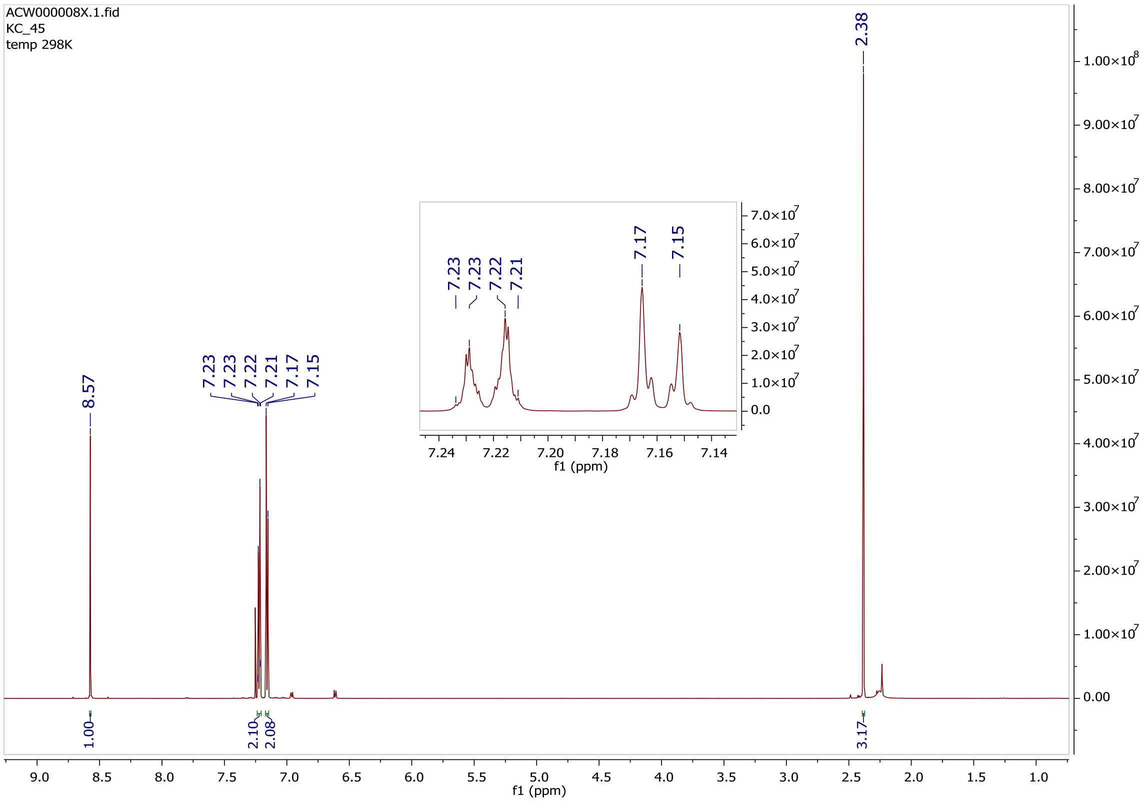

Figure S58. ^{1}H **NMR spectrum of 4e** ($CDCl_3$)

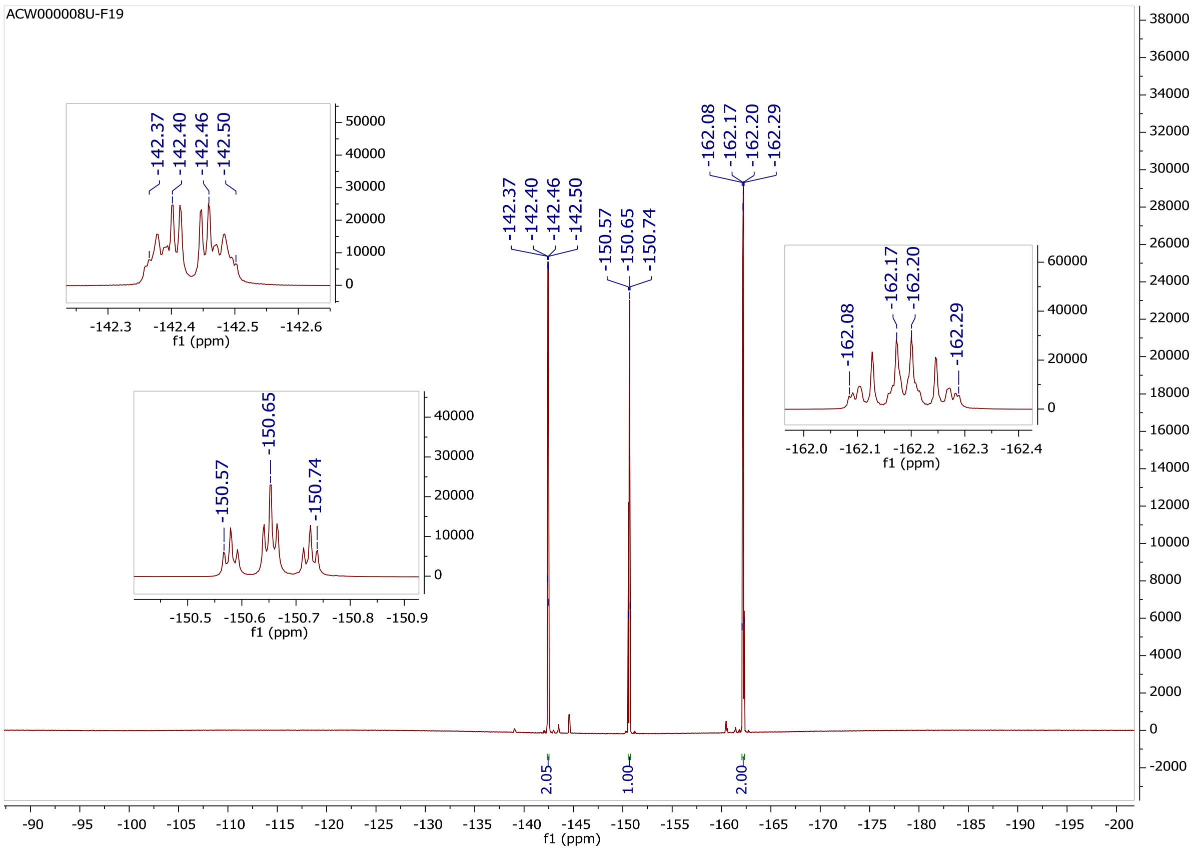

Figure S59. ^{19}F **NMR spectrum of 4e (CDCl$_3$)**

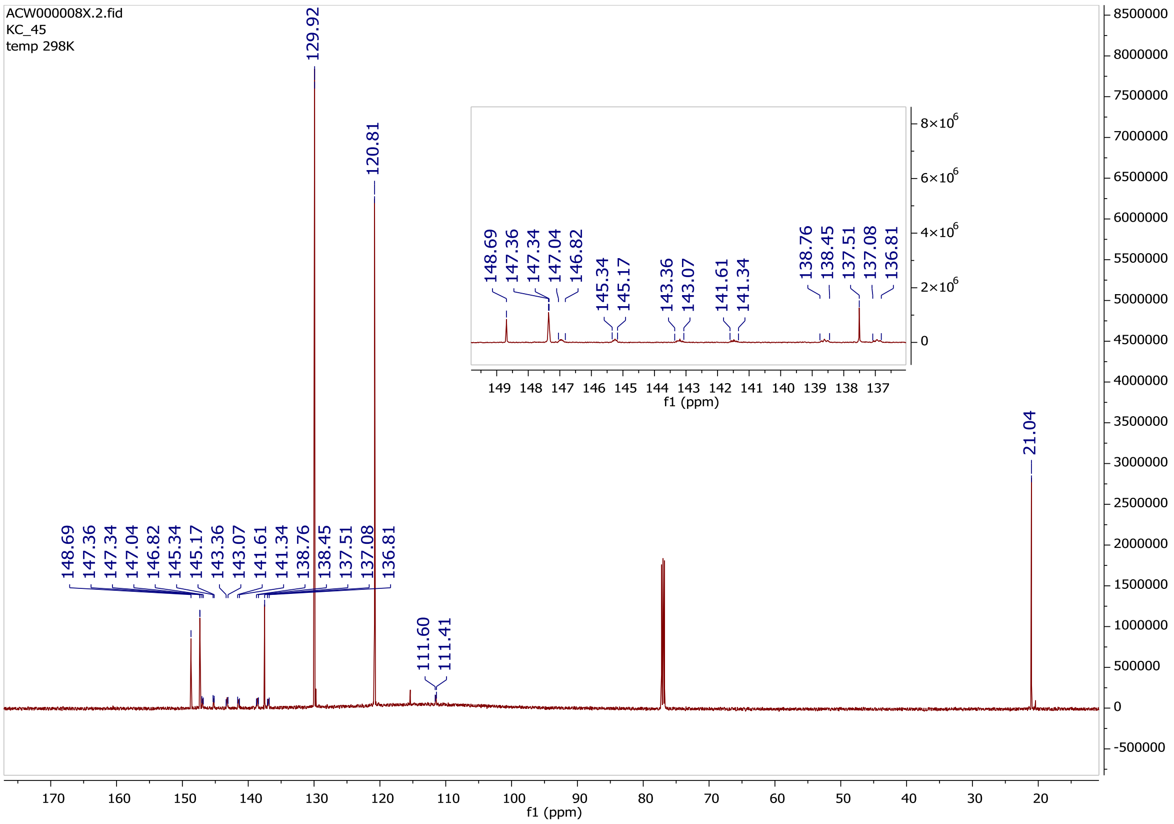

Figure S60. ^{13}C **NMR spectrum of 4e (CDCl$_3$)**

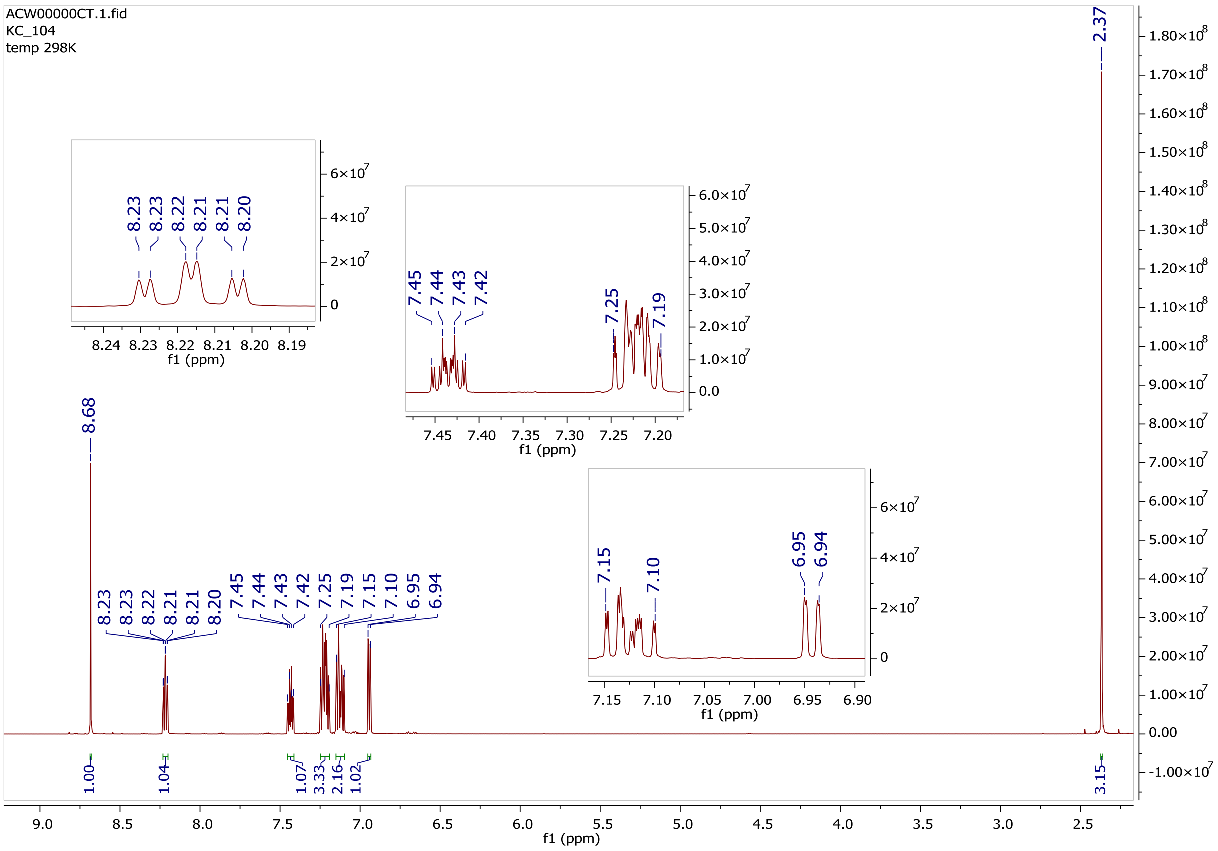

Figure S61. ^{1}**H NMR spectrum of 1f (CDCl$_3$)**

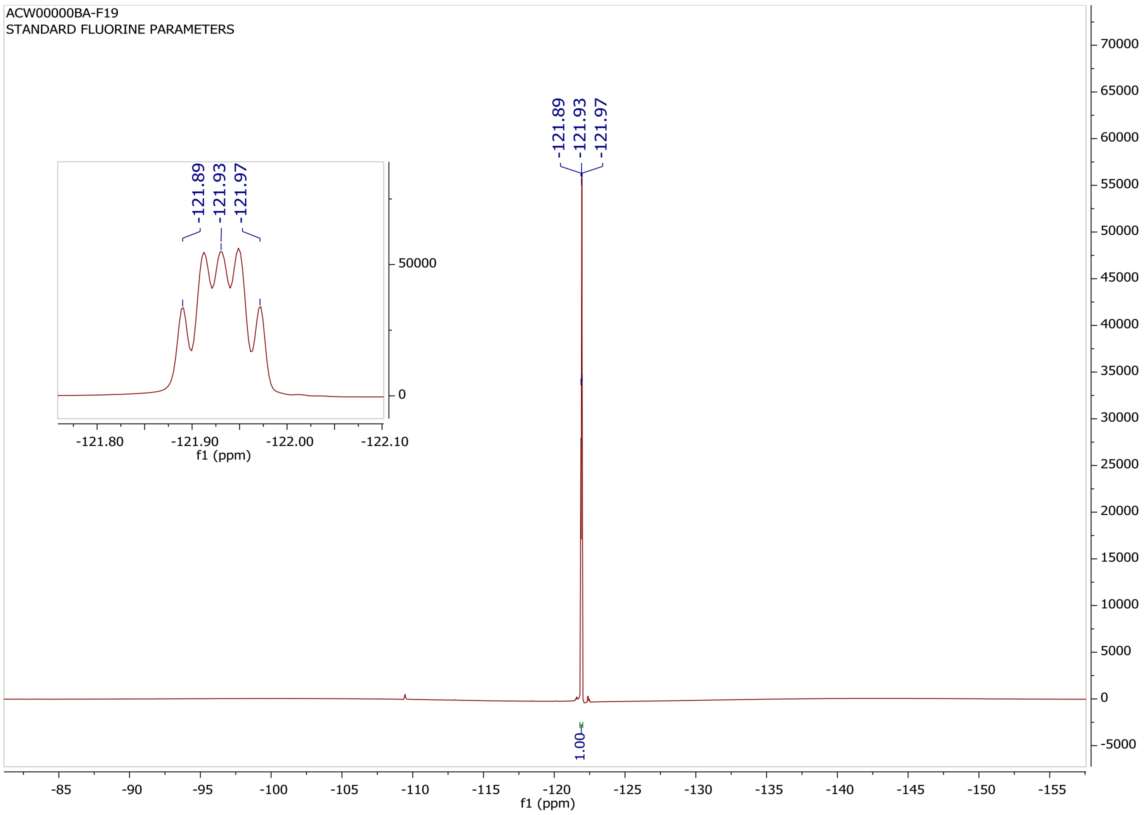

Figure S62. ^{19}**F NMR spectrum of 1f (CDCl$_3$)**

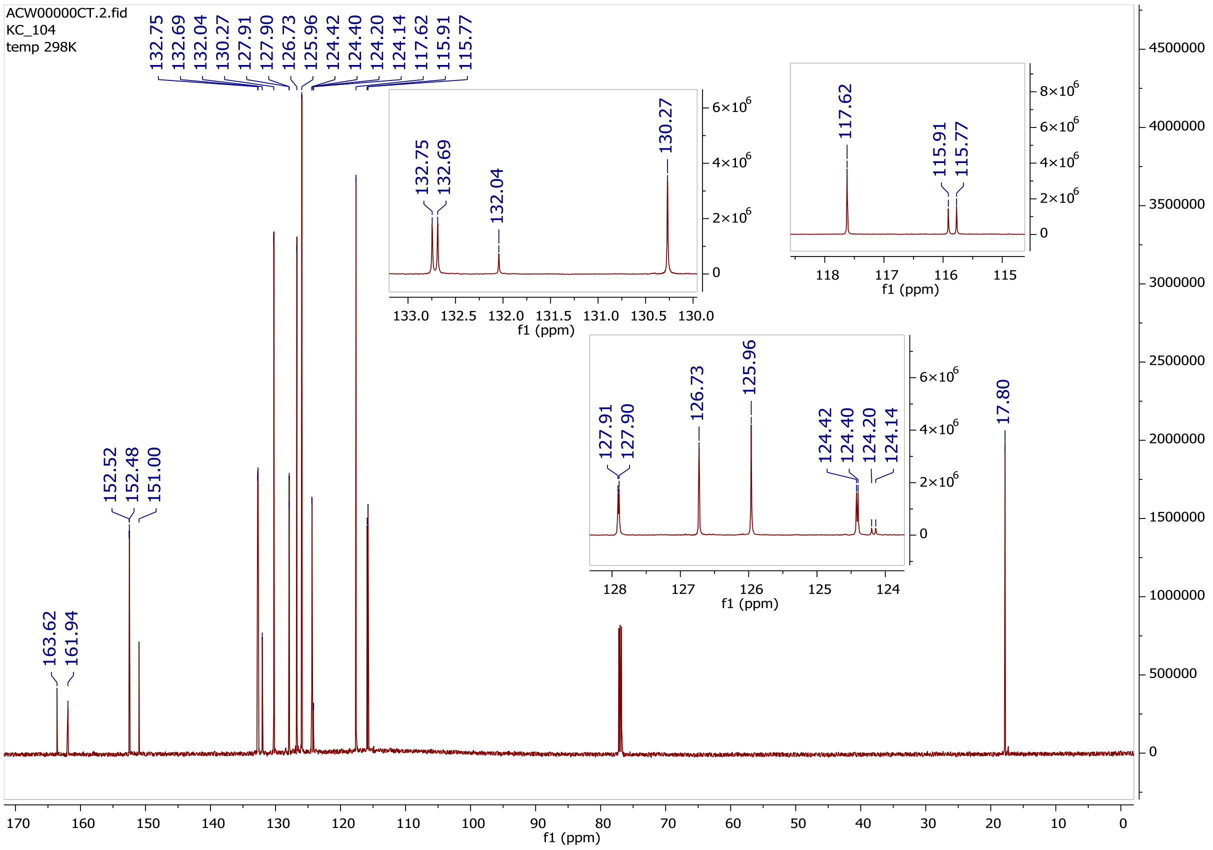

Figure S63. ^{13}C **NMR spectrum of 1f (CDCl$_3$)**

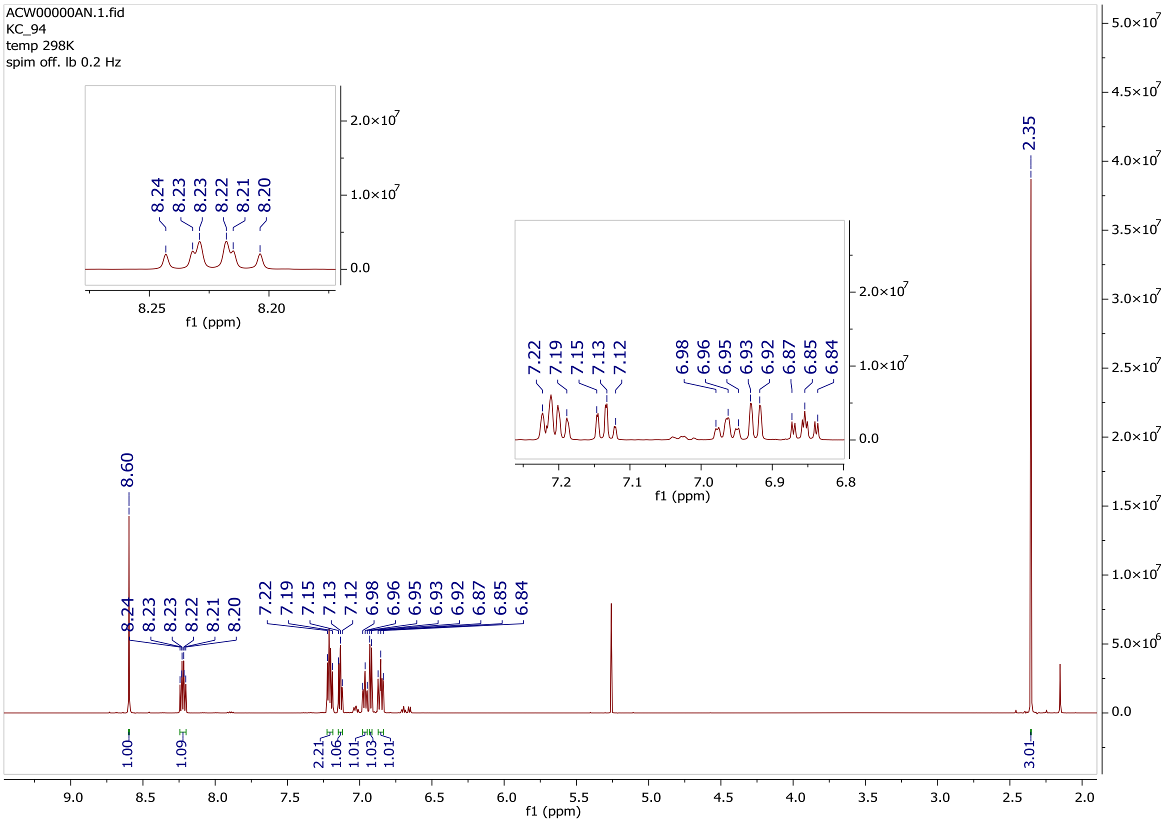

Figure S64. ^{1}H **NMR spectrum of 2f (CDCl$_3$)**

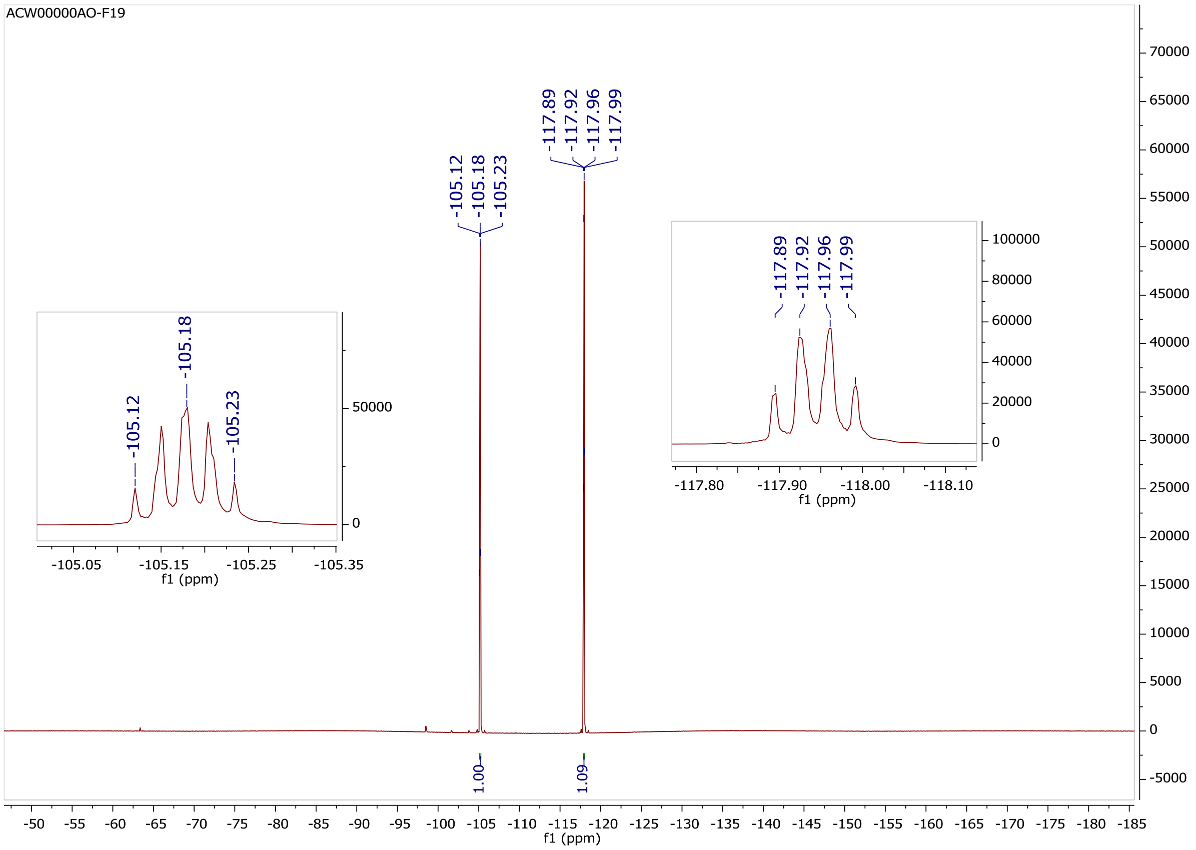

Figure S65. ^{19}**F NMR spectrum of 2f (CDCl$_3$)**

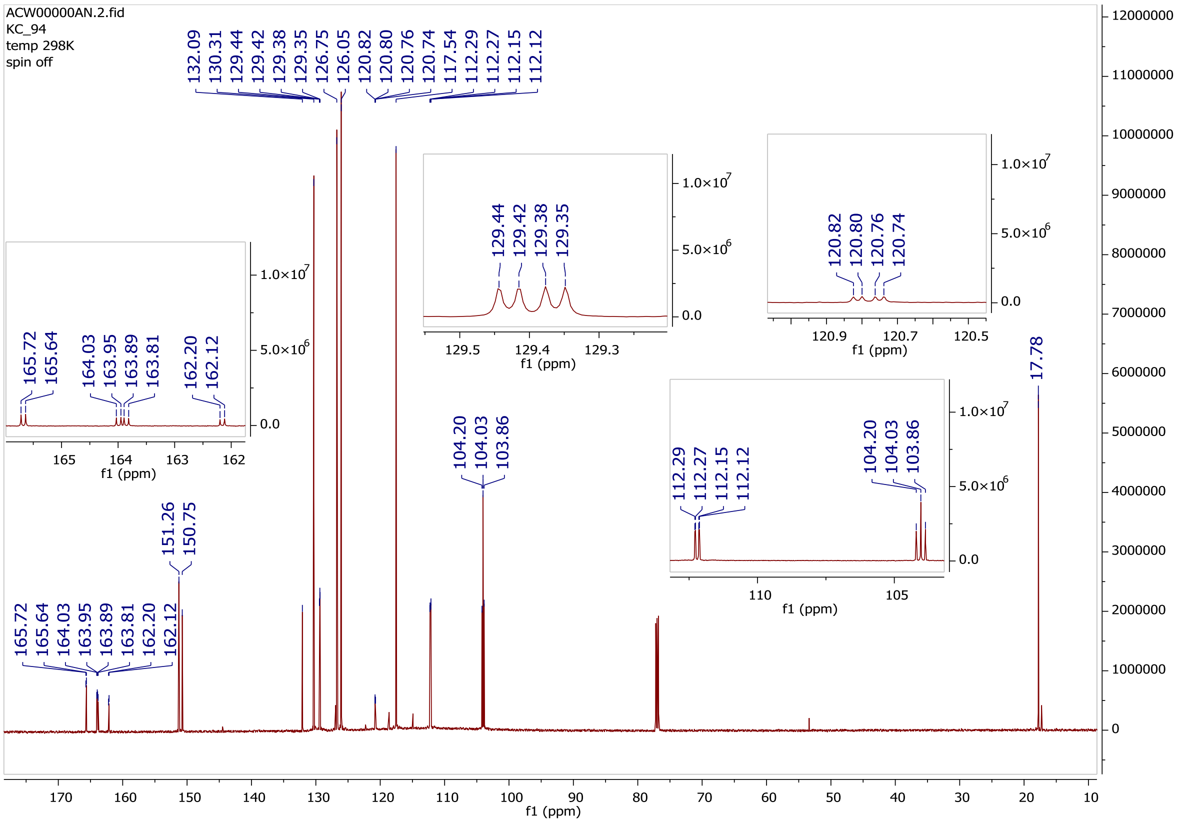

Figure S66. ^{13}**C NMR spectrum of 2f (CDCl$_3$)**

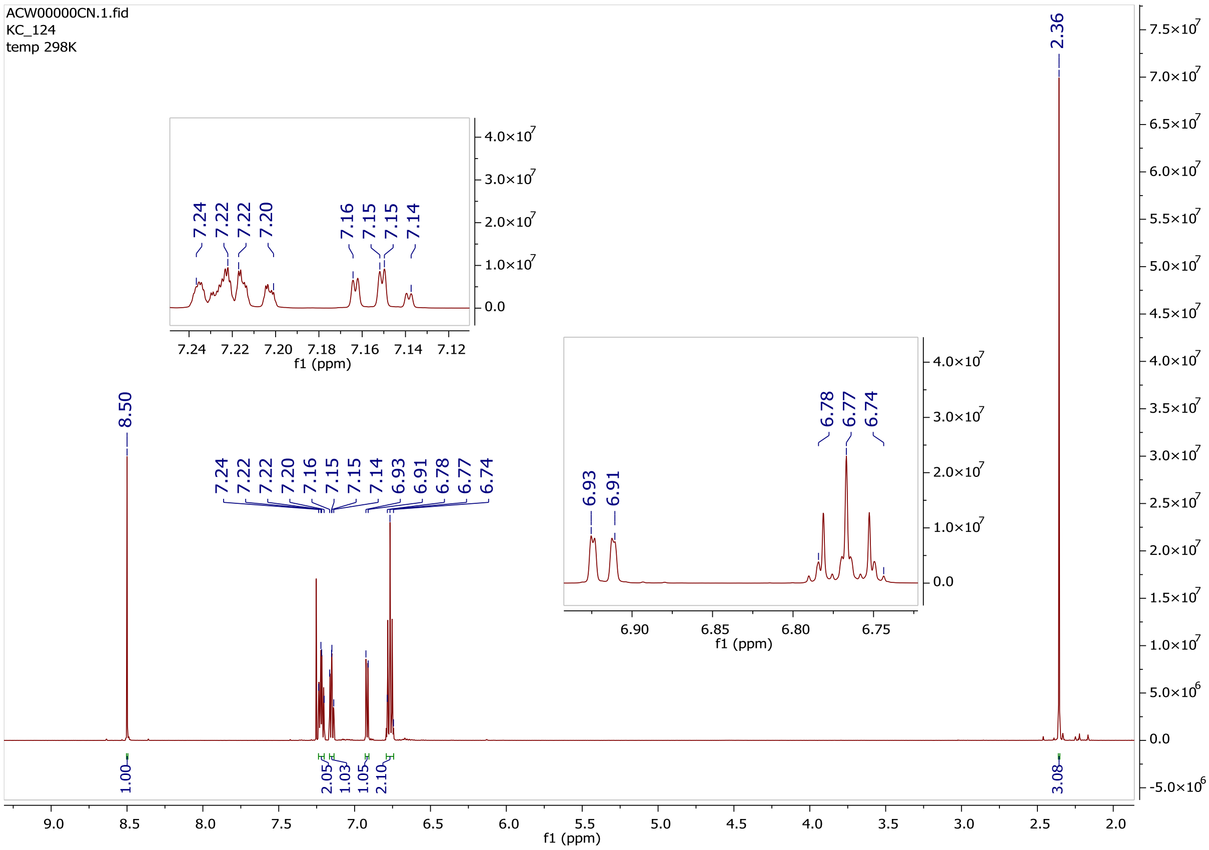

Figure S67. ^{1}H NMR spectrum of 3f ($CDCl_3$)

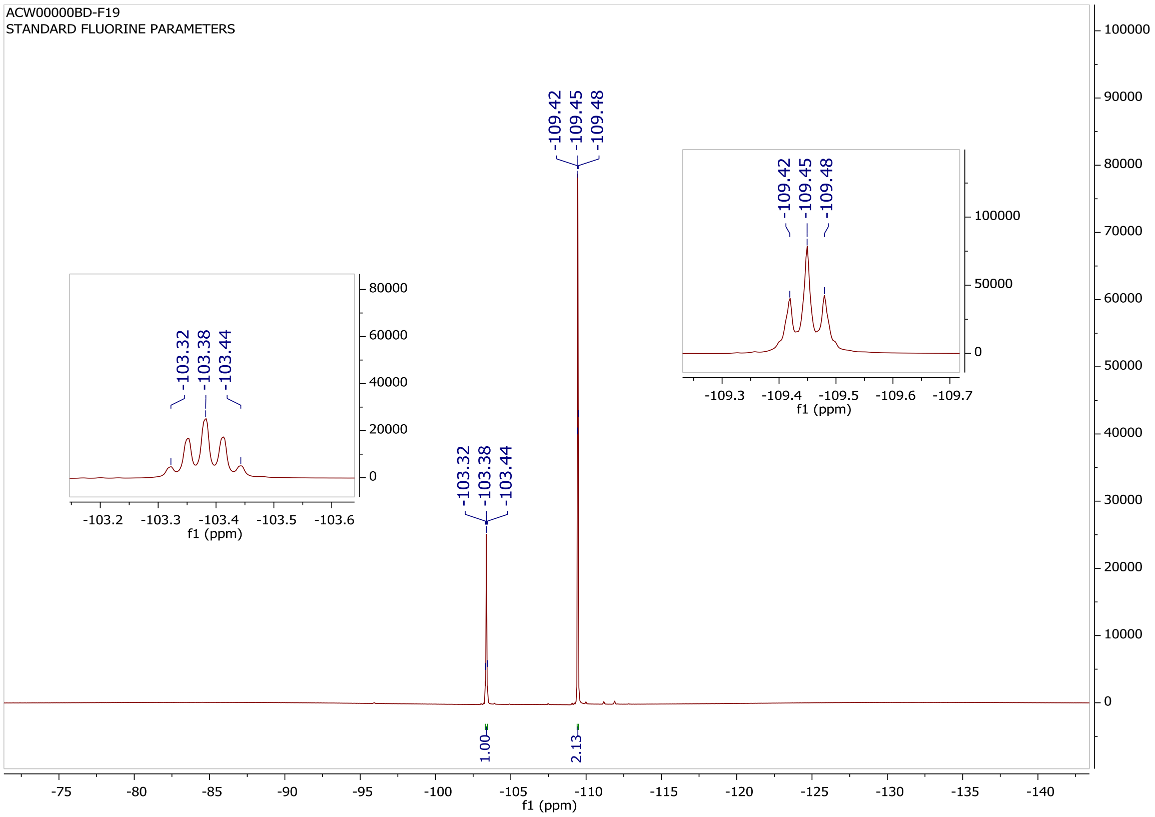

Figure S68. ^{19}F NMR spectrum of 3f ($CDCl_3$)

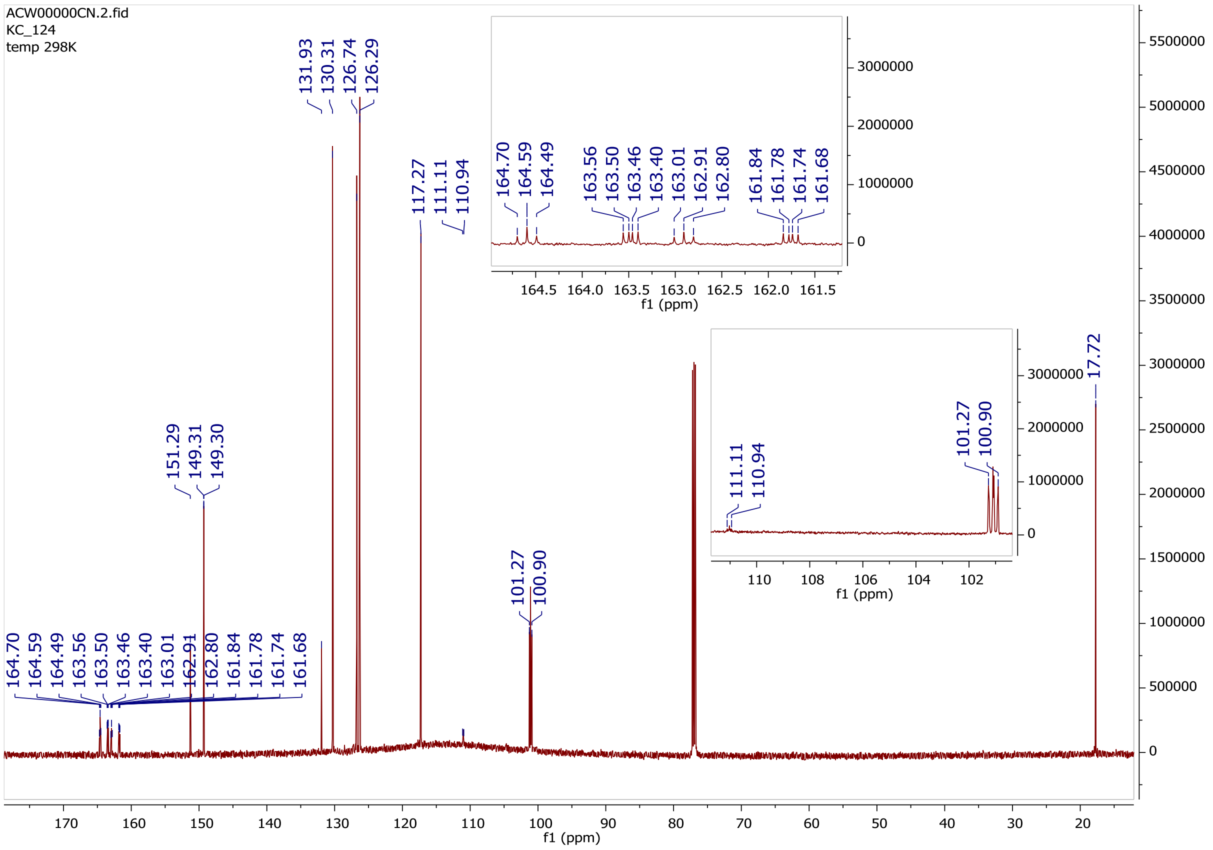

Figure S69. ^{13}**C NMR spectrum of 3f (CDCl$_3$)**

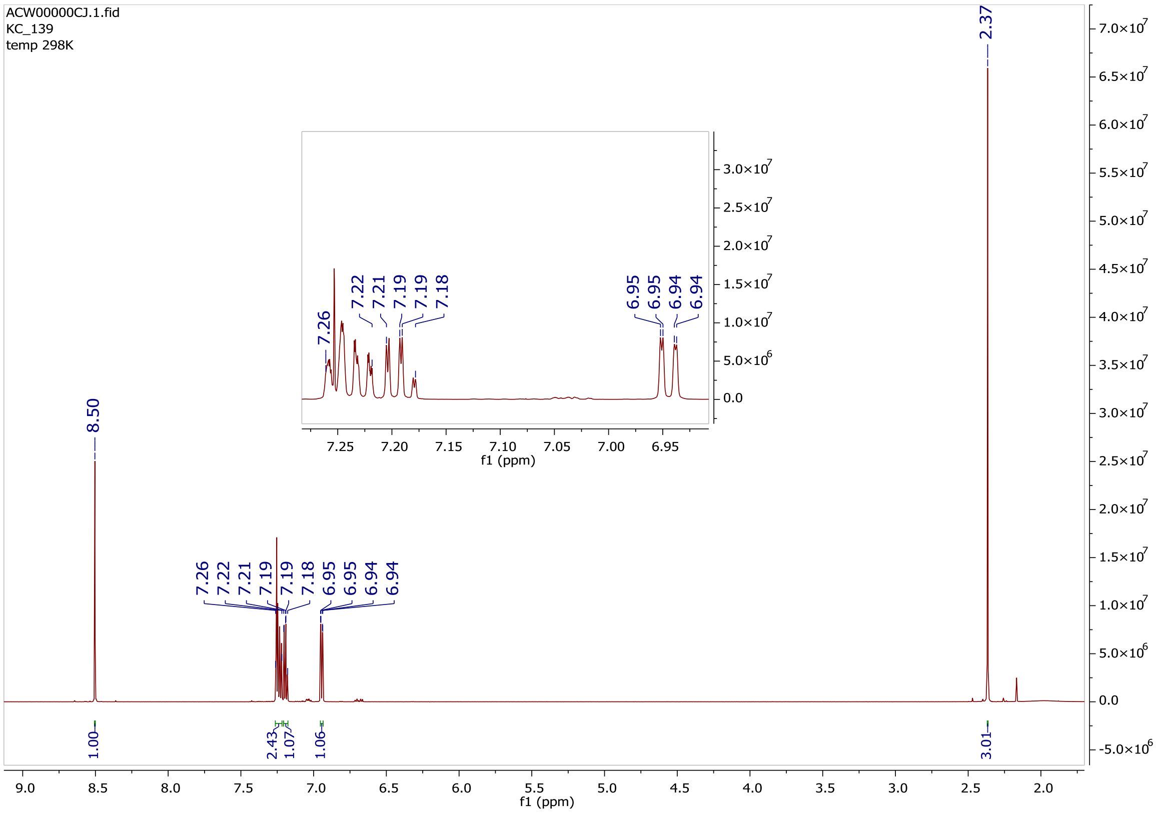

Figure S70. ^{1}**H NMR spectrum of 4f (CDCl$_3$)**

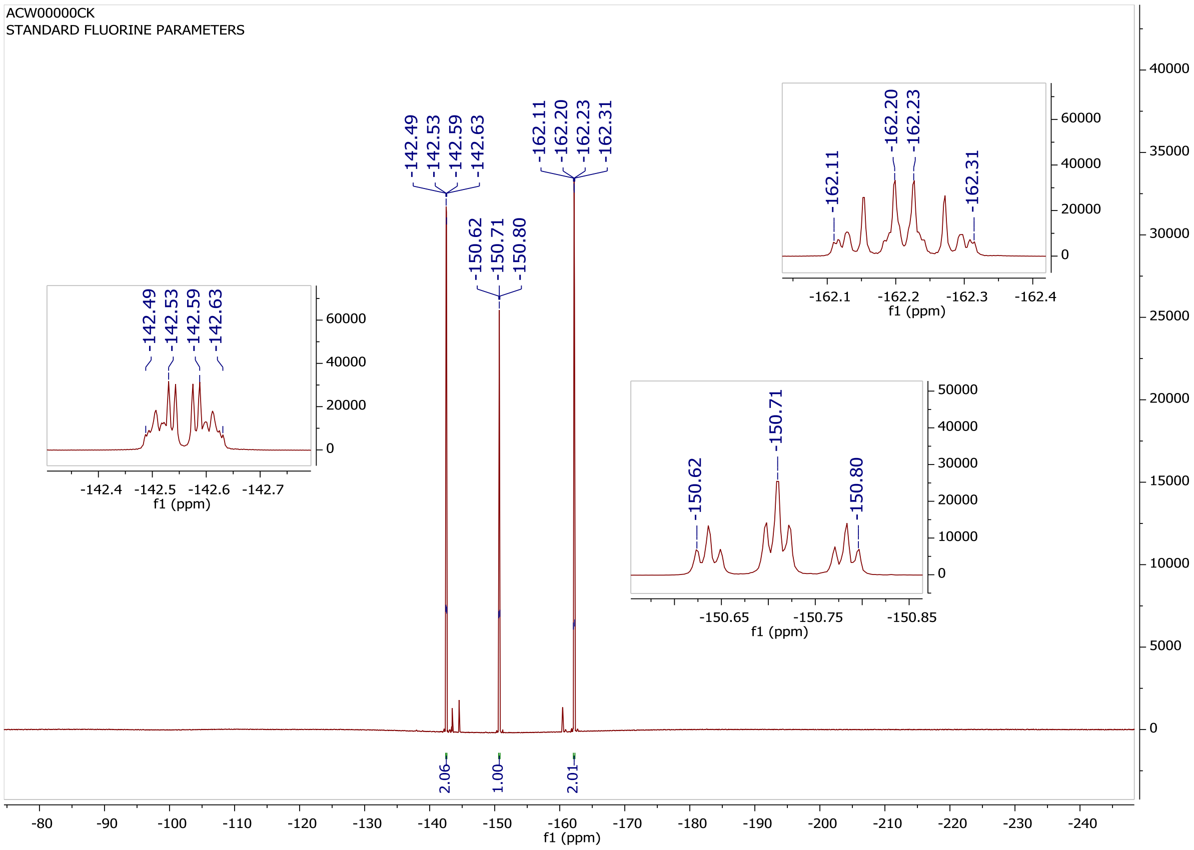

Figure S71. ^{19}**F NMR spectrum of 4f (CDCl$_3$)**

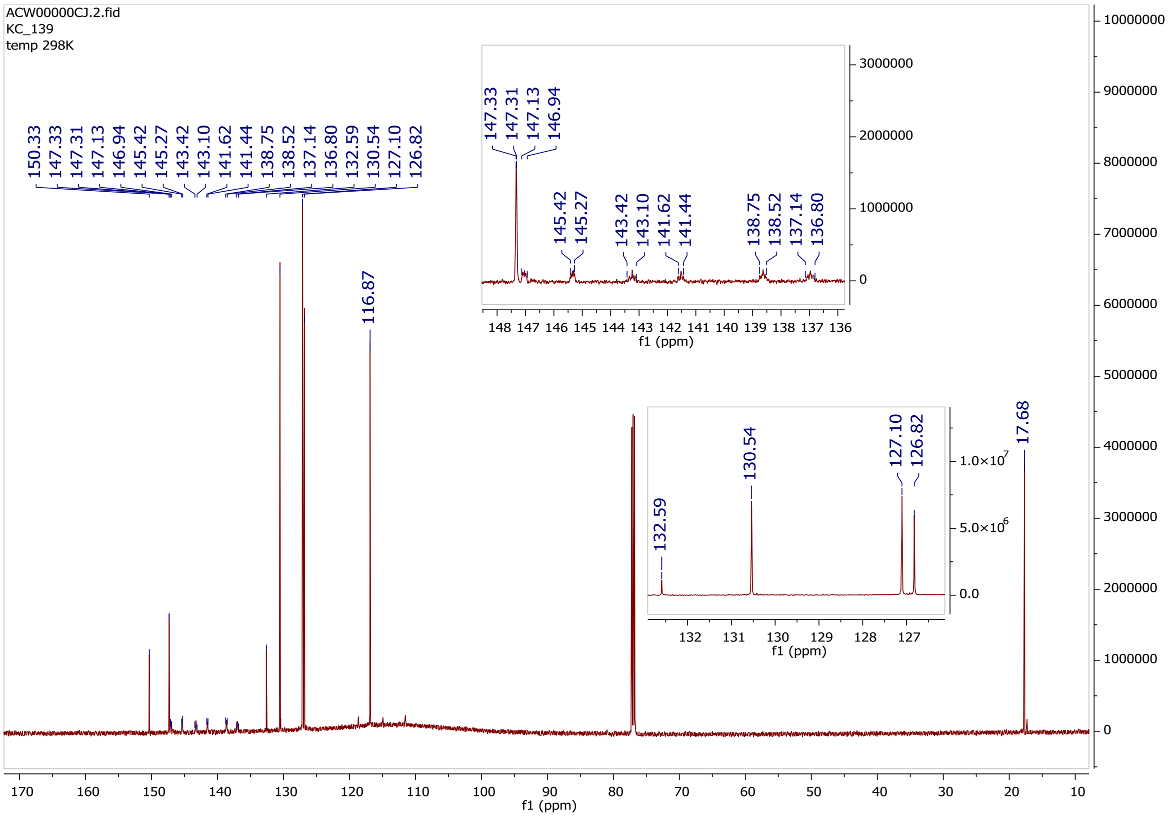

Figure S72. ^{13}**C NMR spectrum of 4f (CDCl$_3$)**

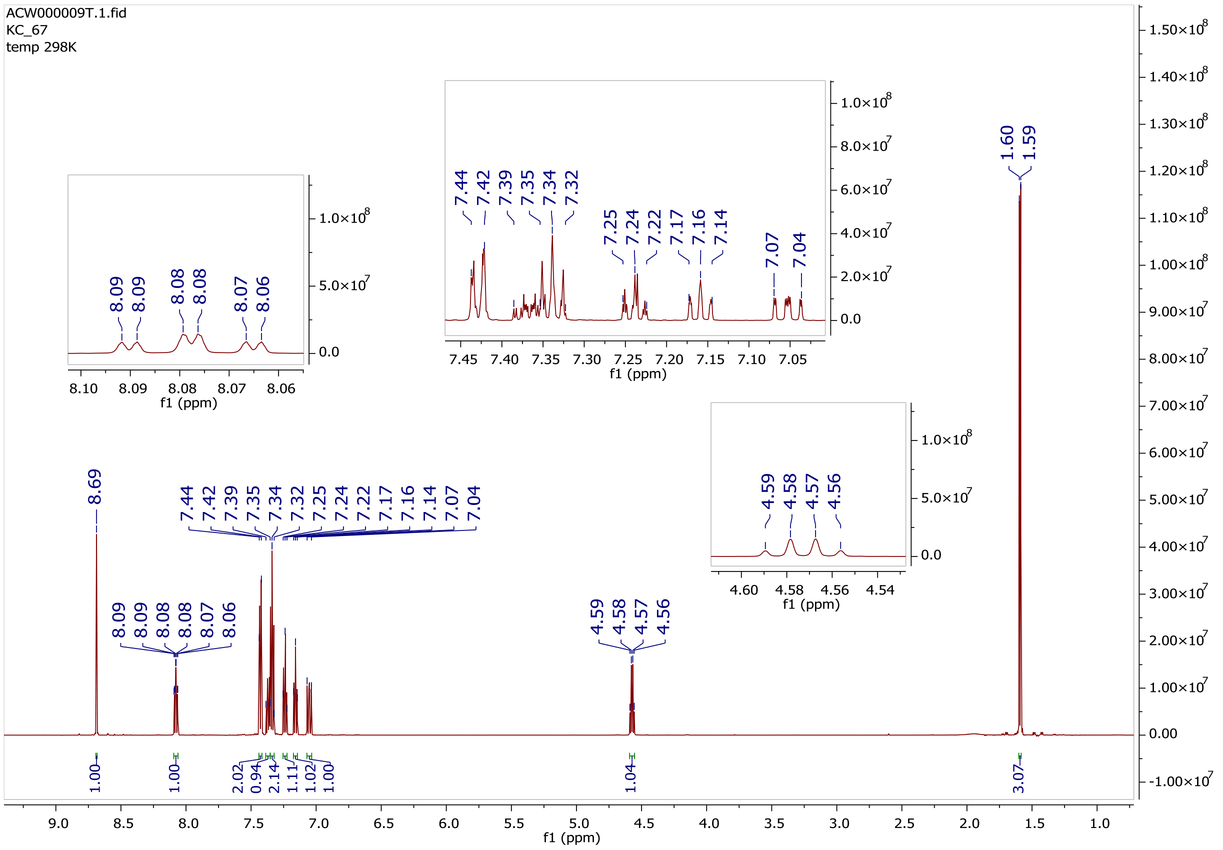

Figure S73. ^{1}**H NMR spectrum of 1g (CDCl$_3$)**

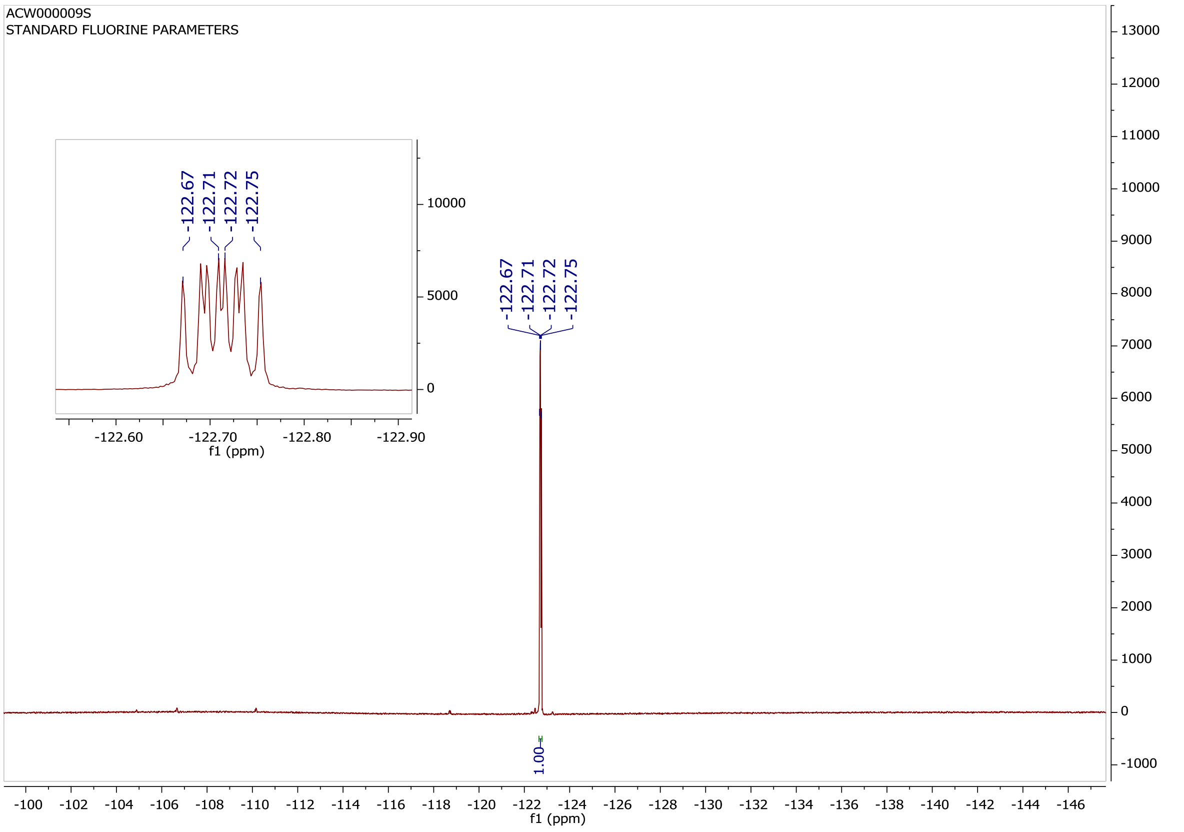

Figure S74. ^{19}**F NMR spectrum of 1g (CDCl$_3$)**

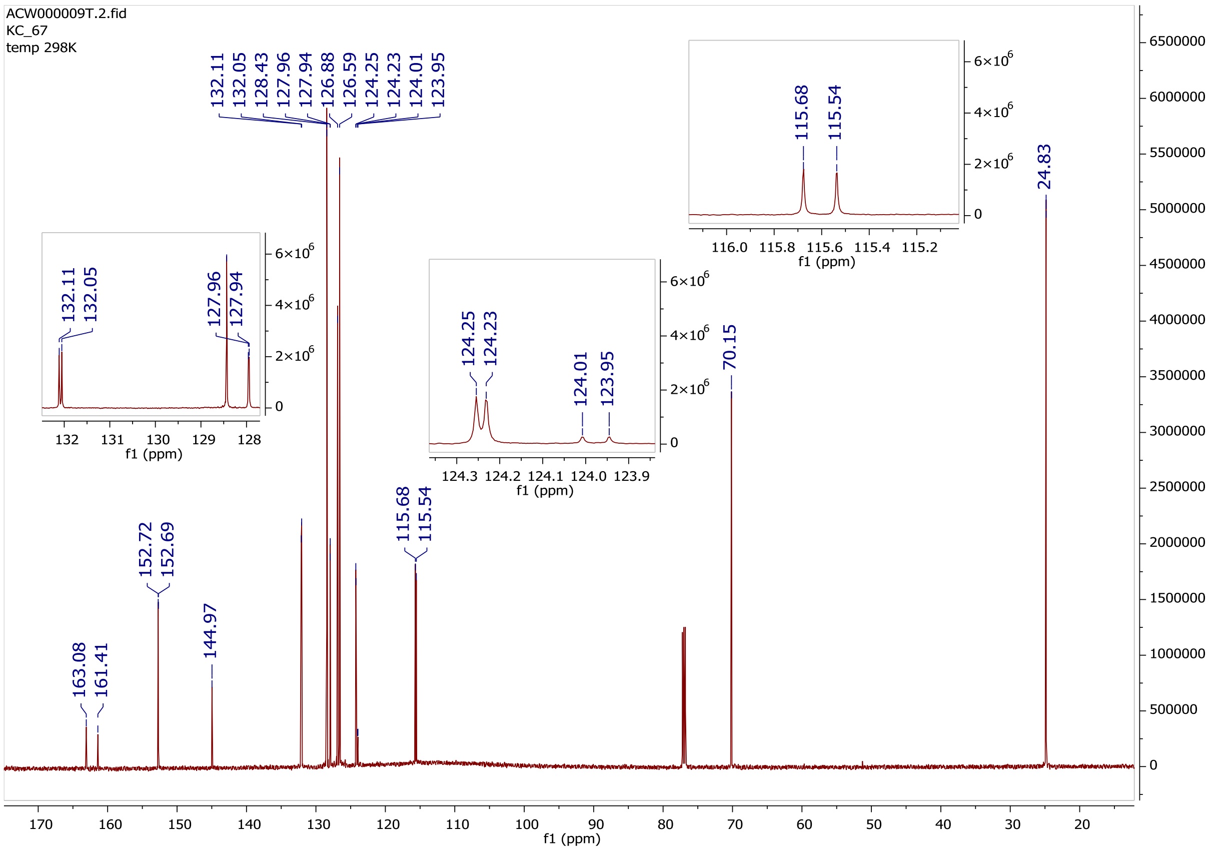

Figure S75. ^{13}C NMR spectrum of 1g ($CDCl_3$)

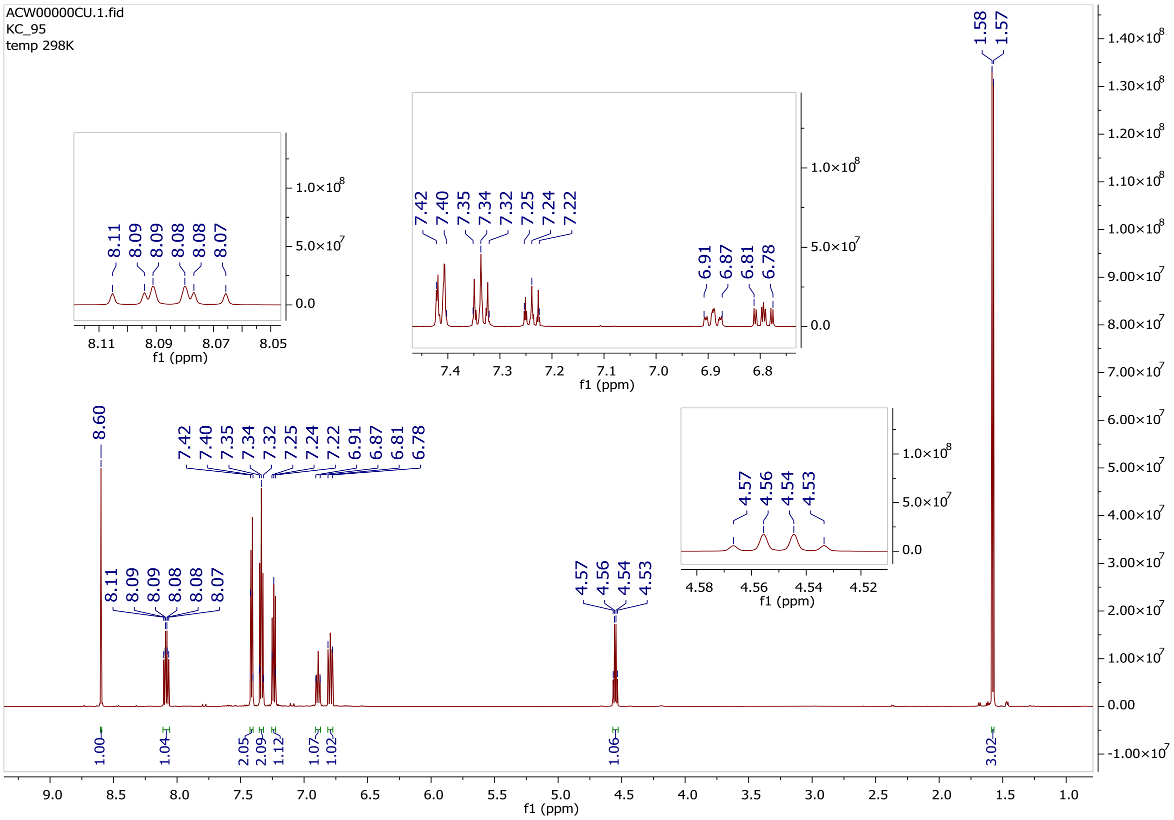

Figure S76. ^{1}H NMR spectrum of 2g ($CDCl_3$)

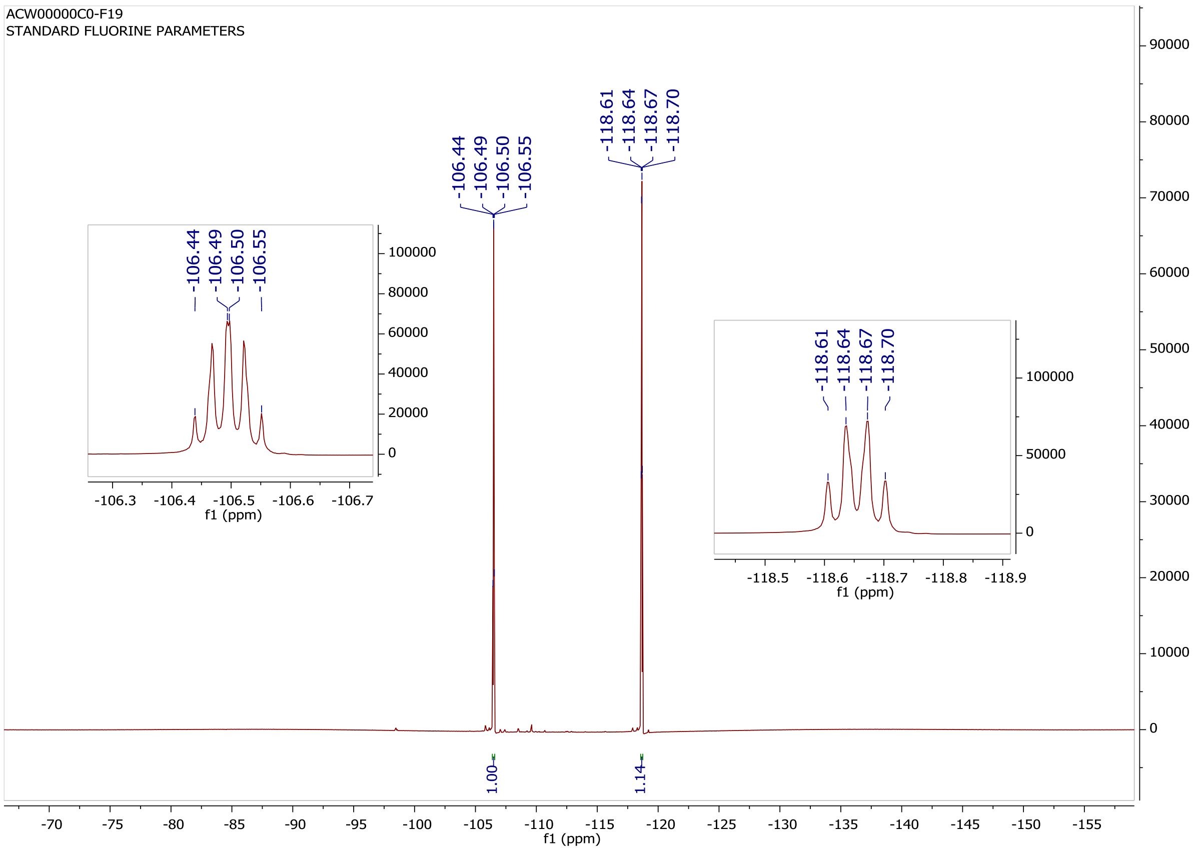

Figure S77. ^{19}F NMR spectrum of 2g ($CDCl_3$)

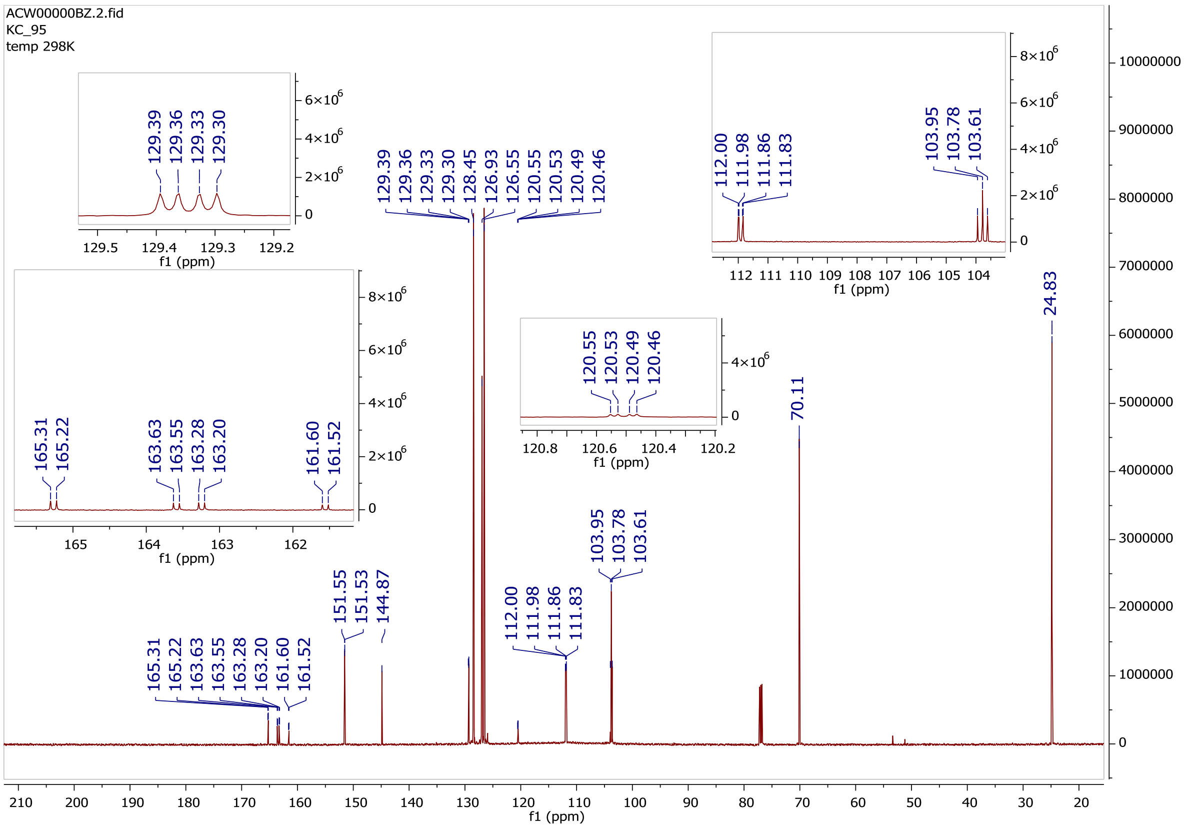

Figure S78. ^{13}C NMR spectrum of 2g ($CDCl_3$)

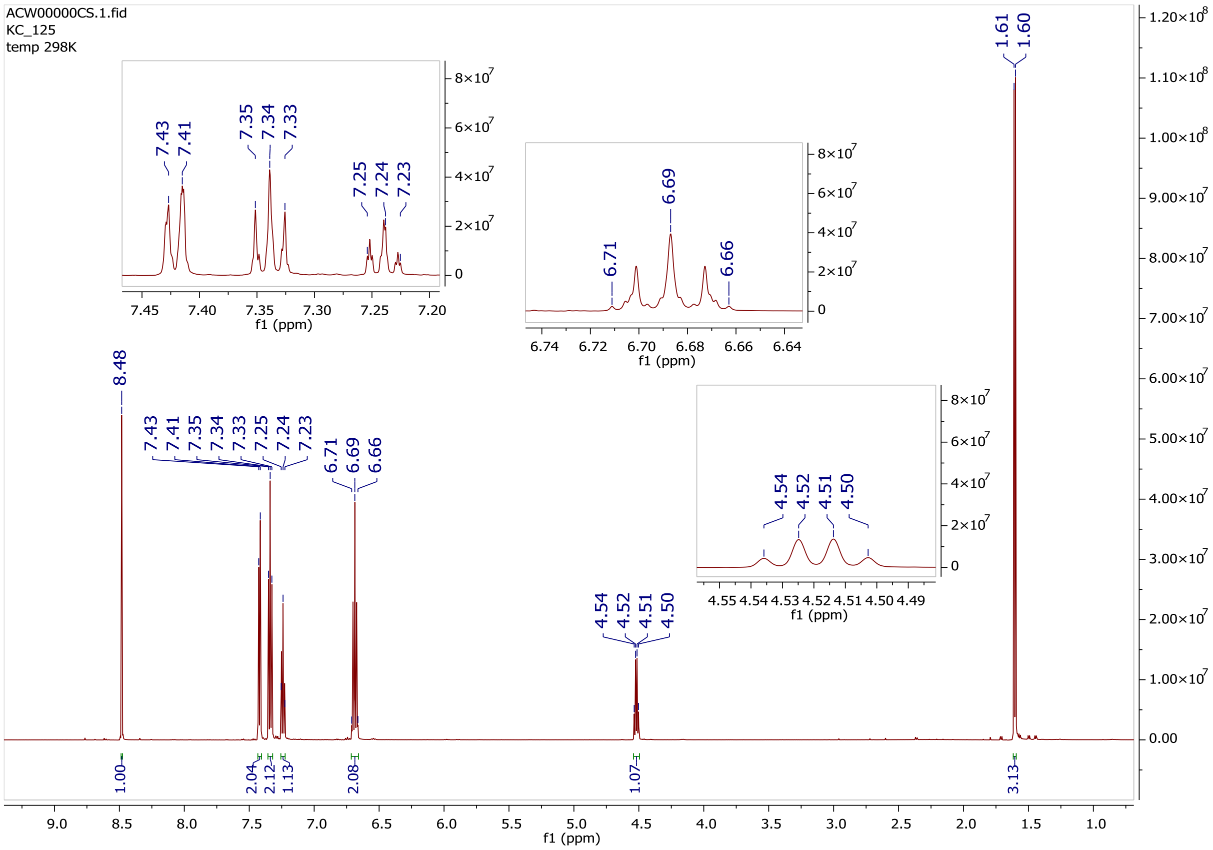

Figure S79. ^{1}H NMR spectrum of 3g ($CDCl_3$)

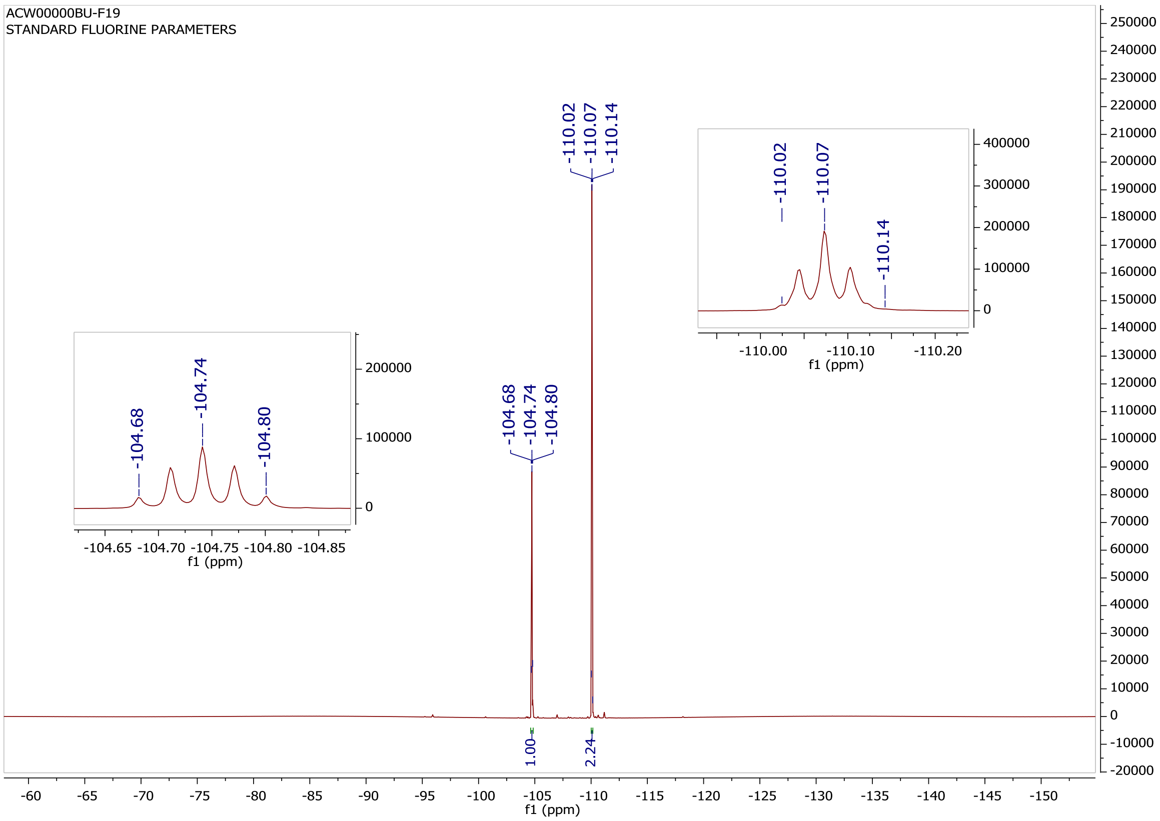

Figure S80. ^{19}F NMR spectrum of 3g ($CDCl_3$)

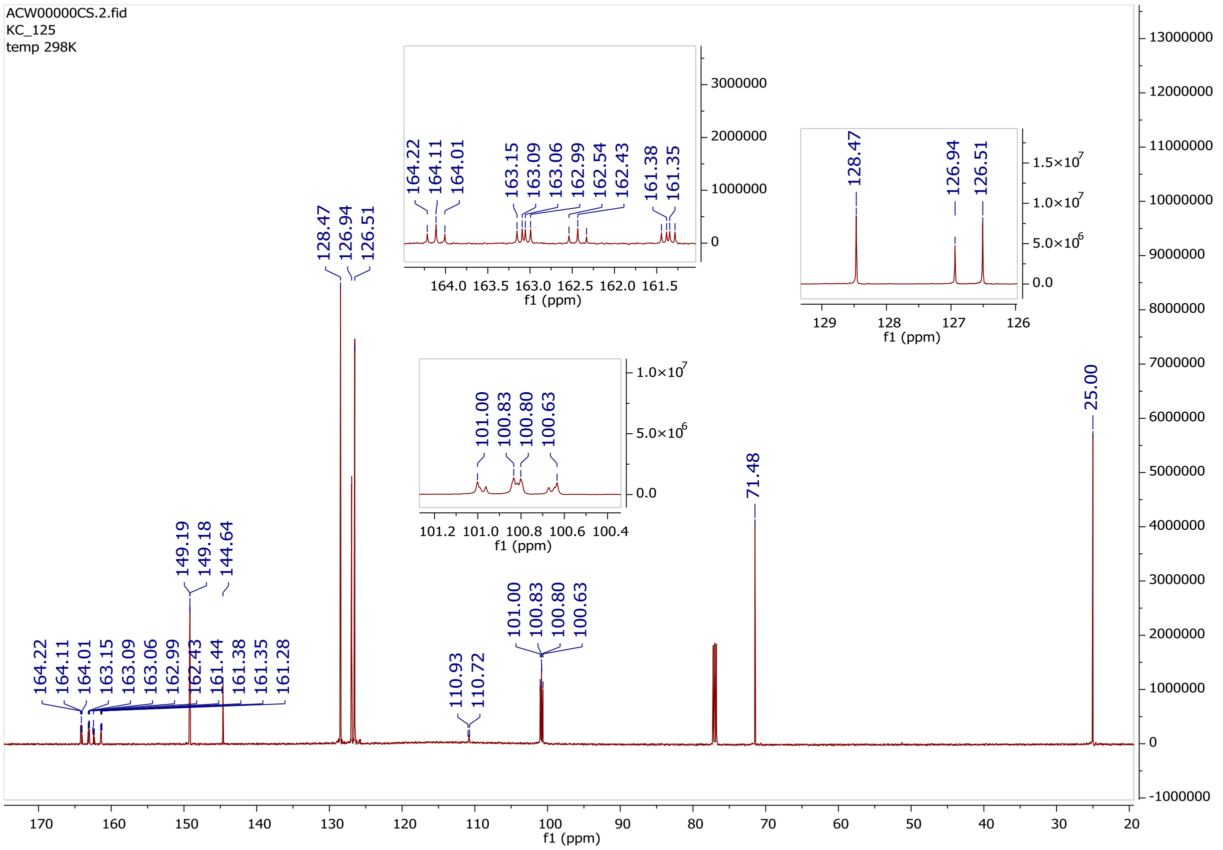

Figure S81. ^{13}**C NMR spectrum of 3g (CDCl$_3$)**

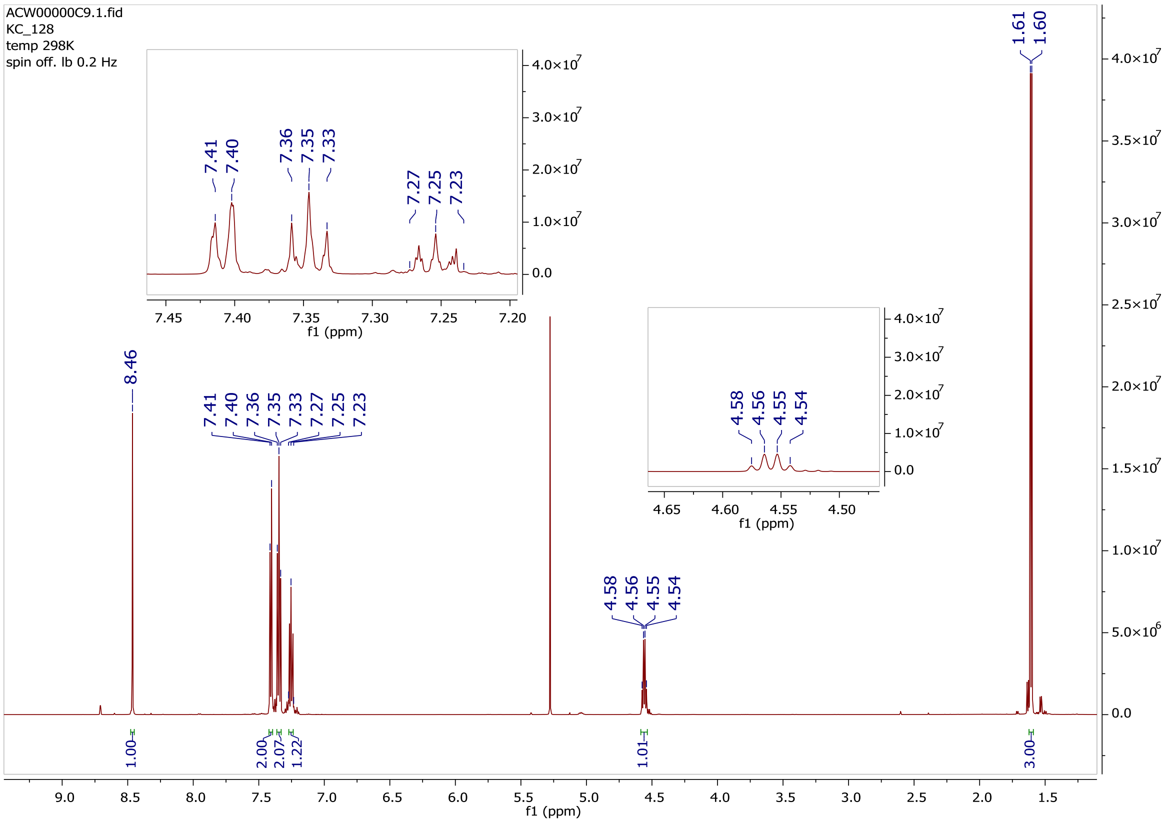

Figure S82. ^{1}**H NMR spectrum of 4g (CDCl$_3$)**

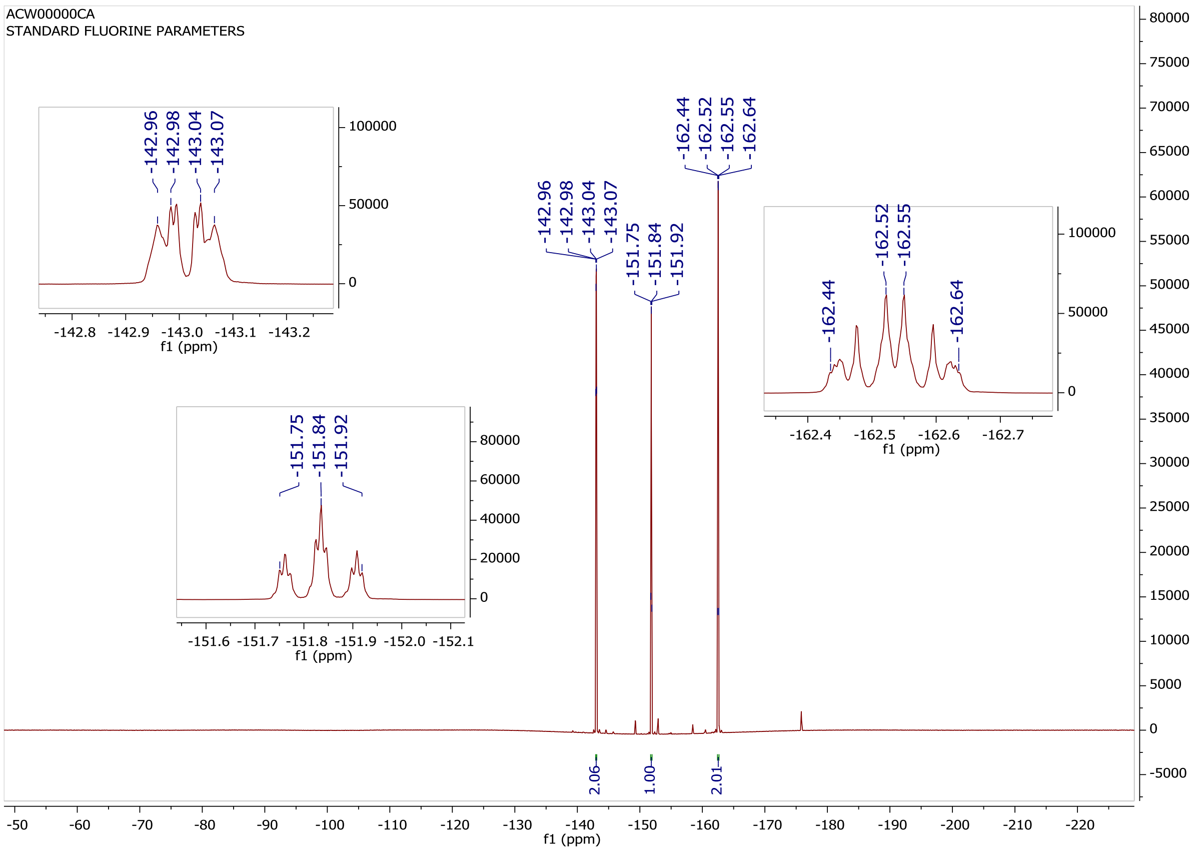

Figure S83. ^{19}F **NMR spectrum of 4g ($CDCl_3$)**

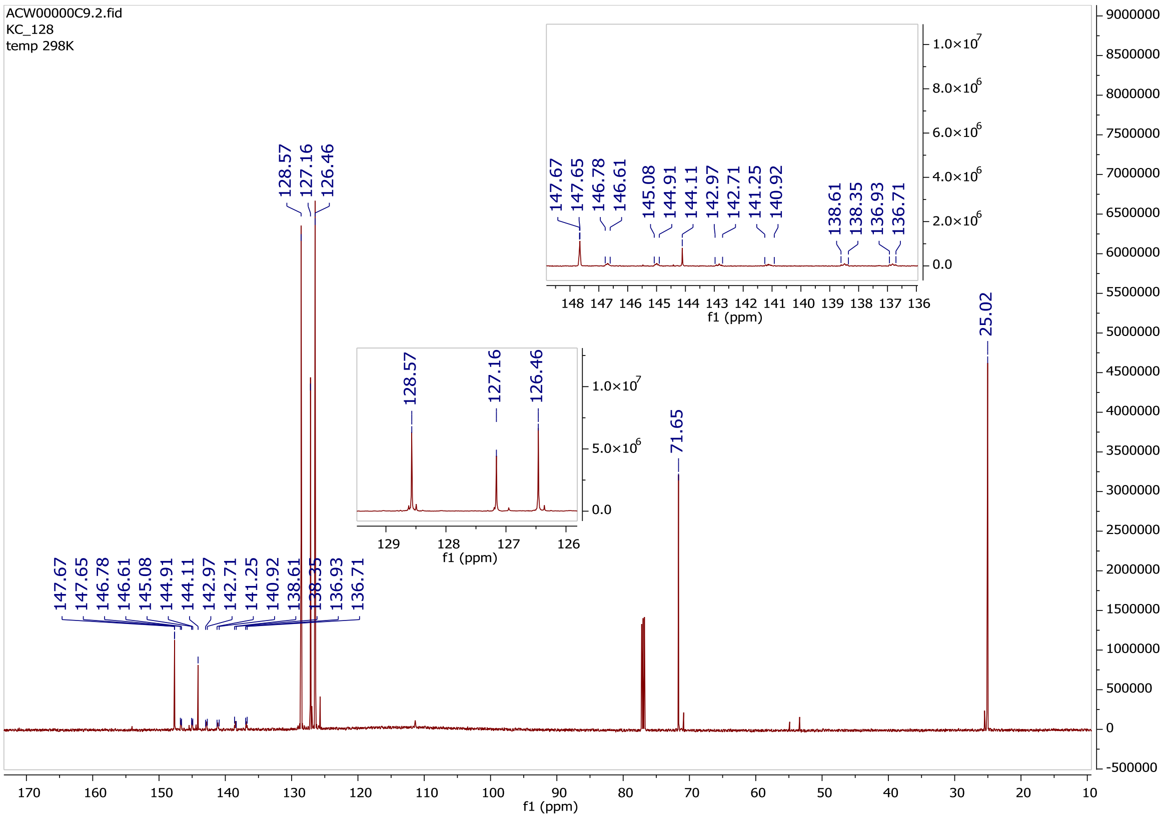

Figure S84. ^{13}C **NMR spectrum of 4g ($CDCl_3$)**

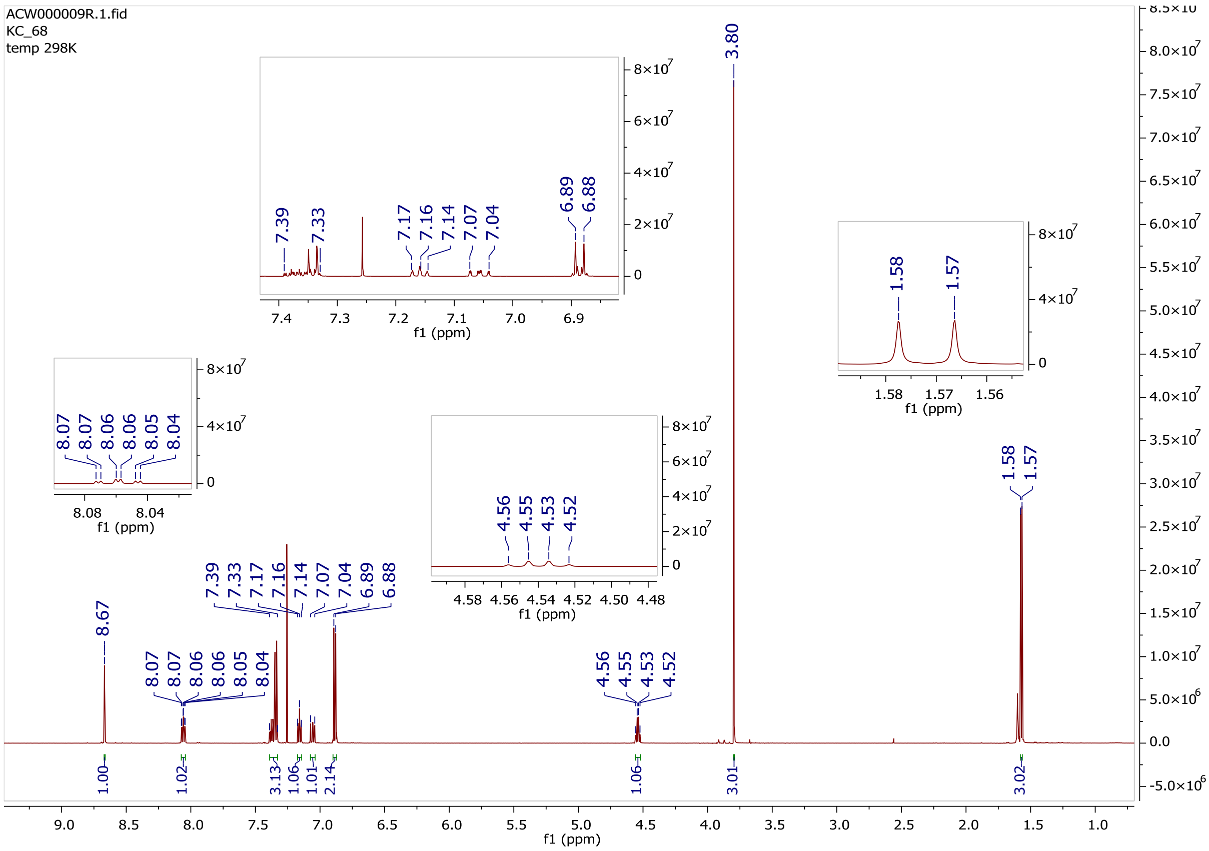

Figure S85. ^{1}H NMR spectrum of 1h ($CDCl_3$)

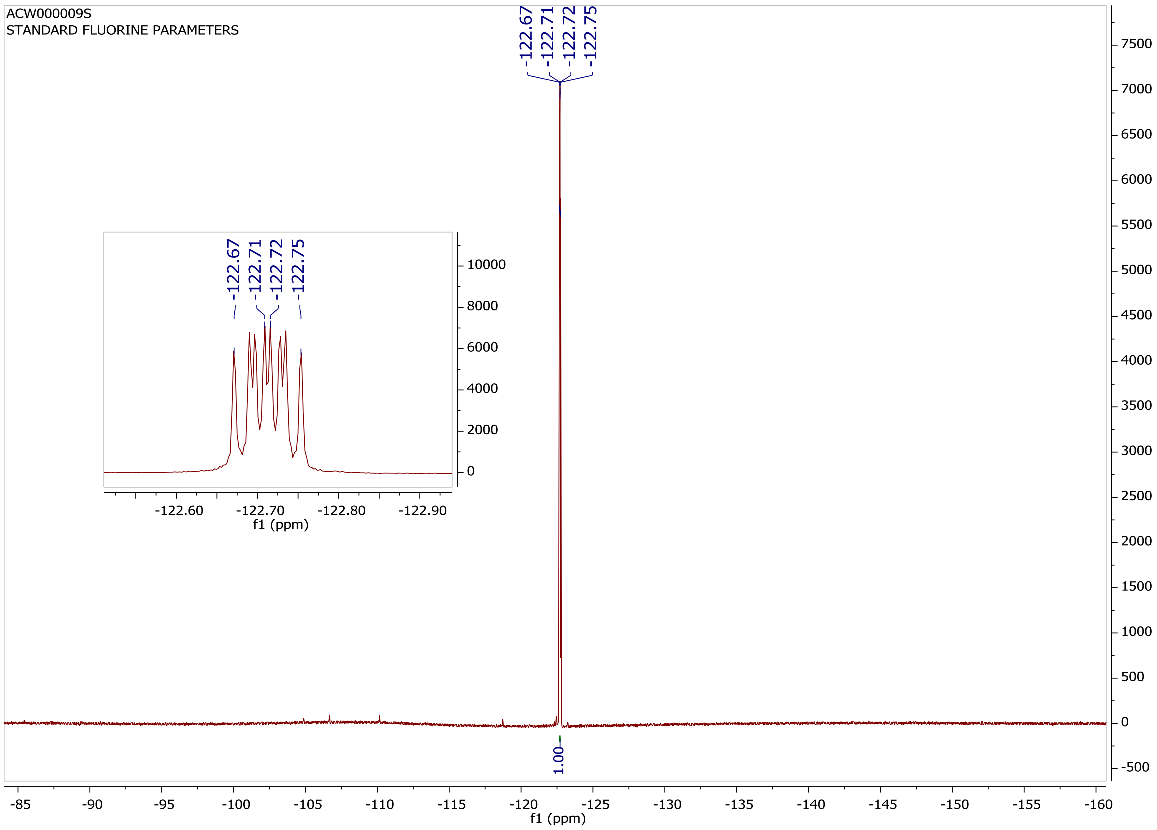

Figure S86. ^{19}F NMR spectrum of 1h ($CDCl_3$)

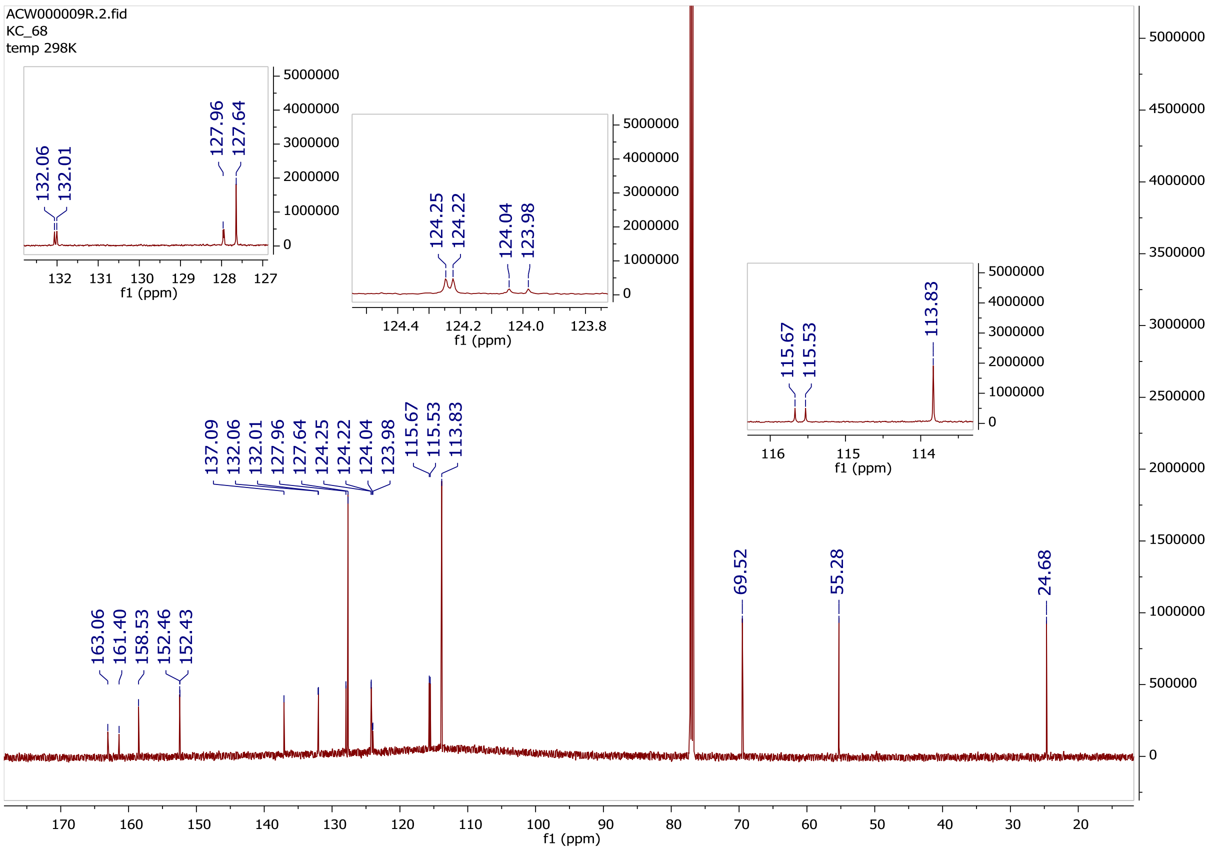

Figure S87. ^{13}C **NMR spectrum of 1h (CDCl$_3$)**

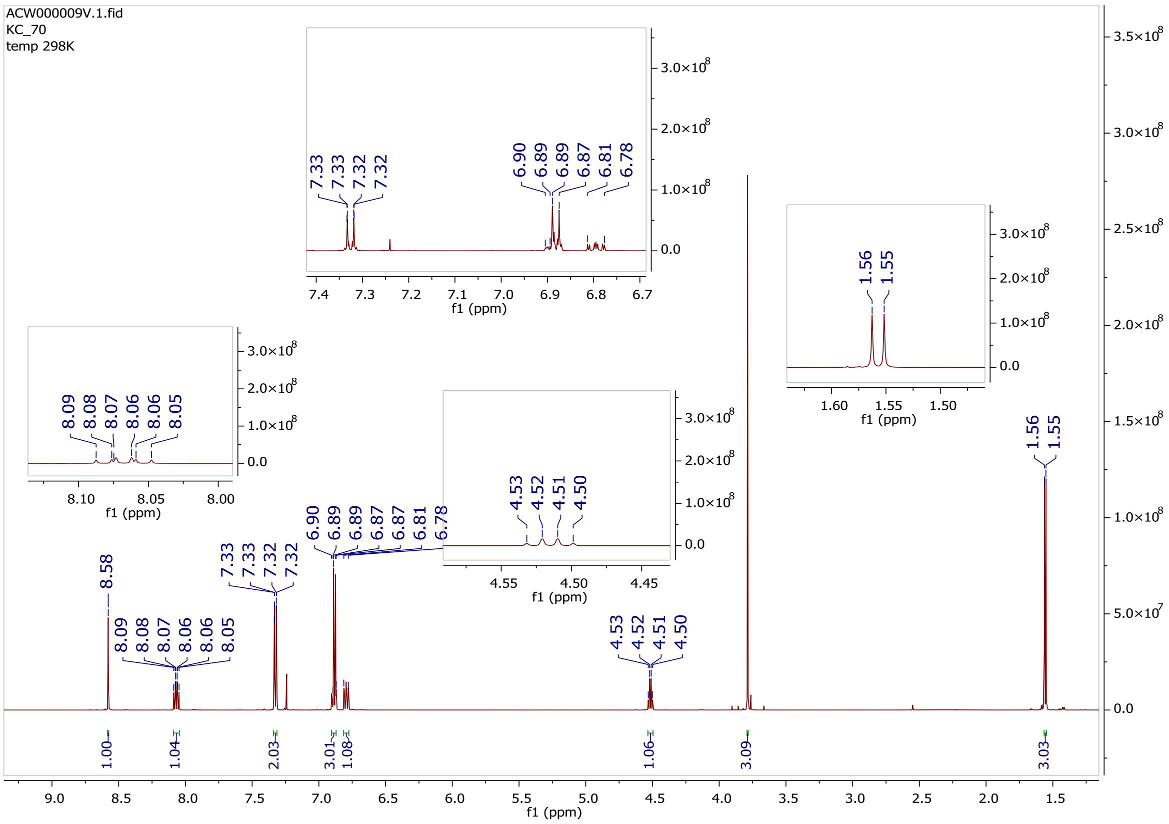

Figure S88. ^{1}H **NMR spectrum of 2h (CDCl$_3$)**

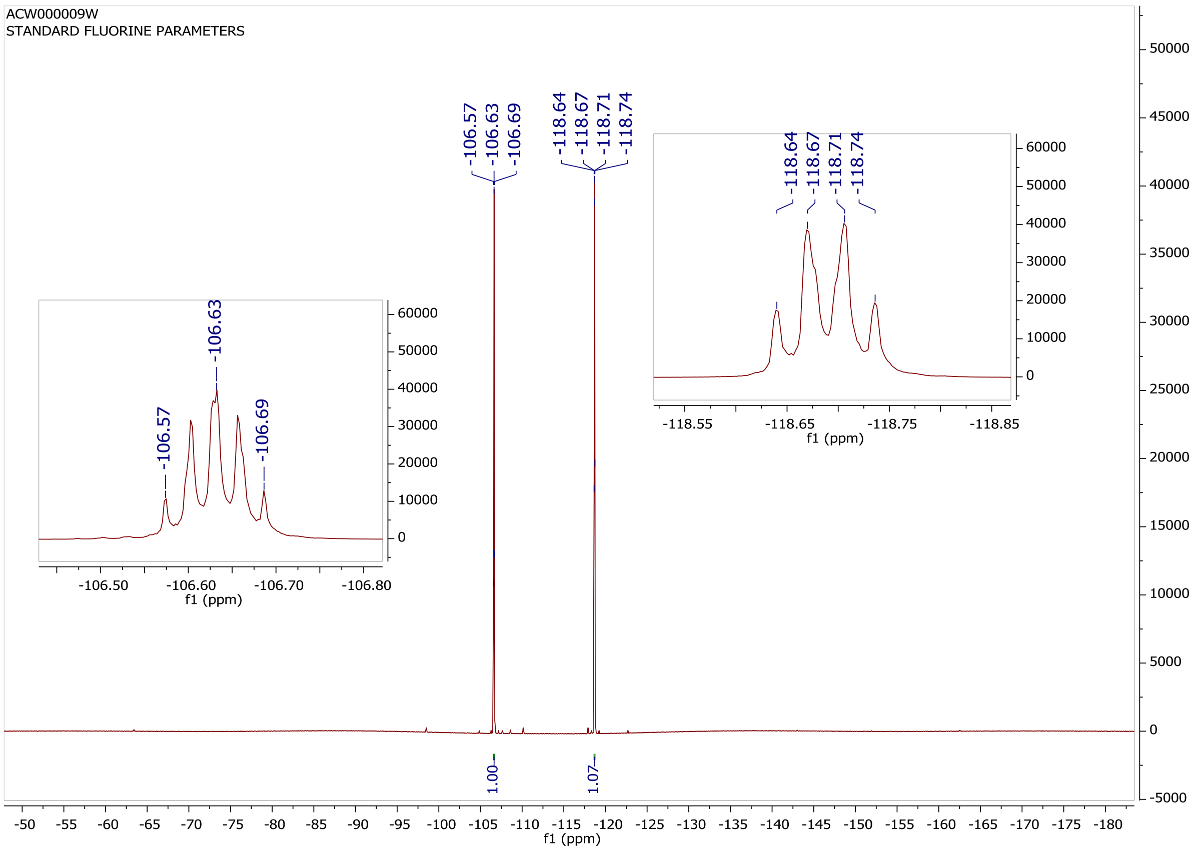

Figure S89. ^{19}**F NMR spectrum of 2h (CDCl$_3$)**

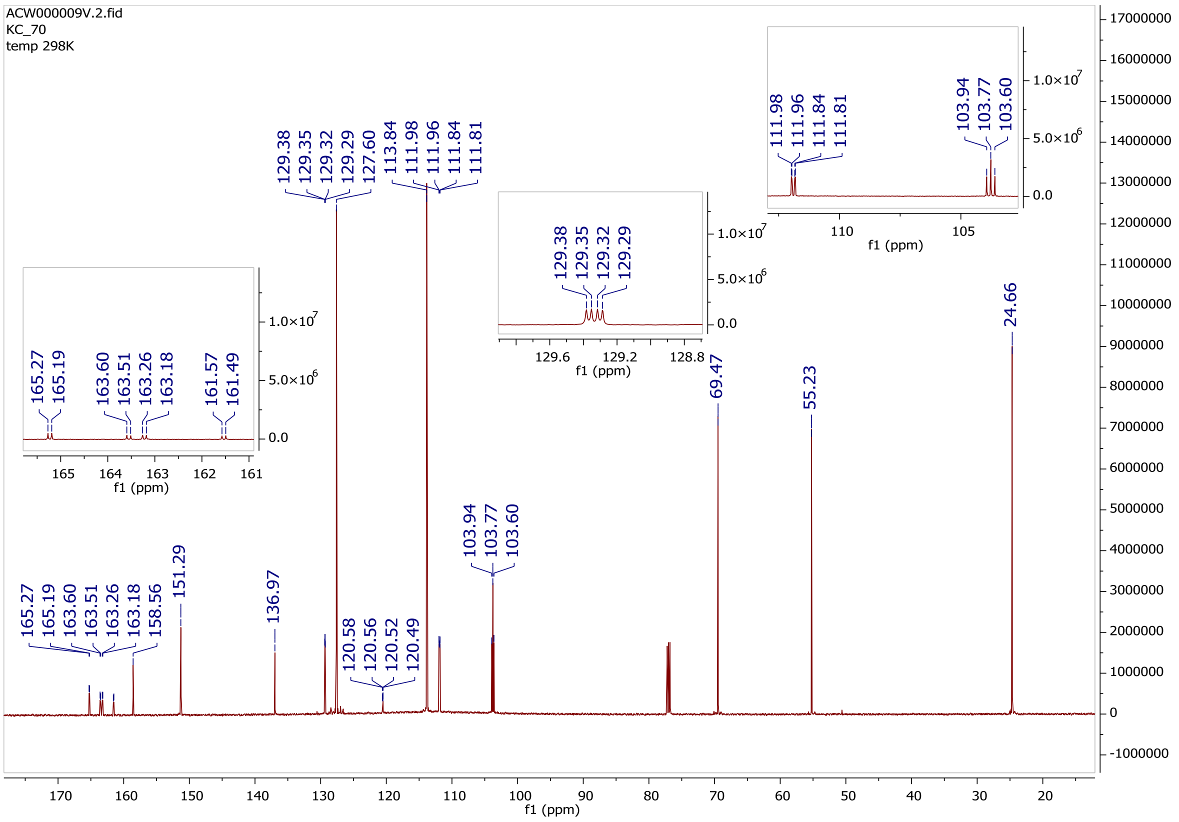

Figure S90. ^{13}**C NMR spectrum of 2h (CDCl$_3$)**

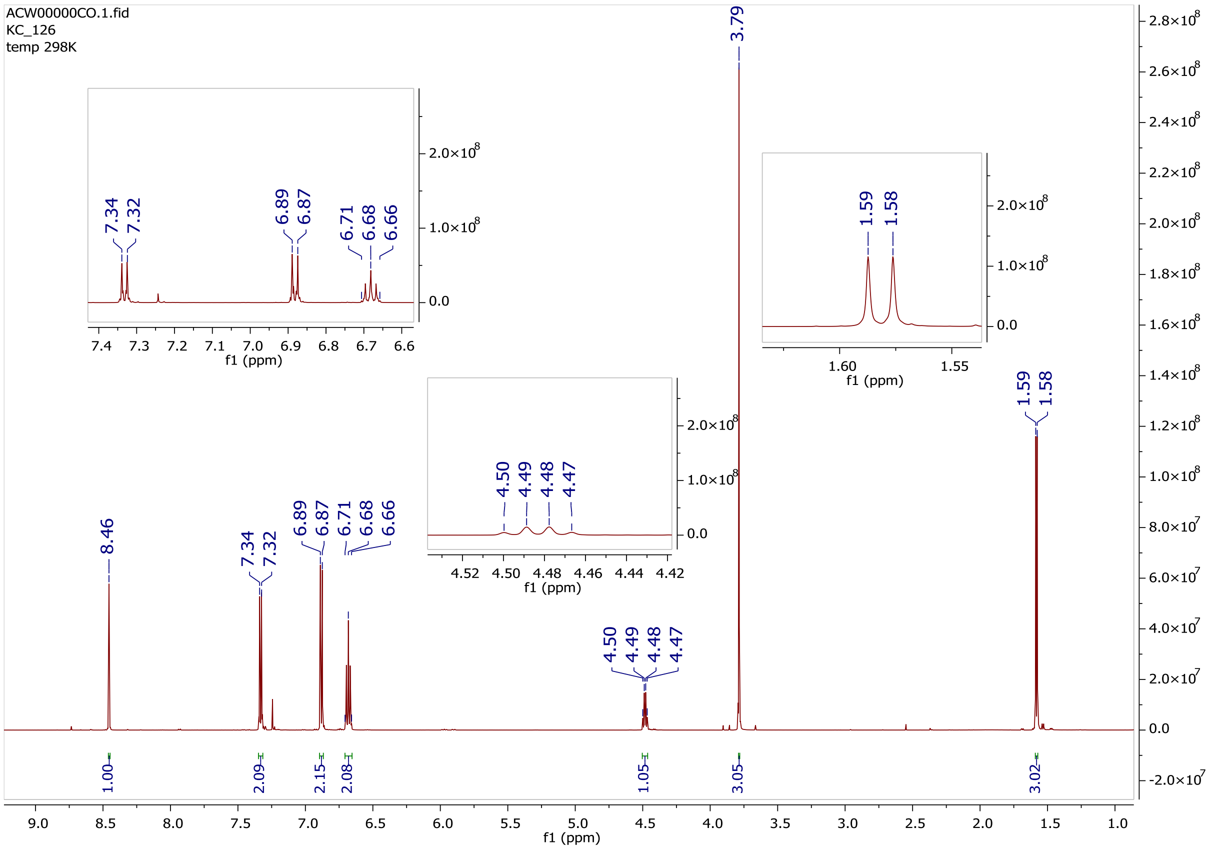

Figure S91. ^{1}H **NMR spectrum of 3h ($CDCl_3$)**

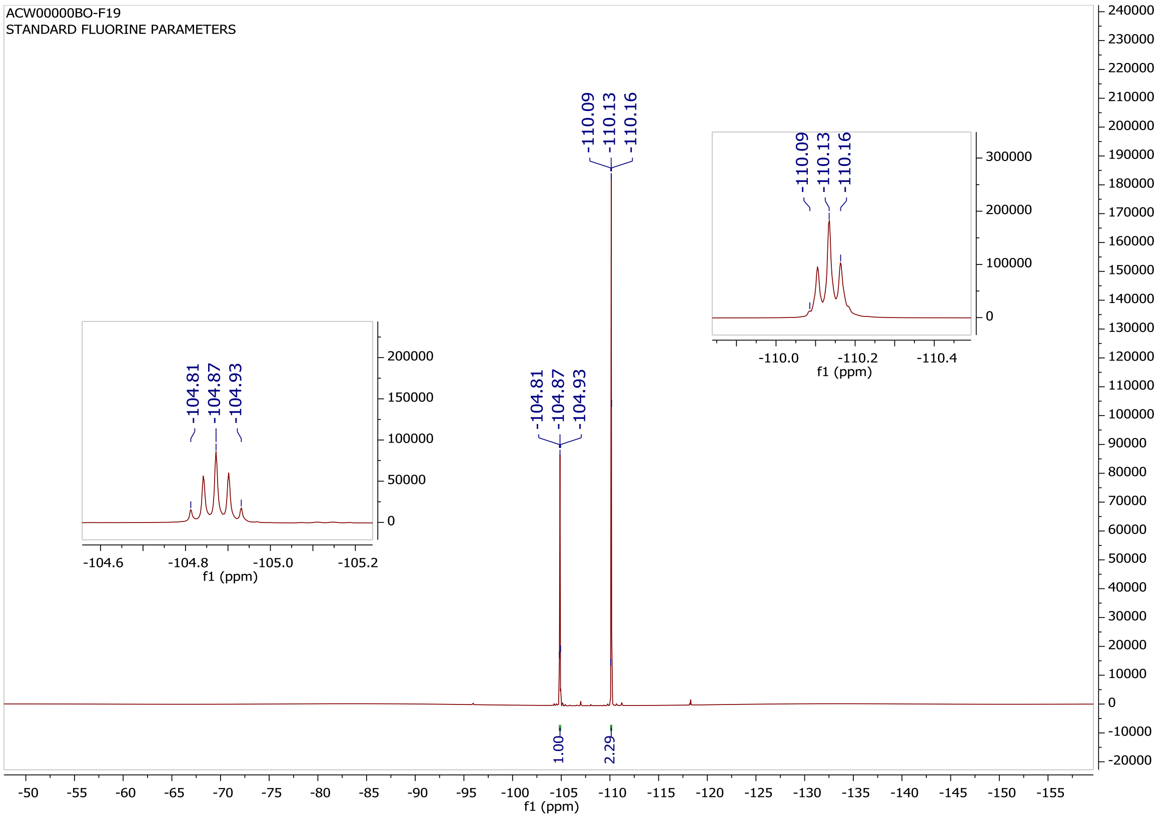

Figure S92. ^{19}F **NMR spectrum of 3h ($CDCl_3$)**

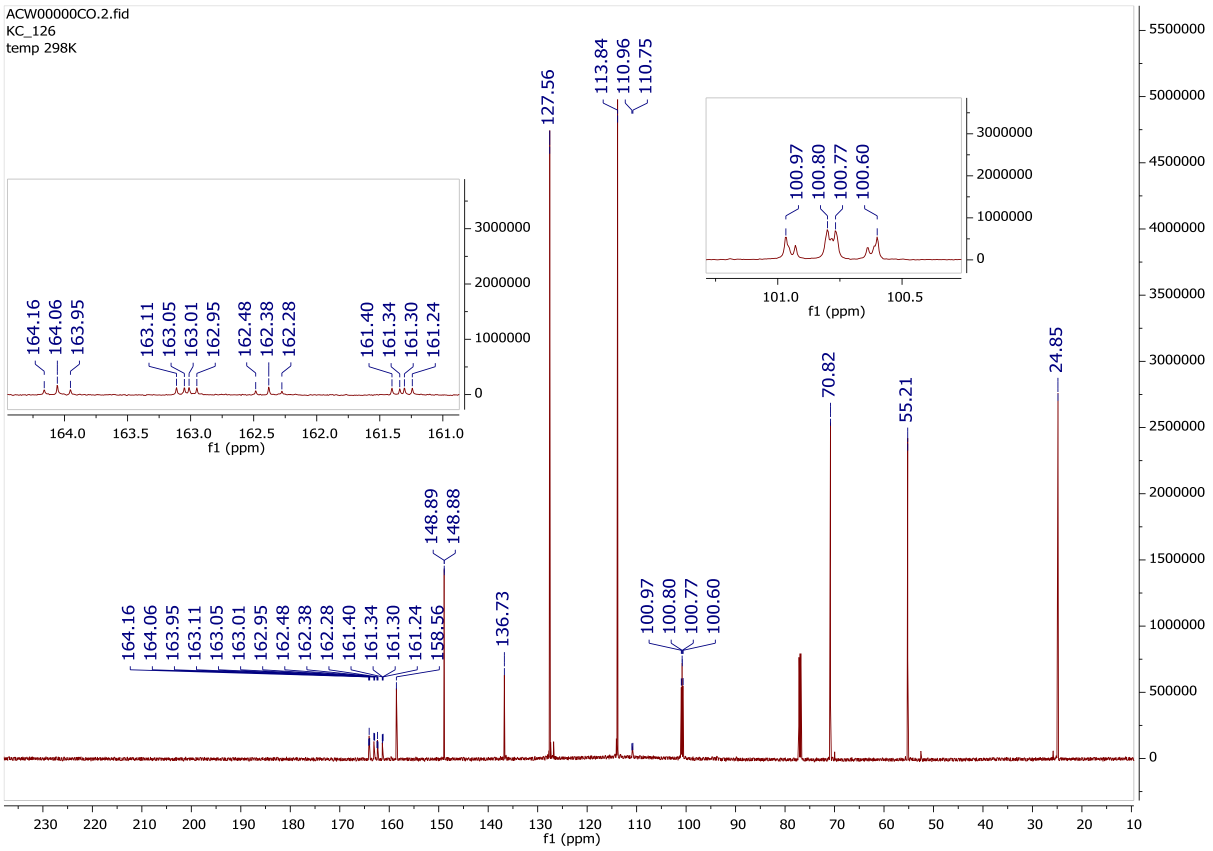

Figure S93. ^{13}**C NMR spectrum of 3h (CDCl$_3$)**

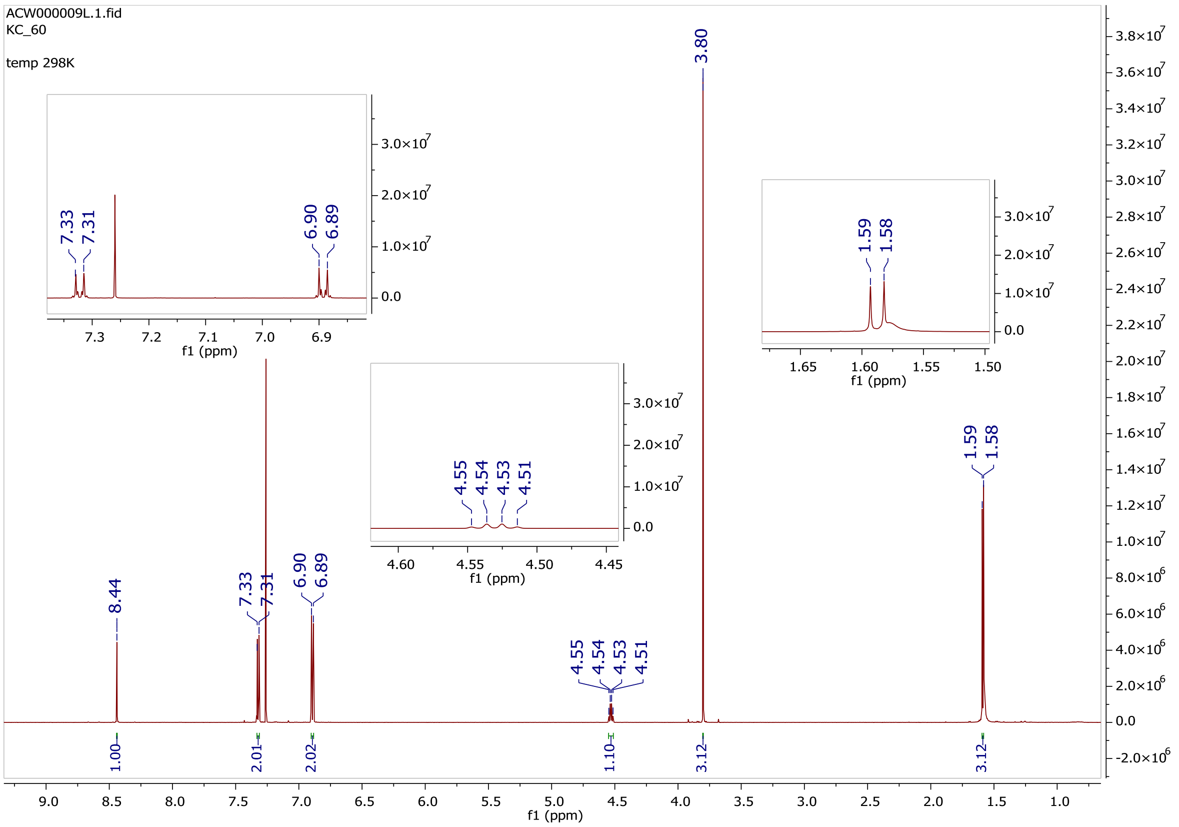

Figure S94. ^{1}**H NMR spectrum of 4h (CDCl$_3$)**

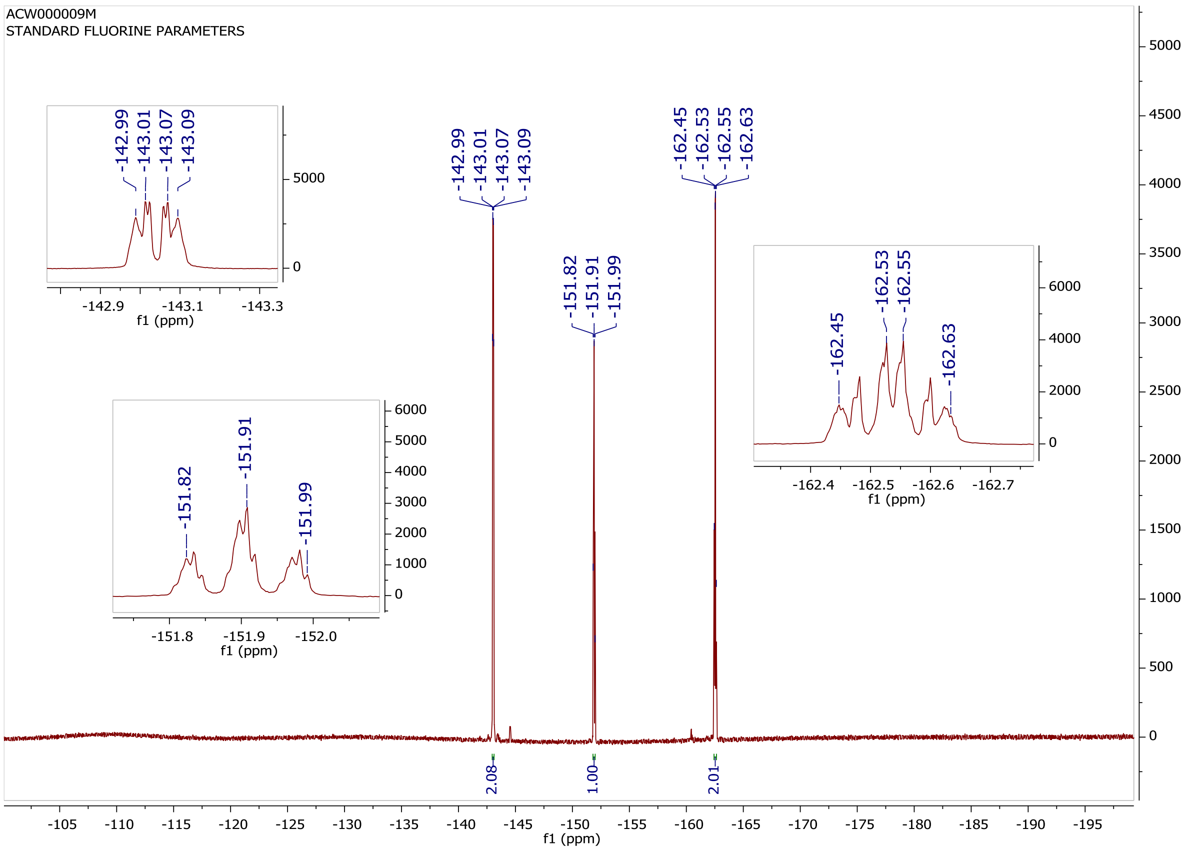

Figure S95. ^{19}**F NMR spectrum of 4h (CDCl$_3$)**

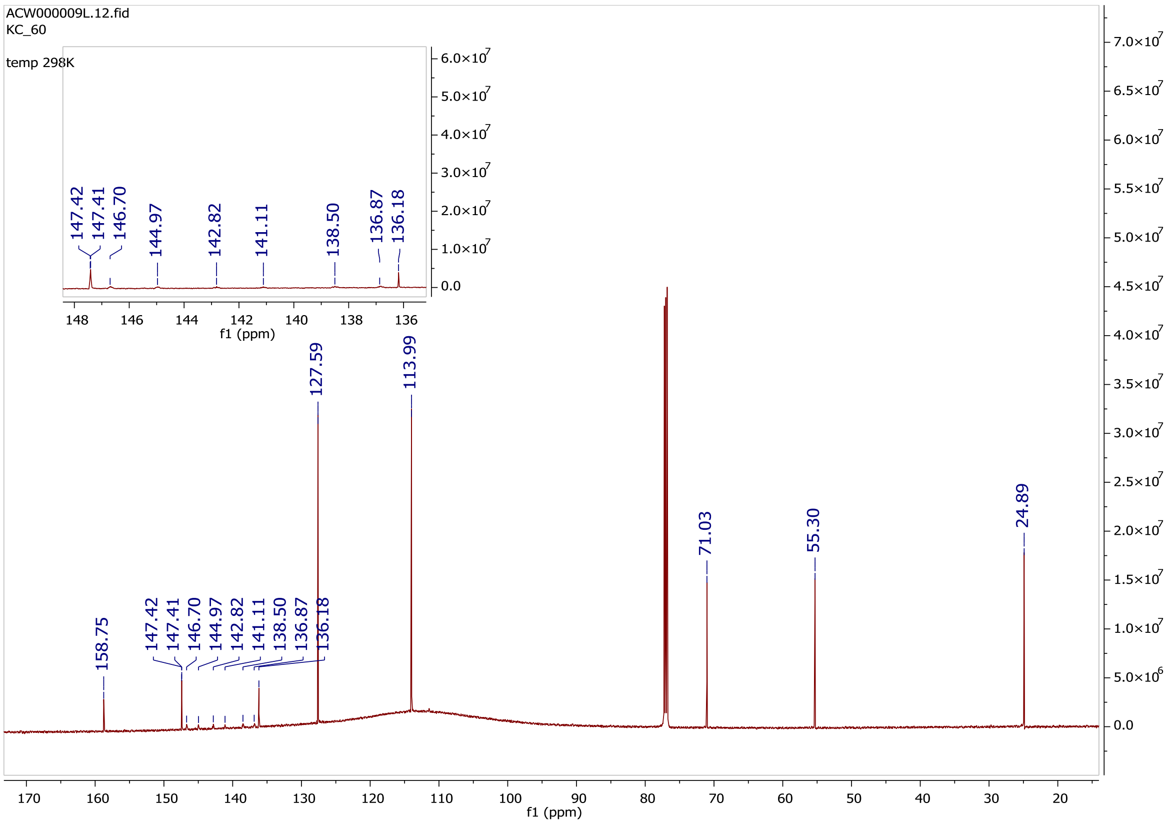

Figure S96. ^{13}**C NMR spectrum of 4h (CDCl$_3$)**

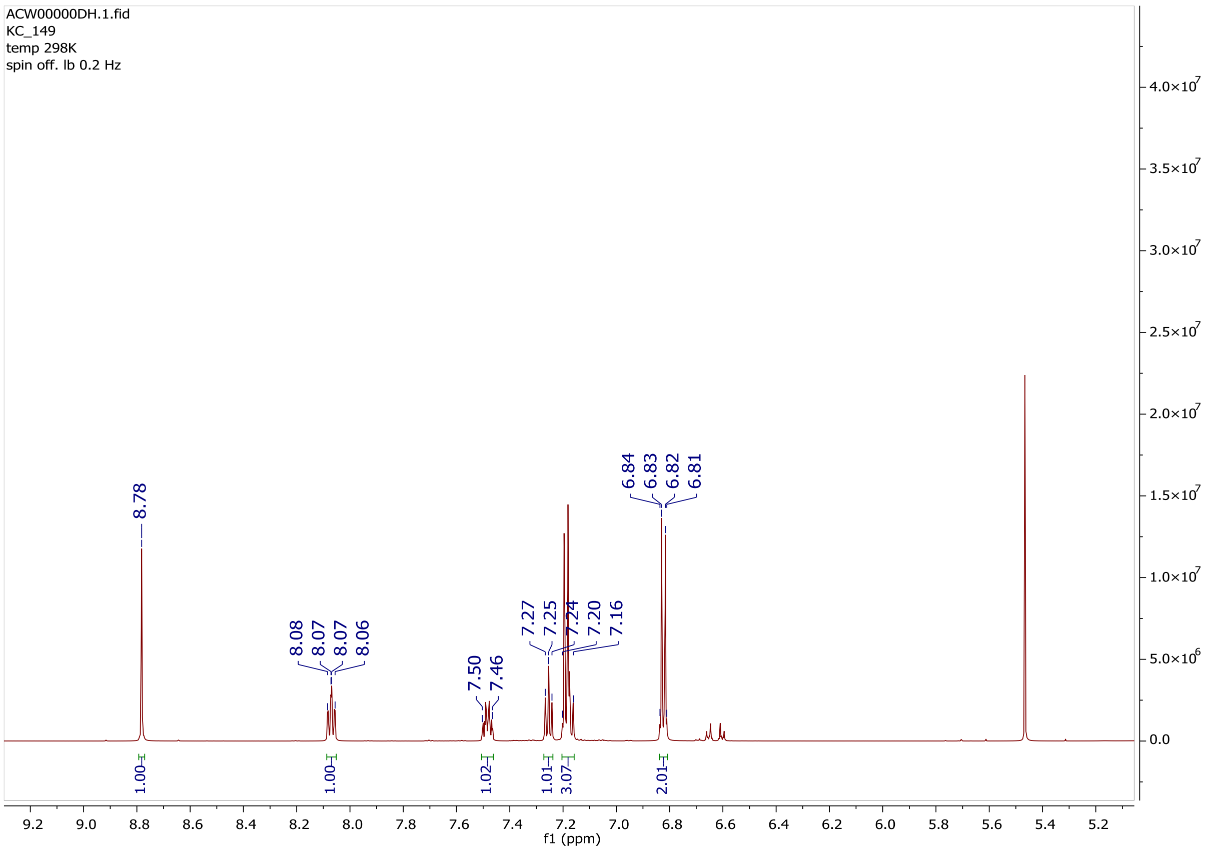

Figure S97. ^{1}H **NMR spectrum of 1i (CD_3OD)**

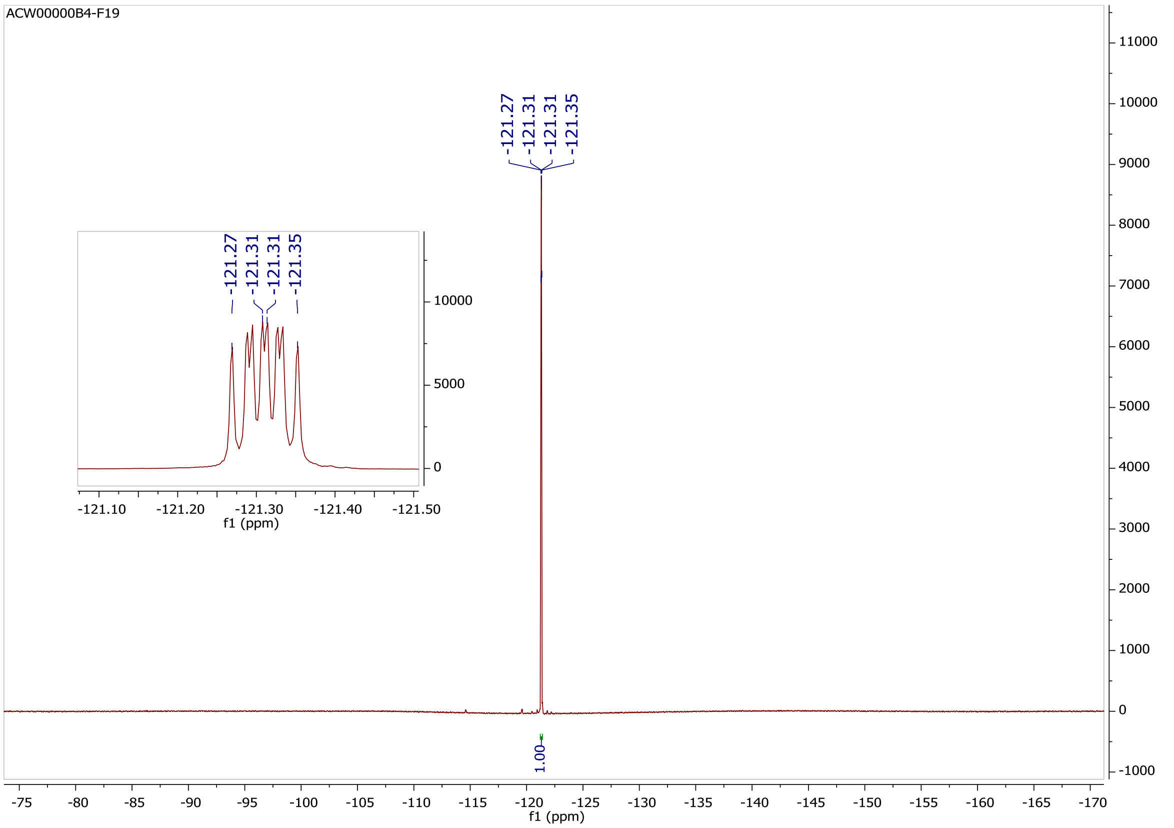

Figure S98. ^{19}F **NMR spectrum of 1i (CD_3OD)**

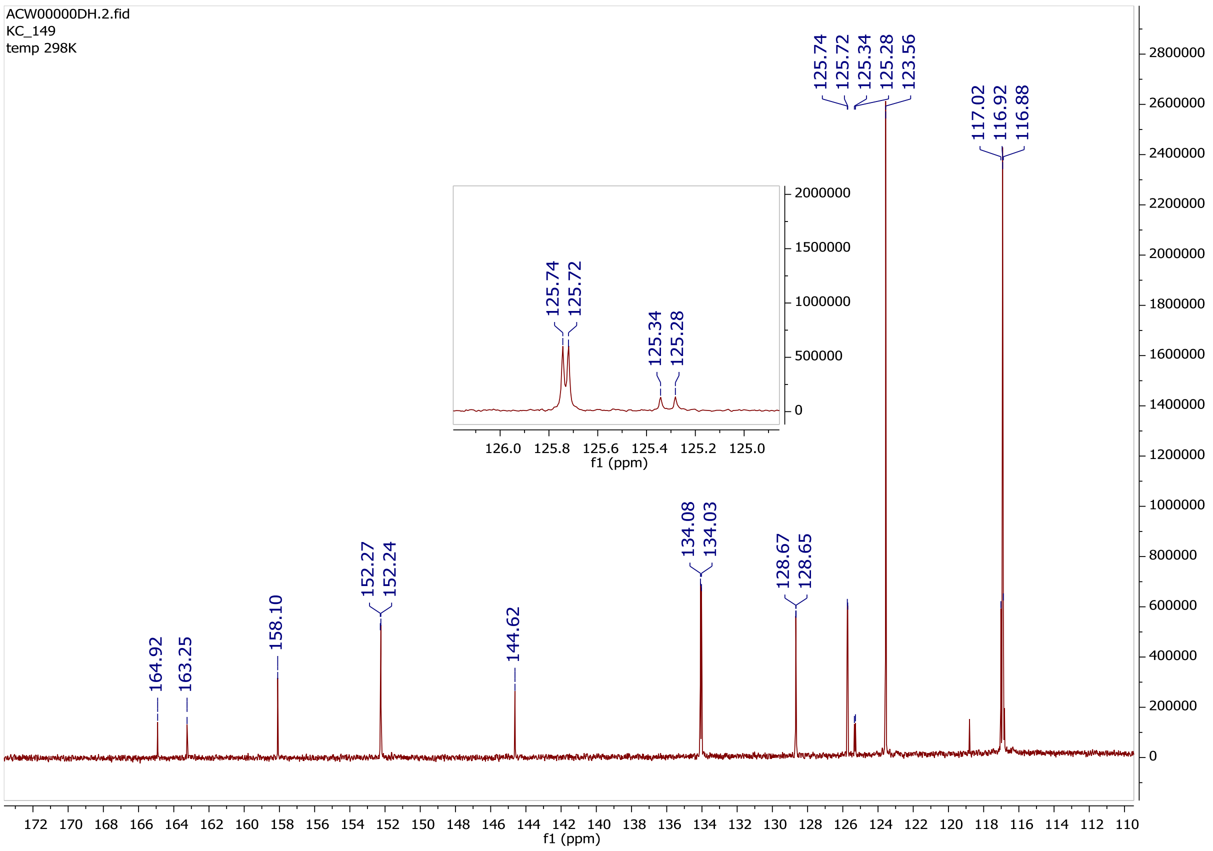

Figure S99. ^{13}**C NMR spectrum of 1i (**CD_3OD**)**

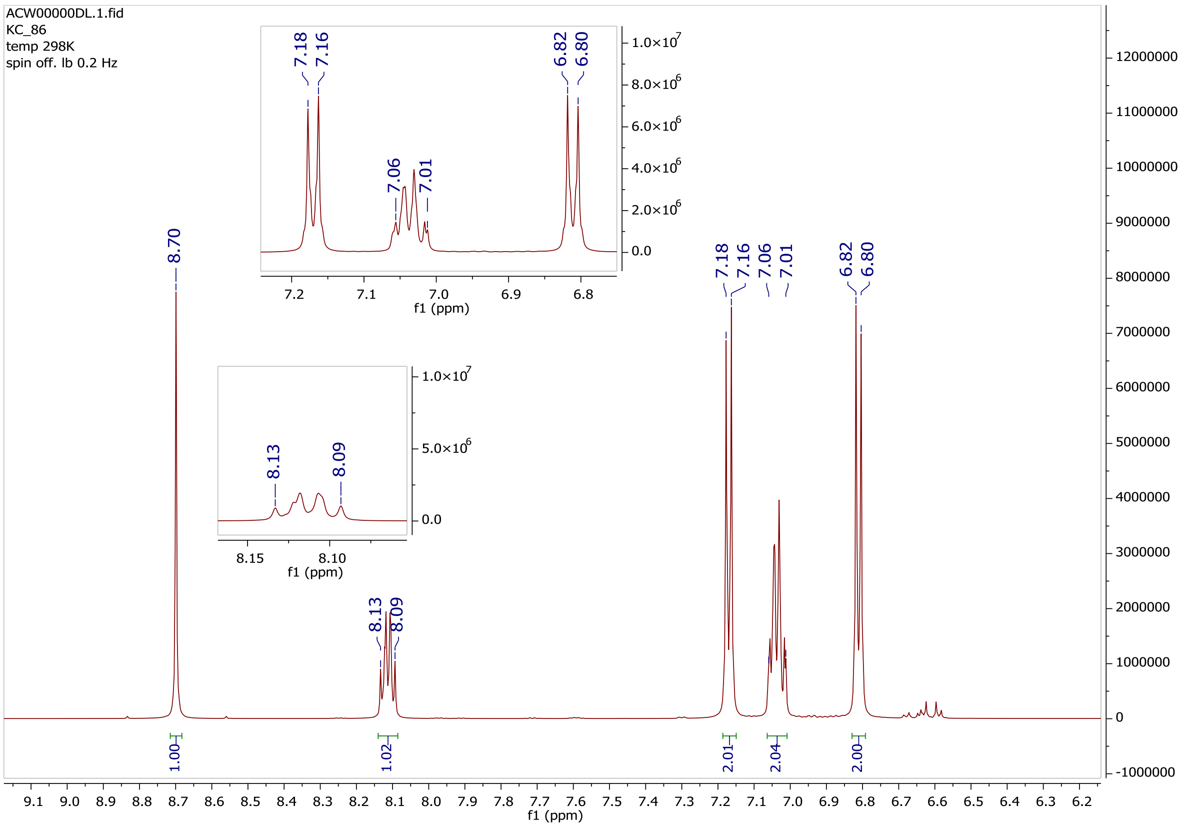

Figure S100. ^{1}**H NMR spectrum of 2i (**CD_3OD**)**

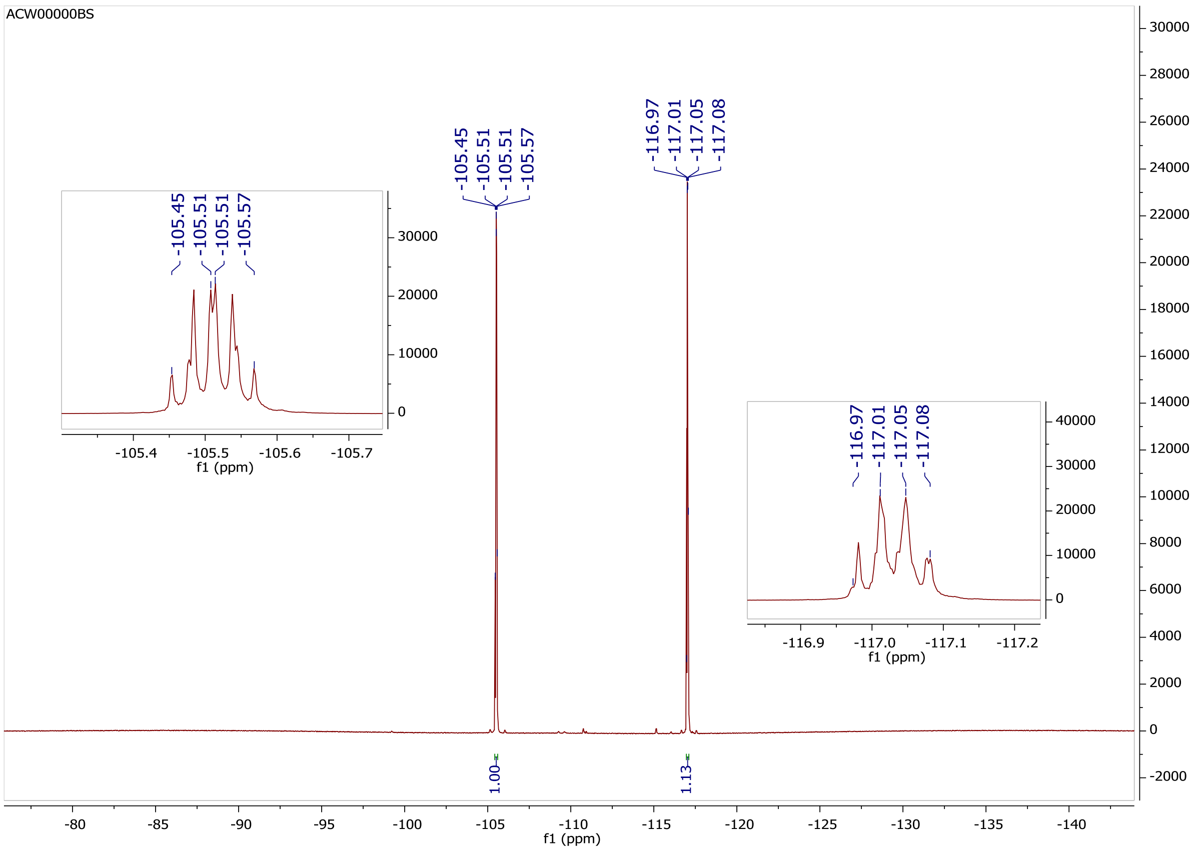

Figure S101. ^{19}**F NMR spectrum of 2i (**CD_3OD**)**

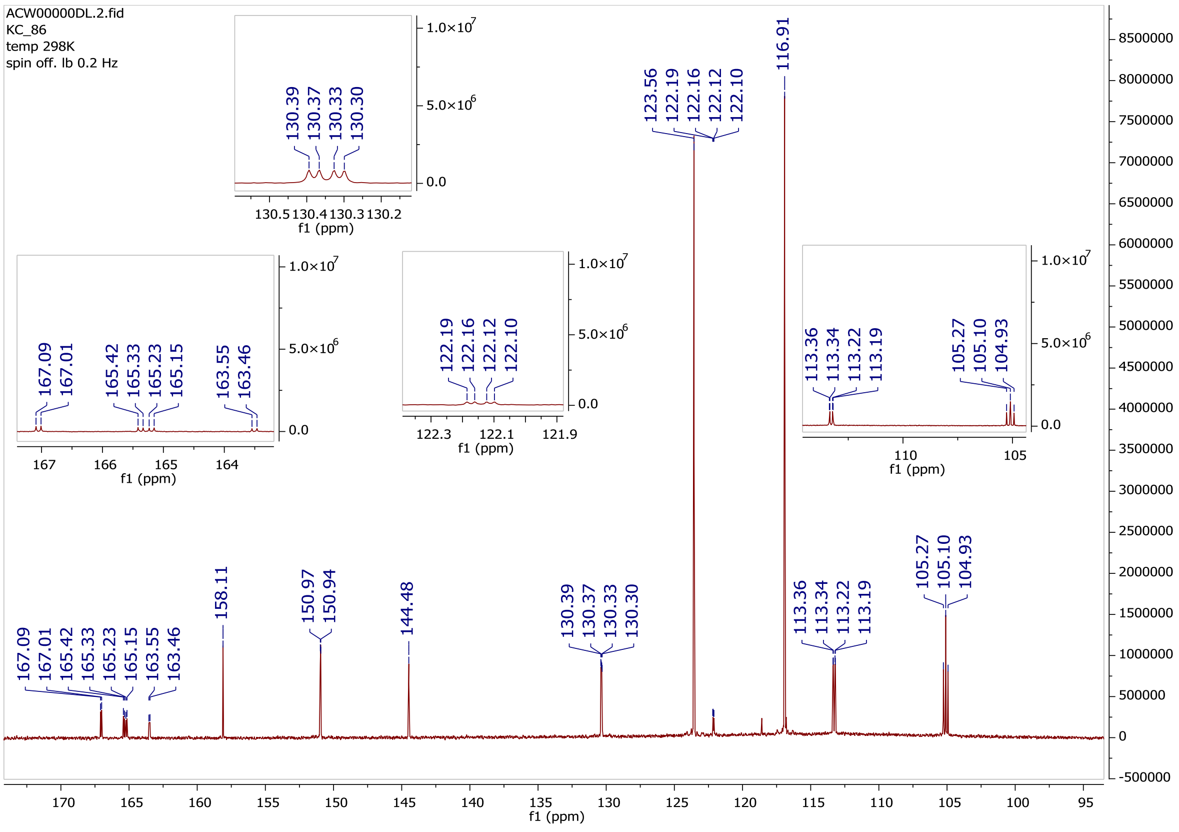

Figure S102. ^{13}**C NMR spectrum of 2i (**CD_3OD**)**

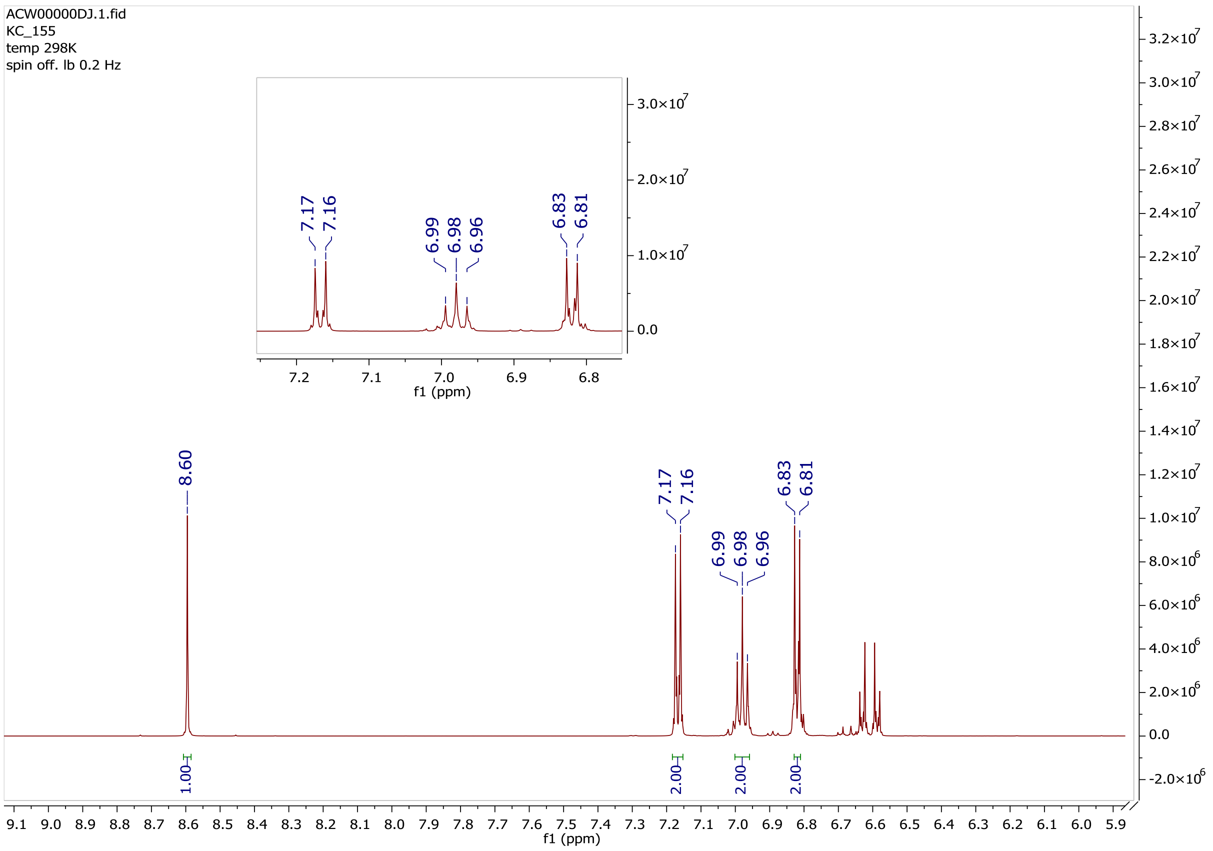

Figure S103. ^{1}**H NMR spectrum of 3i (CD$_3$OD)**

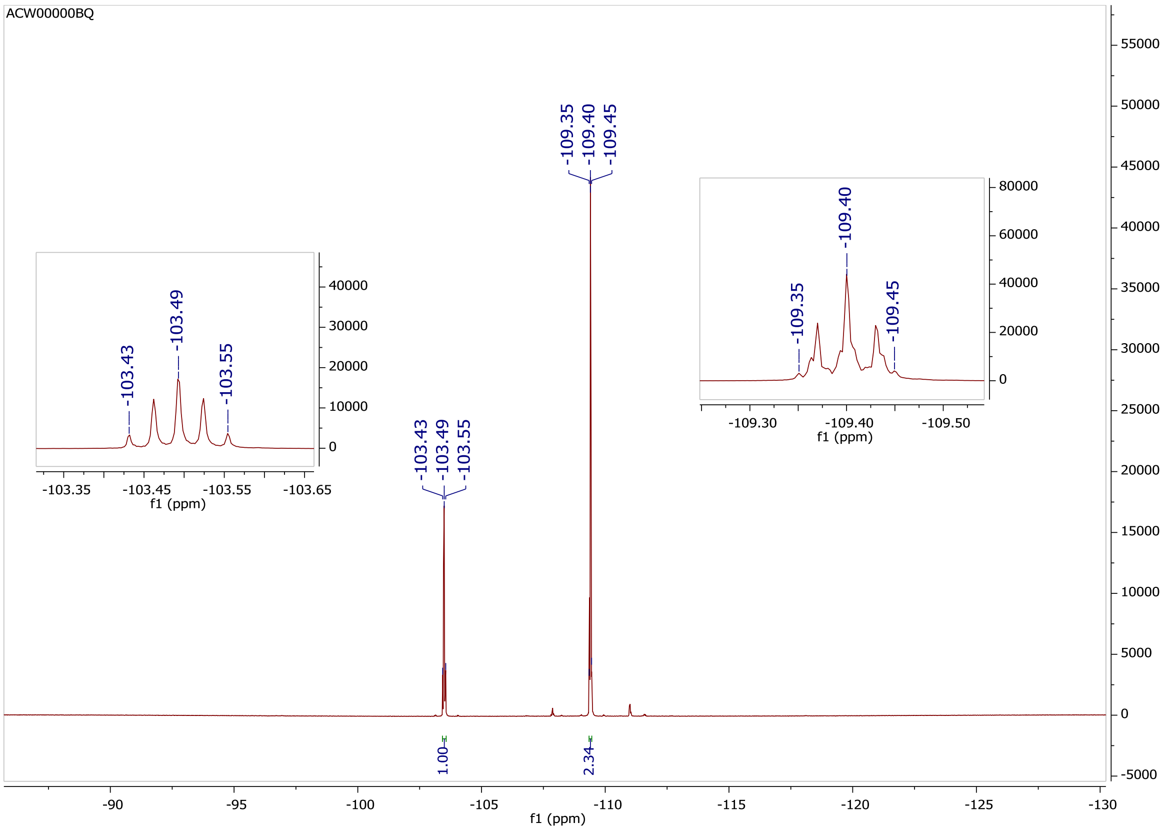

Figure S104. ^{19}**F NMR spectrum of 3i (CD$_3$OD)**

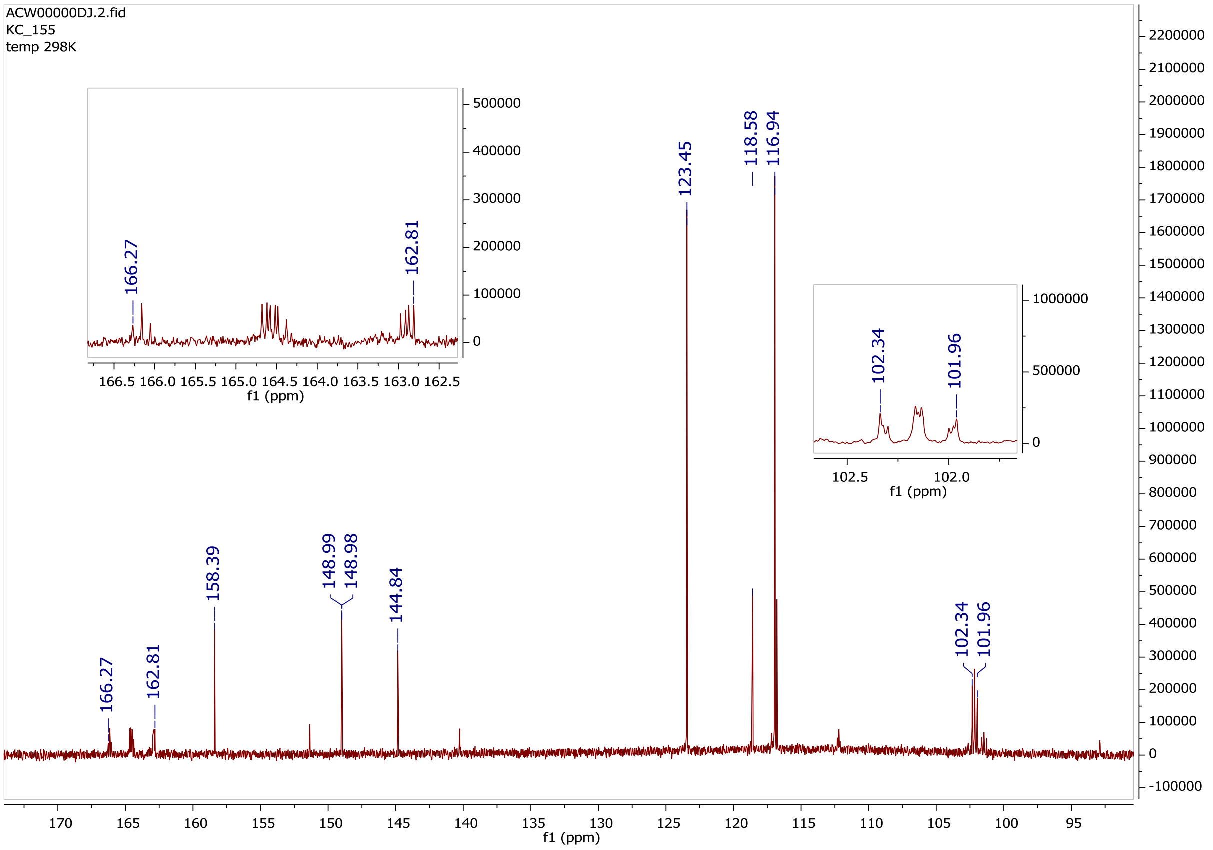

Figure S105. ^{13}**C NMR spectrum of 3i (CD$_3$OD)**

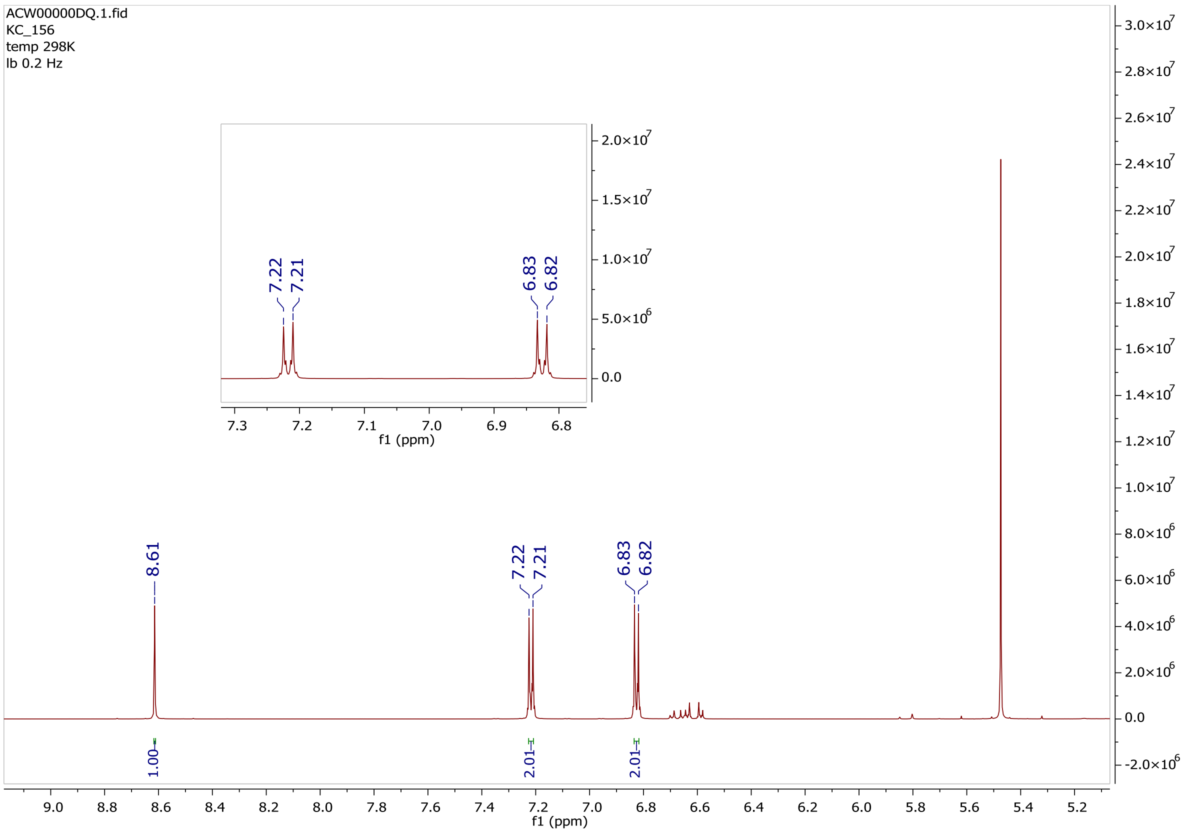

Figure S106. ^{1}**H NMR spectrum of 4i (CD$_3$OD)**

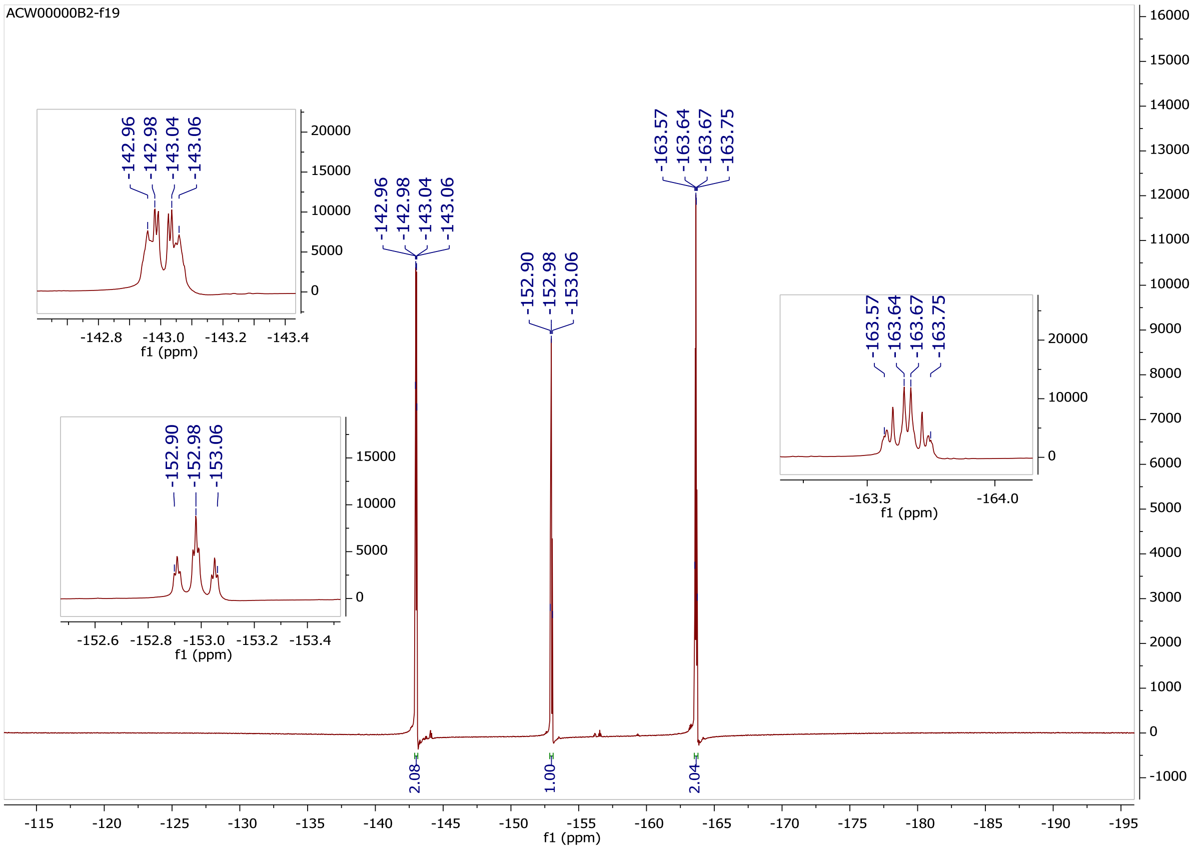

Figure S107. ^{19}**F NMR spectrum of 4i (**CD_3OD**)**

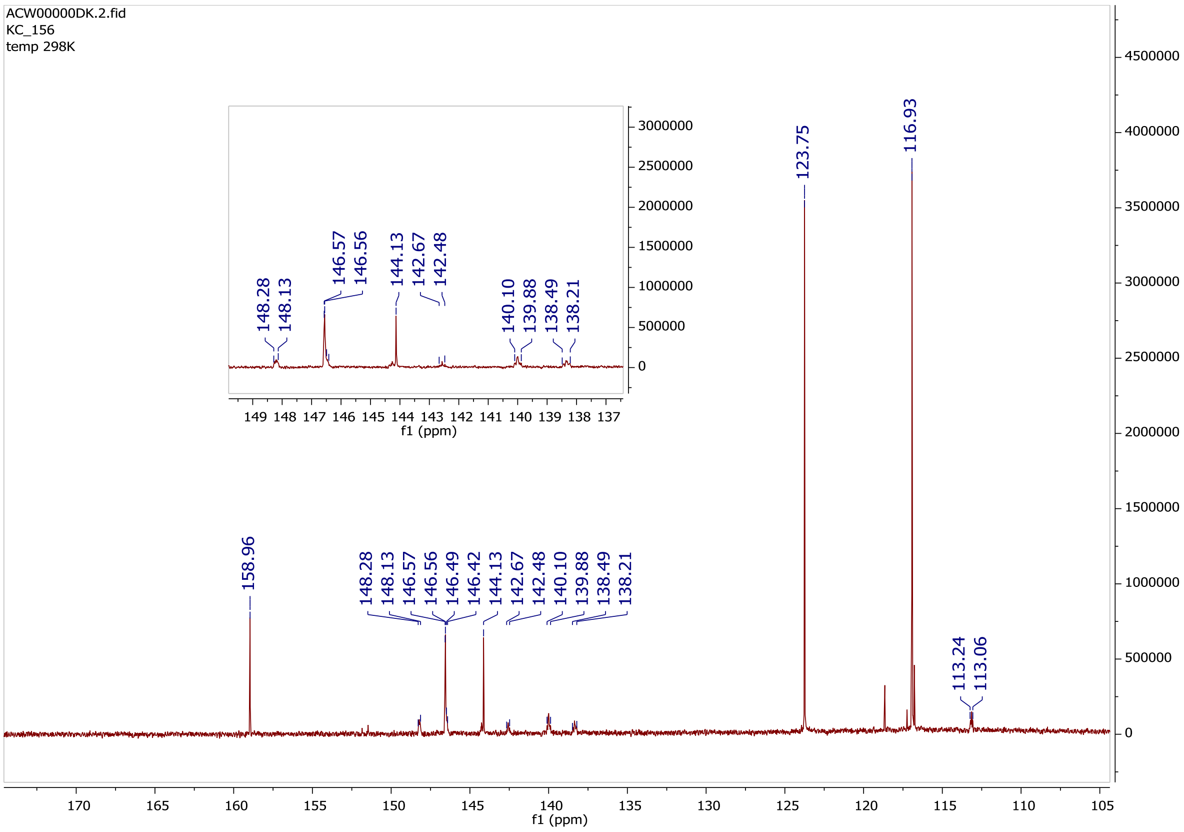

Figure S108. ^{13}**C NMR spectrum of 4i (**CD_3OD**)**

X-ray crystallography data

Table S1. Crystal data, data collection and structure refinement.

Compound	**1c**	**2a**	**3d**	**4d**
Formula	$C_{13}H_9BrFN$	$C_{13}H_8ClF_2N$	$C_{14}H_{10}F_3NO$	$C_{14}H_8F_5NO$
Formula weight	278.12	251.65	265.23	301.21
Crystal system	monoclinic	monoclinic	monoclinic	triclinic
Space group	$P2_1/c$	$P2_1/c$	$P2_1/c$	P-1
a (Å)	13.3894(3)	13.0347(3)	13.50700(18)	6.4217(3)
b (Å)	10.9801(3)	11.4697(3)	7.10152(9)	7.3068(4)
c (Å)	7.72595(19)	7.4832(2)	24.8299(3)	12.8876(6)
α (°)	90	90	90	85.930(4)
β (°)	106.126(2)	106.440(3)	92.4523(11)	80.022(4)
γ (°)	90	90	90	80.491(4)
V(Å^3)	1091.15(5)	1073.03(5)	2379.51(5)	586.82(5)
Z	4	4	8	2
D_x(g cm^{-3})	1.693	1.558	1.481	1.705
F(000)	552	512	1088	304
μ(mm^{-1})	3.749	0.356	1.088	0.162
Reflections:				
collected	3996	9167	10141	9572
unique (R_{int})	2154 (0.024)	2355 (0.022)	4847 (0.022)	2626 (0.019)
with I>2σ(I)	1921	2088	4410	2163
R(F) [I>2σ(I)]	0.0325	0.0299	0.0364	0.0350
wR(F^2) [I>2σ(I)]	0.1849	0.0693	0.0996	0.0966
R(F) [all data]	0.0379	0.0365	0.0398	0.0446
wR(F^2) [all data]	0.0888	0.0727	0.1024	0.1035
Goodness of fit	1.074	1.073	1.049	1.047
max/min Δρ (e·Å^{-}	0.64/-0.69	0.31/-0.20	0.25/-0.18	10.27/-0.28
CCDC number	2149773	2149774	2149775	2150609

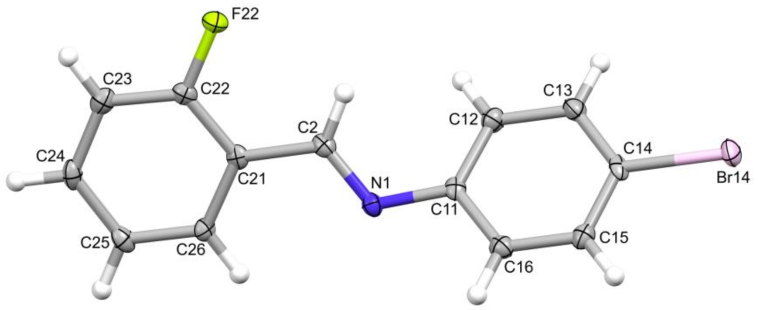

Figure S109. Perspective view of the molecule **1c** as seen in its crystal structure

checkCIF/PLATON report

Structure factors have been supplied for datablock(s) 1

THIS REPORT IS FOR GUIDANCE ONLY. IF USED AS PART OF A REVIEW PROCEDURE FOR PUBLICATION, IT SHOULD NOT REPLACE THE EXPERTISE OF AN EXPERIENCED CRYSTALLOGRAPHIC REFEREE.

No syntax errors found. CIF dictionary Interpreting this report

Datablock: 1

Bond precision:	C-C = 0.0036 A		Wavelength=0.71073
Cell:	a=13.3894(3)	b=10.9801(3)	c=7.72595(19)
	alpha=90	beta=106.126(2)	gamma=90
Temperature:	100 K		

	Calculated	Reported
Volume	1091.15(5)	1091.15(5)
Space group	P 21/c	P 21/c
Hall group	-P 2ybc	-P 2ybc
Moiety formula	C13 H9 Br F N	?
Sum formula	C13 H9 Br F N	C13 H9 Br F N
Mr	278.11	278.12
Dx,g cm-3	1.693	1.693
Z	4	4
Mu (mm-1)	3.749	3.749
F000	552.0	552.0
F000’	551.10	
h,k,lmax	17,13,9	17,13,9
Nref	2343	2154
Tmin,Tmax	0.644,0.687	0.135,1.000
Tmin’	0.434	

Correction method= # Reported T Limits: Tmin=0.135 Tmax=1.000
AbsCorr = MULTI-SCAN

Data completeness= 0.919 Theta(max)= 26.899

R(reflections)= 0.0325(1921) wR2(reflections)= 0.0888(2154)

S = 1.074 Npar= 146

The following ALERTS were generated. Each ALERT has the format
test-name_ALERT_alert-type_alert-level.
Click on the hyperlinks for more details of the test.

Alert level C

```
PLAT911_ALERT_3_C Missing FCF Refl Between Thmin & STh/L=    0.600           5 Report
```

Alert level G

```
PLAT910_ALERT_3_G Missing # of FCF Reflection(s) Below Theta(Min).          3 Note
PLAT912_ALERT_4_G Missing # of FCF Reflections Above STh/L=  0.600        182 Note
PLAT941_ALERT_3_G Average HKL Measurement Multiplicity ..........         1.9 Low
PLAT953_ALERT_1_G Reported (CIF) and Actual (FCF) Hmax Differ by .          1 Units
PLAT978_ALERT_2_G Number C-C Bonds with Positive Residual Density.          9 Info
```

0 **ALERT level A** = Most likely a serious problem - resolve or explain
0 **ALERT level B** = A potentially serious problem, consider carefully
1 **ALERT level C** = Check. Ensure it is not caused by an omission or oversight
5 **ALERT level G** = General information/check it is not something unexpected

1 ALERT type 1 CIF construction/syntax error, inconsistent or missing data
1 ALERT type 2 Indicator that the structure model may be wrong or deficient
3 ALERT type 3 Indicator that the structure quality may be low
1 ALERT type 4 Improvement, methodology, query or suggestion
0 ALERT type 5 Informative message, check

It is advisable to attempt to resolve as many as possible of the alerts in all categories. Often the minor alerts point to easily fixed oversights, errors and omissions in your CIF or refinement strategy, so attention to these fine details can be worthwhile. In order to resolve some of the more serious problems it may be necessary to carry out additional measurements or structure refinements. However, the purpose of your study may justify the reported deviations and the more serious of these should normally be commented upon in the discussion or experimental section of a paper or in the "special_details" fields of the CIF. checkCIF was carefully designed to identify outliers and unusual parameters, but every test has its limitations and alerts that are not important in a particular case may appear. Conversely, the absence of alerts does not guarantee there are no aspects of the results needing attention. It is up to the individual to critically assess their own results and, if necessary, seek expert advice.

Publication of your CIF in IUCr journals

A basic structural check has been run on your CIF. These basic checks will be run on all CIFs submitted for publication in IUCr journals (*Acta Crystallographica*, *Journal of Applied Crystallography*, *Journal of Synchrotron Radiation*); however, if you intend to submit to *Acta Crystallographica Section C* or *E* or *IUCrData*, you should make sure that full publication checks are run on the final version of your CIF prior to submission.

Publication of your CIF in other journals

Please refer to the *Notes for Authors* of the relevant journal for any special instructions relating to CIF submission.

PLATON version of 20/01/2022; check.def file version of 19/01/2022

Datablock 1 - ellipsoid plot

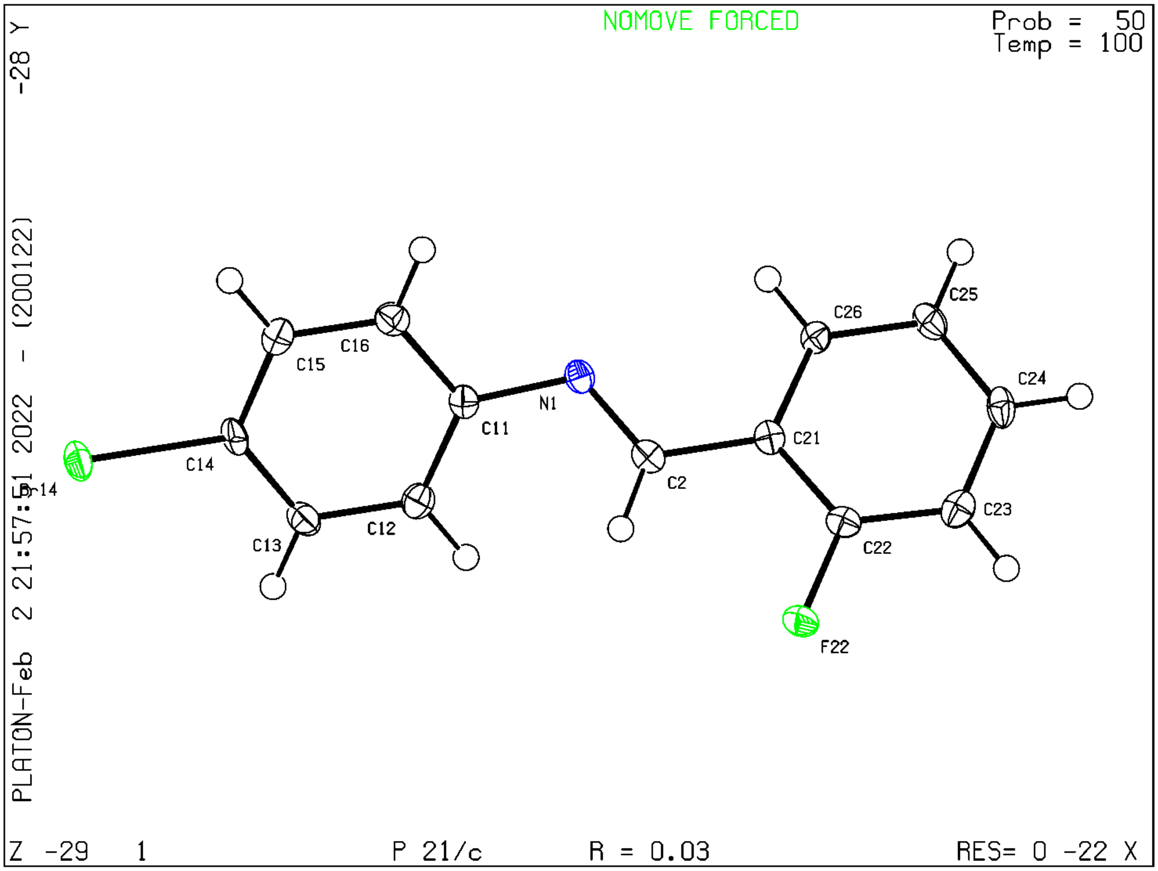

checkCIF/PLATON report

Structure factors have been supplied for datablock(s) 2

THIS REPORT IS FOR GUIDANCE ONLY. IF USED AS PART OF A REVIEW PROCEDURE FOR PUBLICATION, IT SHOULD NOT REPLACE THE EXPERTISE OF AN EXPERIENCED CRYSTALLOGRAPHIC REFEREE.

No syntax errors found. CIF dictionary Interpreting this report

Datablock: 2

```
Bond precision:   C-C = 0.0020 A          Wavelength=0.71073

Cell:             a=13.0347(3)     b=11.4697(3)       c=7.4832(2)
                  alpha=90         beta=106.440(3)    gamma=90
Temperature:      100 K

                 Calculated                    Reported
Volume           1073.03(5)                    1073.03(5)
Space group      P 21/c                        P 21/c
Hall group       -P 2ybc                       -P 2ybc
Moiety formula   C13 H8 Cl F2 N                C13 H8 Cl F2 N
Sum formula      C13 H8 Cl F2 N                C13 H8 Cl F2 N
Mr               251.65                        251.65
Dx,g cm-3        1.558                         1.558
Z                4                             4
Mu (mm-1)        0.356                         0.356
F000             512.0                         512.0
F000'            512.86
h,k,lmax         17,15,10                      16,15,9
Nref             2763                          2355
Tmin,Tmax        0.934,0.965                   0.936,1.000
Tmin'            0.925

Correction method= # Reported T Limits: Tmin=0.936 Tmax=1.000
AbsCorr = MULTI-SCAN

Data completeness= 0.852           Theta(max)= 28.634

R(reflections)= 0.0299( 2088)                          wR2(reflections)=
                                                       0.0727( 2355)
S = 1.073                   Npar= 154
```

The following ALERTS were generated. Each ALERT has the format
test-name_ALERT_alert-type_alert-level.
Click on the hyperlinks for more details of the test.

Alert level C

PLAT906_ALERT_3_C Large K Value in the Analysis of Variance ...... 2.035 Check

Alert level G

PLAT910_ALERT_3_G Missing # of FCF Reflection(s) Below Theta(Min). 4 Note
PLAT912_ALERT_4_G Missing # of FCF Reflections Above STh/L= 0.600 378 Note
PLAT941_ALERT_3_G Average HKL Measurement Multiplicity .... 3.9 Low
PLAT978_ALERT_2_G Number C-C Bonds with Positive Residual Density. 11 Info

0 **ALERT level A** = Most likely a serious problem - resolve or explain
0 **ALERT level B** = A potentially serious problem, consider carefully
1 **ALERT level C** = Check. Ensure it is not caused by an omission or oversight
4 **ALERT level G** = General information/check it is not something unexpected

0 ALERT type 1 CIF construction/syntax error, inconsistent or missing data
1 ALERT type 2 Indicator that the structure model may be wrong or deficient
3 ALERT type 3 Indicator that the structure quality may be low
1 ALERT type 4 Improvement, methodology, query or suggestion
0 ALERT type 5 Informative message, check

It is advisable to attempt to resolve as many as possible of the alerts in all categories. Often the minor alerts point to easily fixed oversights, errors and omissions in your CIF or refinement strategy, so attention to these fine details can be worthwhile. In order to resolve some of the more serious problems it may be necessary to carry out additional measurements or structure refinements. However, the purpose of your study may justify the reported deviations and the more serious of these should normally be commented upon in the discussion or experimental section of a paper or in the "special_details" fields of the CIF. checkCIF was carefully designed to identify outliers and unusual parameters, but every test has its limitations and alerts that are not important in a particular case may appear. Conversely, the absence of alerts does not guarantee there are no aspects of the results needing attention. It is up to the individual to critically assess their own results and, if necessary, seek expert advice.

Publication of your CIF in IUCr journals

A basic structural check has been run on your CIF. These basic checks will be run on all CIFs submitted for publication in IUCr journals (*Acta Crystallographica*, *Journal of Applied Crystallography*, *Journal of Synchrotron Radiation*); however, if you intend to submit to *Acta Crystallographica Section C* or *E* or *IUCrData*, you should make sure that full publication checks are run on the final version of your CIF prior to submission.

Publication of your CIF in other journals

Please refer to the *Notes for Authors* of the relevant journal for any special instructions relating to CIF submission.

PLATON version of 20/01/2022; check.def file version of 19/01/2022

Datablock 2 - ellipsoid plot

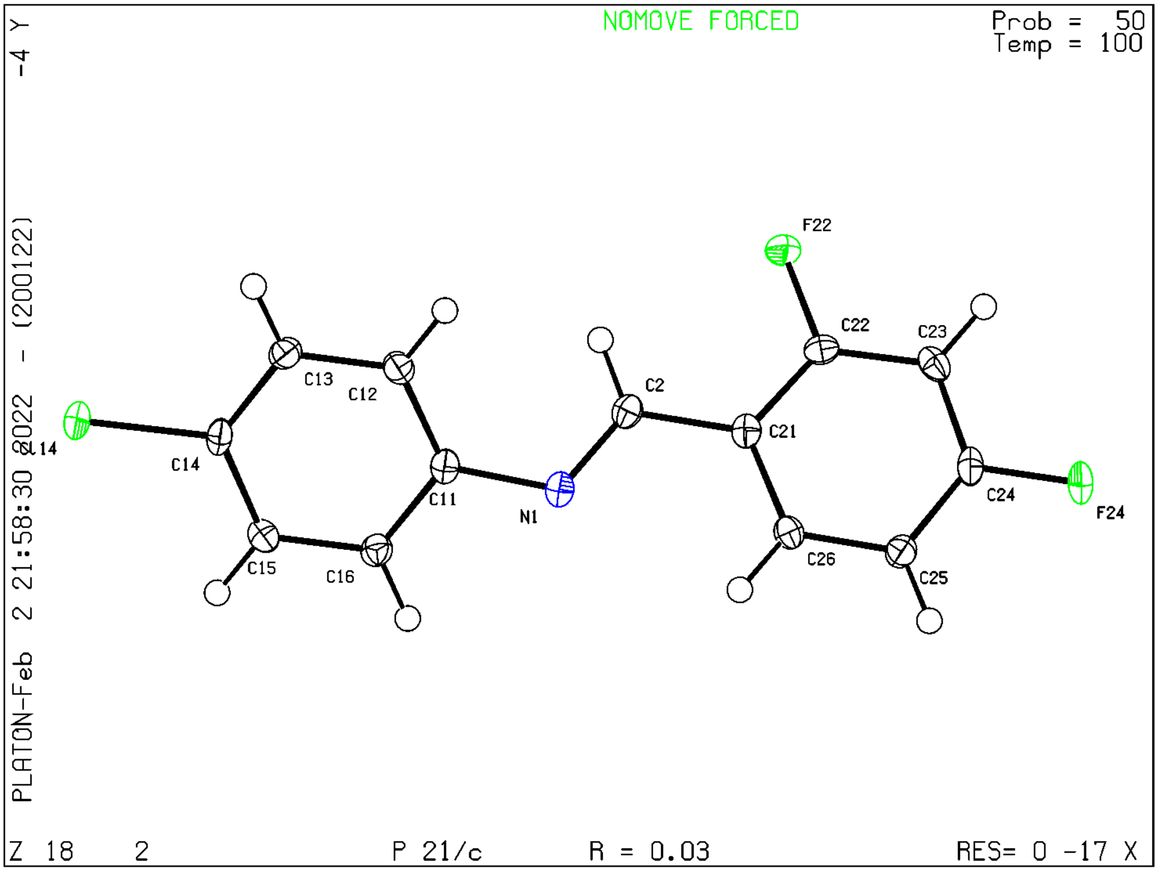

checkCIF/PLATON report

Structure factors have been supplied for datablock(s) 3

THIS REPORT IS FOR GUIDANCE ONLY. IF USED AS PART OF A REVIEW PROCEDURE FOR PUBLICATION, IT SHOULD NOT REPLACE THE EXPERTISE OF AN EXPERIENCED CRYSTALLOGRAPHIC REFEREE.

No syntax errors found. CIF dictionary Interpreting this report

Datablock: 3

```
Bond precision:   C-C = 0.0018 A           Wavelength=1.54184

Cell:            a=13.50700(18)    b=7.10152(9)       c=24.8299(3)
                 alpha=90          beta=92.4523(11)   gamma=90
Temperature:     130 K

                 Calculated                  Reported
Volume           2379.51(5)                  2379.51(5)
Space group      P 21/c                      P 21/c
Hall group       -P 2ybc                     -P 2ybc
Moiety formula   C14 H10 F3 N O              ?
Sum formula      C14 H10 F3 N O              C14 H10 F3 N O
Mr               265.23                      265.23
Dx,g cm-3        1.481                       1.481
Z                8                           8
Mu (mm-1)        1.088                       1.088
F000             1088.0                      1088.0
F000'            1092.26
h,k,lmax         17,8,31                     17,8,31
Nref             4975                        4847
Tmin,Tmax        0.758,0.804                 0.678,1.000
Tmin'            0.651

Correction method= # Reported T Limits: Tmin=0.678 Tmax=1.000
AbsCorr = MULTI-SCAN

Data completeness= 0.974           Theta(max)= 76.306

R(reflections)= 0.0364( 4410)                       wR2(reflections)=
                                                    0.1024( 4847)
S = 1.049                    Npar= 346
```

The following ALERTS were generated. Each ALERT has the format
test-name_ALERT_alert-type_alert-level.
Click on the hyperlinks for more details of the test.

Alert level C

```
PLAT906_ALERT_3_C Large K Value in the Analysis of Variance ......      2.131 Check
PLAT911_ALERT_3_C Missing FCF Refl Between Thmin & STh/L=    0.600          6 Report
```

Alert level G

```
PLAT434_ALERT_2_G Short Inter HL..HL Contact F22A     ..F22B     .       2.81 Ang.
                                              1-x,1-y,1-z  =      3_666 Check
PLAT912_ALERT_4_G Missing # of FCF Reflections Above STh/L=  0.600        123 Note
PLAT913_ALERT_3_G Missing # of Very Strong Reflections in FCF ....          3 Note
PLAT933_ALERT_2_G Number of HKL-OMIT Records in Embedded .res File          1 Note
PLAT941_ALERT_3_G Average HKL Measurement Multiplicity ..........         2.1 Low
PLAT978_ALERT_2_G Number C-C Bonds with Positive Residual Density.         23 Info
```

0 **ALERT level A** = Most likely a serious problem - resolve or explain
0 **ALERT level B** = A potentially serious problem, consider carefully
2 **ALERT level C** = Check. Ensure it is not caused by an omission or oversight
6 **ALERT level G** = General information/check it is not something unexpected

0 ALERT type 1 CIF construction/syntax error, inconsistent or missing data
3 ALERT type 2 Indicator that the structure model may be wrong or deficient
4 ALERT type 3 Indicator that the structure quality may be low
1 ALERT type 4 Improvement, methodology, query or suggestion
0 ALERT type 5 Informative message, check

It is advisable to attempt to resolve as many as possible of the alerts in all categories. Often the minor alerts point to easily fixed oversights, errors and omissions in your CIF or refinement strategy, so attention to these fine details can be worthwhile. In order to resolve some of the more serious problems it may be necessary to carry out additional measurements or structure refinements. However, the purpose of your study may justify the reported deviations and the more serious of these should normally be commented upon in the discussion or experimental section of a paper or in the "special_details" fields of the CIF. checkCIF was carefully designed to identify outliers and unusual parameters, but every test has its limitations and alerts that are not important in a particular case may appear. Conversely, the absence of alerts does not guarantee there are no aspects of the results needing attention. It is up to the individual to critically assess their own results and, if necessary, seek expert advice.

Publication of your CIF in IUCr journals

A basic structural check has been run on your CIF. These basic checks will be run on all CIFs submitted for publication in IUCr journals (*Acta Crystallographica*, *Journal of Applied Crystallography*, *Journal of Synchrotron Radiation*); however, if you intend to submit to *Acta Crystallographica Section C* or *E* or *IUCrData*, you should make sure that full publication checks are run on the final version of your CIF prior to submission.

Publication of your CIF in other journals

Please refer to the *Notes for Authors* of the relevant journal for any special instructions relating to CIF submission.

PLATON version of 20/01/2022; check.def file version of 19/01/2022

Datablock 3 - ellipsoid plot

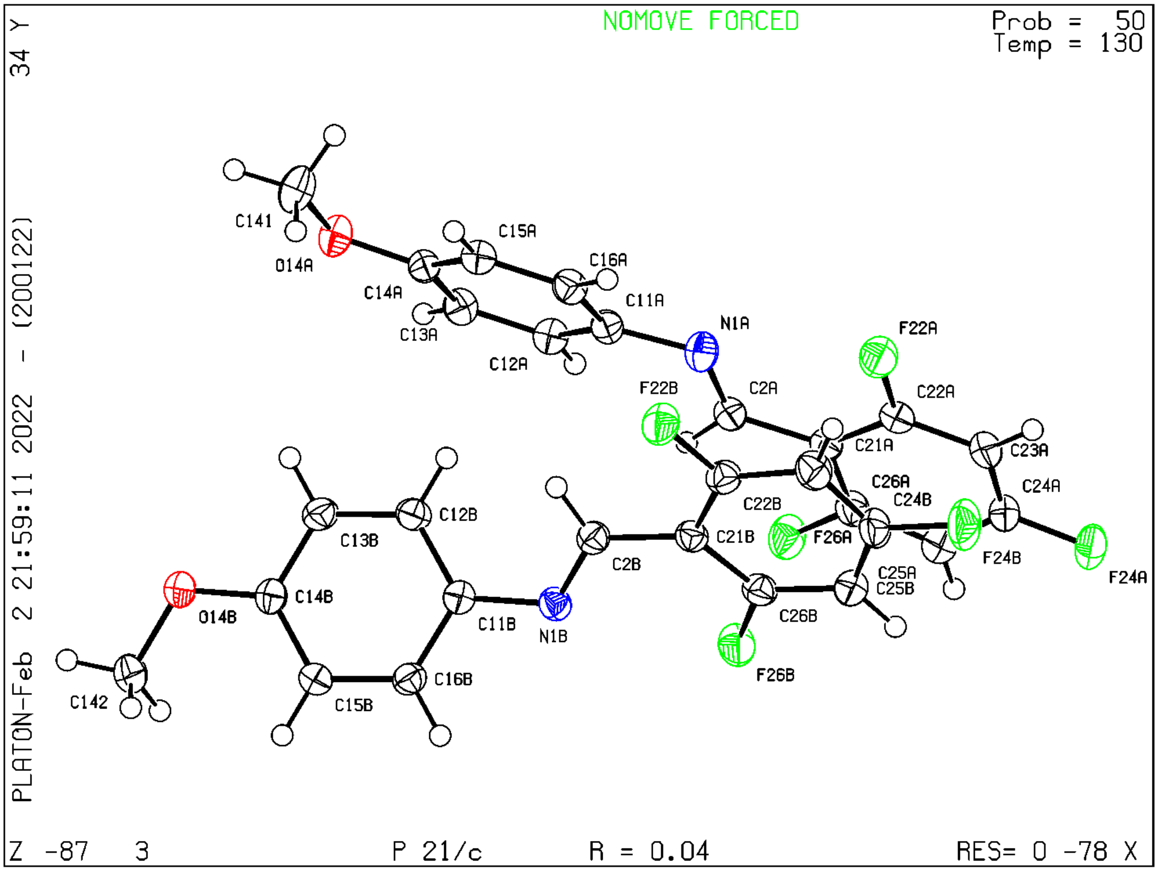

checkCIF/PLATON report

Structure factors have been supplied for datablock(s) 4

THIS REPORT IS FOR GUIDANCE ONLY. IF USED AS PART OF A REVIEW PROCEDURE FOR PUBLICATION, IT SHOULD NOT REPLACE THE EXPERTISE OF AN EXPERIENCED CRYSTALLOGRAPHIC REFEREE.

No syntax errors found. CIF dictionary Interpreting this report

Datablock: 4

```
Bond precision:   C-C = 0.0017 A           Wavelength=0.71073

Cell:           a=6.4217(3)       b=7.3068(4)       c=12.8876(6)
                alpha=85.930(4)   beta=80.022(4)    gamma=80.491(4)
Temperature:    100 K

                 Calculated                   Reported
Volume           586.82(5)                    586.82(5)
Space group      P -1                         P -1
Hall group       -P 1                         -P 1
Moiety formula   C14 H8 F5 N O                C14 H8 F5 N O
Sum formula      C14 H8 F5 N O                C14 H8 F5 N O
Mr               301.21                       301.21
Dx,g cm-3        1.705                        1.705
Z                2                            2
Mu (mm-1)        0.162                        0.162
F000             304.0                        304.0
F000'            304.25
h,k,lmax         8,9,17                       8,9,17
Nref             3019                         2626
Tmin,Tmax        0.962,0.984                  0.913,1.000
Tmin'            0.960

Correction method= # Reported T Limits: Tmin=0.913 Tmax=1.000
AbsCorr = MULTI-SCAN

Data completeness= 0.870           Theta(max)= 28.647

R(reflections)= 0.0350( 2163)                          wR2(reflections)=
                                                       0.1035( 2626)
S = 1.047                    Npar= 191
```

The following ALERTS were generated. Each ALERT has the format
test-name_ALERT_alert-type_alert-level.
Click on the hyperlinks for more details of the test.

Alert level C

PLAT910_ALERT_3_C Missing # of FCF Reflection(s) Below Theta(Min). 7 Note

Alert level G

PLAT154_ALERT_1_G The s.u.'s on the Cell Angles are Equal ..(Note) 0.004 Degree
PLAT912_ALERT_4_G Missing # of FCF Reflections Above STh/L= 0.600 386 Note
PLAT941_ALERT_3_G Average HKL Measurement Multiplicity 3.6 Low
PLAT978_ALERT_2_G Number C-C Bonds with Positive Residual Density. 18 Info

0 **ALERT level A** = Most likely a serious problem - resolve or explain
0 **ALERT level B** = A potentially serious problem, consider carefully
1 **ALERT level C** = Check. Ensure it is not caused by an omission or oversight
4 **ALERT level G** = General information/check it is not something unexpected

1 ALERT type 1 CIF construction/syntax error, inconsistent or missing data
1 ALERT type 2 Indicator that the structure model may be wrong or deficient
2 ALERT type 3 Indicator that the structure quality may be low
1 ALERT type 4 Improvement, methodology, query or suggestion
0 ALERT type 5 Informative message, check

It is advisable to attempt to resolve as many as possible of the alerts in all categories. Often the minor alerts point to easily fixed oversights, errors and omissions in your CIF or refinement strategy, so attention to these fine details can be worthwhile. In order to resolve some of the more serious problems it may be necessary to carry out additional measurements or structure refinements. However, the purpose of your study may justify the reported deviations and the more serious of these should normally be commented upon in the discussion or experimental section of a paper or in the "special_details" fields of the CIF. checkCIF was carefully designed to identify outliers and unusual parameters, but every test has its limitations and alerts that are not important in a particular case may appear. Conversely, the absence of alerts does not guarantee there are no aspects of the results needing attention. It is up to the individual to critically assess their own results and, if necessary, seek expert advice.

Publication of your CIF in IUCr journals

A basic structural check has been run on your CIF. These basic checks will be run on all CIFs submitted for publication in IUCr journals (*Acta Crystallographica*, *Journal of Applied Crystallography*, *Journal of Synchrotron Radiation*); however, if you intend to submit to *Acta Crystallographica Section C* or *E* or *IUCrData*, you should make sure that full publication checks are run on the final version of your CIF prior to submission.

Publication of your CIF in other journals

Please refer to the *Notes for Authors* of the relevant journal for any special instructions relating to CIF submission.

PLATON version of 20/01/2022; check.def file version of 19/01/2022

Datablock 4 - ellipsoid plot

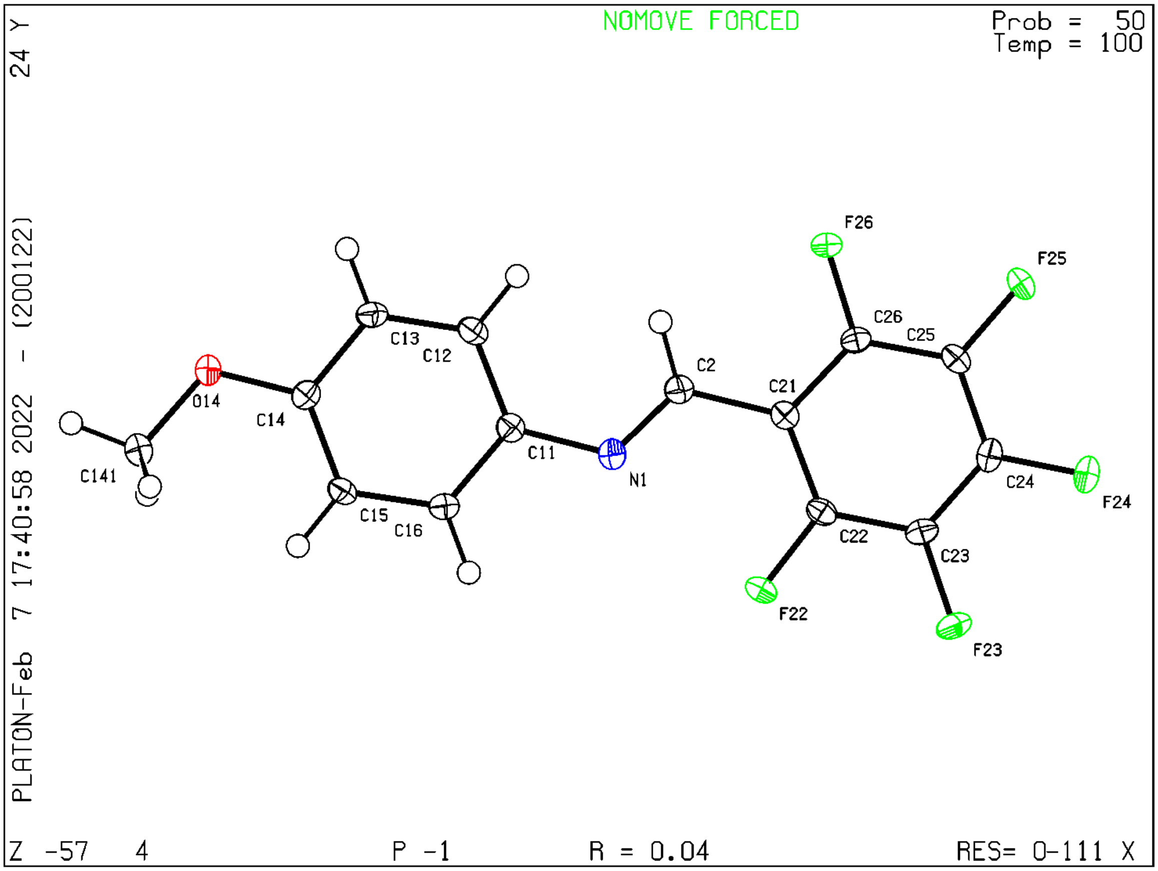

Display Report

Acquisition Parameter

Source Type	ESI	Ion Polarity	Positive	Set Nebulizer	0.3 Bar
Focus	Active	Set Capillary	4200 V	Set Dry Heater	200 °C
Scan Begin	50 m/z	Set End Plate Offset	-500 V	Set Dry Gas	4.0 l/min
Scan End	500 m/z	Set Charging Voltage	2000 V	Set Divert Valve	Source
		Set Corona	0 nA	Set APCI Heater	0 °C

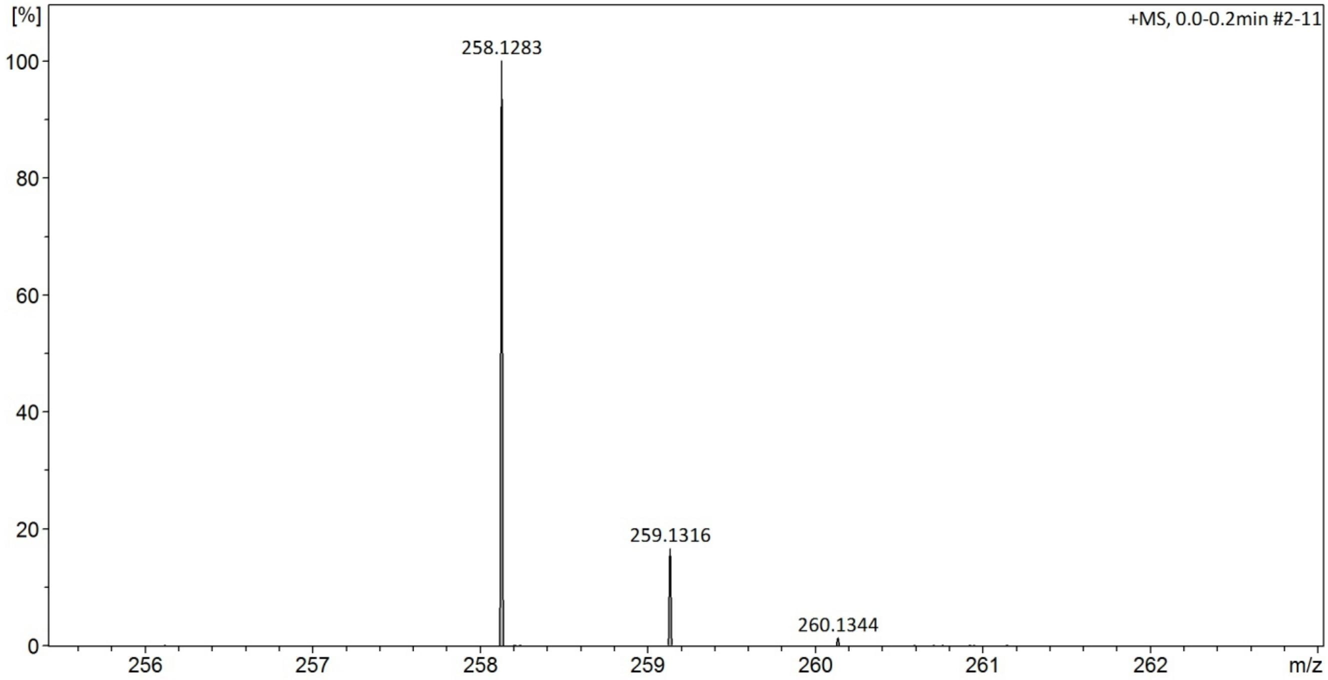

HRMS spectrum of 1h

Display Report

Acquisition Parameter

Source Type	ESI	Ion Polarity	Positive	Set Nebulizer	0.3 Bar
Focus	Active	Set Capillary	5200 V	Set Dry Heater	200 °C
Scan Begin	50 m/z	Set End Plate Offset	-500 V	Set Dry Gas	4.0 l/min
Scan End	500 m/z	Set Charging Voltage	2000 V	Set Divert Valve	Source
		Set Corona	0 nA	Set APCI Heater	0 °C

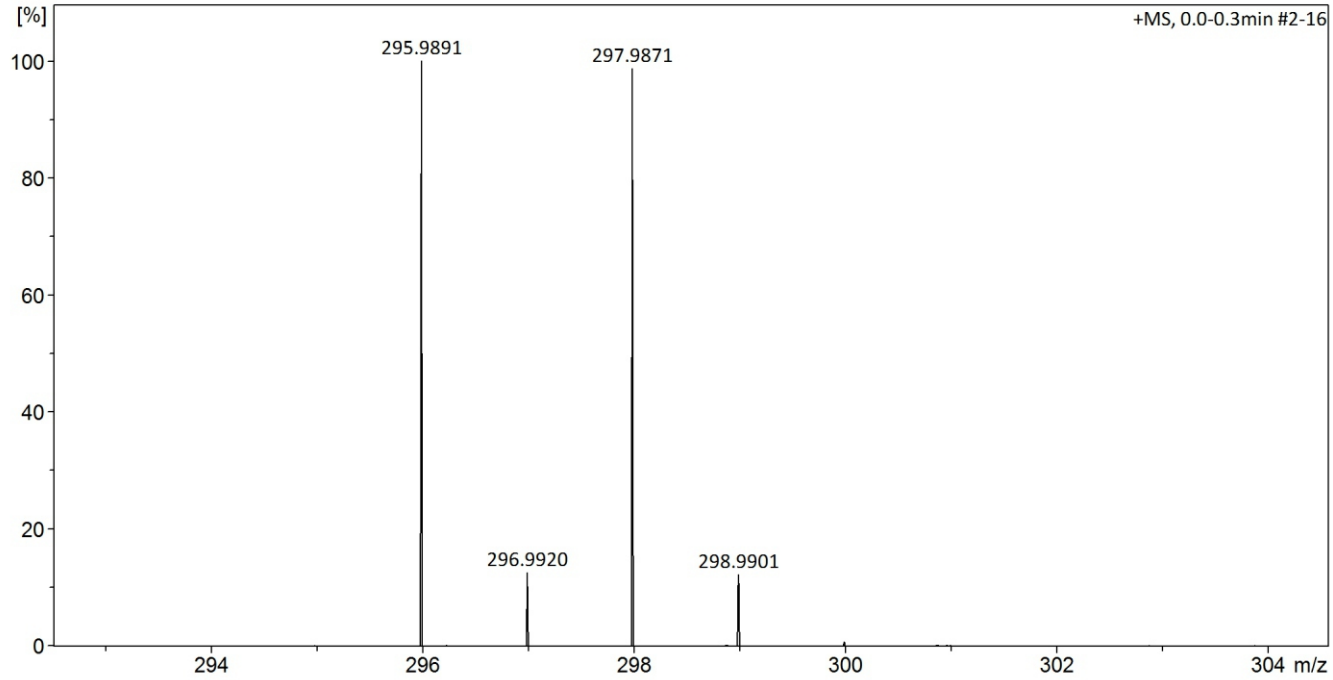

HRMS spectrum of 2c

Display Report

Acquisition Parameter

Source Type	ESI	Ion Polarity	Positive	Set Nebulizer	0.3 Bar
Focus	Active	Set Capillary	4200 V	Set Dry Heater	200 °C
Scan Begin	50 m/z	Set End Plate Offset	-500 V	Set Dry Gas	4.0 l/min
Scan End	500 m/z	Set Charging Voltage	2000 V	Set Divert Valve	Source
		Set Corona	0 nA	Set APCI Heater	0 °C

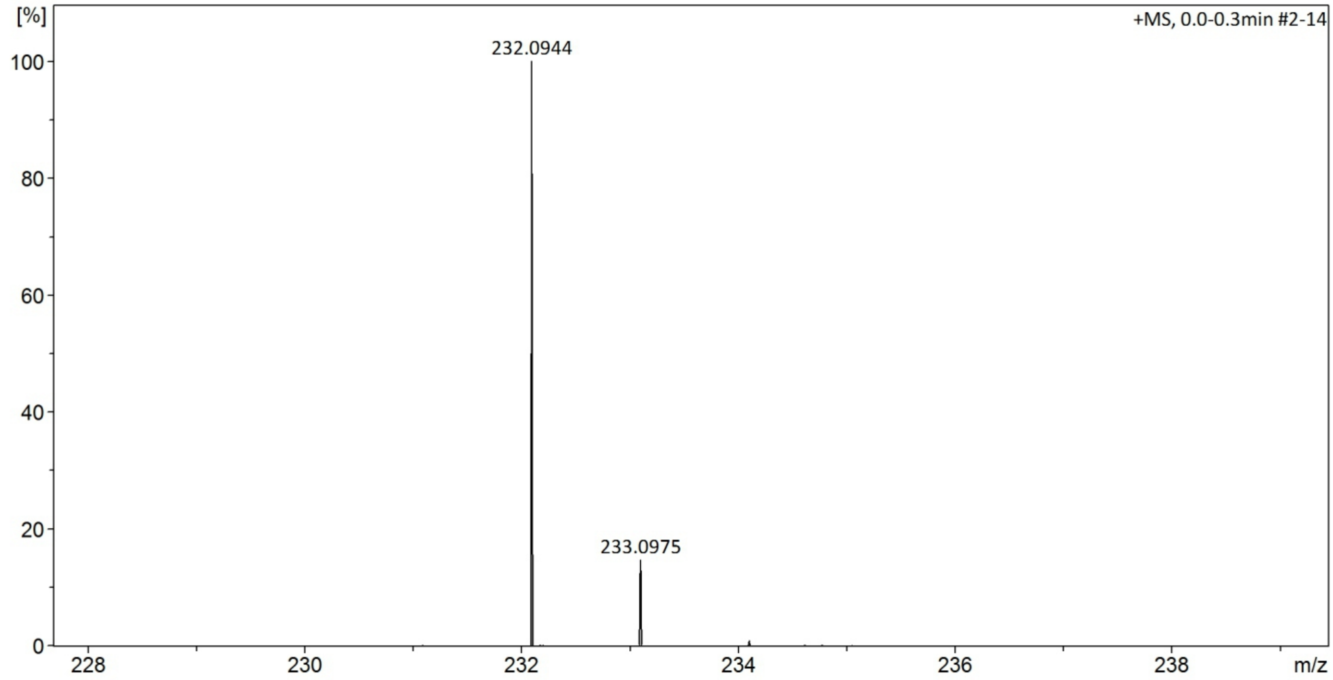

HRMS spectrum of 2f

Display Report

Acquisition Parameter

Source Type	ESI	Ion Polarity	Positive	Set Nebulizer	0.3 Bar
Focus	Active	Set Capillary	4200 V	Set Dry Heater	200 °C
Scan Begin	50 m/z	Set End Plate Offset	-500 V	Set Dry Gas	4.0 l/min
Scan End	500 m/z	Set Charging Voltage	2000 V	Set Divert Valve	Source
		Set Corona	0 nA	Set APCI Heater	0 °C

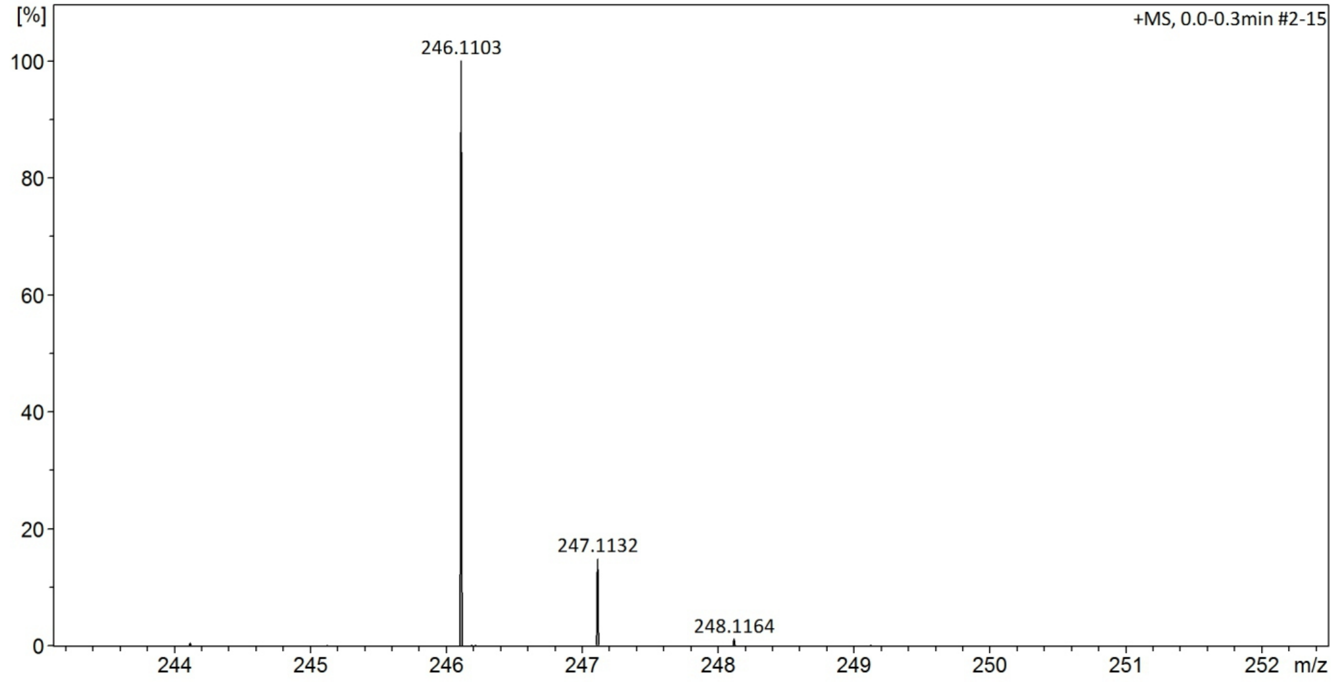

HRMS spectrum of 2g

Display Report

Acquisition Parameter

Source Type	ESI	Ion Polarity	Positive	Set Nebulizer	0.3 Bar
Focus	Active	Set Capillary	5200 V	Set Dry Heater	200 °C
Scan Begin	50 m/z	Set End Plate Offset	-500 V	Set Dry Gas	4.0 l/min
Scan End	500 m/z	Set Charging Voltage	2000 V	Set Divert Valve	Source
		Set Corona	0 nA	Set APCI Heater	0 °C

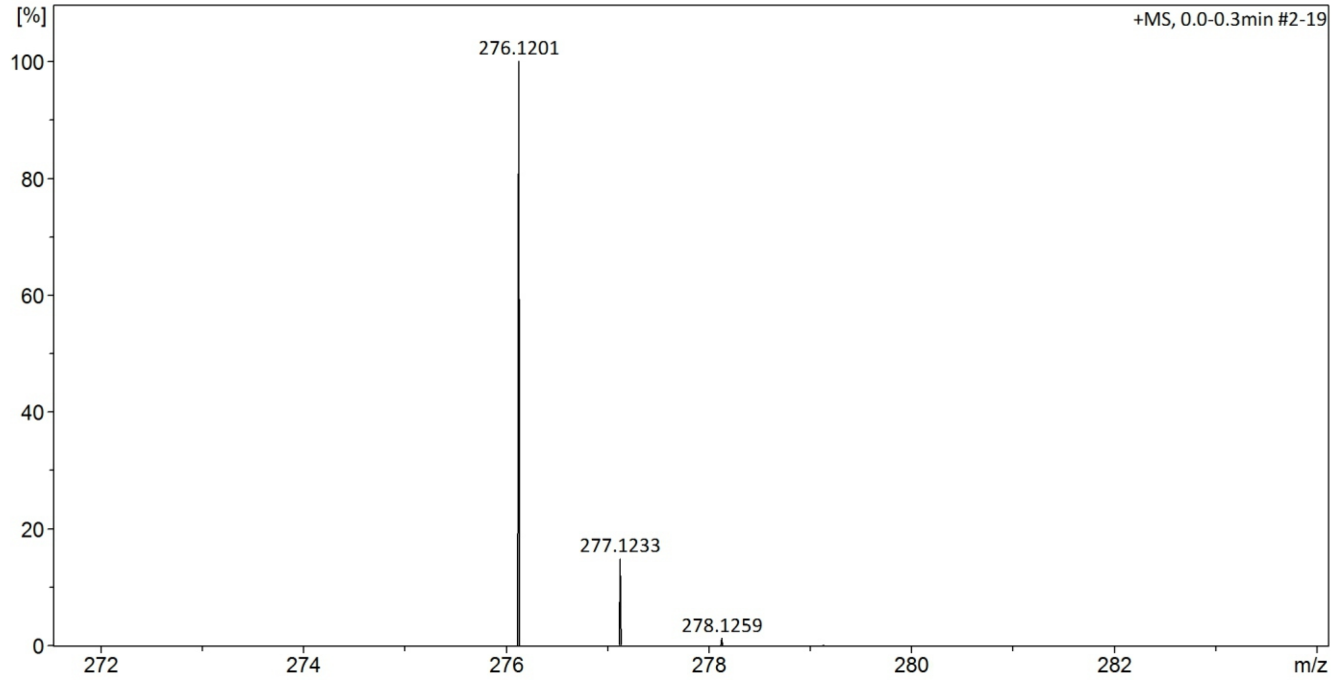

HRMS spectrum of 2h

Display Report

Acquisition Parameter

Source Type	ESI	Ion Polarity	Positive	Set Nebulizer	0.3 Bar
Focus	Active	Set Capillary	5200 V	Set Dry Heater	200 °C
Scan Begin	50 m/z	Set End Plate Offset	-500 V	Set Dry Gas	4.0 l/min
Scan End	500 m/z	Set Charging Voltage	2000 V	Set Divert Valve	Source
		Set Corona	0 nA	Set APCI Heater	0 °C

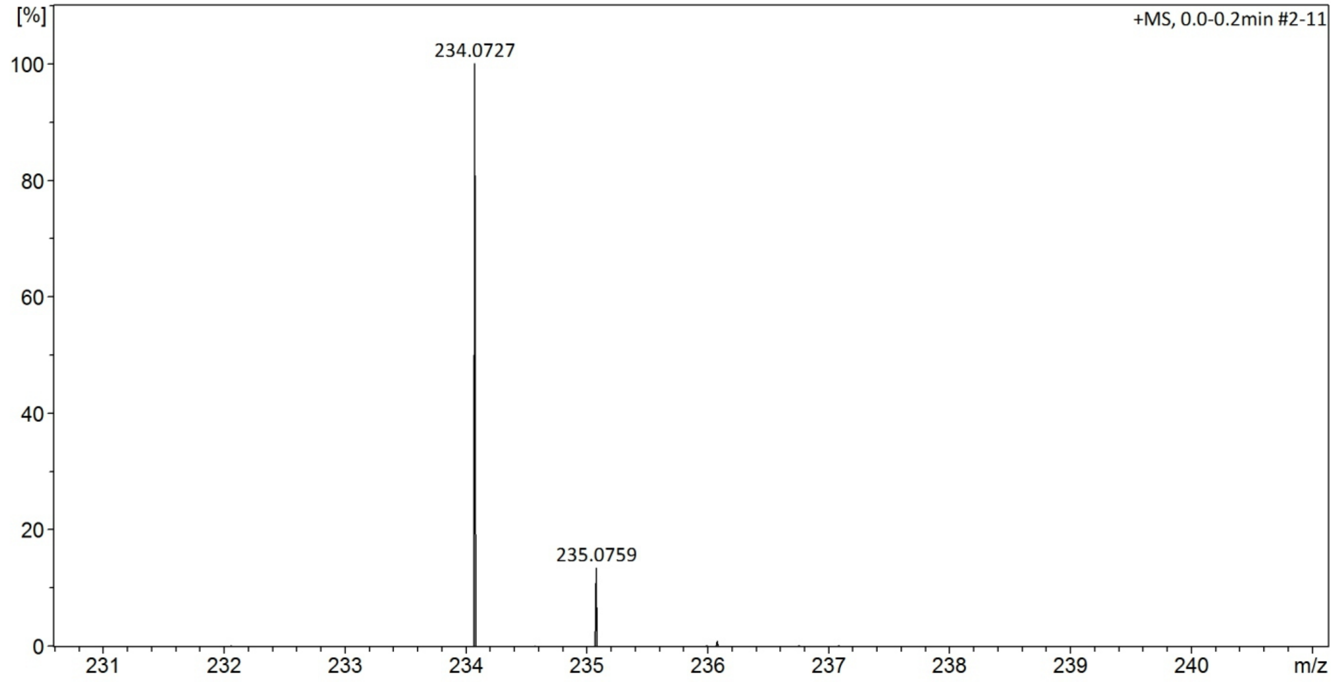

HRMS spectrum of 2i

Display Report

Acquisition Parameter

Source Type	ESI	Ion Polarity	Positive	Set Nebulizer	0.3 Bar
Focus	Active	Set Capillary	4200 V	Set Dry Heater	200 °C
Scan Begin	50 m/z	Set End Plate Offset	-500 V	Set Dry Gas	4.0 l/min
Scan End	500 m/z	Set Charging Voltage	2000 V	Set Divert Valve	Source
		Set Corona	0 nA	Set APCI Heater	0 °C

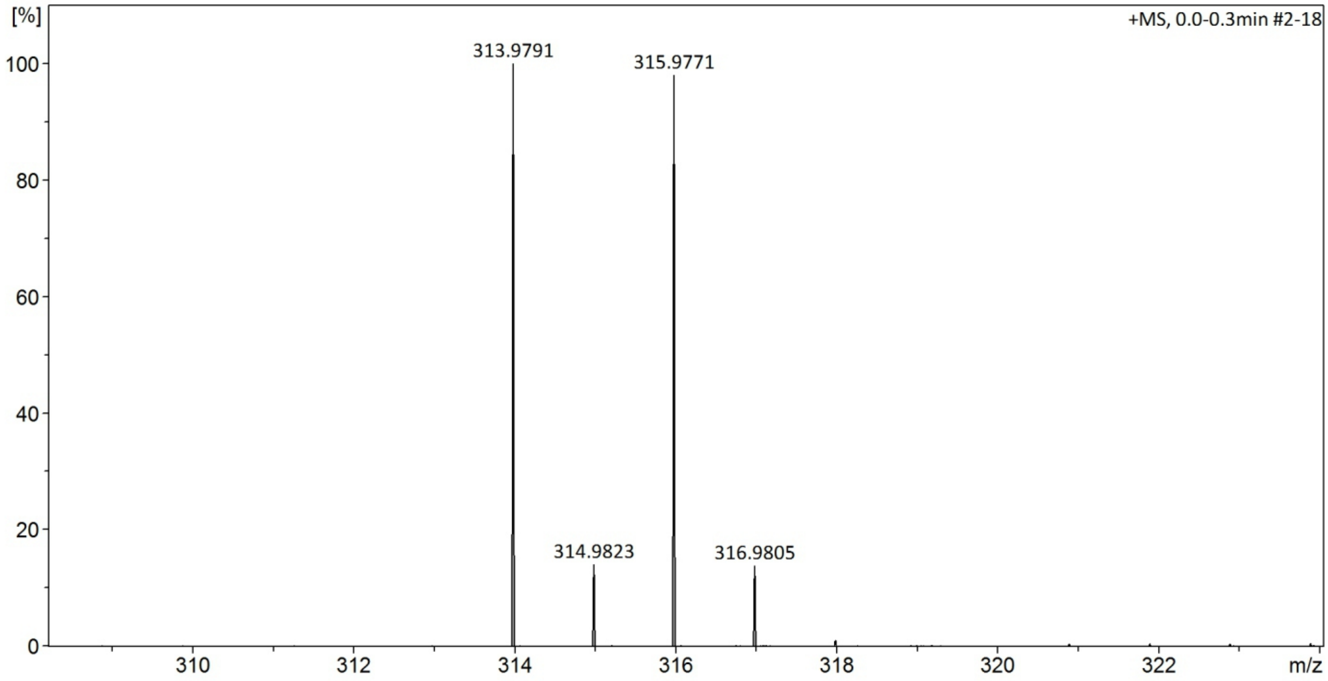

HRMS spectrum of 3c

Display Report

Acquisition Parameter

Source Type	ESI	Ion Polarity	Positive	Set Nebulizer	0.3 Bar
Focus	Active	Set Capillary	5200 V	Set Dry Heater	200 °C
Scan Begin	50 m/z	Set End Plate Offset	-500 V	Set Dry Gas	4.0 l/min
Scan End	500 m/z	Set Charging Voltage	2000 V	Set Divert Valve	Source
		Set Corona	0 nA	Set APCI Heater	0 °C

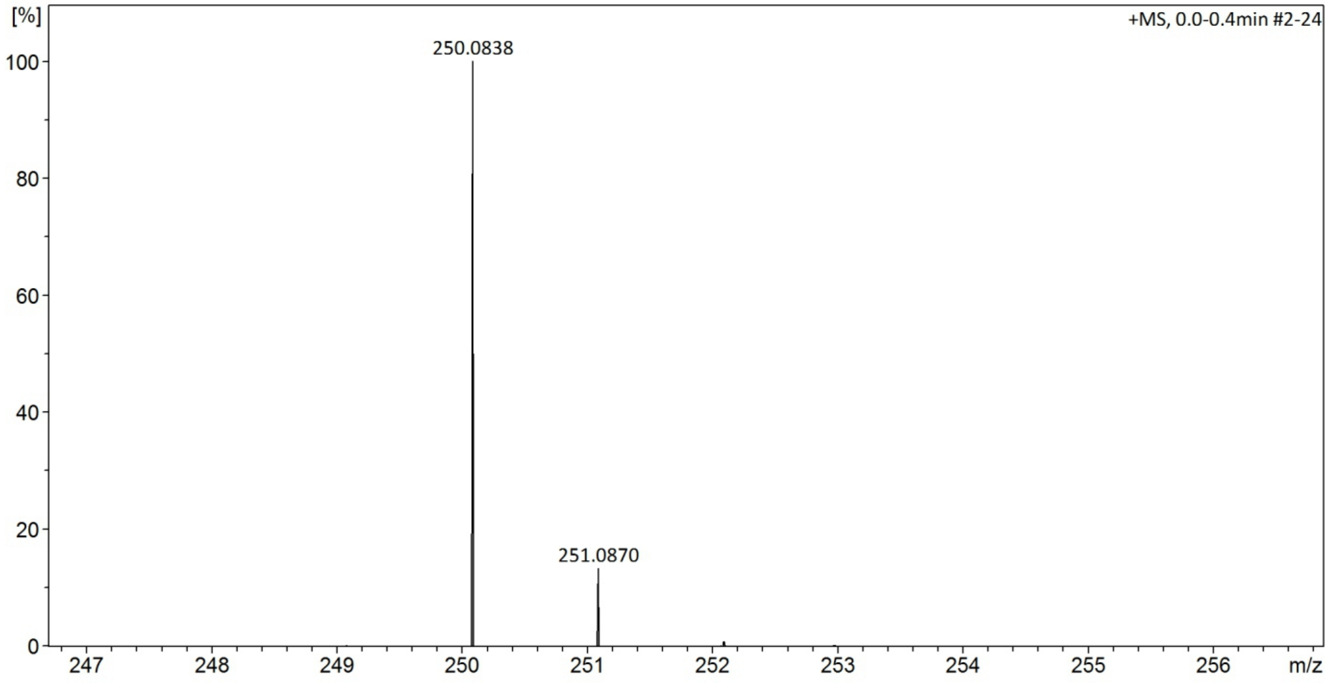

HRMS spectrum of 3e

Display Report

Acquisition Parameter

Source Type	ESI	Ion Polarity	Positive	Set Nebulizer	0.3 Bar
Focus	Active	Set Capillary	5200 V	Set Dry Heater	200 °C
Scan Begin	50 m/z	Set End Plate Offset	-500 V	Set Dry Gas	4.0 l/min
Scan End	500 m/z	Set Charging Voltage	2000 V	Set Divert Valve	Source
		Set Corona	0 nA	Set APCI Heater	0 °C

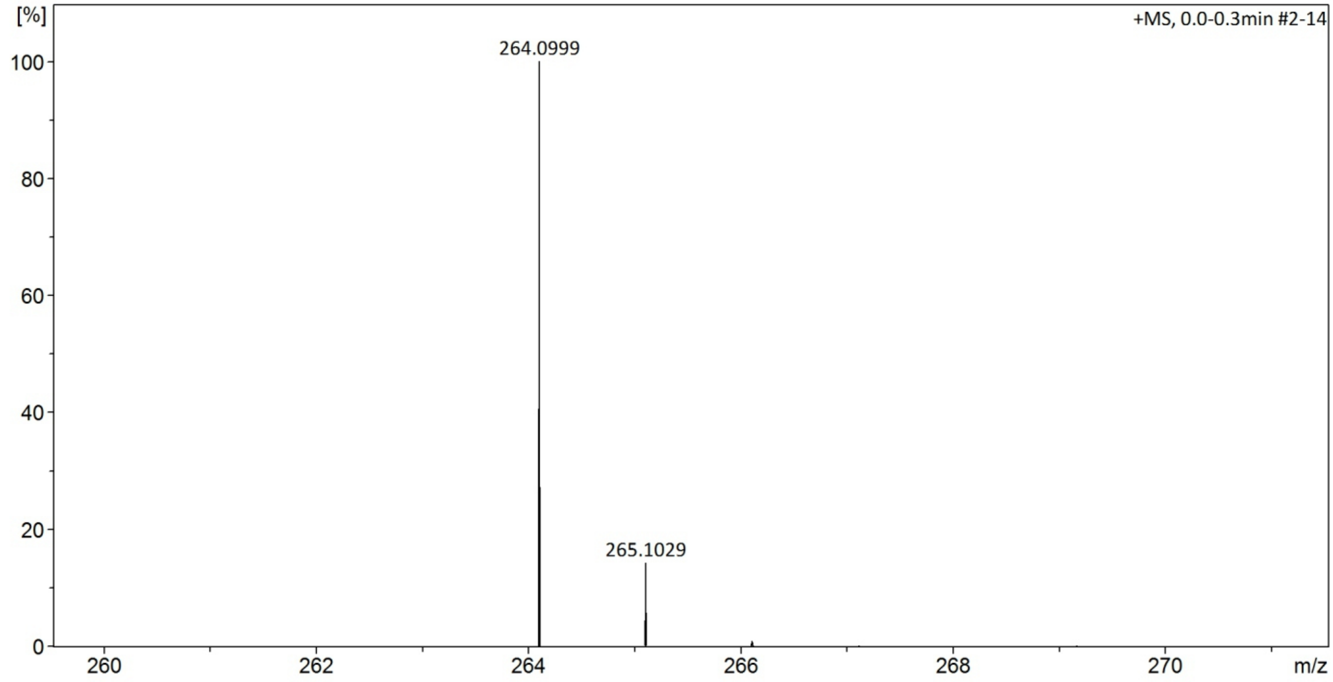

HRMS spectrum of 3g

Display Report

Acquisition Parameter

Source Type	ESI	Ion Polarity	Positive	Set Nebulizer	0.3 Bar
Focus	Active	Set Capillary	5200 V	Set Dry Heater	200 °C
Scan Begin	50 m/z	Set End Plate Offset	-500 V	Set Dry Gas	4.0 l/min
Scan End	500 m/z	Set Charging Voltage	2000 V	Set Divert Valve	Source
		Set Corona	0 nA	Set APCI Heater	0 °C

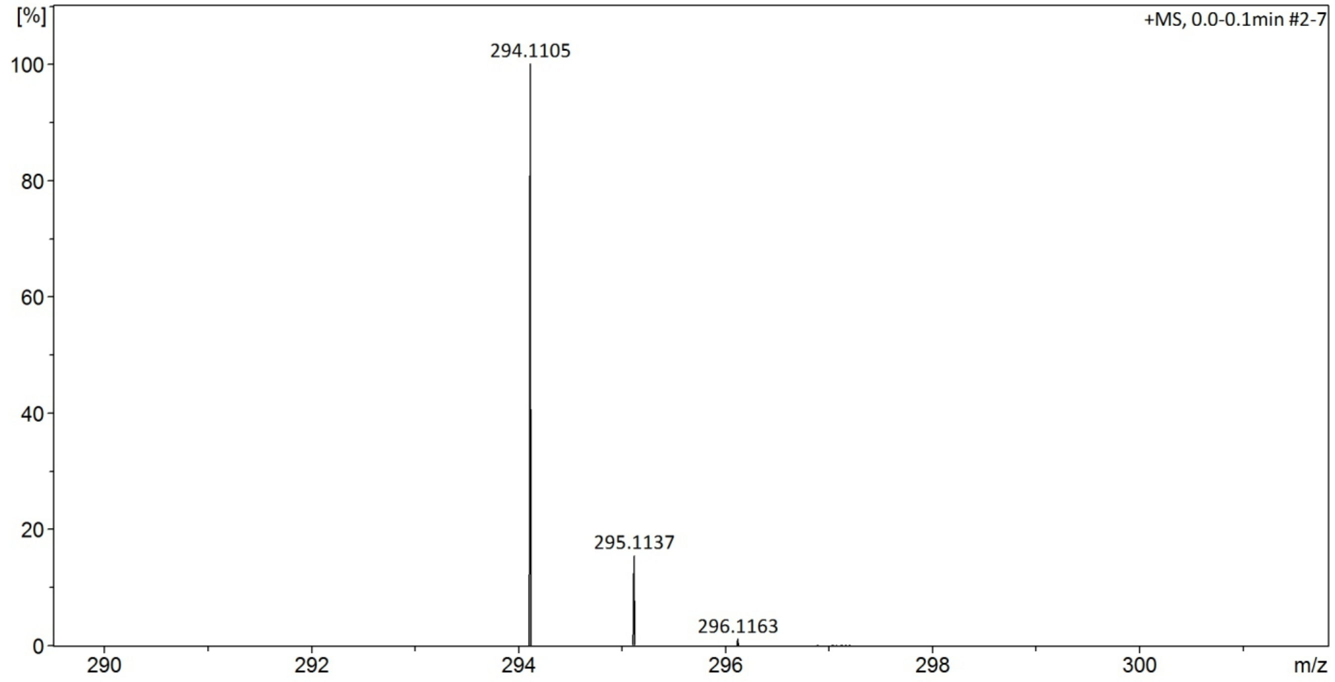

HRMS spectrum of 3h

Display Report

Acquisition Parameter

Source Type	ESI	Ion Polarity	Positive	Set Nebulizer	0.3 Bar
Focus	Active	Set Capillary	5200 V	Set Dry Heater	200 °C
Scan Begin	50 m/z	Set End Plate Offset	-500 V	Set Dry Gas	4.0 l/min
Scan End	500 m/z	Set Charging Voltage	2000 V	Set Divert Valve	Source
		Set Corona	0 nA	Set APCI Heater	0 °C

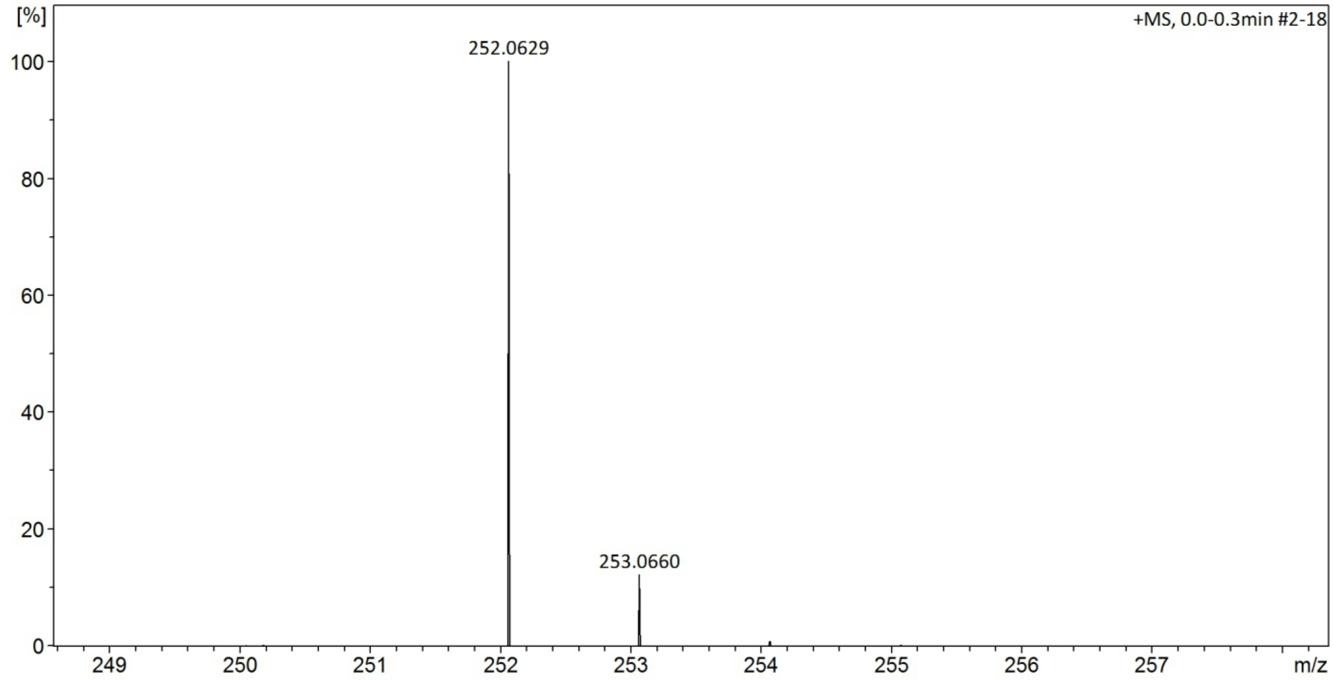

HRMS spectrum of 3i

Display Report

Acquisition Parameter

Source Type	ESI	Ion Polarity	Positive	Set Nebulizer	0.3 Bar
Focus	Active	Set Capillary	5200 V	Set Dry Heater	200 °C
Scan Begin	50 m/z	Set End Plate Offset	-500 V	Set Dry Gas	4.0 l/min
Scan End	500 m/z	Set Charging Voltage	2000 V	Set Divert Valve	Source
		Set Corona	0 nA	Set APCI Heater	0 °C

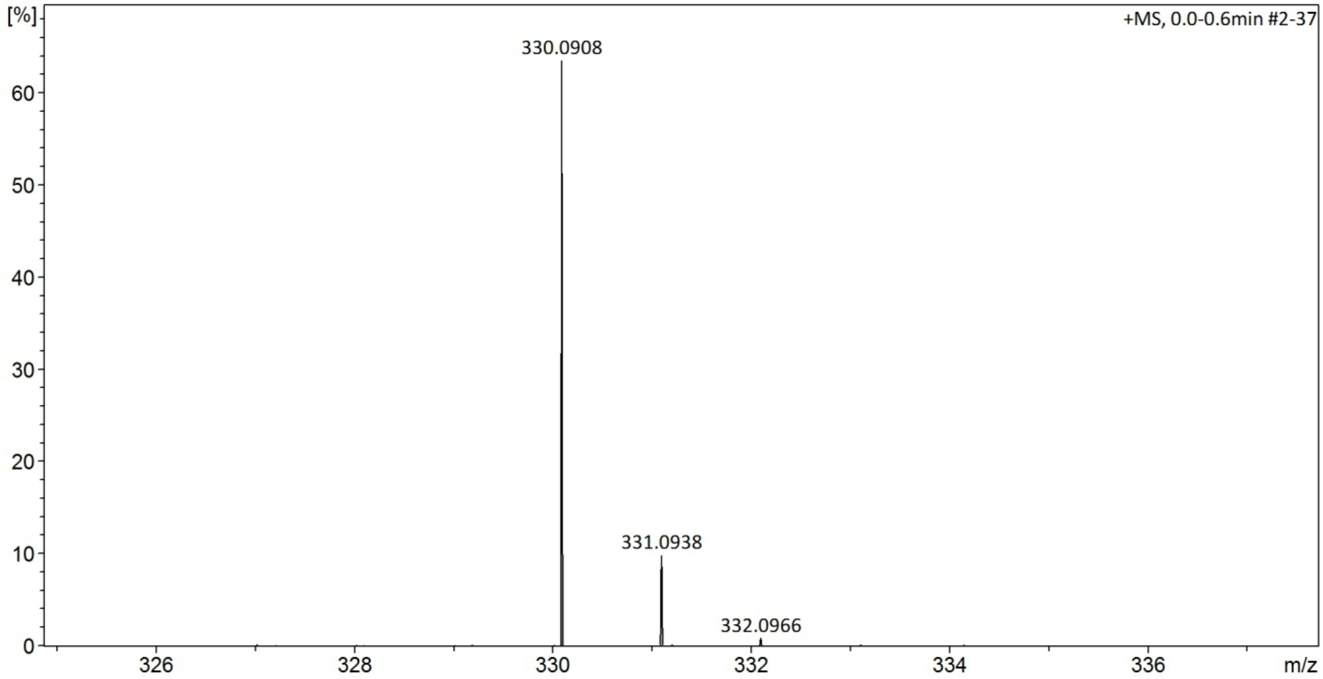

HRMS spectrum of 4h
